# Sulfoxide-mediated oxidative cross-coupling of phenols†

**DOI:** 10.1039/c9sc05668h

**Published:** 2020-01-15

**Authors:** Zhen He, Gregory J. P. Perry, David J. Procter

**Affiliations:** Department of Chemistry, University of Manchester Oxford Rd Manchester M13 9PL UK david.j.procter@manchester.ac.uk

## Abstract

A metal-free, oxidative coupling of phenols with various nucleophiles, including arenes, 1,3-diketones and other phenols, is reported. Cross-coupling is mediated by a sulfoxide which inverts the reactivity of the phenol partner. Crucially, the process shows high selectivity for cross-*versus* homo-coupling and allows efficient access to a variety of aromatic scaffolds including biaryls, benzofurans and, through an iterative procedure, aromatic oligomers.

## Abstract 2

A metal-free, oxidative coupling of phenols with various nucleophiles, including arenes, 1,3-diketones and other phenols, is reported.

## Graphical abstract

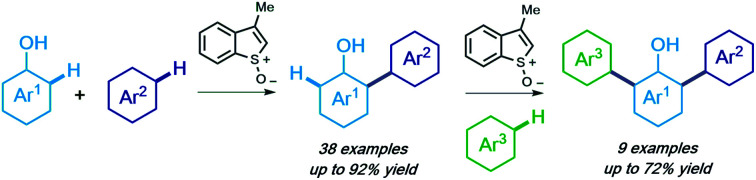

## Introduction

**Scheme 1:**
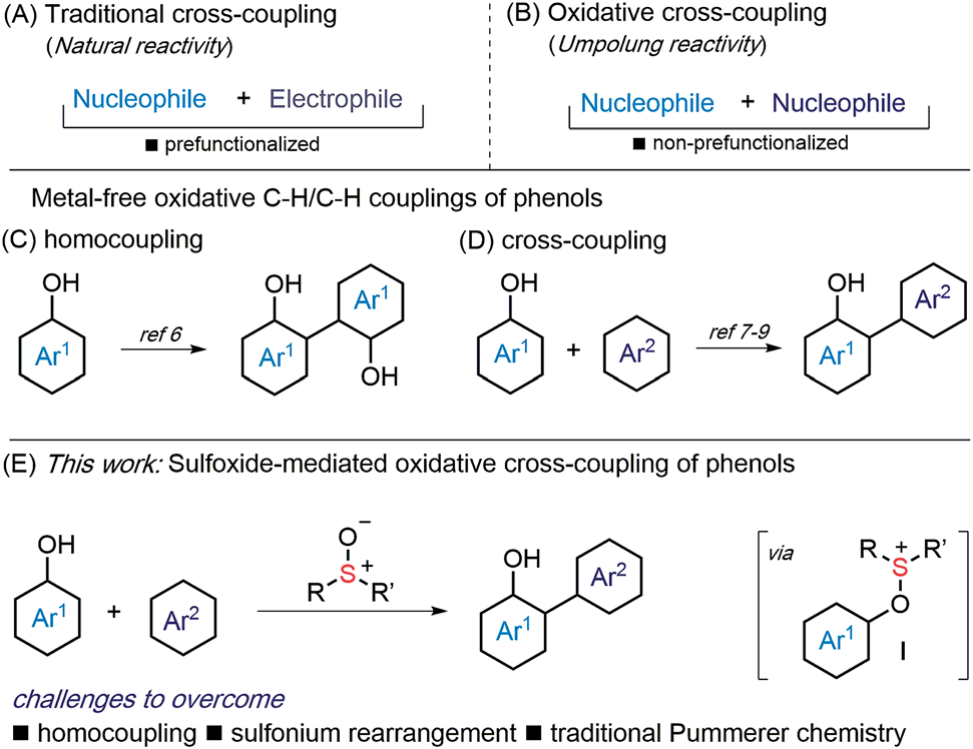
(A and B) Types of cross-coupling. (C and D) Metal-free, oxidative coupling of phenols. (E) Sulfoxide-mediated, oxidative coupling of phenols.

Metal-catalyzed cross-coupling, involving an aryl halide and an organometallic partner, is a powerful tool for biaryl synthesis (Scheme 1A).^1^ However, oxidative, C–H/C–H couplings, involving non-prefunctionalized partners, have recently come to the fore as an attractive alternative (Scheme 1B).^2^ Their development remains a challenge, as the reactivity of one partner must be inverted, and known processes are compromised by the requirement for expensive, supply risk, metal oxidants or metal catalysts.^2^ The development of selective, metal-free C–H/C–H coupling reactions is, therefore, an important goal.^3^

Phenols, in particular unsymmetrical phenol-derived biaryls, are ubiquitous in nature, biomaterials and ligand collections for catalysis.^4^ Approaches to these compounds generally require multiple steps – prefunctionalization of partners or manipulation of protecting groups – and/or the use of metals.^5^ Metal-free oxidative coupling of unprotected phenols is therefore of interest, however, avoiding homocoupling is a challenge (Scheme 1C).^6^ Nevertheless, metal-free cross-coupling of phenols has been described, most notably using electroorganic synthesis^7^ or hypervalent iodine reagents,^8^ amongst other approaches^9^ (Scheme 1D).

We proposed that sulfoxides^10,11^ could be used to invert the reactivity of a phenol partner, thus providing an alternative approach to their oxidative coupling (Scheme 1E). Capture of phenols by sulfoxides will deliver aryloxysulfonium intermediates **I** that are electrophilic and capable of coupling with various nucleophiles (*e.g.* Ar^2^).^12–14^ The major challenge in such an approach is the avoidance of homocoupling.^13^ Furthermore, alternative Pummerer chemistry of the sulfoxide^15^ and rearrangement of sulfonium intermediates **I**^9*a*,10,16^ must be by-passed.

Here we describe the metal-free, oxidative cross-coupling of phenols with various carbon nucleophilic partners, including other phenols, arenes, and 1,3-diketones (Scheme 1E). Couplings deliver biaryls, 2-aryl 1,3-dicarbonyl compounds and benzofurans. An iterative procedure allows selective double functionalization of phenols and the preparation of aryl oligomers.

## Results and discussion

### Oxidative cross-coupling of phenols with phenols, phenol derivatives and arenes

Guided by our previous studies, phenol **1a** in CH_2_Cl_2_ was treated with sulfoxide **4a**, activated using trifluoroacetic anhydride (TFAA), before subsequent addition of **2a** (1.5 equivalents), to give the product of cross-coupling **3a** in 91% isolated yield (see the ESI† for optimisation).

**Scheme 2:**
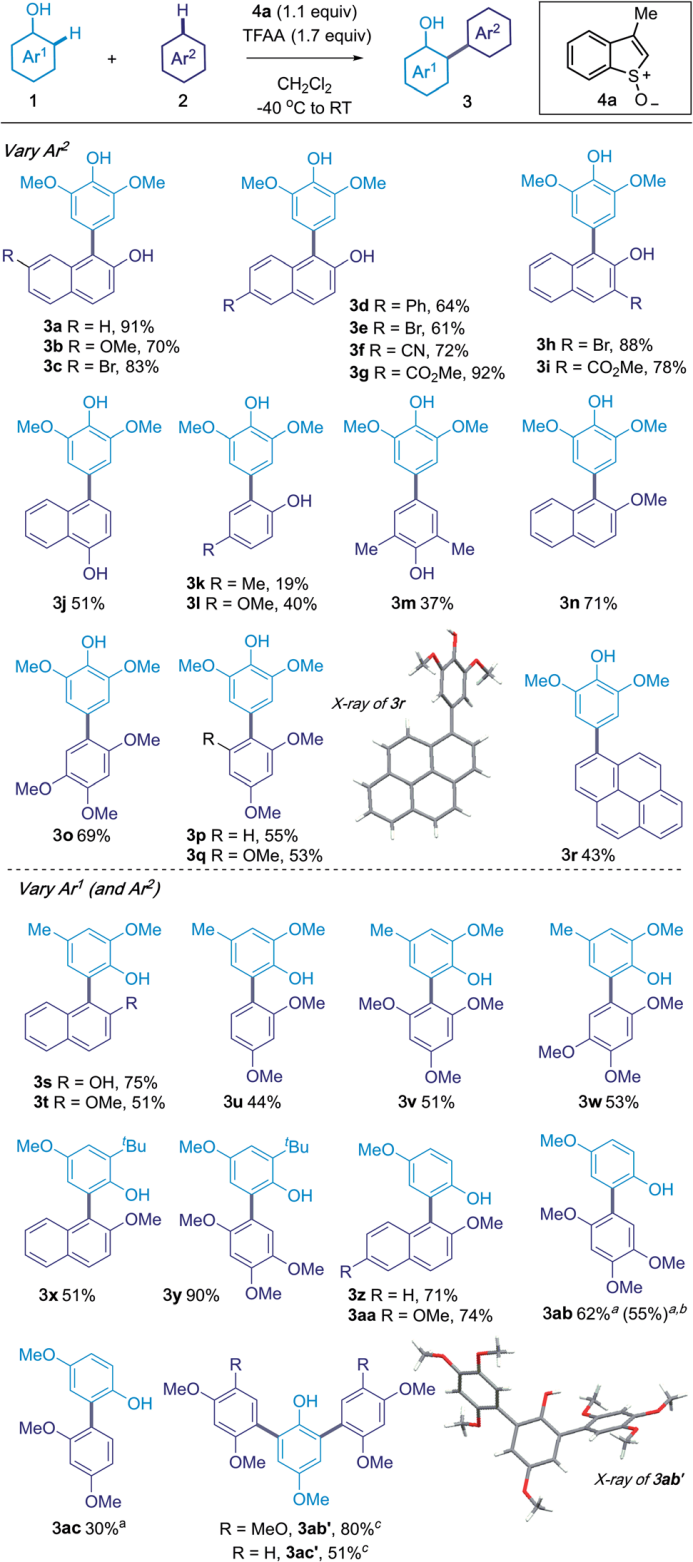
Oxidative cross-coupling of phenols with phenols, phenol derivatives and arenes. Reaction conditions: to sulfoxide **4a** (0.11 mmol) in CH_2_Cl_2_ (1 mL, 0.1 M) in an oven-dried tube flushed with N_2_ at −40 °C was added TFAA (0.17 mmol, 1.7 equiv.). After 5 min, phenol **1** (0.1 mmol in 0.5 mL CH_2_Cl_2_) was added in one portion. Arene **2** (0.15 mmol in 0.5 mL CH_2_Cl_2_) was then added immediately. After 15 min at −40 °C, the mixture was warmed to room temperature and stirred for 2 h. ^*a*^ CH_2_Cl_2_/TFA (1 : 1) as solvent. ^*b*^ Larger scale: (1.2 g of **1** was used). ^*c*^ 2 equiv. of **2** and 2.2 equiv. of **4a**.

2-Naphthols bearing bromo (**3c**, **3e**, **3h**), methoxy (**3b**), phenyl (**3d**), cyano (**3f**) and ester (**3g**, **3i**) groups at the 3-, 6- and 7-positions were found to be compatible with the coupling (Scheme 2). The process also embraced 1-naphthol (**3j**), phenols (**3k–3m**) and their methyl ether derivatives (**3n–3q**). Of particular note, pyrene (**3r**) underwent coupling with **1a** to give **3r**. The structure of **3r** was confirmed by X-ray crystallographic analysis.^17^

The phenol coupling partner (Ar^1^) could also be varied and products of *ortho*-coupling with a range of nucleophilic partners gave products **3s–3ac** (30–90% yield). Interestingly, treatment of 4-methoxyphenol with 1,2,4-trimethoxybenzene, under our standard conditions, gave the product of double arylation **3ab′** in 46% yield. The yield of **3ab′** could be increased by using 2.2 equivalent of the sulfoxide **4a** and 2.0 equivalents of 1,2,4-trimethoxybenzene (80%). Diarylated compound **3ac′** could also be obtained. Interestingly, the couplings could be tuned to favour products of mono- or bis-coupling; using CH_2_Cl_2_/TFA (1 : 1) as solvent favoured formation of the mono-arylated products **3ab** and **3ac**. Finally, the oxidative coupling could be carried out on a gram scale; the use of 1.2 g of 4-methoxyphenol produced 1.6 g of **3ab** (55% isolated yield). In all cross-couplings, 3-methyl benzothiophene was recovered in high yield by chromatography and could be reused.

### Oxidative coupling of phenols with 1,3-diketones

**Scheme 3:**
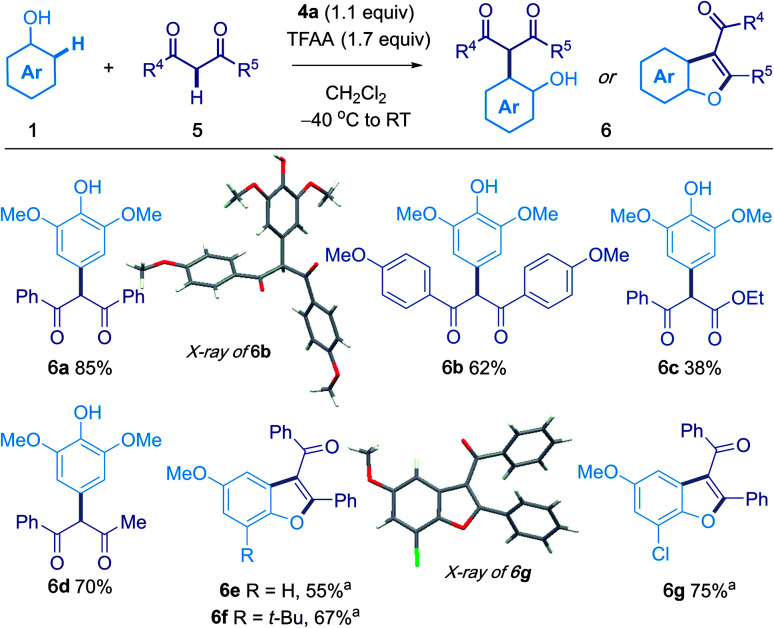
Oxidative coupling of phenols with 1,3-diketones. Reaction conditions: to sulfoxide **4a** (0.11 mmol) in CH_2_Cl_2_ (1 mL, 0.1 M) in an oven dried tube flushed with N_2_ at −40 °C was added TFAA (0.17 mmol, 1.7 equiv.). After 5 min, phenol **1** (0.1 mmol in 0.5 mL CH_2_Cl_2_) was added in one portion. 1,3-Dicarbonyl 5 (0.15 mmol in 0.5 mL CH_2_Cl_2_) was then added immediately. After 15 min at −40 °C, the mixture was warmed to room temperature and stirred for 2 h. ^a^ CH_2_Cl_2_/TFA (1 : 1) as solvent.

1,3-Dicarbonyl compounds could be used as the second nucleophilic partner (Scheme 3). For example, treatment of **1a** with 1,3-diphenylpropane-1,3-dione afforded **6a** in 85% yield. The products of *ortho* coupling underwent cyclization to give benzofuran products; for example, the use of 4-methoxyphenol gave aroyl[*b*]benzofuran^18^**6e** in 55% isolated yield.

### Iterative coupling of three nucleophiles

**Scheme 4:**
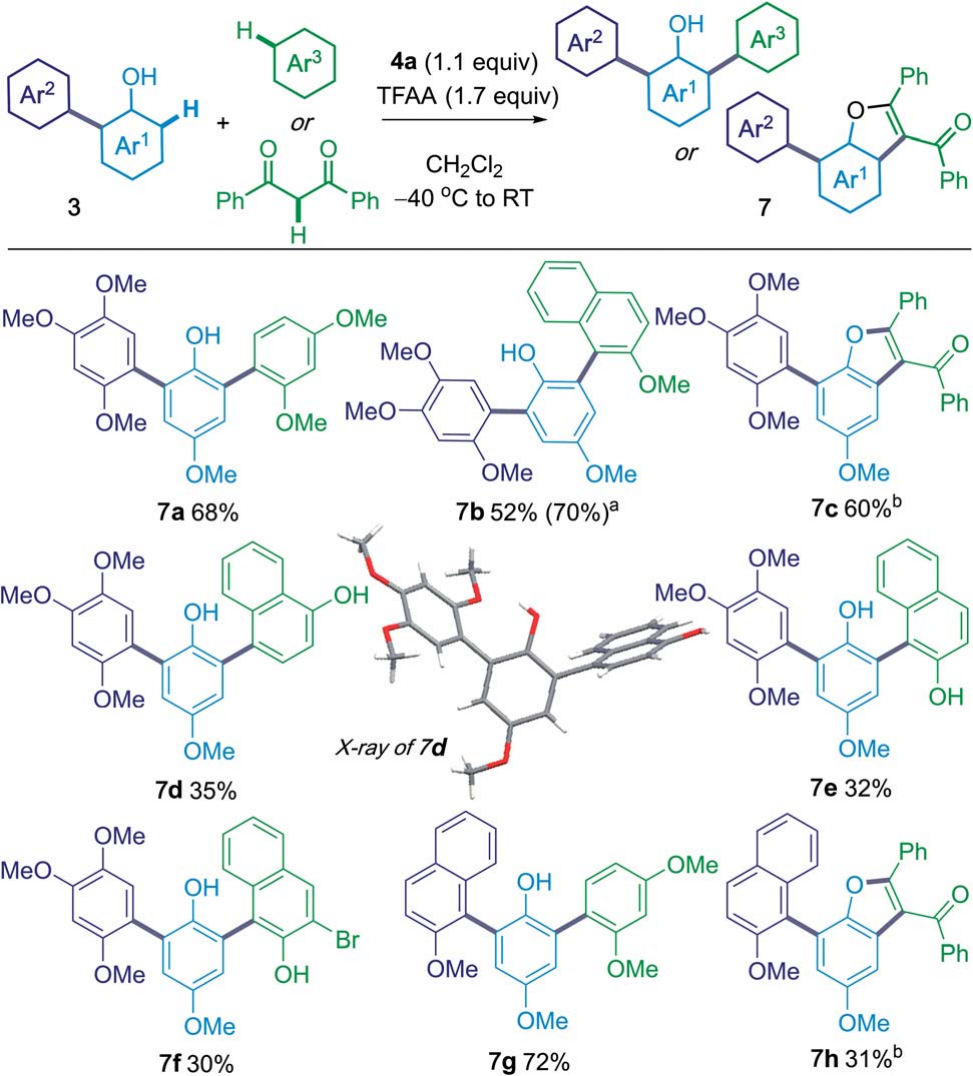
Iterative coupling of three nucleophiles. Reaction conditions: to sulfoxide **4a** (0.11 mmol) in CH_2_Cl_2_ (1 mL, 0.1 M) in an oven dried tube flushed with N_2_ at −40 °C was added TFAA (0.17 mmol, 1.7 equiv.). After 5 min, **3** (0.1 mmol in 0.5 mL CH_2_Cl_2_) was added in one portion. A third nucleophile (0.15 mmol in 0.5 mL CH_2_Cl_2_) was then added immediately. After 15 min at −40 °C, the mixture was warmed to room temperature and stirred for 2 h. ^a^ Compound **3z** was used as the substrate. ^b^ CH_2_Cl_2_/TFA (1 : 1) as solvent.

Intrigued by the formation of the triaryl products **3ab′** and **3ac′** (Scheme 2), we considered an iterative process that would allow the sequential, metal-free, oxidative coupling of phenols with two different nucleophilic partners (Scheme 4). For example, 4-methoxy phenol was first coupled with 1,2,4-trimethoxybenzene to afford **3ab**. Subsequent treatment of **3ab** with 1,3-dimethoxybenzene gave the unsymmetrical, diarylated phenol **7a** in 68% yield. 1,3-Diphenylpropane-1,3-dione could also be used as the third nucleophilic partner and gave C7-arylated benzofurans **7c** and **7h**.^19^

### Mechanistic studies

**Scheme 5:**
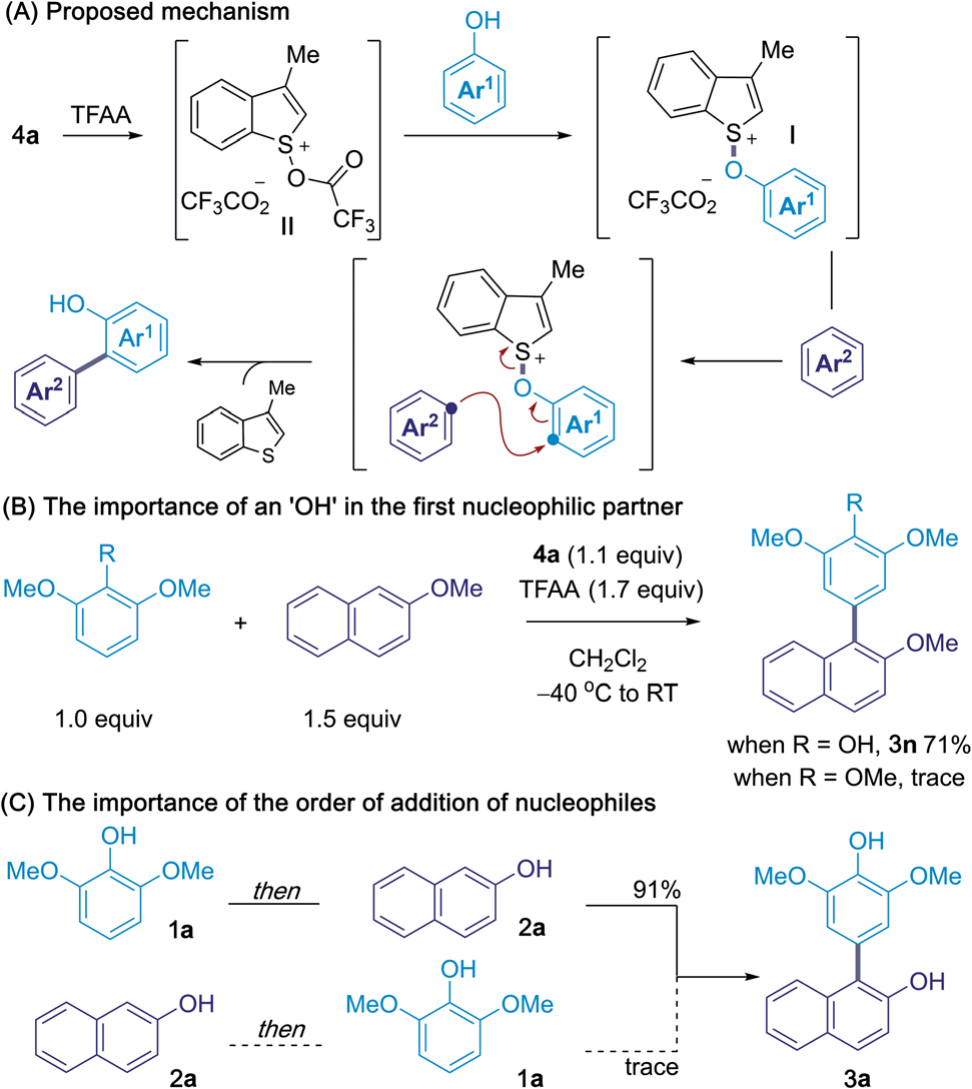
Proposed mechanism and support for the intermediacy of an aryloxysulfonium salt.

Based on the above results, and our previous studies,^10,13^ a possible mechanism for the oxidative cross-coupling is shown in Scheme 5A.^13^ Activation of sulfoxide **4a** with TFAA gives acyloxysulfonium salt **II** and interrupted Pummerer reaction with a phenol coupling partner gives aryloxysulfonium salt **I**. Subsequent attack of the second partner, at the *ortho* or *para* position of the first, results in C–C bond formation and expulsion of 3-methylbenzothiophene. The control experiments in Scheme 5B highlight the important role of the hydroxy group in the first partner and suggest that activation of the phenol occurs *via* intermediate **I**. However, we were unable to detect or isolate this intermediate and further studies are needed to confirm the exact mechanism for phenol activation. Scheme 5C shows that the order in which the two nucleophilic partners are combined can be critical, suggesting that rapid and irreversible, aryloxysulfonium salt formation takes place between the activated sulfoxide **I** and the first phenol partner, and that aryloxysulfonium salt intermediates have very different reactivities.^20^

## Conclusions

In summary, a metal-free, sulfoxide-mediated, oxidative cross-coupling unites phenols and various nucleophilic partners, including phenols, 1,3-diketones and arenes. The capture and inversion of reactivity of the first nucleophilic partner, using an interrupted Pummerer reaction, prior to coupling with the second nucleophile, is key to the cross-coupling. Homocoupling is not observed and alternative Pummerer and rearrangement processes are avoided. Iterative sulfoxide-mediated couplings allow the construction of polyaryl compounds.

## Conflicts of interest

There are no conflicts to declare.

## Supplementary Material

SC-011-C9SC05668H-s001

SC-011-C9SC05668H-s002
