# Supplementary material for: Sulfoxide-mediated oxidative cross-coupling of phenols
Source: Chem Sci. 2020 Jan 15;11(7):2001–5. doi: 10.1039/c9sc05668h (PMC8150100; doi:10.1039/c9sc05668h)

Electronic Supplementary Material (ESI) for Chemical Science.
This journal is © The Royal Society of Chemistry 2020

Table of Contents

General Experimental S2

Optimization of the Sulfoxide-Mediated Oxidative Cross-Coupling S3

General Procedure A. Oxidative Cross-Coupling of Phenols with Phenols, Phenol Derivatives and Arenes S4

General Procedure B. Oxidative Coupling of Phenols with 1,3-Diketones S21

General Procedure C. Iterative Addition of a Third Nucleophilic Partner S26

Mechanistic Studies S32

X-Ray Structures and CCDC Numbers S34

^{1}H and ^{13}C NMR Spectra of Compounds S44

General Experimental

All experiments were performed under an atmosphere of nitrogen, using anhydrous solvents, unless stated otherwise. THF was distilled from sodium/benzophenone. All other solvents and reagents were purchased from commercial sources and used as supplied. ^{1}H NMR spectra were recorded on NMR spectrometers at 400 MHz and 500 MHz and ^{13}C NMR at 100 MHz and 125 MHz. ^{1}H NMR chemical shifts (δ_H) and ^{13}C NMR chemical shifts (δ_C) are quoted in parts per million (ppm) downfield from trimethylsilane (TMS) and coupling constants (*J*) are quoted in Hertz (Hz). Abbreviations for NMR data are s (singlet), d (doublet), t (triplet), q (quartet), quin (quintet), sxt (sextet). Infrared (IR) spectra were recorded on a FTIR spectrometer and mass spectra were obtained using positive or negative electrospray ionisation (ESI), atmospheric pressure chemical ionization (APCI), electron impact ionization (EI) or chemical ionization (CI) techniques. Column chromatography was carried out using silica gel 60 Angstrom (Å), 240-400 mesh. Thin layer chromatography (TLC) was performed on aluminium sheets pre-coated with silica gel, 0.20 mm (Macherey-Nagel, Polygram® Sil G/UV254). TLC plates were visualized by UV absorption, phosphomolybdic acid, vanillin or potassium permanganate solution and heating. Melting points were measured on solids as obtained after chromatography.

Optimization of the Sulfoxide-Mediated Oxidative Cross-Coupling

Table S1. Optimization of the Sulfoxide-Mediated Oxidative Cross-Coupling[a]

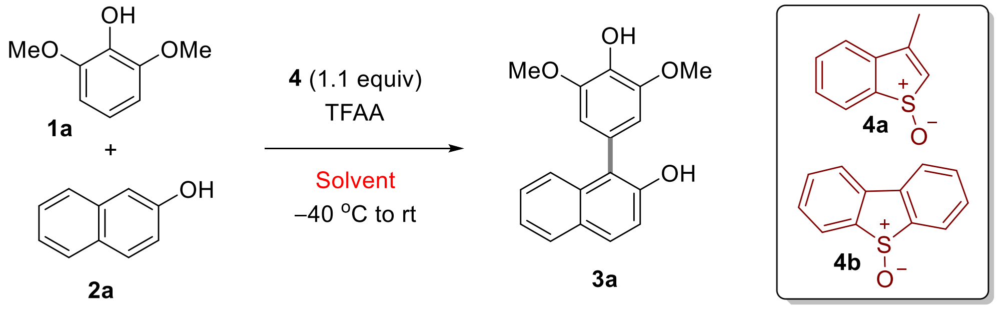

Entry	Sulfoxide (4)	Solvent	Yield (%)
1	**4a**	CH_2Cl_2	72 (68)
2	**4a**	THF	trace
3	**4a**	MeCN	<10
4	**4b**	CH_2Cl_2	67
5	**4a**	CH_2Cl_2	(91)[b]
6	**4a**	CH_2Cl_2	trace[c]

[a] Reaction conditions: Sulfoxide **4** (0.11 mmol) was dissolved in CH_2Cl_2 (1 mL) in an oven dried tube flushed with N_2. TFAA (0.17 mmol, 1.7 equiv) was then added at −40 °C. After 5 min at the same temperature, nucleophile **1a** (0.10 mmol in 0.5 mL CH_2Cl_2) was added in one portion. Nucleophile **2a** (0.1 mmol in 0.5 mL CH_2Cl_2) was then added immediately. After 15 min at −40 °C, the mixture was warmed to room temperature and stirred for 2 h. Yield determined by ^{1}H NMR analysis of the crude reaction mixture. Isolated yield in parentheses. [b] Used 0.15 mmol **2a**. [c] 2-naphthol **2a** added first.

Procedure: Sulfoxide **4** (0.11 mmol) was dissolved in CH_2Cl_2 (1 mL) in an oven dried tube flushed with N_2. TFAA (0.17 mmol, 1.7 equiv) was then added at −40 °C. After 5 min at the same temperature, nucleophile **1a** (0.10 mmol in 0.5 mL CH_2Cl_2) was added in one portion. Nucleophile **2a** (0.10 or 0.15 mmol in 0.5 mL CH_2Cl_2) was then added immediately. After 15 min at −40 °C, the mixture was warmed to room temperature and stirred for 2 h. Saturated aqueous $NaHCO_3$ (0.1 mL) was then added and the aqueous phase was extracted

with CH_2Cl_2 (3 × 3 mL). The combined organic layers were dried over $MgSO_4$ and concentrated *in vacuo*. The crude product was purified by column chromatography on silica gel eluting with *n*-hexane in EtOAc.

General Procedure A. Oxidative Cross-Coupling of Phenols with Phenols, Phenol Derivatives and Arenes

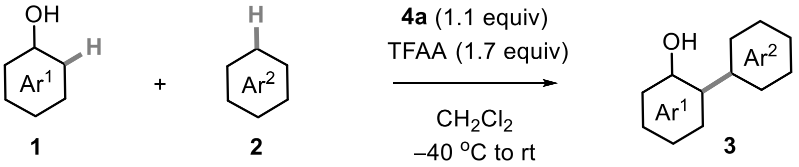

3-Methylbenzo[*b*]thiophene 1-oxide **4a** (0.11 mmol) was dissolved in CH_2Cl_2 (1 mL, indicated if different) in an oven dried tube flushed with N_2. TFAA (0.17 mmol, 1.7 equiv) was then added at −40 °C. After 5 min at the same temperature, nucleophile **1** (0.10 mmol in 0.5 mL CH_2Cl_2, indicated if different) was added in one portion. nucleophile **2** (0.15 mmol in 0.5 mL CH_2Cl_2, indicated if different) was then added immediately. After 15 min at −40 °C, the mixture was warmed to room temperature and stirred for 2 h. Saturated aqueous $NaHCO_3$ (0.1 mL) was then added and the aqueous phase was extracted with CH_2Cl_2 (3 × 3 mL). The combined organic layers were dried over $MgSO_4$ and concentrated *in vacuo*. The crude product was purified by column chromatography on silica gel eluting with *n*-hexane in EtOAc (indicated if different eluent was used).

1-(4-Hydroxy-3,5-dimethoxyphenyl)naphthalen-2-ol (3a)[1]

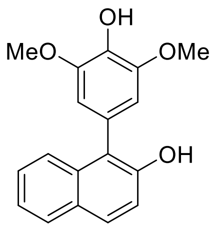

As described in general procedure **A**, 3-methylbenzo[*b*]thiophene 1-oxide **4a** (18 mg, 0.110 mmol), 2,6-dimethoxyphenol (15 mg, 0.100 mmol), and naphthalen-2-ol (22 mg, 0.150 mmol), TFAA (23 *u*L, 0.165 mmol), and CH_2Cl_2 (2 mL), gave **3a** (27 mg, 0.091 mmol, 91%) as a white solid; ^{1}H NMR (400 MHz, $CDCl_3$) δ = 3.90 (s, 6H, OCH_3),

[1] More, N. Y.; Jeganmohan, M. *Org. Lett.* **2015**, *17*, 3042.

5.30 (s, 1H, O*H*), 5.70 (s, 1H, O*H*), 6.64 (s, 2H, ArC*H*), 7.27 (d, *J* = 7.2 Hz, 1H, ArC*H*), 7.32-7.40 (m, 2H, ArC*H*), 7.48 (d, *J* = 8.0 Hz, 1H, ArC*H*), 7.80-7.83 (m, 2H, ArC*H*) ppm; ^{13}C NMR (100 MHz, $CDCl_3$) δ = 56.6 (O*C*H_3), 107.5 (Ar*C*H), 117.3 (Ar*C*H), 121.1 (Ar*C*), 123.4 (Ar*C*H), 124.7 (Ar*C*H), 124.8 (Ar*C*), 126.7 (Ar*C*H), 128.2 (Ar*C*H), 129.0 (Ar*C*), 129.6 (Ar*C*H), 133.6 (Ar*C*), 134.9 (Ar*C*), 148.1 (Ar*C*), 150.4 (Ar*C*) ppm. **HRMS** (ESI): Calcd. for $C_{18}H_{16}O_4Na$ (M+Na$^+$), 319.0941; found 319.0935.

1-(4-Hydroxy-3,5-dimethoxyphenyl)-7-methoxynaphthalen-2-ol (3b)

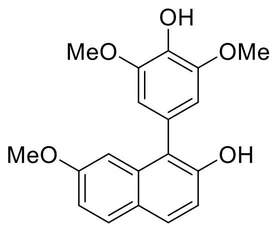

As described in general procedure **A**, 3-methylbenzo[*b*]thiophene 1-oxide **4a** (18 mg, 0.110 mmol), 2,6-dimethoxyphenol (15 mg, 0.100 mmol), and 7-methoxynaphthalen-2-ol (26 mg, 0.150 mmol), TFAA (23 *u*L, 0.165 mmol), and CH_2Cl_2 (2 mL), gave **3b** (23 mg, 0.070 mmol, 70%) as a white solid; m.p: 177-179 ºC; ^{1}H NMR (400 MHz, $CDCl_3$) δ = 3.72 (s, 3H, OC*H*$_3$), 3.90 (s, 6H, OC*H*$_3$), 5.25 (s, 1H, O*H*), 5.70 (s, 1H, O*H*), 6.64 (s, 2H, ArC*H*), 6.77 (d, *J* = 2.8 Hz, 1H, ArC*H*), 7.00 (dd, *J* = 9.0, 2.6 Hz, 1H, ArC*H*), 7.11 (d, *J* = 8.8 Hz, 1H, ArC*H*), 7.70-7.73 (m, 2H, ArC*H*) ppm; ^{13}C NMR (100 MHz, $CDCl_3$) δ = 55.4 (O*C*H_3), 56.6 (O*C*H_3), 103.7 (Ar*C*H), 107.4 (Ar*C*H), 114.8 (Ar*C*H), 115.6 (Ar*C*H), 120.4 (Ar*C*), 124.4 (Ar*C*), 125.0 (Ar*C*), 129.3 (Ar*C*H), 129.8 (Ar*C*H), 134.8 (Ar*C*), 134.8 (Ar*C*), 148.2 (Ar*C*), 151.1 (Ar*C*), 158.5 (Ar*C*) ppm. **ν_{max}** (neat)/cm^{-1} 735, 818, 834, 1033, 1114, 1217, 1265, 1345, 1464, 1514, 1622, 2838, 2941, 3056, 3502; **HRMS** (ESI): Calcd. for $C_{19}H_{18}O_5Na$ (M+Na$^+$), 349.1046; found 349.1040.

7-Bromo-1-(4-hydroxy-3,5-dimethoxyphenyl)naphthalen-2-ol (3c)

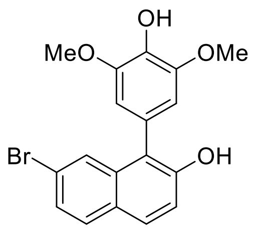

As described in general procedure **A**, 3-methylbenzo[*b*]thiophene 1-oxide **4a** (18 mg, 0.110 mmol), 2,6-dimethoxyphenol (15 mg, 0.100 mmol), and 7-bromonaphthalen-2-ol (33 mg, 0.150 mmol), TFAA (23 *u*L, 0.165 mmol), and CH_2Cl_2 (2 mL), gave **3c** (31 mg, 0.083 mmol, 83%) as a white solid; m.p: 238-240 ºC; ^{1}H NMR (400 MHz, $CDCl_3$) δ = 3.91 (s, 6H, OC*H₃*), 5.35 (s, 1H, O*H*), 5.73 (s, 1H, O*H*), 6.59 (s, 2H, ArC*H*), 7.26 (d, *J* = 8.8 Hz, 1H, ArC*H*), 7.40 (dd, *J* = 8.8, 2.0 Hz, 1H, ArC*H*), 7.59 (d, *J* = 1.6 Hz, 1H, ArC*H*), 7.67 (d, *J* = 8.4 Hz, 1H, ArC*H*), 7.76 (d, *J* = 8.8 Hz, 1H, ArC*H*) ppm; ^{13}C NMR (100 MHz, $CDCl_3$) δ = 56.6 (O*C*H₃), 107.5 (Ar*C*H), 117.8 (Ar*C*H), 120.5 (Ar*C*), 121.3 (Ar*C*), 123.8 (Ar*C*), 126.8 (Ar*C*H), 126.9 (Ar*C*H), 127.3 (Ar*C*), 129.5 (Ar*C*H), 129.8 (Ar*C*H), 134.9 (Ar*C*), 135.1 (Ar*C*), 148.2 (Ar*C*), 151.3 (Ar*C*) ppm. ν_{max} (neat)/cm^{-1} 703, 736, 818, 832, 890, 1018, 1069, 1112, 1200, 1264, 1411, 1498, 1608, 2852, 2927, 3448; **HRMS** (ESI): Calcd. for $C_{18}H_{15}O_4BrNa$ (M+Na$^+$), 397.0046; found 397.0040.

1-(4-Hydroxy-3,5-dimethoxyphenyl)-6-phenylnaphthalen-2-ol (3d)

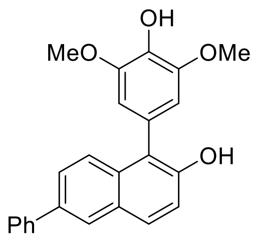

As described in general procedure **A**, 3-methylbenzo[*b*]thiophene 1-oxide **4a** (18 mg, 0.110 mmol), 2,6-dimethoxyphenol (15 mg, 0.100 mmol), and 6-phenylnaphthalen-2-ol (33 mg, 0.150 mmol), TFAA (23 *u*L, 0.165 mmol), and CH_2Cl_2 (2 mL), gave **3d** (24 mg, 0.064 mmol, 64%) as a white solid; m.p: 216-218 ºC; ^{1}H NMR (400 MHz, $CDCl_3$) δ = 3.91 (s, 6H, OC*H₃*), 5.33 (s, 1H, O*H*), 5.71 (s, 1H, O*H*), 6.66 (s, 2H, ArC*H*), 7.30 (d, *J* = 8.8 Hz, 1H, ArC*H*), 7.36 (t, *J* = 7.4 Hz, 1H, ArC*H*), 7.47 (t, *J* = 7.6 Hz, 2H, ArC*H*), 7.55 (d, *J* = 8.8 Hz, 1H, ArC*H*), 7.65 (dd, *J* = 8.6, 1.8 Hz, 1H, ArC*H*), 7.70 (d, *J* = 7.2 Hz, 2H, ArC*H*), 7.87 (d, *J* = 8.8 Hz, 1H, ArC*H*), 8.02 (d, *J* = 1.2 Hz, 1H, ArC*H*) ppm; ^{13}C NMR (100 MHz, $CDCl_3$) δ = 56.6 (O*C*H₃), 107.6 (Ar*C*H), 117.8 (Ar*C*H), 121.1 (Ar*C*), 124.6 (Ar*C*), 125.4 (Ar*C*H), 126.1 (Ar*C*H), 126.3 (Ar*C*H),

127.29 (Ar*C*H), 127.34 (Ar*C*H), 129.0 (Ar*C*H), 129.2 (Ar*C*), 129.9 (Ar*C*H), 132.8 (Ar*C*), 135.0 (Ar*C*), 136.2 (Ar*C*), 141.1 (Ar*C*), 148.2 (Ar*C*), 150.6 (Ar*C*) ppm. **ν_{max}** (neat)/cm^{-1} 736, 1114, 1216, 1242, 1264, 1358, 1417, 1455, 1494, 1519, 1596, 2941, 3517; **HRMS** (ESI): Calcd. for $C_{24}H_{20}O_4Na$ (M+Na$^+$), 395.1254; found 395.1237.

6-Bromo-1-(4-hydroxy-3,5-dimethoxyphenyl)naphthalen-2-ol (3e)[2]

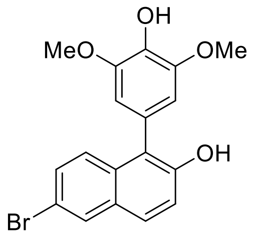

As described in general procedure **A**, 3-methylbenzo[*b*]thiophene 1-oxide **4a** (18 mg, 0.110 mmol), 2,6-dimethoxyphenol (15 mg, 0.100 mmol), and 6-bromonaphthalen-2-ol (33 mg, 0.150 mmol), TFAA (23 *u*L, 0.165 mmol), and CH_2Cl_2 (2 mL), gave **3e** (23 mg, 0.061 mmol, 61%) as a white solid; ^{1}H NMR (400 MHz, $CDCl_3$) δ = 3.89 (s, 6H, OC*H₃*), 5.32 (s, 1H, O*H*), 5.70 (s, 1H, O*H*), 6.59 (s, 2H, ArC*H*), 7.27 (d, J = 9.2 Hz, 1H, ArC*H*), 7.34 (d, J = 9.2 Hz, 1H, ArC*H*), 7.42 (dd, J = 9.2, 2.0 Hz, 1H, ArC*H*), 7.70 (d, J = 8.8 Hz, 1H, ArC*H*), 7.96 (d, J = 2.0 Hz, 1H, ArC*H*) ppm; ^{13}C NMR (100 MHz, $CDCl_3$) δ = 56.6 (O*C*H₃), 107.4 (Ar*C*H), 117.2 (Ar*C*), 118.5 (Ar*C*H), 121.4 (Ar*C*), 124.1 (Ar*C*), 126.7 (Ar*C*H), 128.6 (Ar*C*H), 129.9 (Ar*C*H), 130.0 (Ar*C*H), 130.1 (Ar*C*), 132.2 (Ar*C*), 135.1 (Ar*C*), 148.2 (Ar*C*), 150.8 (Ar*C*) ppm. **HRMS** (ESI): Calcd. for $C_{18}H_{15}O_4BrNa$ (M+Na$^+$), 397.0046; found 397.0039.

6-Hydroxy-5-(4-hydroxy-3,5-dimethoxyphenyl)-2-naphthonitrile (3f)

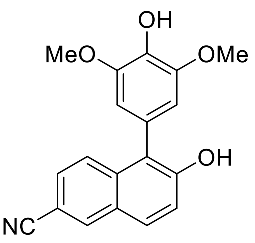

As described in general procedure **A**, 3-methylbenzo[*b*]thiophene 1-oxide **4a** (18 mg, 0.110 mmol), 2,6-dimethoxyphenol (15 mg, 0.100 mmol), and 6-hydroxy-2-naphthonitrile (25 mg, 0.150 mmol), TFAA (23 *u*L, 0.165 mmol), and CH_2Cl_2 (2 mL), gave **3f** (23 mg, 0.072 mmol, 72%) as a white solid; m.p: 230-232 ºC; ^{1}H

[2] Morimoto, K.; Sakamoto, K.; Ohshika, T.; Dohi, T.; Kita, Y. *Angew. Chem. Int. Ed.* **2016**, *55*, 3652.

NMR (400 MHz, Acetone-d_6) δ = 3.84 (s, 6H, OC*H₃*), 6.61 (s, 2H, ArC*H*), 7.40-7.43 (m, 2H, ArC*H*+O*H*), 7.54 (dd, *J* = 8.8, 1.6 Hz, 1H, ArC*H*), 7.61 (d, *J* = 8.8 Hz, 1H, ArC*H*), 7.98 (d, *J* = 8.8 Hz, 1H, ArC*H*), 8.29 (s, 1H, O*H*), 8.36 (s, 1H, ArC*H*) ppm; ^{13}C NMR (100 MHz, Acetone-d_6) δ = 57.8 (O*C*H$_3$), 107.8 (Ar*C*), 110.3 (Ar*C*H), 121.2 (*C*N), 122.1 (Ar*C*H), 124.4 (Ar*C*), 126.3 (Ar*C*), 128.2 (Ar*C*H), 128.5 (Ar*C*H), 129.5 (Ar*C*), 131.6 (Ar*C*H), 135.9 (Ar*C*H), 137.8 (Ar*C*), 137.9 (Ar*C*), 150.3 (Ar*C*), 156.6 (Ar*C*) ppm. **ν_{max}** (neat)/cm^{-1} 703, 733, 829, 897, 1112, 1213, 1264, 1356, 1421, 1519, 1615, 2225, 2936, 3421; **HRMS** (ESI): Calcd. for $C_{19}H_{15}O_4NNa$ (M+Na$^+$), 344.0893; found 344.0889.

Methyl 6-hydroxy-5-(4-hydroxy-3,5-dimethoxyphenyl)-2-naphthoate (3g)[3]

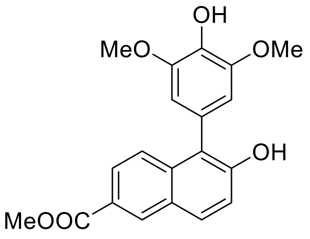

As described in general procedure **A**, 3-methylbenzo[*b*]thiophene 1-oxide **4a** (18 mg, 0.110 mmol), 2,6-dimethoxyphenol (15 mg, 0.100 mmol), and methyl 6-hydroxy-2-naphthoate (30 mg, 0.150 mmol), TFAA (23 *u*L, 0.165 mmol), and CH_2Cl_2 (2 mL), gave **3g** (24 mg, 0.092 mmol, 92%) as a white solid; ^{1}H NMR (400 MHz, $CDCl_3$) δ = 3.90 (s, 6H, OC*H₃*), 3.96 (s, 3H, COOC*H₃*), 5.51 (s, 1H, O*H*), 5.73 (s, 1H, O*H*), 6.61 (s, 2H, ArC*H*), 7.32 (d, *J* = 8.8 Hz, 1H, ArC*H*), 7.50 (d, *J* = 8.8 Hz, 1H, ArC*H*), 7.90-7.94 (m, 2H, ArC*H*), 8.57 (d, *J* = 1.6 Hz, 1H, ArC*H*) ppm; ^{13}C NMR (100 MHz, $CDCl_3$) δ = 52.3 (COO*C*H$_3$), 56.6 (O*C*H$_3$), 107.5 (Ar*C*H), 118.2 (Ar*C*H), 121.4 (Ar*C*), 124.0 (Ar*C*), 124.97 (Ar*C*), 125.02 (Ar*C*H), 126.1 (Ar*C*H), 128.0 (Ar*C*), 131.1 (Ar*C*H), 131.3 (Ar*C*H), 135.1 (Ar*C*), 136.0 (Ar*C*), 148.2 (Ar*C*), 152.6 (Ar*C*), 167.5 (*C*OOCH$_3$) ppm. **HRMS** (ESI): Calcd. for $C_{20}H_{18}O_6Na$ (M+Na$^+$), 377.0996; found 377.0990.

[3] Libman, A.; Shalit, H.; Vainer, Y.; Narute, S.; Kozuch, S.; Pappo, D. *J. Am. Chem. Soc.* **2015**, *137,* 11453.

3-Bromo-1-(4-hydroxy-3,5-dimethoxyphenyl)naphthalen-2-ol (3h)[3]

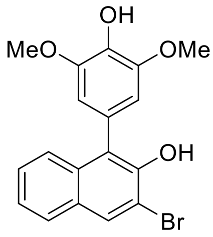

As described in general procedure **A**, 3-methylbenzo[*b*]thiophene 1-oxide **4a** (18 mg, 0.110 mmol), 2,6-dimethoxyphenol (15 mg, 0.100 mmol), and 3-bromonaphthalen-2-ol (33 mg, 0.15 mmol), TFAA (23 *u*L, 0.165 mmol), and CH_2Cl_2 (2 mL), gave **3h** (33 mg, 0.088 mmol, 88%) as a white solid; ^{1}H NMR (400 MHz, $CDCl_3$) δ = 3.89 (s, 6H, O*CH₃*), 5.66 (s, 1H, O*H*), 5.70 (s, 1H, O*H*), 6.61 (s, 2H, ArC*H*), 7.34-7.46 (m, 3H, ArC*H*), 7.72-7.75 (m, 1H, ArC*H*), 8.10 (s, 1H, ArC*H*) ppm; ^{13}C NMR (100 MHz, $CDCl_3$) δ = 56.5 (O*C*H₃), 107.3 (Ar*C*H), 111.7 (Ar*C*), 122.9 (Ar*C*), 124.4 (Ar*C*H), 124.9 (Ar*C*), 125.2 (Ar*C*H), 127.0 (Ar*C*H), 127.2 (Ar*C*H), 129.5 (Ar*C*), 131.6 (Ar*C*H), 133.0 (Ar*C*), 135.0 (Ar*C*), 146.9 (Ar*C*), 147.9 (Ar*C*) ppm. **HRMS** (ESI): Calcd. for $C_{18}H_{15}O_4BrNa$ (M+Na$^+$), 397.0046; found 397.0040.

Methyl 3-hydroxy-4-(4-hydroxy-3,5-dimethoxyphenyl)-2-naphthoate (3i)[3]

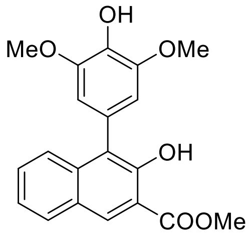

As described in general procedure **A**, 3-methylbenzo[*b*]thiophene 1-oxide **4a** (18 mg, 0.110 mmol), 2,6-dimethoxyphenol (15 mg, 0.100 mmol), and methyl 3-hydroxy-2-naphthoate (30 mg, 0.150 mmol), TFAA (23 *u*L, 0.165 mmol), and CH_2Cl_2 (2 mL), gave **3i** (27 mg, 0.078 mmol, 78%) as a white solid; ^{1}H NMR (400 MHz, $CDCl_3$) δ = 3.89 (s, 6H, O*CH₃*), 4.06 (s, 3H, COO*CH₃*), 5.62 (s, 1H, O*H*), 6.62 (s, 2H, ArC*H*), 7.35 (t, J = 8.0 Hz, 1H, ArC*H*), 7.42-7.46 (m, 1H, ArC*H*), 7.54 (d, J = 8.4 Hz, 1H, ArC*H*), 7.86 (d, J = 8.0 Hz, 1H, ArC*H*), 8.56 (s, 1H, ArC*H*), 10.73 (s, 1H, O*H*) ppm; ^{13}C NMR (100 MHz, $CDCl_3$) δ = 52.9 (COO*C*H₃), 56.4 (O*C*H₃), 107.5 (Ar*C*H), 113.9 (Ar*C*), 123.9 (Ar*C*H), 124.1 (Ar*C*), 125.2 (Ar*C*H), 126.4 (Ar*C*), 127.0 (Ar*C*), 129.3 (Ar*C*H), 129.6 (Ar*C*H), 132.1 (Ar*C*H), 134.2 (Ar*C*), 137.3 (Ar*C*), 147.2 (Ar*C*), 153.2 (Ar*C*), 170.7 (*C*OOCH₃) ppm. **HRMS** (ESI): Calcd. for $C_{20}H_{18}O_6Na$ (M+Na$^+$), 377.0996; found 377.0989.

4-(4-Hydroxy-3,5-dimethoxyphenyl)naphthalen-1-ol (3j)

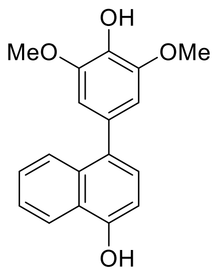

As described in general procedure **A**, 3-methylbenzo[*b*]thiophene 1-oxide **4a** (18 mg, 0.110 mmol), 2,6-dimethoxyphenol (15 mg, 0.10 mmol), and naphthalen-1-ol (22 mg, 0.150 mmol), TFAA (23 *u*L, 0.165 mmol), and CH_2Cl_2 (2 mL), gave **3j** (15 mg, 0.051 mmol, 51%) as a white solid; m.p: 171-173 ºC; ^{1}H NMR (400 MHz, $CDCl_3$) δ = 3.94 (s, 6H, OC*H*$_3$), 5.64 (s, 1H, O*H*), 5.96 (s, 1H, O*H*), 6.74 (s, 2H, ArC*H*), 7.35 (t, J = 8.4 Hz, 1H, ArC*H*), 7.46-7.53 (m, 3H, ArC*H*), 7.81-7.84 (m, 1H, ArC*H*), 8.27-8.29 (m, 1H, ArC*H*) ppm; ^{13}C NMR (100 MHz, $CDCl_3$) δ = 56.6 (O*C*H$_3$), 106.1 (Ar*C*H), 120.2 (Ar*C*H), 121.5 (Ar*C*), 122.5 (Ar*C*H), 124.3 (Ar*C*), 125.7 (Ar*C*H), 126.5 (Ar*C*H), 127.6 (Ar*C*H), 128.4 (Ar*C*), 134.2 (Ar*C*), 134.7 (Ar*C*), 147.9 (Ar*C*), 148.0 (Ar*C*) ppm. **ν$_{max}$** (neat)/cm^{-1} 736, 809, 845, 936, 1020, 1070, 1114, 1211, 1245, 1266, 1383, 1416, 1455, 1519, 1611, 2841, 2939, 3053, 3515; **HRMS** (ESI): Calcd. for $C_{18}H_{16}O_4Na$ (M+Na$^+$), 319.0941; found 319.0931.

3',5'-Dimethoxy-5-methyl-[1,1'-biphenyl]-2,4'-diol (3k)[2]

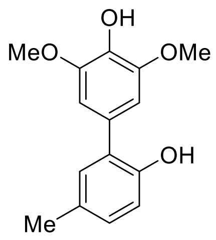

As described in general procedure **A**, 3-methylbenzo[*b*]thiophene 1-oxide **4a** (18 mg, 0.110 mmol), 2,6-dimethoxyphenol (15 mg, 0.100 mmol), and *p*-cresol (16 mg, 0.15 mmol), TFAA (23 *u*L, 0.165 mmol), and CH_2Cl_2 (2 mL), gave **3k** (5 mg, 0.019 mmol, 19%) as a white solid; ^{1}H NMR (400 MHz, $CDCl_3$) δ = 2.32 (s, 3H, C*H*$_3$), 3.92 (s, 6H, OC*H*$_3$), 5.15 (s, 1H, O*H*), 5.58 (s, 1H, O*H*), 6.65 (s, 2H, ArC*H*), 6.88 (d, J = 8.0 Hz, 1H, ArC*H*), 7.04-7.06 (m, 2H, ArC*H*), ppm; ^{13}C NMR (125 MHz, $CDCl_3$) δ = 20.6 (*C*H$_3$), 56.6 (O*C*H$_3$), 105.8 (Ar*C*H), 115.6 (Ar*C*H), 128.0 (Ar*C*), 128.2 (Ar*C*), 129.6 (Ar*C*H), 130.0 (Ar*C*), 130.6 (Ar*C*H), 134.6 (Ar*C*), 147.8 (Ar*C*), 150.3 (Ar*C*) ppm.

3',5,5'-Trimethoxy-[1,1'-biphenyl]-2,4'-diol (3l)

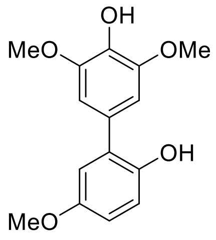

As described in general procedure **A**, 3-methylbenzo[*b*]thiophene 1-oxide **4a** (18 mg, 0.110 mmol), 2,6-dimethoxyphenol (15 mg, 0.100 mmol), and 4-methoxyphenol (19 mg, 0.150 mmol), TFAA (23 *u*L, 0.165 mmol), and CH_2Cl_2 (2 mL), gave **3l** (11 mg, 0.040 mmol, 40%) as a white solid; m.p: 148-150 °C; ^{1}H NMR (400 MHz, $CDCl_3$) δ = 3.80 (s, 6H, OC*H*$_3$), 3.91 (s, 3H, OC*H*$_3$), 5.02 (s, 1H, O*H*), 5.62 (s, 1H, O*H*), 6.66 (s, 2H, ArC*H*), 6.79-6.83 (m, 2H, ArC*H*), 6.91 (d, *J* = 8.4 Hz, 1H, ArC*H*) ppm; ^{13}C NMR (100 MHz, $CDCl_3$) δ = 56.0 (O*C*H$_3$), 56.4 (O*C*H$_3$), 105.7 (Ar*C*H), 114.3 (Ar*C*H), 115.4 (Ar*C*H), 116.4 (Ar*C*H), 128.1 (Ar*C*), 128.9 (Ar*C*), 134.7 (Ar*C*), 146.6 (Ar*C*), 147.7 (Ar*C*), 153.6 (Ar*C*) ppm. **v$_{max}$** (neat)/cm^{-1} 736, 839, 1038, 1113, 1214, 1344, 1407, 1464, 1494, 1610, 2836, 2938, 3057, 3440; **HRMS** (ESI): Calcd. for $C_{15}H_{17}O_5$ (M+H$^+$), 277.1071; found 277.1068.

3,5-Dimethoxy-3',5'-dimethyl-[1,1'-biphenyl]-4,4'-diol (3m)[4]

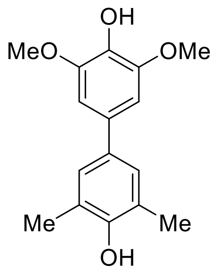

As described in general procedure **A**, 3-methylbenzo[*b*]thiophene 1-oxide **4a** (18 mg, 0.110 mmol), 2,6-dimethoxyphenol (15 mg, 0.100 mmol), and 2,6-dimethylphenol (18 mg, 0.150 mmol), TFAA (23 *u*L, 0.165 mmol), and CH_2Cl_2 (2 mL), gave **3m** (10 mg, 0.037 mmol, 37%) as a yellow solid; ^{1}H NMR (400 MHz, $CDCl_3$) δ = 2.31 (s, 6H, C*H*$_3$), 3.95 (s, 6H, OC*H*$_3$), 4.65 (s, 1H, O*H*), 5.48 (s, 1H, O*H*), 6.72 (s, 2H, ArC*H*), 7.15 (s, 2H, ArC*H*) ppm; ^{13}C NMR (100 MHz, $CDCl_3$) δ = 16.2 (*C*H$_3$), 56.5 (O*C*H$_3$), 103.9 (Ar*C*H), 123.4 (Ar*C*), 127.3 (Ar*C*H), 133.1 (Ar*C*), 133.8 (Ar*C*), 134.0 (Ar*C*), 147.3 (Ar*C*), 151.7 (Ar*C*) ppm.

[4] Quell, T.; Beiser, N.; Dyballa, K. M.; Franke, R.; Waldvogel, S. R. *Eur. J. Org. Chem.* **2016**, 4307.

2,6-Dimethoxy-4-(2-methoxynaphthalen-1-yl)phenol (3n)[2]

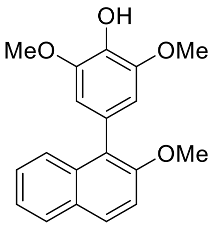

As described in general procedure **A**, 3-methylbenzo[*b*]thiophene 1-oxide **4a** (18 mg, 0.110 mmol), 2,6-dimethoxyphenol (15 mg, 0.100 mmol), and 2-methoxynaphthalene (24 mg, 0.150 mmol), TFAA (23 *u*L, 0.165 mmol), and CH_2Cl_2 (2 mL), gave **3n** (22 mg, 0.071 mmol, 71%) as a white solid; ^{1}H NMR (400 MHz, $CDCl_3$) δ = 3.87 (s, 3H, OCH_3), 3.88 (s, 6H, OCH_3), 5.60 (s, 1H, O*H*), 6.59 (s, 2H, ArC*H*), 7.34-7.38 (m, 3H, ArC*H*), 7.54-7.56 (m, 1H, ArC*H*), 7.81-7.83 (m, 1H, ArC*H*), 7.88 (d, *J* = 8.8 Hz, 1H, ArC*H*) ppm; ^{13}C NMR (100 MHz, $CDCl_3$) δ = 56.4 (O*C*H_3), 57.0 (O*C*H_3), 107.7 (Ar*C*H), 113.9 (Ar*C*H), 123.7 (Ar*C*H), 125.5 (Ar*C*H), 126.5 (Ar*C*H), 127.4 (Ar*C*), 127.9 (Ar*C*H), 129.10 (Ar*C*H), 129.13 (Ar*C*), 133.9 (Ar*C*), 134.0 (Ar*C*), 147.0 (Ar*C*), 153.9 (Ar*C*) ppm. **HRMS** (ESI): Calcd. for $C_{19}H_{17}O_4$ (M-H$^+$), 309.1132; found 309.1135.

2,6-Dimethoxy-4-(2-methoxynaphthalen-1-yl)phenol (3o)[2]

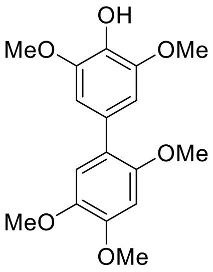

As described in general procedure **A**, 3-methylbenzo[*b*]thiophene 1-oxide **4a** (18 mg, 0.110 mmol), 2,6-dimethoxyphenol (15 mg, 0.100 mmol), and 1,2,4-trimethoxybenzene (25 mg, 0.150 mmol), TFAA (23 *u*L, 0.165 mmol), and CH_2Cl_2 (2 mL), gave **3o** (21 mg, 0.069 mmol, 69%) as a white solid; ^{1}H NMR (400 MHz, $CDCl_3$) δ = 3.77 (s, 3H, OCH_3), 3.88 (s, 3H, OCH_3), 3.91 (s, 6H, OCH_3), 3.94 (s, 3H, OCH_3), 6.62 (s, 1H, ArC*H*), 6.73 (s, 2H, ArC*H*), 6.86 (s, 1H, ArC*H*) ppm; ^{13}C NMR (100 MHz, $CDCl_3$) δ = 56.4 (O*C*H_3), 56.5 (O*C*H_3), 56.9 (O*C*H_3), 57.0 (O*C*H_3), 98.8 (Ar*C*H), 106.5 (Ar*C*H), 114.8 (Ar*C*H), 122.8 (Ar*C*), 129.6 (Ar*C*), 134.0 (Ar*C*), 143.4 (Ar*C*), 146.8 (Ar*C*), 149.0 (Ar*C*), 150.8 (Ar*C*) ppm.

2',3,5,5'-Tetramethoxy-[1,1'-biphenyl]-4-ol (3p)[5]

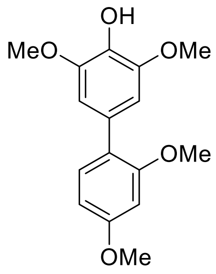

As described in general procedure **A**, 3-methylbenzo[*b*]thiophene 1-oxide **4a** (18 mg, 0.110 mmol), 2,6-dimethoxyphenol (15 mg, 0.100 mmol), and 1,3-dimethoxybenzene (21 mg, 0.150 mmol), TFAA (23 *u*L, 0.165 mmol), and CH_2Cl_2 (2 mL), gave **3p** (16 mg, 0.055 mmol, 55%) as colorless oil; ^{1}H NMR (400 MHz, $CDCl_3$) δ = 3.80 (s, 3H, OCH_3), 3.85 (s, 3H, OCH_3), 3.90 (s, 6H, OCH_3), 5.50 (s, 1H, O*H*), 6.55-6.59 (m, 2H, ArC*H*), 6.72 (s, 2H, ArC*H*), 7.22 (d, *J* = 8.8 Hz, 1H, ArC*H*) ppm; ^{13}C NMR (100 MHz, $CDCl_3$) δ = 55.6 (O*C*H$_3$), 55.8 (O*C*H$_3$), 56.5 (O*C*H$_3$), 99.2 (Ar*C*H), 104.7 (Ar*C*H), 106.6 (Ar*C*H), 123.8 (Ar*C*), 129.6 (Ar*C*), 131.2 (Ar*C*H), 133.9 (Ar*C*), 146.7 (Ar*C*), 157.5 (Ar*C*), 160.2 (Ar*C*) ppm.

2',3,4',5,6'-Pentamethoxy-[1,1'-biphenyl]-4-ol (3q)[6]

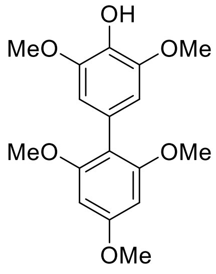

As described in general procedure **A**, 3-methylbenzo[*b*]thiophene 1-oxide **4a** (18 mg, 0.110 mmol), 2,6-dimethoxyphenol (15 mg, 0.100 mmol), and 1,3,5-trimethoxybenzene (25 mg, 0.150 mmol), TFAA (23 *u*L, 0.165 mmol), and CH_2Cl_2 (2 mL), gave **3q** (17 mg, 0.053 mmol, 53%) as a white solid; ^{1}H NMR (400 MHz, $CDCl_3$) δ = 3.74 (s, 6H, OCH_3), 3.866-3.871 (m, 9H, OCH_3), 5.50 (s, 1H, O*H*), 6.24 (s, 2H, ArC*H*), 6.56 (s, 2H, ArC*H*) ppm; ^{13}C NMR (100 MHz, $CDCl_3$) δ = 55.5 (O*C*H$_3$), 56.0 (O*C*H$_3$), 56.4 (O*C*H$_3$), 91.1 (Ar*C*H), 108.1 (Ar*C*H), 112.6 (Ar*C*), 124.9 (Ar*C*), 133.7 (Ar*C*), 146.6 (Ar*C*), 158.6 (Ar*C*), 160.5 (Ar*C*) ppm.

[5] Morimoto, K.; Sakamoto, K.; Ohnishi, Y.; Miyamoto, T.; Ito, M.; Dohi, T.; Kita, Y. *Chem. - Eur. J.* **2013**, *19*, 8726.

[6] More, N. Y.; Jeganmohan, M. *Eur. J. Org. Chem.* **2017**, *2017*, 4305.

2,6-Dimethoxy-4-(pyren-1-yl)phenol (3r)

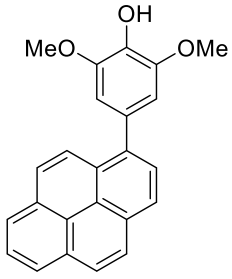

As described in general procedure **A**, 3-methylbenzo[*b*]thiophene 1-oxide **4a** (18 mg, 0.110 mmol), 2,6-dimethoxyphenol (15 mg, 0.100 mmol), and pyrene (30 mg, 0.150 mmol), TFAA (23 *u*L, 0.165 mmol), and CH_2Cl_2 (2 mL), gave **3r** (15 mg, 0.043 mmol, 43%) as a pink solid; m.p: 197-198 ºC; ^{1}H NMR (400 MHz, $CDCl_3$) δ = 3.95 (s, 6H, OC*H₃*), 5.66 (s, 1H, O*H*), 6.85 (s, 2H, ArC*H*), 7.98-8.24 (m, 9H, ArC*H*) ppm; ^{13}C NMR (100 MHz, $CDCl_3$) δ = 56.6 (O*C*H₃), 107.6 (Ar*C*H), 124.7 (Ar*C*H), 124.95 (Ar*C*), 125.04 (Ar*C*H), 125.1 (Ar*C*), 125.3 (Ar*C*H), 125.4 (Ar*C*H), 126.2 (Ar*C*H), 127.51 (Ar*C*H), 127.54 (Ar*C*H), 127.6 (Ar*C*H), 127.7 (Ar*C*H), 128.8 (Ar*C*), 130.6 (Ar*C*), 131.1 (Ar*C*), 131.6 (Ar*C*), 132.5 (Ar*C*), 134.3 (Ar*C*), 138.0 (Ar*C*), 147.1 (Ar*C*) ppm. **ν$_{max}$** (neat)/cm^{-1} 736, 842, 905, 1112, 1209, 1313, 1341, 1452, 1501, 1611, 2839, 2937, 3038, 3515; **HRMS** (ESI): Calcd. for $C_{24}H_{19}O_3$ (M+H$^+$), 355.1329; found 355.1322.

1-(2-Hydroxy-3-methoxy-5-methylphenyl)naphthalen-2-ol (3s)[3]

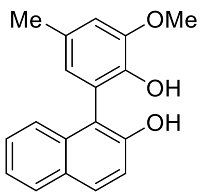

As described in general procedure **A**, 3-methylbenzo[*b*]thiophene 1-oxide **4a** (18 mg, 0.110 mmol), 2-methoxy-4-methylphenol (14 mg, 0.100 mmol), and naphthalen-2-ol (22 mg, 0.150 mmol), TFAA (23 *u*L, 0.165 mmol), and CH_2Cl_2 (2 mL), gave **3s** (21 mg, 0.075 mmol, 75%) as a white solid; ^{1}H NMR (400 MHz, $CDCl_3$) δ = 2.33 (s, 3H, C*H₃*), 3.94 (s, 3H, OC*H₃*), 5.36 (s, 1H, O*H*), 5.56 (s, 1H, O*H*), 6.68 (s, 1H, ArC*H*), 6.80 (s, 1H, ArC*H*), 7.22-7.34 (m, 3H, ArC*H*), 7.42 (d, *J* = 8.4 Hz, 1H, ArC*H*), 7.77-7.79 (m, 2H, ArC*H*) ppm; ^{13}C NMR (100 MHz, $CDCl_3$) δ = 21.3 (*C*H₃), 56.2 (O*C*H₃), 112.2 (Ar*C*H), 116.6 (Ar*C*), 117.9 (Ar*C*H), 119.4 (Ar*C*), 123.5 (Ar*C*H), 124.5 (Ar*C*H), 124.9 (Ar*C*H), 126.6 (Ar*C*H), 128.2 (Ar*C*H), 129.3 (Ar*C*), 130.0 (Ar*C*H), 130.5 (Ar*C*), 133.2 (Ar*C*), 141.8 (Ar*C*), 147.3 (Ar*C*), 150.9 (Ar*C*) ppm. **HRMS** (ESI): Calcd. for $C_{18}H_{16}O_3K$ (M+K$^+$), 319.0731; found 319.0731.

2-Methoxy-6-(2-methoxynaphthalen-1-yl)-4-methylphenol (3t)[7]

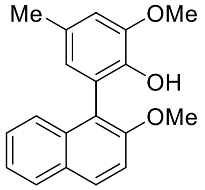

As described in general procedure **A**, 3-methylbenzo[*b*]thiophene 1-oxide **4a** (18 mg, 0.110 mmol), 2-methoxy-4-methylphenol (14 mg, 0.100 mmol), and 2-methoxynaphthalene (24 mg, 0.150 mmol), TFAA (23 *u*L, 0.165 mmol), and CH_2Cl_2 (2 mL), gave **3t** (22 mg, 0.071 mmol, 71%) as a white solid; ^{1}H NMR (500 MHz, $CDCl_3$) δ = 2.36 (s, 3H, CH_3), 3.89 (s, 3H, OCH_3), 3.95 (s, 3H, OCH_3), 5.36 (s, 1H, O*H*), 6.66 (s, 1H, ArC*H*), 6.79 (d, *J* = 1.5 Hz, 1H, ArC*H*), 7.32-7.40 (m, 3H, ArC*H*), 7.47-7.49 (m, 1H, ArC*H*), 7.81-7.83 (m, 1H, ArC*H*), 7.90 (d, *J* = 9.0 Hz, 1H, ArC*H*) ppm; ^{13}C NMR (125 MHz, $CDCl_3$) δ = 21.4 (*C*H_3), 56.06 (O*C*H_3), 56.13 (O*C*H_3), 111.3 (Ar*C*H), 114.1 (Ar*C*H), 120.7 (Ar*C*), 122.3 (Ar*C*), 123.7 (Ar*C*H), 124.5 (Ar*C*H), 125.4 (Ar*C*H), 126.5 (Ar*C*H), 128.0 (Ar*C*H), 129.0 (Ar*C*), 129.3 (Ar*C*), 129.7 (Ar*C*H), 133.7 (Ar*C*), 141.5 (Ar*C*), 146.9 (Ar*C*), 154.4 (Ar*C*) ppm.

2',3,4'-Trimethoxy-5-methyl-[1,1'-biphenyl]-2-ol (3u)[8]

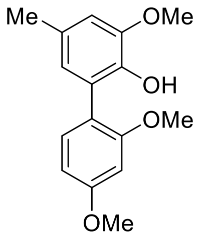

As described in general procedure **A**, 3-methylbenzo[*b*]thiophene 1-oxide **4a** (18 mg, 0.110 mmol), 2-methoxy-4-methylphenol (14 mg, 0.100 mmol), and 1,3-dimethoxybenzene (21 mg, 0.150 mmol), TFAA (23 *u*L, 0.165 mmol), and CH_2Cl_2 (2 mL), gave **3u** (16 mg, 0.055 mmol, 55%) as colorless oil; ^{1}H NMR (400 MHz, $CDCl_3$) δ = 2.32 (s, 3H, CH_3), 3.82 (s, 3H, OCH_3), 3.85 (s, 3H, OCH_3), 3.90 (s, 3H, OCH_3), 5.77 (s, 1H, O*H*), 6.58-6.61 (m, 2H, ArC*H*), 6.66 (s, 1H, ArC*H*), 6.70 (s, 1H, ArC*H*), 7.22 (d, *J* = 8.0 Hz, 1H, ArC*H*) ppm; ^{13}C NMR (100 MHz, $CDCl_3$) δ = 21.3 (*C*H_3), 55.6 (O*C*H_3), 56.0 (O*C*H_3), 56.1 (O*C*H_3), 99.0 (Ar*C*H), 105.1 (Ar*C*H), 111.2 (Ar*C*H), 119.6 (Ar*C*), 123.8 (Ar*C*H), 125.2 (Ar*C*), 129.2 (Ar*C*), 132.3 (Ar*C*H), 141.0 (Ar*C*), 147.3 (Ar*C*), 157.3 (Ar*C*), 160.7 (Ar*C*) ppm.

---

[7] Bering, L.; Vogt, M.; Paulussen, F. M.; Antonchick, A. P. *Org. Lett.* **2018**, *20*, 4077.

[8] Lips, S.; Wiebe, A.; Elsler, B.; Schollmeyer, D.; Dyballa, K. M.; Franke, R.; Waldvogel, S. R. *Angew. Chem., Int. Ed.* **2016**, *55*, 10872.

2',3,4',6'-Tetramethoxy-5-methyl-[1,1'-biphenyl]-2-ol (3v)[9]

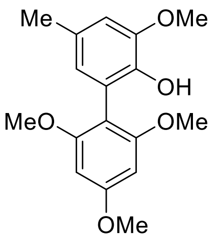

As described in general procedure **A**, 3-methylbenzo[*b*]thiophene 1-oxide **4a** (18 mg, 0.110 mmol), 2-methoxy-4-methylphenol (14 mg, 0.100 mmol), and 1,3,5-trimethoxybenzene (25 mg, 0.150 mmol), TFAA (23 *u*L, 0.165 mmol), and CH_2Cl_2 (2 mL), gave **3v** (15 mg, 0.051 mmol, 51%) as a white solid; ^{1}H NMR (400 MHz, $CDCl_3$) δ = 2.31 (s, 3H, C*H₃*), 3.74 (s, 6H, OCH_3), 3.86 (s, 3H, OCH_3), 3.89 (s, 3H, OCH_3), 5.36 (s, 1H, O*H*), 6.24 (s, 2H, ArC*H*), 6.59 (s, 1H, ArC*H*), 6.68 (s, 1H, ArC*H*) ppm; ^{13}C NMR (100 MHz, $CDCl_3$) δ = 21.4 (CH_3), 55.5 (OCH_3), 55.9 (OCH_3), 56.2 (OCH_3), 91.3 (Ar*C*H), 107.6 (Ar*C*), 111.2 (Ar*C*H), 120.6 (Ar*C*), 124.8 (Ar*C*H), 128.5 (Ar*C*), 141.4 (Ar*C*), 146.8 (Ar*C*), 158.9 (Ar*C*), 161.2 (Ar*C*) ppm.

2',3,4',5'-Tetramethoxy-5-methyl-[1,1'-biphenyl]-2-ol (3w)[2]

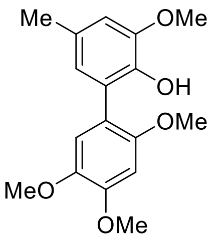

As described in general procedure **A**, 3-methylbenzo[*b*]thiophene 1-oxide **4a** (18 mg, 0.110 mmol), 2-methoxy-4-methylphenol (14 mg, 0.100 mmol), and 1,3,4-trimethoxybenzene (25 mg, 0.150 mmol), TFAA (23 *u*L, 0.165 mmol), and CH_2Cl_2 (2 mL), gave **3w** (16 mg, 0.053 mmol, 53%) as a white solid; ^{1}H NMR (400 MHz, $CDCl_3$) δ = 2.33 (s, 3H, CH_3), 3.80 (s, 3H, OCH_3), 3.86 (s, 3H, OCH_3), 3.91 (s, 3H, OCH_3), 3.94 (s, 3H, OCH_3), 5.96 (s, 1H, O*H*), 6.65 (s, 1H, ArC*H*), 6.69 (s, 1H, ArC*H*), 6.71 (s, 1H, ArC*H*), 6.85 (s, 1H, ArC*H*) ppm; ^{13}C NMR (100 MHz, $CDCl_3$) δ = 21.3 (CH_3), 56.2 (OCH_3), 56.3 (OCH_3), 56.6 (OCH_3), 57.5 (OCH_3), 98.6 (Ar*C*H), 111.4 (Ar*C*H), 115.2 (Ar*C*H), 118.7 (Ar*C*), 123.6 (Ar*C*H), 125.4 (Ar*C*), 129.4 (Ar*C*), 141.0 (Ar*C*), 143.8 (Ar*C*), 147.6 (Ar*C*), 149.4 (Ar*C*), 150.5 (Ar*C*) ppm.

---

[9] Gaster, E.; Vainer, Y.; Regev, A.; Narute, S.; Sudheendran, K.; Werbeloff, A.; Shalit, H.; Pappo, D. *Angew. Chem., Int. Ed.* **2015**, *54*, 4198.

2-(*tert*-Butyl)-4-methoxy-6-(2-methoxynaphthalen-1-yl)phenol (3x)[7]

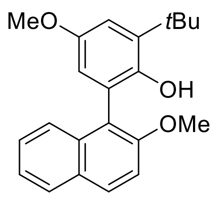

As described in general procedure **A**, 3-methylbenzo[*b*]thiophene 1-oxide **4a** (18 mg, 0.110 mmol), 2-(*tert*-butyl)-4-methoxyphenol (18 mg, 0.100 mmol), and 2-methoxynaphthalene (24 mg, 0.150 mmol), TFAA (23 *u*L, 0.165 mmol), and CH_2Cl_2 (2 mL), gave **3x** (17 mg, 0.051 mmol, 51%) as a white solid; ^{1}H NMR (400 MHz, $CDCl_3$) δ = 1.47 (s, 9H, C(C*H*$_3$)$_3$), 3.76 (s, 3H, OC*H*$_3$), 3.91 (s, 3H, OC*H*$_3$), 4.78 (s, 1H, O*H*), 6.62 (d, *J* = 3.2 Hz, 1H, ArC*H*), 7.01 (d, *J* = 2.8 Hz, 1H, ArC*H*), 7.38-7.42 (m, 3H, ArC*H*), 7.50-7.52 (m, 1H, ArC*H*), 7.83-7.86 (m, 1H, ArC*H*), 7.96 (d, *J* = 9.2 Hz, 1H, ArC*H*) ppm; ^{13}C NMR (100 MHz, $CDCl_3$) δ = 29.7 (C(*C*H$_3$)$_3$), 35.4 (*C*(CH$_3$)$_3$), 55.7 (O*C*H$_3$), 56.9 (O*C*H$_3$), 113.0 (Ar*C*H), 113.8 (Ar*C*H), 114.2 (Ar*C*H), 119.2 (Ar*C*), 123.3 (Ar*C*), 124.2 (Ar*C*H), 125.2 (Ar*C*H), 127.2 (Ar*C*H), 128.2 (Ar*C*H), 129.6 (Ar*C*), 130.6 (Ar*C*H), 133.9 (Ar*C*), 137.0 (Ar*C*), 146.6 (Ar*C*), 152.6 (Ar*C*), 154.8 (Ar*C*) ppm.

3-(*tert*-Butyl)-2',4',5,5'-tetramethoxy-[1,1'-biphenyl]-2-ol (3y)[2]

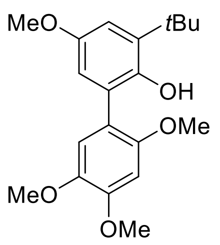

As described in general procedure **A**, 3-methylbenzo[*b*]thiophene 1-oxide **4a** (18 mg, 0.110 mmol), 2-(*tert*-butyl)-4-methoxyphenol (18 mg, 0.100 mmol), and 1,3,4-trimethoxybenzene (25 mg, 0.150 mmol), TFAA (23 *u*L, 0.165 mmol), and CH_2Cl_2 (2 mL), gave **3y** (31 mg, 0.090 mmol, 90%) as a white solid; ^{1}H NMR (400 MHz, $CDCl_3$) δ = 1.45 (s, 9H, C(C*H*$_3$)$_3$), 3.80 (s, 3H, OC*H*$_3$), 3.82 (s, 3H, OC*H*$_3$), 3.87 (s, 3H, OC*H*$_3$), 3.95 (s, 3H, OC*H*$_3$), 6.09 (s, 1H, O*H*), 6.66 (s, 2H, ArC*H*), 6.86 (s, 1H, ArC*H*), 6.92 (s, 1H, ArC*H*) ppm; ^{13}C NMR (100 MHz, $CDCl_3$) δ = 29.8 (C(*C*H$_3$)$_3$), 35.3 (*C*(CH$_3$)$_3$), 55.8 (O*C*H$_3$), 56.4 (O*C*H$_3$), 56.7 (O*C*H$_3$), 57.7 (O*C*H$_3$), 98.7 (Ar*C*H), 112.8 (Ar*C*H), 113.5 (Ar*C*H), 115.7 (Ar*C*H), 119.3 (Ar*C*), 127.4 (Ar*C*), 139.3 (Ar*C*), 144.5 (Ar*C*), 146.6 (Ar*C*), 149.79 (Ar*C*), 149.85 (Ar*C*), 153.0 (Ar*C*) ppm.

4-Methoxy-2-(2-methoxynaphthalen-1-yl)phenol (3z)[10]

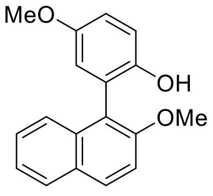

As described in general procedure **A**, 3-methylbenzo[*b*]thiophene 1-oxide **4a** (18 mg, 0.110 mmol), 4-methoxyphenol (12 mg, 0.100 mmol), and 2-methoxynaphthalene (24 mg, 0.150 mmol), TFAA (23 *u*L, 0.165 mmol), and CH_2Cl_2 (2 mL), gave **3z** (20 mg, 0.071 mmol, 71%) as a white solid; ^{1}H NMR (400 MHz, $CDCl_3$) δ = 3.78 (s, 3H, OC*H3*), 3.91 (s, 3H, OC*H3*), 4.70 (s, 1H, O*H*), 6.79 (d, *J* = 2.8 Hz, 1H, ArC*H*), 6.94 (d, *J* = 8.8, 2.8 Hz, 1H, ArC*H*), 7.04 (d, *J* = 8.8 Hz, 1H, ArC*H*), 7.36-7.42 (m, 3H, ArC*H*), 7.54-7.56 (m, 1H, ArC*H*), 7.84-7.86 (m, 1H, ArC*H*), 7.95 (d, *J* = 8.8 Hz, 1H, ArC*H*) ppm; ^{13}C NMR (100 MHz, $CDCl_3$) δ = 55.9 (O*C*H3), 56.9 (O*C*H3), 113.6 (Ar*C*H), 115.2 (Ar*C*H), 116.93 (Ar*C*H), 116.95 (Ar*C*H), 118.8 (Ar*C*), 123.2 (Ar*C*), 124.2 (Ar*C*H), 125.1 (Ar*C*H), 127.2 (Ar*C*H), 128.2 (Ar*C*H), 129.6 (Ar*C*), 130.6 (Ar*C*H), 133.7 (Ar*C*), 148.0 (Ar*C*), 153.5 (Ar*C*), 154.5 (Ar*C*) ppm.

2-(2,6-Dimethoxynaphthalen-1-yl)-4-methoxyphenol (3aa)

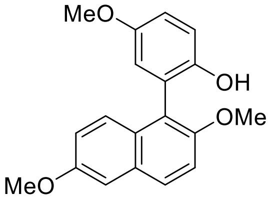

As described in general procedure **A**, 3-methylbenzo[*b*]thiophene 1-oxide **4a** (18 mg, 0.110 mmol), 4-methoxyphenol (12 mg, 0.100 mmol), and 2,6-dimethoxynaphthalene (28 mg, 0.150 mmol), TFAA (23 *u*L, 0.165 mmol), and CH_2Cl_2 (2 mL), gave **3aa** (23 mg, 0.074 mmol, 74%) as a white solid; m.p: 111-113 ºC; ^{1}H NMR (400 MHz, $CDCl_3$) δ = 3.78 (s, 3H, OC*H3*), 3.87 (s, 3H, OC*H3*), 3.91 (s, 3H, OC*H3*), 4.76 (s, 1H, O*H*), 6.77 (d, *J* = 3.2 Hz, 1H, ArC*H*), 6.93 (dd, *J* = 8.6, 3.4 Hz, 1H, ArC*H*), 7.03 (d, *J* = 8.8 Hz, 1H, ArC*H*), 7.08 (dd, *J* = 9.2 Hz, 1H, ArC*H*), 7.15 (d, *J* = 2.4 Hz, 1H, ArC*H*), 7.36 (d, *J* = 8.8 Hz, 1H, ArC*H*), 7.47 (d, *J* = 9.2 Hz, 1H, ArC*H*), 7.83 (d, *J* = 8.8 Hz, 1H, ArC*H*) ppm; ^{13}C NMR (100 MHz, $CDCl_3$) δ = 55.5 (O*C*H3), 55.8 (O*C*H3), 57.2

[10] Dohi, T.; Washimi, N.; Kamitanaka, T.; Fukushima, K.; Kita, Y. *Angew. Chem., Int. Ed.* **2011**, *50*, 6142.

(OCH_3), 106.1 (Ar*C*H), 114.3 (Ar*C*H), 115.2 (Ar*C*H), 116.96 (Ar*C*H), 117.02 (Ar*C*H), 119.4 (Ar*C*), 120.0 (Ar*C*H), 123.4 (Ar*C*), 126.8 (Ar*C*H), 129.0 (Ar*C*), 129.1 (Ar*C*H), 130.7 (Ar*C*), 148.0 (Ar*C*), 152.9 (Ar*C*), 153.4 (Ar*C*), 156.5 (Ar*C*) ppm. **ν_{max}** (neat)/cm^{-1} 735, 827, 851, 1035, 1065, 1169, 1251, 1337, 1376, 1495, 1508, 1596, 1627, 2836, 2940, 3000, 3454; **HRMS** (ESI): Calcd. for $C_{19}H_{19}O_4$ ($M+H^+$), 311.1278; found 311.1268.

2',4',5,5'-Tetramethoxy-[1,1'-biphenyl]-2-ol (3ab)[2]

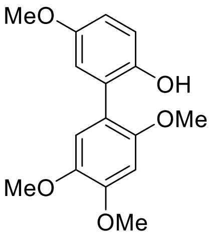

As described in general procedure **A**, 3-methylbenzo[*b*]thiophene 1-oxide **4a** (18 mg, 0.110 mmol), 4-methoxyphenol (12 mg, 0.100 mmol), 1,3,4-trimethoxybenzene (25 mg, 0.150 mmol), TFAA (23 *u*L, 0.165 mmol), and CH_2Cl_2/TFA (1 mL/1 mL), gave **3ab** (18 mg, 0.062 mmol, 62%) as a white solid; ^1H NMR (400 MHz, $CDCl_3$) δ = 3.80 (s, 3H, OCH_3), 3.85 (s, 3H, OCH_3), 3.87 (s, 3H, OCH_3), 3.95 (s, 3H, OCH_3), 6.23 (s, 1H, O*H*), 6.66 (s, 1H, ArC*H*), 6.82-6.86 (m, 3H, ArC*H*), 6.97 (d, *J* = 8.8 Hz, 1H, ArC*H*) ppm; ^{13}C NMR (100 MHz, $CDCl_3$) δ = 55.9 (OCH_3), 56.4 (OCH_3), 56.6 (OCH_3), 57.9 (OCH_3), 98.8 (Ar*C*H), 114.2 (Ar*C*H), 115.3 (Ar*C*H), 116.3 (Ar*C*H), 118.5 (Ar*C*H), 119.0 (Ar*C*), 127.0 (Ar*C*), 144.6 (Ar*C*), 147.8 (Ar*C*), 149.6 (Ar*C*), 149.9 (Ar*C*), 153.9 (Ar*C*) ppm. (The reaction scale up to 10 mmol, yielded **3ab** in 55% yield.)

2',4',5-Trimethoxy-[1,1'-biphenyl]-2-ol (3ac)[11]

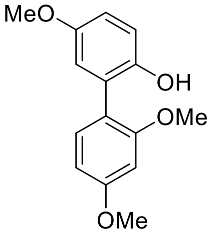

As described in general procedure **A**, 3-methylbenzo[*b*]thiophene 1-oxide **4a** (18 mg, 0.110 mmol), 4-methoxyphenol (12 mg, 0.100 mmol), 1,3-dimethoxybenzene (21 mg, 0.150 mmol), TFAA (23 *u*L, 0.165 mmol), and CH_2Cl_2/TFA (1 mL/1 mL), gave **3ac** (8 mg, 0.030 mmol, 30%) as colorless oil; ^1H NMR (400 MHz, $CDCl_3$) δ = 3.79 (s, 3H,

---

[11] T. Dohi, T. Kamitanaka, S. Watanabe, Y. Hu, N. Washimi, Y. Kita, *Chem. Eur. J.* **2012**, *18*, 13614.

OCH_3), 3.87 (s, 3H, OCH_3), 3.88 (s, 3H, OCH_3), 5.85 (s, 1H, O*H*), 6.61 (d, *J* = 2.4 Hz, 1H, ArC*H*), 6.66 (dd, *J* = 8.4, 2.4 Hz, 1H, ArC*H*), 6.77 (d, *J* = 2.8 Hz, 1H, ArC*H*), 6.84 (dd, *J* = 8.8, 2.8 Hz, 1H, ArC*H*), 6.94 (d, *J* = 8.8 Hz, 1H, ArC*H*), 7.25-7.27 (m, 1H, ArC*H*) ppm; ^{13}C NMR (100 MHz, $CDCl_3$) δ = 55.7 (O*C*H$_3$), 55.9 (O*C*H$_3$), 56.3 (O*C*H$_3$), 99.3 (Ar*C*H), 106.4 (Ar*C*H), 114.5 (Ar*C*H), 116.4 (Ar*C*H), 118.1 (Ar*C*H), 119.7 (Ar*C*), 126.9 (Ar*C*), 133.0 (Ar*C*H), 147.8 (Ar*C*), 153.9 (Ar*C*), 159.6 (Ar*C*), 161.1 (Ar*C*) ppm.

2,2'',4,4'',5,5',5''-Heptamethoxy-[1,1':3',1''-terphenyl]-2'-ol (3ab')

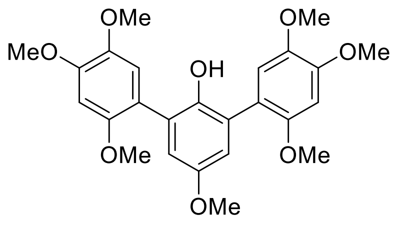

As described in general procedure **A**, 3-methylbenzo[*b*]thiophene 1-oxide **4a** (18 mg, 0.110 mmol), 4-methoxyphenol (12 mg, 0.100 mmol), 1,3,4-trimethoxybenzene (33 mg, 0.20 mmol), TFAA (23 *u*L, 0.165 mmol), and CH_2Cl_2 (2 mL), gave **3ab'** (37 mg, 0.080 mmol, 80%) as a white solid; m.p: 167-169 ºC; ^{1}H NMR (400 MHz, $CDCl_3$) δ = 3.81 (s, 6H, OCH_3), 3.82 (s, 3H, OCH_3), 3.87 (s, 6H, OCH_3), 3.94 (s, 6H, OCH_3), 6.29 (s, 1H, O*H*), 6.65 (s, 2H, ArC*H*), 6.86 (s, 2H, ArC*H*), 6.93 (s, 2H, ArC*H*) ppm; ^{13}C NMR (100 MHz, $CDCl_3$) δ = 55.9 (O*C*H$_3$), 56.3 (O*C*H$_3$), 56.6 (O*C*H$_3$), 57.4 (O*C*H$_3$), 98.5 (Ar*C*H), 115.4 (Ar*C*H), 116.0 (Ar*C*H), 119.5 (Ar*C*), 127.9 (Ar*C*), 143.8 (Ar*C*), 145.4 (Ar*C*), 149.5 (Ar*C*), 150.5 (Ar*C*), 153.1 (Ar*C*) ppm. **ν_{max}** (neat)/cm^{-1} 702, 734, 854, 1031, 1099, 1203, 1267, 1329, 1393, 1436, 1462, 1512, 1611, 2841, 2936, 3399; **HRMS** (ESI): Calcd. for $C_{25}H_{28}O_8Na$ (M+Na$^+$), 479.1676; found 479.1659.

2,2'',4,4'',5'-Pentamethoxy-[1,1':3',1''-terphenyl]-2'-ol (3ac')[12]

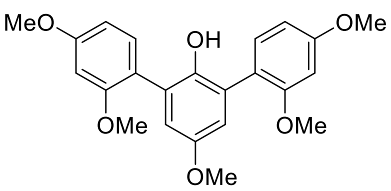

As described in general procedure **A**, 3-methylbenzo[*b*]thiophene 1-oxide **4a** (18 mg, 0.110 mmol), 4-methoxyphenol (12 mg, 0.100 mmol), 1,3-dimethoxybenzene (28 mg, 0.20 mmol), TFAA (23 *u*L, 0.165 mmol), and CH_2Cl_2 (2 mL), gave **3ac'** (20 mg, 0.051 mmol, 51%) as a pink oil; ^{1}H NMR (400 MHz, $CDCl_3$) δ = 3.80 (s, 3H, OC*H₃*), 3.83 (s, 6H, OC*H₃*), 3.86 (s, 6H, OC*H₃*), 6.59 (d, *J* = 2.4 Hz, 2H, ArC*H*), 6.62 (dd, *J* = 2.4 Hz, 2H, ArC*H*), 6.82 (s, 2H, ArC*H*), 7.31 (d, *J* = 8.0 Hz, 2H, ArC*H*) ppm; ^{13}C NMR (100 MHz, $CDCl_3$) δ = 55.6 (O*C*H₃), 55.8 (O*C*H₃), 56.1 (O*C*H₃), 99.0 (Ar*C*H), 105.3 (Ar*C*H), 116.1 (Ar*C*H), 120.5 (Ar*C*), 127.6 (Ar*C*), 132.6 (Ar*C*H), 145.4 (Ar*C*), 153.1 (Ar*C*), 157.3 (Ar*C*), 160.7 (Ar*C*) ppm.

General Procedure B. Oxidative Coupling of Phenols with 1,3-Diketones

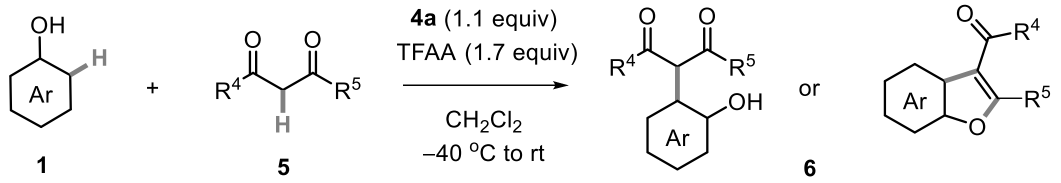

3-Methylbenzo[*b*]thiophene 1-oxide **4a** (0.11 mmol, 1.1 equiv) was dissolved in CH_2Cl_2 (1 mL, indicated if different) in an oven dried tube flushed with N_2. TFAA (0.17 mmol, 1.7 equiv) was then added at −40 ºC. After 5 min at the same temperature, nucleophile **1** (0.10 mmol in 0.5 mL CH_2Cl_2, indicated if different) was added in one portion. 1,3-Diketone **5** (0.15 mmol in 0.5 mL CH_2Cl_2, indicated if different) was then added immediately. After 15 min at −40 ºC, the mixture was warmed to room temperature and stirred for 2 h. Saturated aqueous $NaHCO_3$ (0.1 mL) was then added and the aqueous phase was extracted with CH_2Cl_2 (3 × 3 mL). The combined organic layers were dried over $MgSO_4$ and concentrated *in vacuo*. The crude product

[12] Sharma, S.; Parumala, S. K. R.; Peddinti, R. K. *J. Org. Chem.* **2017**, *82*, 9367.

was purified by column chromatography on silica gel eluting with *n*-hexane in EtOAc (indicated if different eluent was used).

2-(4-Hydroxy-3,5-dimethoxyphenyl)-1,3-diphenylpropane-1,3-dione (6a)

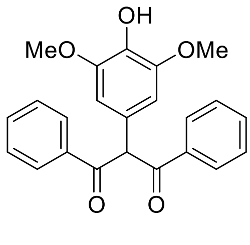

As described in general procedure **B**, 3-methylbenzo[*b*]thiophene 1-oxide **4a** (18 mg, 0.110 mmol), 2,6-dimethoxyphenol (15 mg, 0.100 mmol), and 1,3-diphenylpropane-1,3-dione (33 mg, 0.150 mmol), TFAA (23 *u*L, 0.165 mmol), and CH_2Cl_2 (2 mL), gave **6a** (27 mg, 0.085 mmol, 85%) as a yellow solid; m.p: 100-103 ºC; ^{1}H NMR (500 MHz, $CDCl_3$) δ = 3.87 (s, 6H, OC*H₃*), 5.52 (s, 1H, O*H*), 6.46 (s, 1H, C*H*), 6.59 (s, 2H, ArC*H*), 7.42 (t, *J* = 7.5 Hz, 4H, ArC*H*), 7.56 (t, *J* = 7.2 Hz, 2H, ArC*H*), 7.97 (d, *J* = 7.5 Hz, 4H, ArC*H*) ppm; ^{13}C NMR (125 MHz, $CDCl_3$) δ = 56.6 (O*C*H₃), 62.8 (*C*H), 106.9 (Ar*C*H), 124.0 (Ar*C*), 128.8 (Ar*C*H), 129.0 (Ar*C*H), 133.7 (Ar*C*H), 134.9 (Ar*C*), 136.0 (Ar*C*), 147.5 (Ar*C*), 194.3 (*C*=O) ppm. $\mathbf{v_{max}}$ (neat)/cm^{-1} 733, 787, 800, 1001, 1111, 1206, 1258, 1285, 1320, 1429, 1447, 1462, 1515, 1580, 1595, 1613, 1668, 1699, 2842, 2939, 3448; **HRMS** (ESI): Calcd. for $C_{23}H_{20}O_5Na$ (M+Na$^+$), 399.1203; found 399.1189.

2-(4-Hydroxy-3,5-dimethoxyphenyl)-1,3-bis(4-methoxyphenyl)propane-1,3-dione (6b)

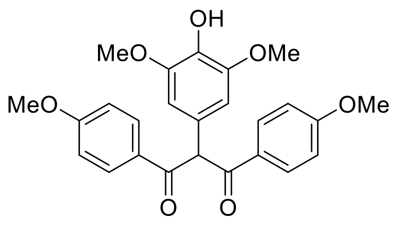

As described in general procedure **B**, 3-methylbenzo[*b*]thiophene 1-oxide **4a** (18 mg, 0.110 mmol), 2,6-dimethoxyphenol (15 mg, 0.100 mmol), and 1,3-bis(4-methoxyphenyl)propane-1,3-dione (43 mg, 0.150 mmol), TFAA (23 *u*L, 0.165 mmol), and CH_2Cl_2 (2 mL), gave **6b** (25 mg, 0.062 mmol, 62%) as a yellow solid; m.p: 155-157 ºC; ^{1}H NMR (400 MHz, $CDCl_3$) δ = 3.84 (s, 6H, OC*H₃*), 3.86 (s, 6H, OC*H₃*), 5.52 (s, 1H, O*H*), 6.37 (s, 1H, C*H*), 6.58 (s, 2H, ArC*H*), 6.90 (d, *J* = 8.8 Hz, 4H, ArC*H*), 7.95 (d, *J* = 8.8 Hz, 4H, ArC*H*) ppm; ^{13}C NMR (100 MHz, $CDCl_3$) δ = 55.6 (O*C*H₃), 56.5

(OCH_3), 62.4 (*C*H), 106.8 (Ar*C*H), 114.2 (Ar*C*H), 124.7 (Ar*C*), 129.1 (Ar*C*), 131.1 (Ar*C*H), 134.7 (Ar*C*), 147.4 (Ar*C*), 163.9 (Ar*C*), 193.0 (*C*=O) ppm. **ν_{max}** (neat)/cm^{-1} 734, 813, 846, 1020, 1112, 1168, 1210, 1260, 1316, 1462, 1511, 1574, 1599, 1662, 1687, 2839, 2936, 3440; **HRMS** (ESI): Calcd. for $C_{25}H_{23}O_7$ ($M-H^+$), 435.1449; found 435.1451.

Ethyl 2-(4-hydroxy-3,5-dimethoxyphenyl)-3-oxo-3-phenylpropanoate (6c)

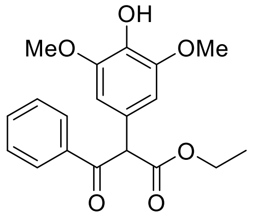

As described in general procedure **B**, 3-methylbenzo[*b*]thiophene 1-oxide **4a** (18 mg, 0.110 mmol), 2,6-dimethoxyphenol (15 mg, 0.100 mmol), and ethyl 3-oxo-3-phenylpropanoate (29 mg, 0.150 mmol), TFAA (23 *u*L, 0.165 mmol), and CH_2Cl_2 (2 mL), gave **6c** (13 mg, 0.038 mmol, 38%) as a yellow oil; ^1H NMR (400 MHz, $CDCl_3$) δ = 1.25 (t, J = 7.2 Hz, 3H, $CO_2CH_2CH_3$), 3.87 (s, 6H, OCH_3), 4.21-4.26 (m, 2H, $CO_2CH_2CH_3$), 5.49-5.51 (m, 2H, C*H*+O*H*), 6.62 (s, 2H, ArC*H*), 7.43 (t, J = 7.6 Hz, 2H, ArC*H*), 7.55 (t, J = 7.4 Hz, 1H, ArC*H*), 7.96 (d, J = 7.6 Hz, 2H, ArC*H*) ppm; ^{13}C NMR (100 MHz, $CDCl_3$) δ = 14.2 ($CO_2CH_2CH_3$), 56.5 (OCH_3), 60.5 (*C*H), 61.9 ($CO_2CH_2CH_3$), 106.6 (Ar*C*H), 123.9 (Ar*C*), 128.9 (Ar*C*H), 129.0 (Ar*C*H), 133.7 (Ar*C*H), 135.0 (Ar*C*), 136.0 (Ar*C*), 147.3 (Ar*C*), 169.1 ($CO_2CH_2CH_3$), 193.6 (*C*=O) ppm. **ν_{max}** (neat)/cm^{-1} 734, 912, 1009, 1113, 1156, 1217, 1264, 1430, 1448, 1463, 1517, 1615, 1682, 1745, 2844, 2939, 3524; **HRMS** (ESI): Calcd. for $C_{19}H_{21}O_6$ ($M+H^+$), 345.1333; found 345.1320.

2-(4-Hydroxy-3,5-dimethoxyphenyl)-1-phenylbutane-1,3-dione (6d)

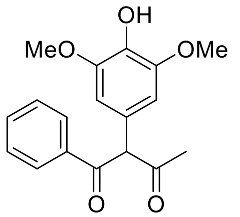

As described in general procedure **B**, 3-methylbenzo[*b*]thiophene 1-oxide **4a** (18 mg, 0.110 mmol), 2,6-dimethoxyphenol (15 mg, 0.100 mmol), and 1-phenylbutane-1,3-dione (24 mg, 0.150 mmol), TFAA (23 *u*L, 0.165 mmol), and CH_2Cl_2 (2 mL), gave **6d** (22 mg, 0.070 mmol, 70%) as a light yellow oil; ketone/enol = 0.7:1.0; ^1H NMR

(400 MHz, $CDCl_3$) δ = 2.10 (s, 3H, CH_3, enol form), 2.27 (s, 3H, CH_3, ketone form), 3.75 (s, 6H, OCH_3, enol form), 3.87 (s, 6H, OCH_3, ketone form), 5.52 (s, 1H, O*H*, enol form), 5.53 (s, 1H, O*H*, ketone form), 5.56 (s, 1H, C*H*, ketone form), 6.33 (s, 2H, ArC*H*, enol form), 6.53 (s, 2H, ArC*H*, ketone form), 7.18 (t, *J* = 7.6 Hz, 2H, ArC*H*, enol form), 7.26-7.31 (m, 3H, ArC*H*, enol form), 7.44 (t, *J* = 7.6 Hz, 2H, ArC*H*, ketone form), 7.55 (t, *J* = 7.4 Hz, 1H, ArC*H*, ketone form), 7.44 (d, *J* = 7.2 Hz, 2H, ArC*H*, ketone form) ppm; ^{13}C NMR (100 MHz, $CDCl_3$) δ = 25.7 (CH$_3$, enol form), 29.3 (CH$_3$, ketone form), 56.5 (OCH$_3$, enol form), 56.6 (OCH$_3$, ketone form), 68.1 (*C*H, ketone form), 106.2 (Ar*C*H), 108.8 (Ar*C*H), 114.5 (Ar*C*), 124.4 (Ar*C*), 127.9 (Ar*C*H), 128.0 (Ar*C*), 128.89 (Ar*C*H), 128.93 (Ar*C*H), 129.0 (Ar*C*H), 130.5 (Ar*C*H), 133.7 (Ar*C*H), 134.2 (Ar*C*), 134.9 (Ar*C*), 136.2 (Ar*C*), 136.3 (Ar*C*), 147.3 (Ar*C*), 147.6 (Ar*C*), 183.0 (C=*C*-OH, enol form), 195.5 (*C*=O, ketone form), 197.0 (*C*=O, enol form), 203.6 (*C*=O, ketone form) ppm. **HRMS** (ESI): Calcd. for $C_{18}H_{18}O_5Na$ (M+Na$^+$), 337.1046; found 337.1036.

(5-Methoxy-2-phenylbenzofuran-3-yl)(phenyl)methanone (6e)

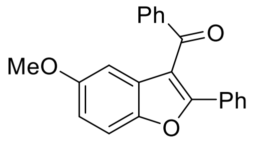

As described in general procedure **B**, 3-methylbenzo[*b*]thiophene 1-oxide **4a** (18 mg, 0.110 mmol), 4-methoxyphenol (12 mg, 0.100 mmol), and 1,3-diphenylpropane-1,3-dione (33 mg, 0.150 mmol), TFAA (23 *u*L, 0.165 mmol), and CH_2Cl_2/TFA (1 mL/1 mL), gave **6e** (18 mg, 0.055 mmol, 55%) as colorless oil; ^{1}H NMR (400 MHz, $CDCl_3$) δ = 3.79 (s, 3H, OCH_3), 6.97 (dd, *J* = 8.6, 2.6 Hz, 1H, ArC*H*), 7.09 (d, *J* = 2.4 Hz, 1H, ArC*H*), 7.24-7.32 (m, 5H, ArC*H*), 7.44-7.48 (m, 2H, ArC*H*), 7.60-7.62 (m, 2H, ArC*H*), 7.80-7.83 (m, 2H, ArC*H*) ppm; ^{13}C NMR (100 MHz, $CDCl_3$) δ = 56.0 (OCH$_3$), 103.5 (Ar*C*H), 111.9 (Ar*C*H), 114.8 (Ar*C*H), 116.4 (Ar*C*), 128.46 (Ar*C*H), 128.49 (Ar*C*H), 128.6 (Ar*C*H), 129.2 (Ar*C*), 129.7 (Ar*C*H), 129.8 (Ar*C*), 130.0 (Ar*C*H), 133.2 (Ar*C*H), 137.9 (Ar*C*), 149.1 (Ar*C*), 156.9 (Ar*C*), 158.8 (Ar*C*), 192.6 (*C*=O) ppm. $\mathbf{v_{max}}$ (neat)/cm^{-1} 738, 803, 901, 1027, 1064, 1171, 1205, 1264, 1373, 1475, 1599, 1642, 3058; **HRMS** (ESI): Calcd. for $C_{22}H_{16}O_3Na$

(M+Na+), 351.0992; found 351.0979.

(7-(*tert*-Butyl)-5-methoxy-2-phenylbenzofuran-3-yl)(phenyl)methanone (6f)

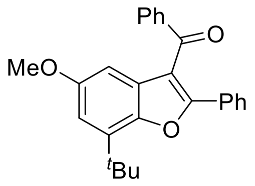

As described in general procedure **B**, 3-methylbenzo[*b*]thiophene 1-oxide **4a** (18 mg, 0.110 mmol), 2-(*tert*-butyl)-4-methoxyphenol (17 mg, 0.100 mmol), and 1,3-diphenylpropane-1,3-dione (33 mg, 0.150 mmol), TFAA (23 *u*L, 0.165 mmol), and CH_2Cl_2/TFA (1 mL/1 mL), gave **6f** (26 mg, 0.068 mmol, 68%) as colorless oil; ^{1}H NMR (400 MHz, $CDCl_3$) δ = 1.57 (s, 9H, C(C*H*$_3$)$_3$), 3.77 (s, 3H, OC*H*$_3$), 6.90 (d, *J* = 2.4 Hz, 1H, ArC*H*), 6.92 (d, *J* = 2.4 Hz, 1H, ArC*H*), 7.27-7.34 (m, 5H, ArC*H*), 7.48 (t, *J* = 7.4 Hz, 1H, ArC*H*), 7.61-7.63 (m, 2H, ArC*H*), 7.85 (d, *J* = 7.6 Hz, 2H, ArC*H*) ppm; ^{13}C NMR (100 MHz, $CDCl_3$) δ = 30.0 (C(*C*H$_3$)$_3$), 34.6 (*C*(CH$_3$)$_3$), 55.9 (O*C*H$_3$), 100.4 (Ar*C*H), 112.5 (Ar*C*H), 116.3 (Ar*C*), 128.46 (Ar*C*H), 128.48 (Ar*C*H), 128.50 (Ar*C*H), 129.4 (Ar*C*), 129.6 (Ar*C*H), 129.9 (Ar*C*H), 130.0 (Ar*C*), 133.2 (Ar*C*H), 136.1 (Ar*C*), 138.0 (Ar*C*), 147.4 (Ar*C*), 156.7 (Ar*C*), 157.5 (Ar*C*), 192.9 (*C*=O) ppm. $\mathbf{v_{max}}$ (neat)/cm^{-1} 738, 807, 892, 905, 1051, 1088, 1201, 1238, 1265, 1412, 1480, 1598, 1645, 2957; **HRMS** (ESI): Calcd. for $C_{26}H_{24}O_3Na$ (M+Na+), 407.1618; found 407.1606.

(7-Chloro-5-methoxy-2-phenylbenzofuran-3-yl)(phenyl)methanone (6g)

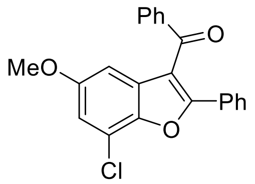

As described in general procedure **B**, 3-methylbenzo[*b*]thiophene 1-oxide **4a** (18 mg, 0.110 mmol), 2-chloro-4-methoxyphenol (16 mg, 0.100 mmol), and 1,3-diphenylpropane-1,3-dione (33 mg, 0.150 mmol), TFAA (23 *u*L, 0.165 mmol), and CH_2Cl_2/TFA (1 mL/1 mL), gave **6g** (27 mg, 0.075 mmol, 75%) as a white solid; m.p: 134-136 ºC; ^{1}H NMR (400 MHz, $CDCl_3$) δ = 3.81 (s, 3H, OC*H*$_3$), 7.03 (br s, 2H, ArC*H*), 7.28-7.35 (m, 5H, ArC*H*), 7.49 (t, *J* = 7.6 Hz, 1H, ArC*H*), 7.65 (d, *J* = 8.0 Hz, 2H, ArC*H*), 7.83 (d, *J* = 7.6 Hz, 2H, ArC*H*) ppm; ^{13}C NMR (100 MHz, $CDCl_3$) δ = 56.2 (O*C*H$_3$), 102.5 (Ar*C*H), 114.7 (Ar*C*H), 116.6 (Ar*C*), 117.0 (Ar*C*), 128.5 (Ar*C*H), 128.6

(Ar*C*H), 128.8 (Ar*C*H), 129.1 (Ar*C*), 129.9 (Ar*C*H), 130.1 (Ar*C*H), 130.2 (Ar*C*), 133.4 (Ar*C*H), 137.6 (Ar*C*), 145.1 (Ar*C*), 157.2 (Ar*C*), 159.3 (Ar*C*), 192.1 (*C*=O) ppm. **ν_{max}** (neat)/cm^{-1} 737, 879, 906, 1036, 1072, 1206, 1264, 1423, 1476, 1590, 1645, 3059; **HRMS** (ESI): Calcd. for $C_{22}H_{15}O_3ClNa$ ($M+Na^+$), 385.0602; found 385.0595.

General Procedure C. Iterative Addition of a Third Nucleophilic Partner

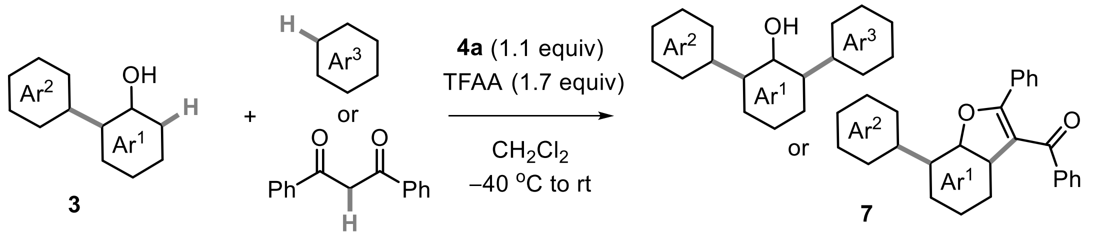

3-Methylbenzo[*b*]thiophene 1-oxide **4a** (0.11 mmol, 1.1 equiv) was dissolved in CH_2Cl_2 (1 mL, indicated if different) in an oven dried tube flushed with N_2. TFAA (0.17 mmol, 1.7 equiv) was then added at −40 ºC. After 5 min at the same temperature, **3** (0.10 mmol in 0.5 mL CH_2Cl_2, indicated if different) was added in one portion. A third nucleophile (0.15 mmol in 0.5 mL CH_2Cl_2, indicated if different) was then added immediately. After 15 min at −40 ºC, the mixture was warmed to room temperature and stirred for 2 h. Saturated aqueous $NaHCO_3$ (0.1 mL) was then added and the aqueous phase was extracted with CH_2Cl_2 (3 × 3 mL). The combined organic layers were dried over $MgSO_4$ and concentrated *in vacuo*. The crude product was purified by column chromatography on silica gel eluting with *n*-hexane in EtOAc (indicated if different eluent was used).

2,2'',4,4'',5,5'-Hexamethoxy-[1,1':3',1''-terphenyl]-2'-ol (7a)

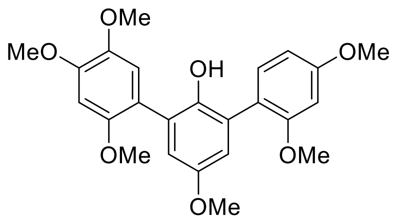

As described in general procedure **C**, 3-methylbenzo[*b*]thiophene 1-oxide **4a** (18 mg, 0.110 mmol), 2',4',5,5'-tetramethoxy-[1,1'-biphenyl]-2-ol **3ab** (29 mg, 0.100 mmol), 1,3-dimethoxybenzene (21 mg, 0.150 mmol), TFAA (23 *u*L, 0.165 mmol), and CH_2Cl_2 (2 mL), gave **7a** (29 mg, 0.068 mmol, 68%) as a pink oil; ^{1}H NMR (400 MHz, $CDCl_3$) δ = 3.81 (br s, 6H, OC*H₃*), 3.83 (s, 3H, OC*H₃*), 3.86 (s, 3H, OC*H₃*), 3.87 (s, 3H, OC*H₃*), 3.94 (s, 3H, OC*H₃*), 6.13 (s, 1H, O*H*), 6.59-6.65 (m, 3H, ArC*H*), 6.83-6.85 (m, 2H, ArC*H*), 6.94 (s, 1H, ArC*H*), 7.30 (d, *J* = 8.4 Hz, 1H, ArC*H*) ppm; ^{13}C NMR (100 MHz, $CDCl_3$) δ = 55.6 (O*C*H₃), 55.9 (O*C*H₃), 56.0 (O*C*H₃), 56.3 (O*C*H₃), 56.6 (O*C*H₃), 57.4 (O*C*H₃), 98.6 (Ar*C*H), 99.1 (Ar*C*H), 105.2 (Ar*C*H), 115.5 (Ar*C*H), 116.0 (Ar*C*H), 116.1 (Ar*C*H), 119.5 (Ar*C*), 120.5 (Ar*C*), 127.6 (Ar*C*), 127.9 (Ar*C*), 132.5 (Ar*C*H), 143.8 (Ar*C*), 145.4 (Ar*C*), 149.5 (Ar*C*), 150.4 (Ar*C*), 153.1 (Ar*C*), 157.4 (Ar*C*), 160.7 (Ar*C*) ppm. ν_{max} (neat)/cm^{-1} 834, 1029, 1158, 1204, 1273, 1302, 1413, 1436, 1461, 1509, 1610, 2836, 2936, 3405; **HRMS** (ESI): Calcd. for $C_{24}H_{26}O_7Na$ (M+Na$^+$), 449.1571; found 449.1555.

2',4',5,5'-Tetramethoxy-3-(2-methoxynaphthalen-1-yl)-[1,1'-biphenyl]-2-ol (7b)

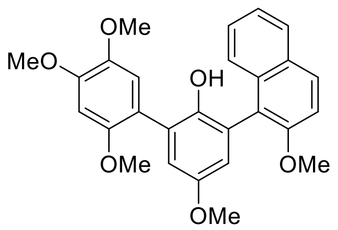

As described in general procedure **C**, 3-methylbenzo[*b*]thiophene 1-oxide **4a** (18 mg, 0.110 mmol), 2',4',5,5'-tetramethoxy-[1,1'-biphenyl]-2-ol **3ab** (29 mg, 0.10 mmol), 2-methoxynaphthalene (24 mg, 0.150 mmol), TFAA (23 *u*L, 0.165 mmol), and CH_2Cl_2 (2 mL), gave **7b** (23 mg, 0.052 mmol, 52%) as a pink oil; ^{1}H NMR (400 MHz, $CDCl_3$) δ = 3.82 (br s, 6H, OC*H₃*), 3.90-3.91 (m, 6H, OC*H₃*), 3.94 (s, 3H, OC*H₃*), 5.90 (s, 1H, O*H*), 6.64 (s, 1H, ArC*H*), 6.85 (d, *J* = 3.2 Hz, 1H, ArC*H*), 6.96 (d, *J* = 3.2 Hz, 1H, ArC*H*), 7.01 (s, 1H, ArC*H*), 7.33-7.42 (m, 3H, ArC*H*), 7.60 (d, *J* = 9.2 Hz, 1H, ArC*H*), 7.83-7.85 (m, 1H, ArC*H*), 7.92 (d, *J* = 9.2 Hz, 1H, ArC*H*) ppm; ^{13}C NMR (100 MHz, $CDCl_3$) δ = 55.9 (O*C*H₃), 56.3

(OCH_3), 56.7 (OCH_3), 57.1 (OCH_3), 57.8 (OCH_3), 98.8 (Ar*C*H), 114.1 (Ar*C*H), 115.5 (Ar*C*H), 116.5 (Ar*C*H), 116.6 (Ar*C*H), 119.5 (Ar*C*), 121.6 (Ar*C*), 123.7 (Ar*C*H), 125.4 (Ar*C*H), 125.6 (Ar*C*), 126.6 (Ar*C*H), 127.4 (Ar*C*), 128.1 (Ar*C*H), 129.3 (Ar*C*), 129.7 (Ar*C*H), 133.7 (Ar*C*), 144.3 (Ar*C*), 145.9 (Ar*C*), 149.7 (Ar*C*), 150.1 (Ar*C*), 153.2 (Ar*C*), 154.4 (Ar*C*) ppm. **ν_{max}** (neat)/cm^{-1} 811, 858, 1028, 1202, 1393, 1435, 1460, 1510, 1593, 2839, 2937, 3381; **HRMS** (ESI): Calcd. for $C_{27}H_{26}O_6Na$ ($M+Na^+$), 469.1622; found 469.1606. (Another method using **3z** and 1,3,4-trimethoxybenzene as substrates, gave **7b** in 70% yield.)

(5-Methoxy-2-phenyl-7-(2,4,5-trimethoxyphenyl)benzofuran-3-yl)(phenyl)methanone (7c)

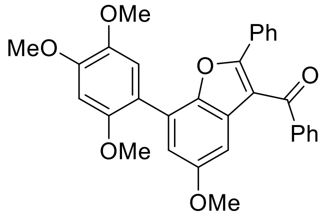

As described in general procedure **C**, 3-methylbenzo[*b*]thiophene 1-oxide **4a** (18 mg, 0.110 mmol), 2',4',5,5'-tetramethoxy-[1,1'-biphenyl]-2-ol **3ab** (29 mg, 0.100 mmol), 1,3-diphenylpropane-1,3-dione (33 mg, 0.150 mmol), TFAA (23 *u*L, 0.165 mmol), CH_2Cl_2 (1 mL), and TFA (1 mL), gave **7c** (30 mg, 0.060 mmol, 60%) as a light brown oil; ^1H NMR (400 MHz, $CDCl_3$) δ = 3.81 (s, 3H, OCH_3), 3.84 (s, 3H, OCH_3), 3.89 (s, 3H, OCH_3), 3.99 (s, 3H, OCH_3), 6.72 (s, 1H, ArC*H*), 7.03 (d, *J* = 2.4 Hz, 1H, ArC*H*), 7.10 (d, *J* = 2.4 Hz, 1H, ArC*H*), 7.14 (s, 1H, ArC*H*), 7.22-7.26 (m, 3H, ArC*H*), 7.32 (t, *J* = 7.6 Hz, 2H, ArC*H*), 7.32 (t, *J* = 7.4 Hz, 1H, ArC*H*), 7.56-7.58 (m, 2H, ArC*H*), 7.86 (t, *J* = 7.2 Hz, 2H, ArC*H*) ppm; ^{13}C NMR (100 MHz, $CDCl_3$) δ = 56.1 (OCH_3), 56.3 (OCH_3), 56.7 (OCH_3). 56.9 (OCH_3), 98.3 (Ar*C*H), 102.4 (Ar*C*H), 115.0 (Ar*C*H), 116.1 (Ar*C*H), 116.3 (Ar*C*), 116.5 (Ar*C*), 123.4 (Ar*C*), 128.4 (Ar*C*H), 128.46 (Ar*C*H), 128.51 (Ar*C*H), 129.2 (Ar*C*), 129.6 (Ar*C*H), 129.8 (Ar*C*), 130.0 (Ar*C*H), 133.2 (Ar*C*H), 138.0 (Ar*C*), 143.3 (Ar*C*), 146.9 (Ar*C*), 149.9 (Ar*C*), 151.7 (Ar*C*), 156.7 (Ar*C*), 158.3 (Ar*C*), 192.7 (C=O) ppm. **ν_{max}** (neat)/cm^{-1} 739, 905, 1035, 1205, 1265, 1395, 1417, 1515, 1597, 2933; **HRMS** (ESI): Calcd. for $C_{31}H_{27}O_6$ ($M+H^+$), 495.1802; found 495.1791.

4-(2-Hydroxy-2',4',5,5'-tetramethoxy-[1,1'-biphenyl]-3-yl)naphthalen-1-ol (7d)

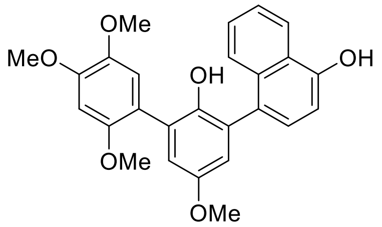

As described in general procedure **C**, 3-methylbenzo[*b*]thiophene 1-oxide **4a** (18 mg, 0.110 mmol), 2',4',5,5'-tetramethoxy-[1,1'-biphenyl]-2-ol **3ab** (29 mg, 0.100 mmol), naphthalen-1-ol (22 mg, 0.150 mmol), TFAA (23 *u*L, 0.165 mmol), and CH_2Cl_2 (2 mL), gave **7d** (15 mg, 0.035 mmol, 35%) as a white solid; m.p: 140-142 ºC; ^{1}H NMR (400 MHz, $CDCl_3$) δ = 3.83 (s, 3H, OC*H3*), 3.85 (s, 3H, OC*H3*), 3.90 (s, 3H, OC*H3*), 3.94 (s, 3H, OC*H3*), 5.69 (s, 1H, O*H*), 5.93 (s, 1H, O*H*), 6.65 (s, 1H, ArC*H*), 6.82 (d, *J* = 7.6 Hz, 1H, ArC*H*), 6.88 (d, *J* = 3.2 Hz, 1H, ArC*H*), 6.94 (d, *J* = 3.2 Hz, 1H, ArC*H*), 6.98 (s, 1H, ArC*H*), 7.32 (d, *J* = 7.6 Hz, 1H, ArC*H*), 7.43-7.51 (m, 2H, ArC*H*), 7.72-7.74 (m, 1H, ArC*H*), 8.22-8.25 (m, 1H, ArC*H*) ppm; ^{13}C NMR (100 MHz, $CDCl_3$) δ = 56.0 (O*C*H3), 56.4 (O*C*H3), 56.7 (O*C*H3), 57.6 (O*C*H3), 98.5 (Ar*C*H), 108.4 (Ar*C*H), 115.5 (Ar*C*H), 116.3 (Ar*C*H), 116.5 (Ar*C*H), 119.0 (Ar*C*), 122.1 (Ar*C*H), 124.6 (Ar*C*), 125.2 (Ar*C*H), 126.4 (Ar*C*H), 126.6 (Ar*C*H), 127.2 (Ar*C*), 127.6 (Ar*C*H), 129.2 (Ar*C*), 129.7 (Ar*C*), 133.1 (Ar*C*), 144.3 (Ar*C*), 145.5 (Ar*C*), 149.8 (Ar*C*), 150.0 (Ar*C*), 151.6 (Ar*C*), 153.3 (Ar*C*) ppm. **ν_{max}** (neat)/cm^{-1} 738, 796, 1027, 1205, 1264, 1388, 1461, 1513, 1588, 2961, 3380; **HRMS** (ESI): Calcd. for $C_{26}H_{23}O_6$ (M-H$^+$), 431.1500; found 431.1493.

1-(2-Hydroxy-2',4',5,5'-tetramethoxy-[1,1'-biphenyl]-3-yl)naphthalen-2-ol (7e)

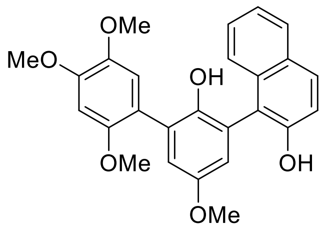

As described in general procedure **C**, 3-methylbenzo[*b*]thiophene 1-oxide **4a** (18 mg, 0.110 mmol), 2',4',5,5'-tetramethoxy-[1,1'-biphenyl]-2-ol **3ab** (29 mg, 0.100 mmol), naphthalen-2-ol (22 mg, 0.150 mmol), TFAA (23 *u*L, 0.165 mmol), and CH_2Cl_2 (2 mL), gave **7e** (14 mg, 0.032 mmol, 32%) as a white solid; m.p: 220-222 ºC; ^{1}H NMR (400 MHz, $CDCl_3$) δ = 3.82 (s, 3H, OC*H3*), 3.85 (s, 3H, OC*H3*), 3.92 (s, 3H, OC*H3*), 3.95 (s, 3H, OC*H3*), 5.61 (s, 1H, O*H*), 6.16 (s, 1H, O*H*), 6.65 (s, 1H, ArC*H*), 6.90 (d, *J* = 3.2 Hz,

1H, ArC*H*), 6.98 (s, 1H, ArC*H*), 7.02 (d, *J* = 3.2 Hz, 1H, ArC*H*), 7.30-7.41 (m, 3H, ArC*H*), 7.59 (d, *J* = 8.4 Hz, 1H, ArC*H*), 7.82-7.85 (m, 2H, ArC*H*) ppm; ^{13}C NMR (100 MHz, $CDCl_3$) δ = 56.0 (O*C*H_3), 56.4 (O*C*H_3), 56.7 (O*C*H_3), 57.6 (O*C*H_3), 98.4 (Ar*C*H), 115.4 (Ar*C*H), 116.5 (Ar*C*H), 117.8 (Ar*C*), 118.0 (Ar*C*H), 118.1 (Ar*C*H), 118.3 (Ar*C*), 122.7 (Ar*C*), 123.5 (Ar*C*H), 125.0 (Ar*C*H), 126.6 (Ar*C*H), 128.3 (Ar*C*H), 128.5 (Ar*C*), 129.3 (Ar*C*), 130.0 (Ar*C*H), 133.3 (Ar*C*), 144.5 (Ar*C*), 146.0 (Ar*C*), 149.9 (Ar*C*), 150.1 (Ar*C*), 151.0 (Ar*C*), 153.9 (Ar*C*) ppm. **ν_{max}** (neat)/cm^{-1} 822, 858, 952, 1029, 1203, 1393, 1457, 1511, 1596, 1611, 2845, 2944, 3012, 3361; **HRMS** (ESI): Calcd. for $C_{26}H_{25}O_6$ (M+H$^+$), 433.1646; found 433.1641.

3-Bromo-1-(2-hydroxy-2',4',5,5'-tetramethoxy-[1,1'-biphenyl]-3-yl)naphthalen-2-ol (7f)

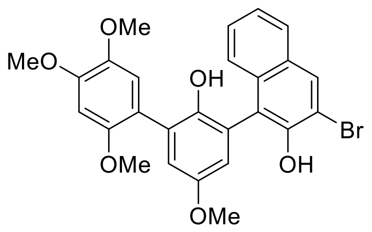

As described in general procedure **C**, 3-methylbenzo[*b*]thiophene 1-oxide **4a** (18 mg, 0.110 mmol), 2',4',5,5'-tetramethoxy-[1,1'-biphenyl]-2-ol **3ab** (29 mg, 0.100 mmol), 3-bromonaphthalen-2-ol (33 mg, 0.150 mmol), TFAA (23 *u*L, 0.165 mmol), and CH_2Cl_2 (2 mL), gave **7f** (15 mg, 0.030 mmol, 30%) as a pink solid; m.p: 159-162 ºC; ^{1}H NMR (400 MHz, $CDCl_3$) δ = 3.82 (s, 3H, OC*H$_3$*), 3.85 (s, 3H, OC*H$_3$*), 3.92 (s, 3H, OC*H$_3$*), 3.95 (s, 3H, OC*H$_3$*), 5.90 (s, 1H, O*H*), 6.25 (s, 1H, O*H*), 6.65 (s, 1H, ArC*H*), 6.86 (d, *J* = 3.2 Hz, 1H, ArC*H*), 6.98 (s, 1H, ArC*H*), 7.02 (d, *J* = 3.2 Hz, 1H, ArC*H*), 7.34-7.41 (m, 2H, ArC*H*), 7.53 (d, *J* = 9.2 Hz, 1H, ArC*H*), 7.75 (d, *J* = 7.8 Hz, 1H, ArC*H*), 8.12 (s, 1H, ArC*H*) ppm; ^{13}C NMR (100 MHz, $CDCl_3$) δ = 56.0 (O*C*H_3), 56.4 (O*C*H_3), 56.7 (O*C*H_3), 57.7 (O*C*H_3), 98.4 (Ar*C*H), 112.4 (Ar*C*), 115.3 (Ar*C*H), 116.1 (Ar*C*H), 118.0 (Ar*C*H), 118.3 (Ar*C*), 119.9 (Ar*C*), 123.2 (Ar*C*), 124.4 (Ar*C*H), 125.3 (Ar*C*H), 126.9 (Ar*C*H), 127.3 (Ar*C*H), 128.5 (Ar*C*), 129.7 (Ar*C*), 131.9 (Ar*C*H), 132.8 (Ar*C*), 144.5 (Ar*C*), 145.9 (Ar*C*), 147.2 (Ar*C*), 149.7 (Ar*C*), 150.1 (Ar*C*), 153.8 (Ar*C*) ppm. **ν_{max}** (neat)/cm^{-1} 866, 1030, 1145, 1204, 1330, 1392, 1439, 1463, 1511, 1611, 2935, 3338; **HRMS** (ESI): Calcd. for $C_{26}H_{23}BrO_6Na$ (M+Na$^+$), 533.0570; found 533.0558.

2',4',5-Trimethoxy-3-(3-methoxynaphthalen-2-yl)-[1,1'-biphenyl]-2-ol (7g)

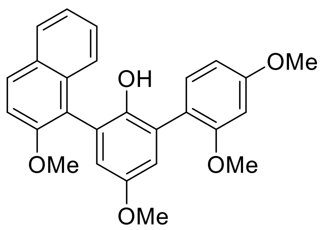

As described in general procedure **C**, 3-methylbenzo[*b*]thiophene 1-oxide **4a** (18 mg, 0.110 mmol), 2',4',5,5'-tetramethoxy-[1,1'-biphenyl]-2-ol **3z** (28 mg, 0.100 mmol), 1,3-dimethoxybenzene (21 mg, 0.150 mmol), TFAA (23 *u*L, 0.165 mmol), and CH_2Cl_2 (2 mL), gave **7g** (30 mg, 0.072 mmol, 72%) as an orange oil; ^{1}H NMR (400 MHz, $CDCl_3$) δ = 3.81 (s, 3H, OCH_3), 3.84 (s, 3H, OCH_3), 3.86 (s, 3H, OCH_3), 3.90 (s, 3H, OCH_3), 5.57 (s, 1H, O*H*), 6.59 (d, *J* = 2.4 Hz, 1H, ArC*H*), 6.66 (dd, *J* = 8.4, 2.4 Hz, 1H, ArC*H*), 6.83 (d, *J* = 2.8 Hz, 1H, ArC*H*), 6.93 (d, *J* = 3.2 Hz, 1H, ArC*H*), 7.33-7.42 (m, 4H, ArC*H*), 7.62 (d, *J* = 8.0 Hz, 1H, ArC*H*), 7.83-7.85 (m, 1H, ArC*H*), 7.91 (d, *J* = 9.2 Hz, 1H, ArC*H*) ppm; ^{13}C NMR (100 MHz, $CDCl_3$) δ = 55.6 (O*C*H$_3$), 55.8 (O*C*H$_3$), 56.2 (O*C*H$_3$), 57.1 (O*C*H$_3$), 99.2 (Ar*C*H), 105.8 (Ar*C*H), 114.1 (Ar*C*H), 116.5 (Ar*C*H), 116.7 (Ar*C*H), 120.3 (Ar*C*), 121.5 (Ar*C*), 123.8 (Ar*C*H), 125.0 (Ar*C*), 125.5 (Ar*C*H), 126.6 (Ar*C*H), 127.3 (Ar*C*), 128.1 (Ar*C*H), 129.3 (Ar*C*), 129.7 (Ar*C*H), 132.8 (Ar*C*H), 133.7 (Ar*C*), 145.9 (Ar*C*), 153.1 (Ar*C*), 154.4 (Ar*C*), 157.0 (Ar*C*), 160.9 (Ar*C*) ppm. ν_{max} (neat)/cm^{-1} 736, 810, 1045, 1088, 1158, 1207, 1266, 1460, 1509, 1611, 2837, 2936, 2999, 3409; **HRMS** (ESI): Calcd. for $C_{26}H_{25}O_5$ (M+H$^+$), 417.1702; found 417.1690.

(5-Methoxy-7-(3-methoxynaphthalen-2-yl)-2-phenylbenzofuran-3-yl)(phenyl)methanone (7h)

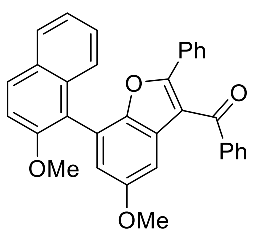

As described in general procedure **C**, 3-methylbenzo[*b*]thiophene 1-oxide **4a** (18 mg, 0.110 mmol), 2',4',5,5'-tetramethoxy-[1,1'-biphenyl]-2-ol **3z** (28 mg, 0.100 mmol), 1,3-diphenylpropane-1,3-dione (33 mg, 0.150 mmol), TFAA (23 *u*L, 0.165 mmol), CH_2Cl_2 (1 mL), and TFA (1 mL), gave **7h** (15 mg, 0.031 mmol, 31%) as a pink oil; ^{1}H NMR (400 MHz, $CDCl_3$) δ = 3.84 (s, 3H, OCH_3), 3.90 (s, 3H, OCH_3), 6.70 (d, *J* = 2.4 Hz, 1H, ArC*H*), 7.11-7.20 (m, 4H, ArC*H*), 7.31-7.50 (m, 8H, ArC*H*), 7.56-7.58 (m, 1H, ArC*H*), 7.87-7.89 (m, 3H,

ArC*H*), 7.99 (d, *J* = 9.2 Hz, 1H, ArC*H*) ppm; ^{13}C NMR (100 MHz, $CDCl_3$) δ = 56.1 (O*C*H$_3$), 57.0 (O*C*H$_3$), 103.1 (Ar*C*H), 113.8 (Ar*C*H), 116.5 (Ar*C*), 117.3 (Ar*C*H), 118.9 (Ar*C*), 121.2 (Ar*C*), 123.8 (Ar*C*H), 125.2 (Ar*C*H), 126.8 (Ar*C*H), 128.1 (Ar*C*H), 128.2 (Ar*C*H), 128.5 (Ar*C*H), 128.6 (Ar*C*H), 129.17 (Ar*C*), 129.23 (Ar*C*), 129.6 (Ar*C*H), 129.7 (Ar*C*), 130.1 (Ar*C*H), 130.2 (Ar*C*H), 133.2 (Ar*C*H), 133.5 (Ar*C*), 138.0 (Ar*C*), 147.9 (Ar*C*), 154.8 (Ar*C*), 156.7 (Ar*C*), 158.8 (Ar*C*), 192.8 (C=O) ppm. $\mathbf{v_{max}}$ (neat)/cm^{-1} 736, 813, 904, 1201, 1267, 1411, 1469, 1597, 1642, 2840, 2956, 3053; **HRMS** (ESI): Calcd. for $C_{33}H_{24}O_4Na$ (M+Na$^+$), 507.1567; found 507.1547.

Mechanistic Studies

I. The importance of an "OH" in the first nucleophilic partner

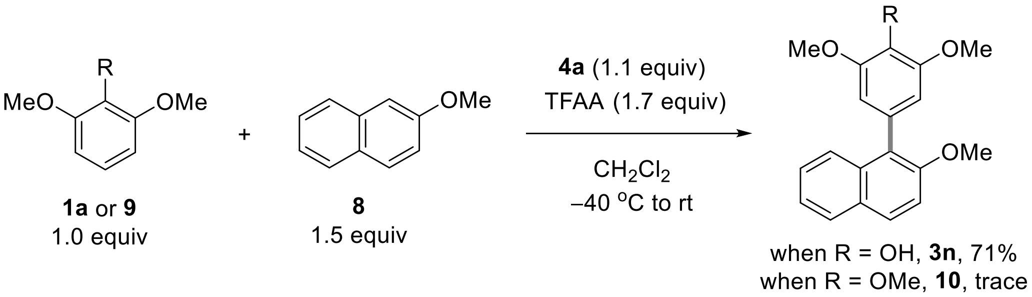

Procedure: Sulfoxide **4a** (0.11 mmol, 1.1 equiv) was dissolved in CH_2Cl_2 (1 mL) in an oven dried tube flushed with N_2. TFAA (0.17 mmol, 1.7 equiv) was then added at −40 ºC. After 5 min at the same temperature, nucleophile **1a** or **9** (0.1 mmol in 0.5 mL CH_2Cl_2) was added in one portion. Nucleophile **8** (0.15 mmol in 0.5 mL CH_2Cl_2) was then added immediately. After 15 min at −40 ºC, the mixture was warmed to room temperature and stirred for 2 h. Saturated aqueous $NaHCO_3$ was then added and the aqueous phase was extracted with CH_2Cl_2 (3 × 3 mL). The combined organic layers were dried over $MgSO_4$ and concentrated *in vacuo*. When R was OH, 71% of the desired product **3t** was obtained. However, when R was OMe, only a trace amount of the desired product **10** was observed.

II. The importance of the order of addition of nucleophiles

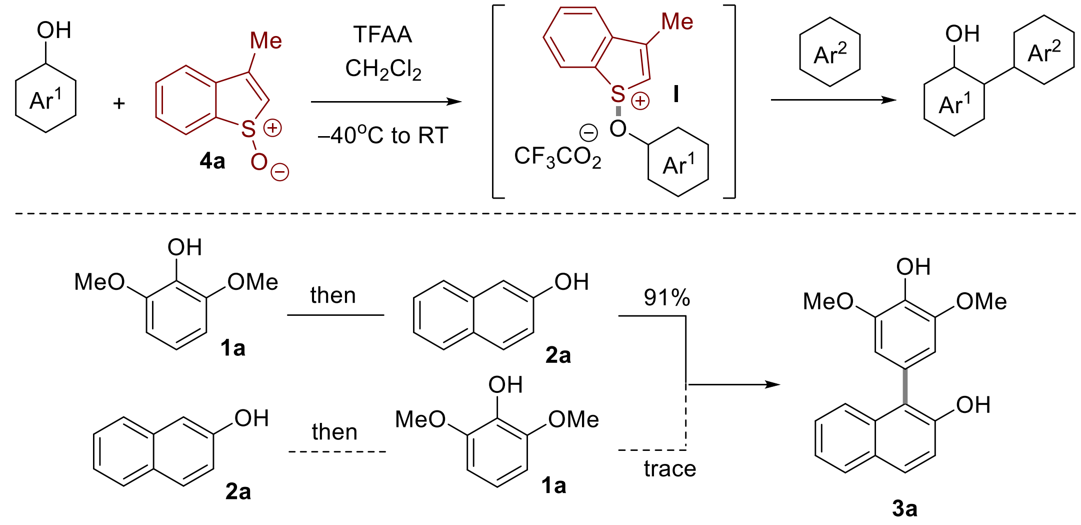

Procedure: Sulfoxide **4a** (0.11 mmol, 1.1 equiv) was dissolved in CH_2Cl_2 (1 mL) in an oven dried tube flushed with N_2. TFAA (0.17 mmol, 1.7 equiv) was then added at −40 ºC. After 5 min at the same temperature, nucleophile **1a** (top reaction) or **2a** (bottom reaction) (0.1 mmol in 0.5 mL CH_2Cl_2) was added in one portion. Nucleophile **2a** (top reaction) or **1a** (bottom reaction) (0.15 mmol in 0.5 mL CH_2Cl_2) was then added immediately. After 15 min at −40 ºC, the mixture was warmed to room temperature and stirred for 2 h. Saturated aqueous $NaHCO_3$ was then added and the aqueous phase was extracted with CH_2Cl_2 (3 × 3 mL). The combined organic layers were dried over $MgSO_4$ and concentrated *in vacuo*. When compound **1a** was added first, 91% of **3a** was obtained. However, when compound **2a** was added first, only a trace amount of **3a** was observed.

X-Ray Structures and CCDC Numbers

X-ray structure of 3r

CCDC 1944706

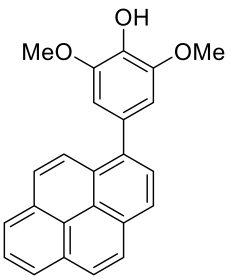

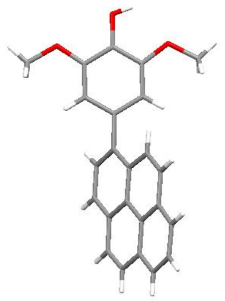

Table S2. Crystal data and details of data collection and refinement for compound **3r**

Bond precision	C–C = 0.0027 A	Wavelength	0.71073
Cell:	a = 21.6130 (13)	b = 12.1859 (7)	c = 13.5922 (7)
	alpha = 90	beta = 104.043 (6)	gamma = 90
Temperature	150 K		
	Calculated	Reported	
Volume	3472.8 (4)	3472.8 (4)	
Space group	C2/c	C12/c1	
Hall group	-C 2yc	-C 2yc	
Moiety formula	$C_{24}H_{18}O_3$	$C_{24}H_{18}O_3$	
Sum formula	$C_{24}H_{18}O_3$	$C_{24}H_{18}O_3$	
Mr	354.38	354.38	
Dx, g cm^{-3}	1.356	1.356	
Z	8	8	
Mu (mm^{-1})	0.089	0.089	
F000	1488.0	1488.0	

F000’	1488.71	
h, k, lmax	29, 16, 18	28, 16, 18
Nref	4600	3970
Tmin, Tmax	0.979, 0.982	0.821, 1.000
Tmin’	0.965	
Correction method = # Reported T Limits: Tmin = 0.821 Tmax = 1.000 AbsCorr = MULTI-SCAN		

Data completeness	0.863	Theta (max)	28.958
R (reflections)	0.0530 (3099)	wR2 (reflections)	0.1351 (3970)
S	1.071	Npar	247

X-ray structure of 3ab’

CCDC 1944707

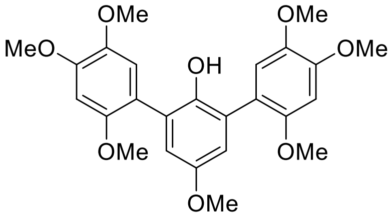

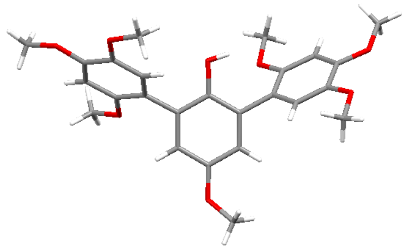

Table S3. Crystal data and details of data collection and refinement for compound **3ab’**

Bond precision	C–C = 0.0030 A	Wavelength	0.71073
Cell:	a = 29.9022 (18)	b = 11.0935 (6)	c = 13.4816 (7)
	alpha = 90	beta = 99.730 (5)	gamma = 90
Temperature	150 K		
	Calculated	Reported	
Volume	4407.8 (4)	4407.8 (4)	
Space group	C2/c	C12/c1	
Hall group	-C 2yc	-C 2yc	
Moiety formula	$C_{25}H_{28}O_8$	$C_{25}H_{28}O_8$	
Sum formula	$C_{25}H_{28}O_8$	$C_{25}H_{28}O_8$	
Mr	456.47	456.47	
Dx, g cm^{-3}	1.376	1.376	
Z	8	8	
Mu (mm^{-1})	0.103	0.103	
F000	1936.0	1936.0	
F000’	1937.13		

h, k, lmax	40, 15, 18		40, 15, 17
Nref	5941		5108
Tmin, Tmax	0.970, 0.980		0.461, 1.000
Tmin’	0.970		
Correction method = # Reported T Limits: Tmin = 0.461 Tmax = 1.000 AbsCorr = MULTI-SCAN			
Data completeness	0.860	Theta (max)	29.150
R (reflections)	0.0545 (3801)	wR2 (reflections)	0.1334 (5108)
S	1.042	Npar	338

X-ray structure of 6b

CCDC 1944708

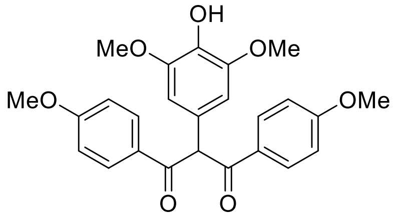

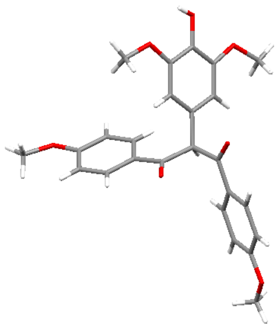

Table S4. Crystal data and details of data collection and refinement for compound **6b**

Bond precision	C–C = 0.0057 A	Wavelength	0.71073
Cell:	a = 12.5579 (10)	b = 5.2167 (4)	c = 35.021 (3)
	alpha = 90	beta = 94.917 (7)	gamma = 90
Temperature	150 K		
	Calculated	Reported	
Volume	2285.8 (3)	2285.8 (3)	
Space group	P 21/n	P1 21/n1	
Hall group	-P 2yn	-P 2yn	
Moiety formula	$C_{25}H_{24}O_7$ [+solvent]	$C_{25}H_{24}O_7$	
Sum formula	$C_{25}H_{24}O_7$ [+solvent]	$C_{25}H_{24}O_7$	
Mr	436.44	436.44	
Dx, g cm^{-3}	1.268	1.268	
Z	4	4	
Mu (mm^{-1})	0.093	0.093	
F000	920.0	920.0	
F000’	920.53		

h, k, lmax	17, 7, 47	17, 6, 47
Nref	6128	5341
Tmin, Tmax	0.967, 0.982	0.562, 1.000
Tmin’	0.963	

Correction method = # Reported T Limits: Tmin = 0.562 Tmax = 1.000 AbsCorr = MULTI-SCAN			
Data completeness	0.872	Theta (max)	29.096
R (reflections)	0.0968 (3169)	wR2 (reflections)	0.2306 (5341)
S	1.083	Npar	294

X-ray structure of 6g

CCDC 1944710

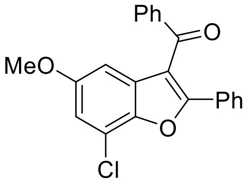

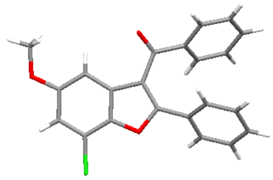

Table S5. Crystal data and details of data collection and refinement for compound **6g**

Bond precision	C–C = 0.0030 A	Wavelength	0.71073
Cell:	a = 16.2589 (10)	b = 6.7715 (3)	c = 16.4568 (9)
	alpha = 90	beta = 106.511 (6)	gamma = 90
Temperature	150 K		
	Calculated	Reported	
Volume	1737.14 (17)	1737.13 (17)	
Space group	P 21/n	P1 21/n1	
Hall group	-P 2yn	-P 2yn	
Moiety formula	$C_{22}H_{15}ClO_3$	$C_{22}H_{15}ClO_3$	
Sum formula	$C_{22}H_{15}ClO_3$	$C_{22}H_{15}ClO_3$	
Mr	362.79	362.79	
Dx, g cm^{-3}	1.387	1.387	
Z	4	4	
Mu (mm^{-1})	0.239	0.239	
F000	752.0	752.0	
F000’	752.93		
h, k, lmax	22, 9, 22	22, 9, 21	
Nref	4616	4055	
Tmin, Tmax	0.931, 0.953	0.682, 1.000	

Tmin’	0.931		
Correction method = # Reported T Limits: Tmin = 0.682 Tmax = 1.000 AbsCorr = MULTI-SCAN			
Data completeness	0.878	Theta (max)	28.988
R (reflections)	0.0521 (2977)	wR2 (reflections)	0.1222 (4055)
S	1.053	Npar	236

X-ray structure of 7d

CCDC 1944709

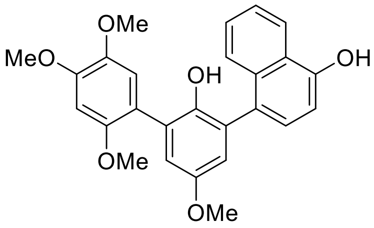

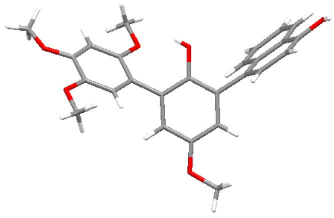

Table S6. Crystal data and details of data collection and refinement for compound **7d**

<table>
<tr><td>Bond precision</td><td>C–C = 0.0051 A</td><td>Wavelength</td><td colspan="2">0.71073</td></tr>
<tr><td rowspan="2">Cell:</td><td>a = 14.145 (2)</td><td>b = 7.441 (1)</td><td colspan="2">c = 24.560 (4)</td></tr>
<tr><td>alpha = 90</td><td>beta = 96.170 (13)</td><td colspan="2">gamma = 90</td></tr>
<tr><td>Temperature</td><td colspan="4">150 K</td></tr>
<tr><td></td><td colspan="2">Calculated</td><td colspan="2">Reported</td></tr>
<tr><td>Volume</td><td colspan="2">2570.0 (7)</td><td colspan="2">2570.0 (6)</td></tr>
<tr><td>Space group</td><td colspan="2">P 21/c</td><td colspan="2">P1 21/c1</td></tr>
<tr><td>Hall group</td><td colspan="2">-P 2ybc</td><td colspan="2">-P 2ybc</td></tr>
<tr><td>Moiety formula</td><td colspan="2">$C_{26}H_{24}O_6$ [+ solvent]</td><td colspan="2">$C_{26}H_{24}O_6$</td></tr>
<tr><td>Sum formula</td><td colspan="2">$C_{26}H_{24}O_6$ [+ solvent]</td><td colspan="2">$C_{26}H_{24}O_6$</td></tr>
<tr><td>Mr</td><td colspan="2">432.45</td><td colspan="2">432.45</td></tr>
<tr><td>Dx, g cm^{-3}</td><td colspan="2">1.118</td><td colspan="2">1.118</td></tr>
<tr><td>Z</td><td colspan="2">4</td><td colspan="2">4</td></tr>
<tr><td>Mu (mm^{-1})</td><td colspan="2">0.079</td><td colspan="2">0.079</td></tr>
<tr><td>F000</td><td colspan="2">912.0</td><td colspan="2">912.0</td></tr>
<tr><td>F000’</td><td colspan="2">912.50</td><td colspan="2"></td></tr>
<tr><td>h, k, lmax</td><td colspan="2">16, 8, 29</td><td colspan="2">16, 8, 29</td></tr>
<tr><td>Nref</td><td colspan="2">4521</td><td colspan="2">4517</td></tr>
</table>

Tmin, Tmax	0.981, 0.992		0.832, 1.000
Tmin’	0.977		
Correction method = # Reported T Limits: Tmin = 0.832 Tmax = 1.000 AbsCorr = MULTI-SCAN			
Data completeness	0.999	Theta (max)	24.998
R (reflections)	0.0709 (2218)	wR2 (reflections)	0.1760 (4517)
S	0.975	Npar	295

^{1}H and ^{13}C NMR Spectra of Compounds

3a ^{1}H NMR (400 MHz, $CDCl_3$)

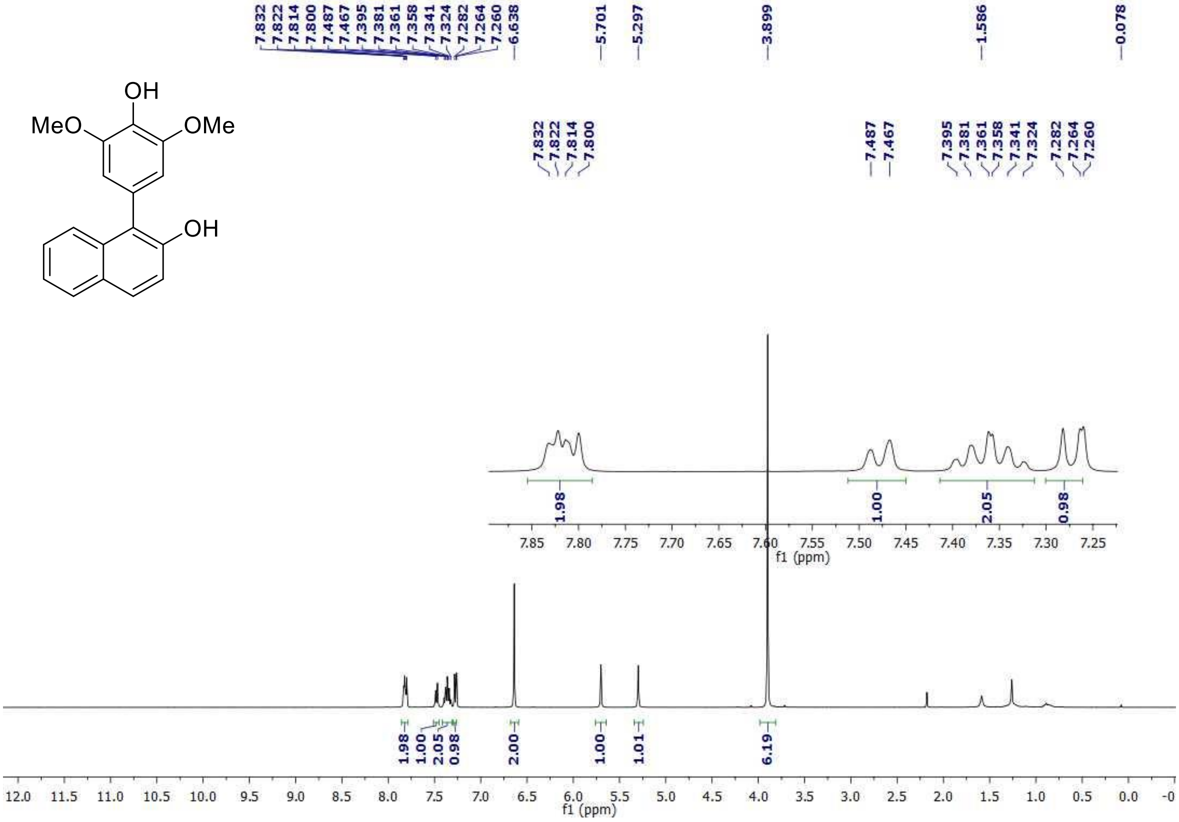

3a ^{13}C NMR (100 MHz, $CDCl_3$)

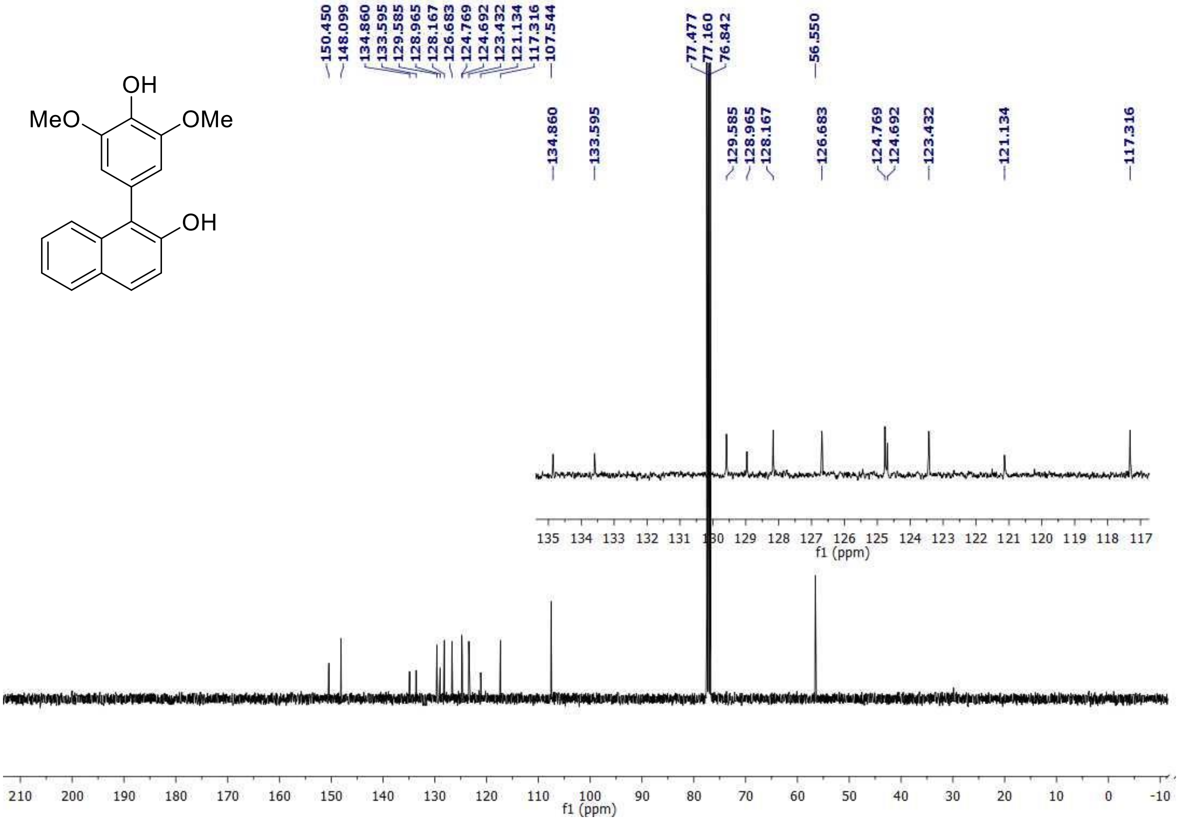

3b ^{1}H NMR (400 MHz, $CDCl_3$)

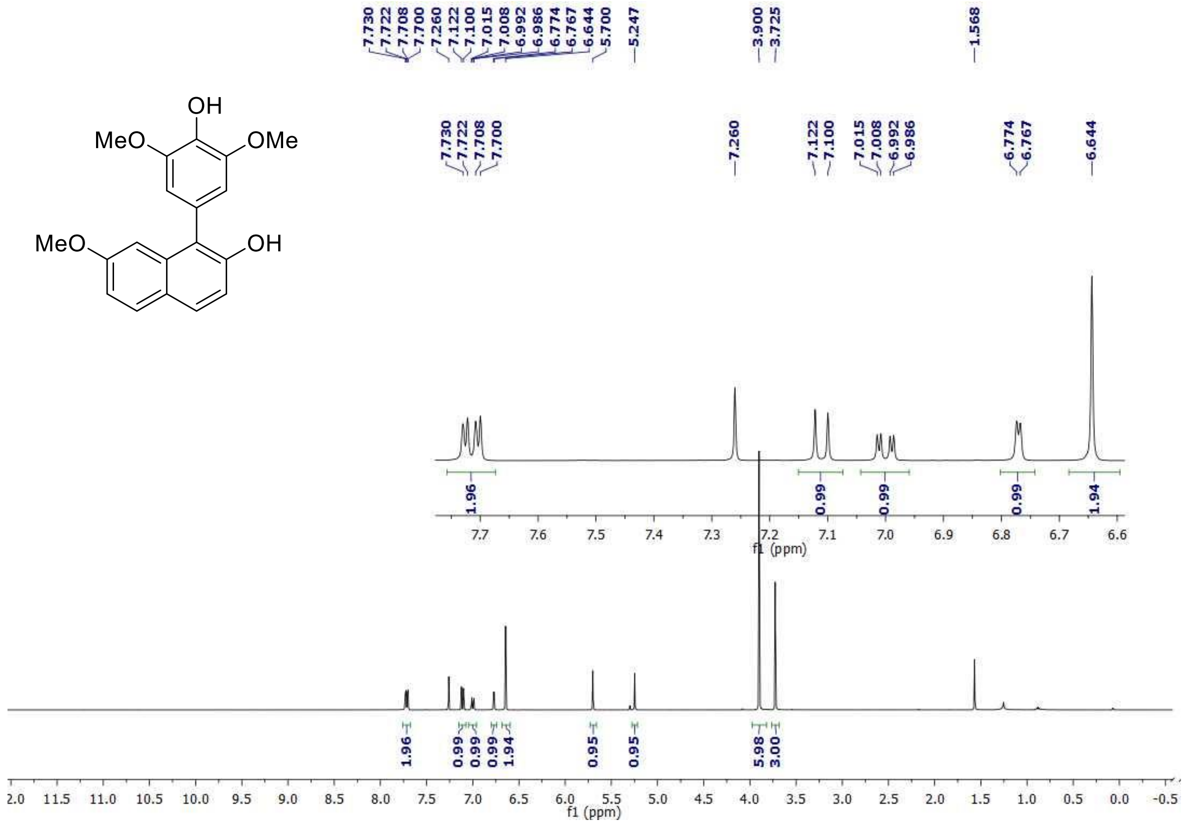

3b ^{13}C NMR (100 MHz, $CDCl_3$)

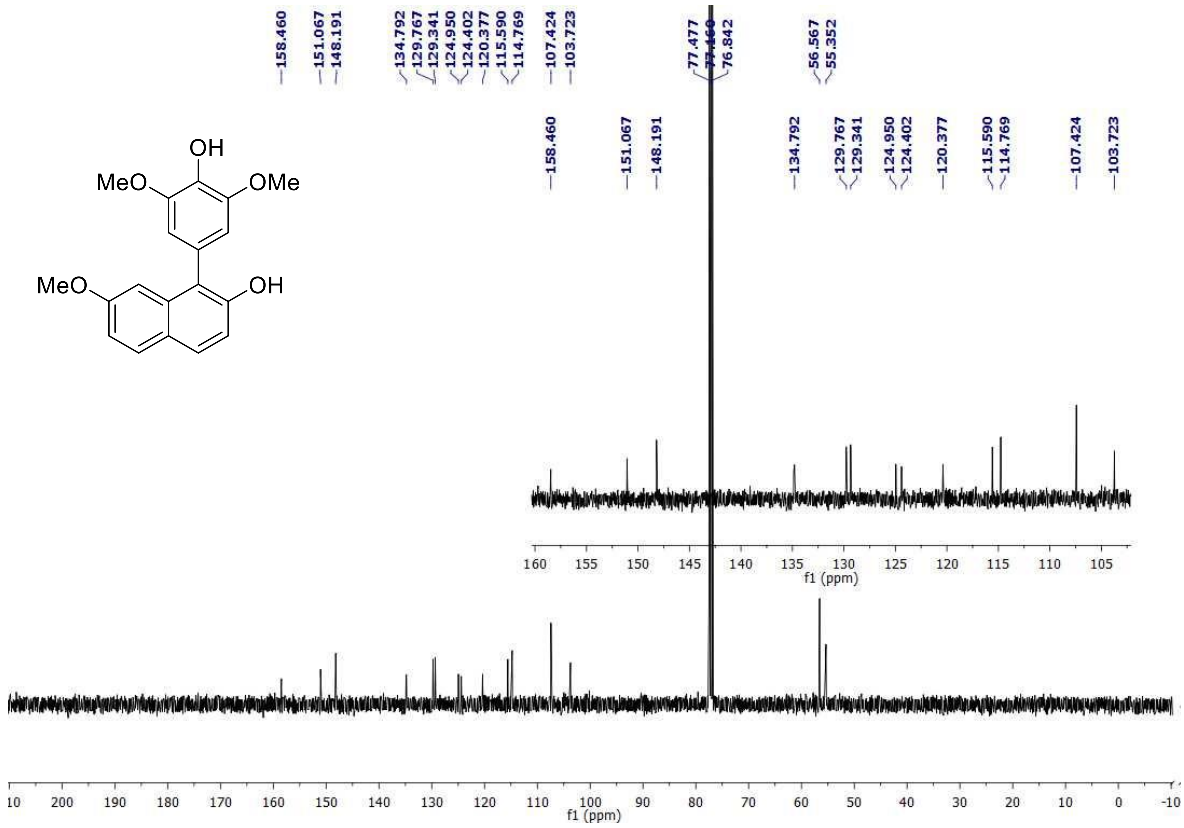

3c ^{1}H NMR (400 MHz, $CDCl_3$)

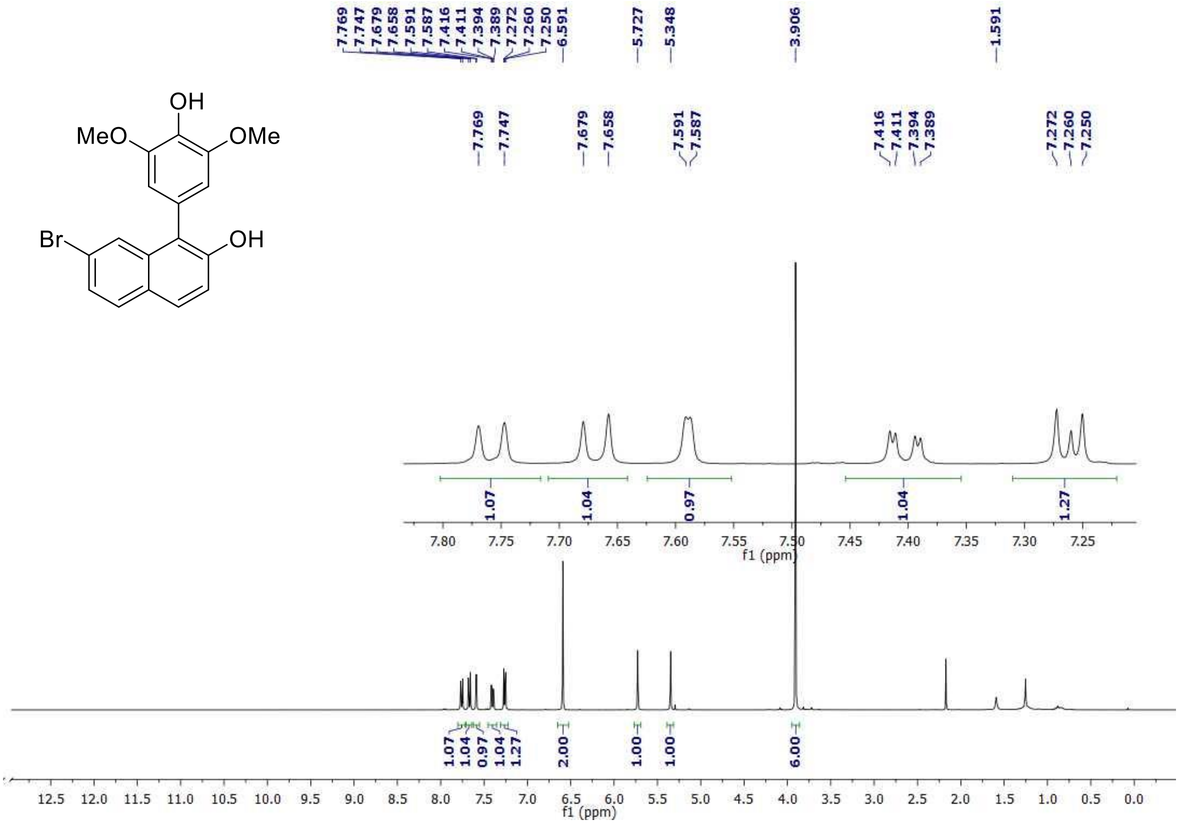

3c ^{13}C NMR (100 MHz, $CDCl_3$)

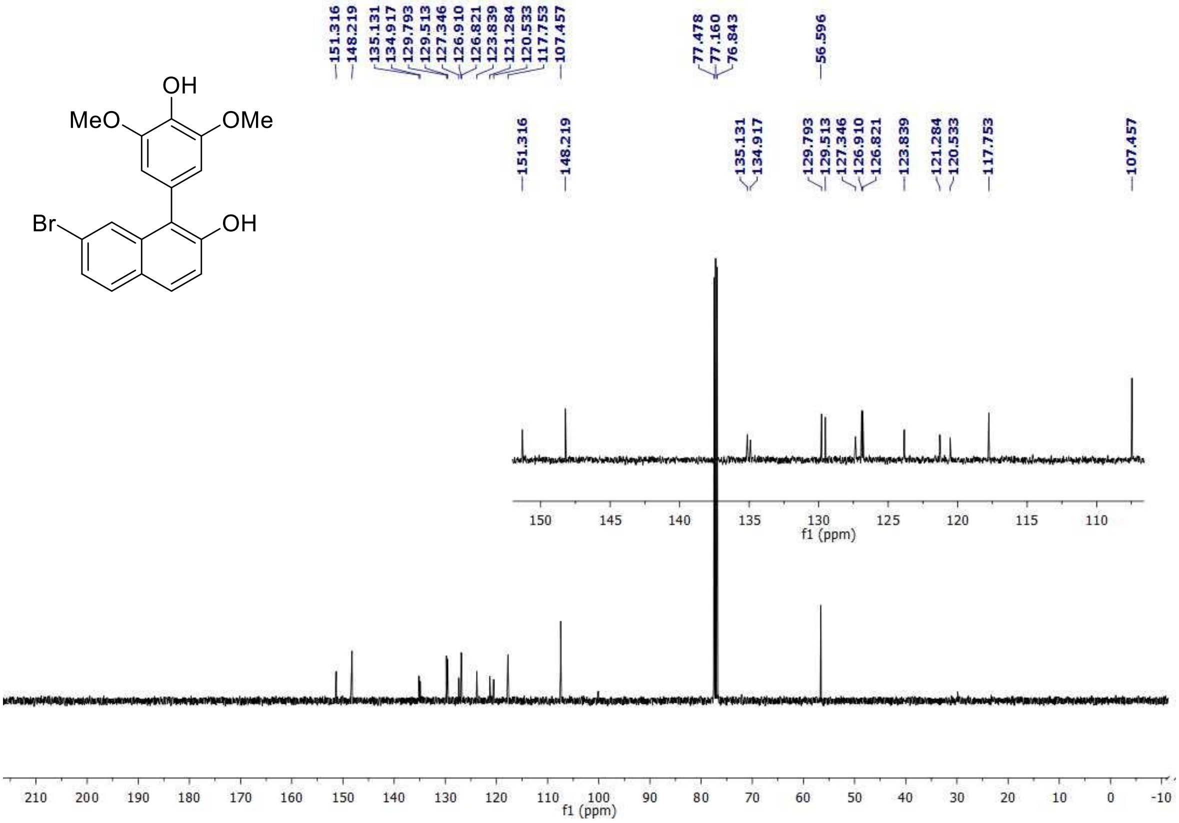

3d ^{1}H NMR (400 MHz, $CDCl_3$)

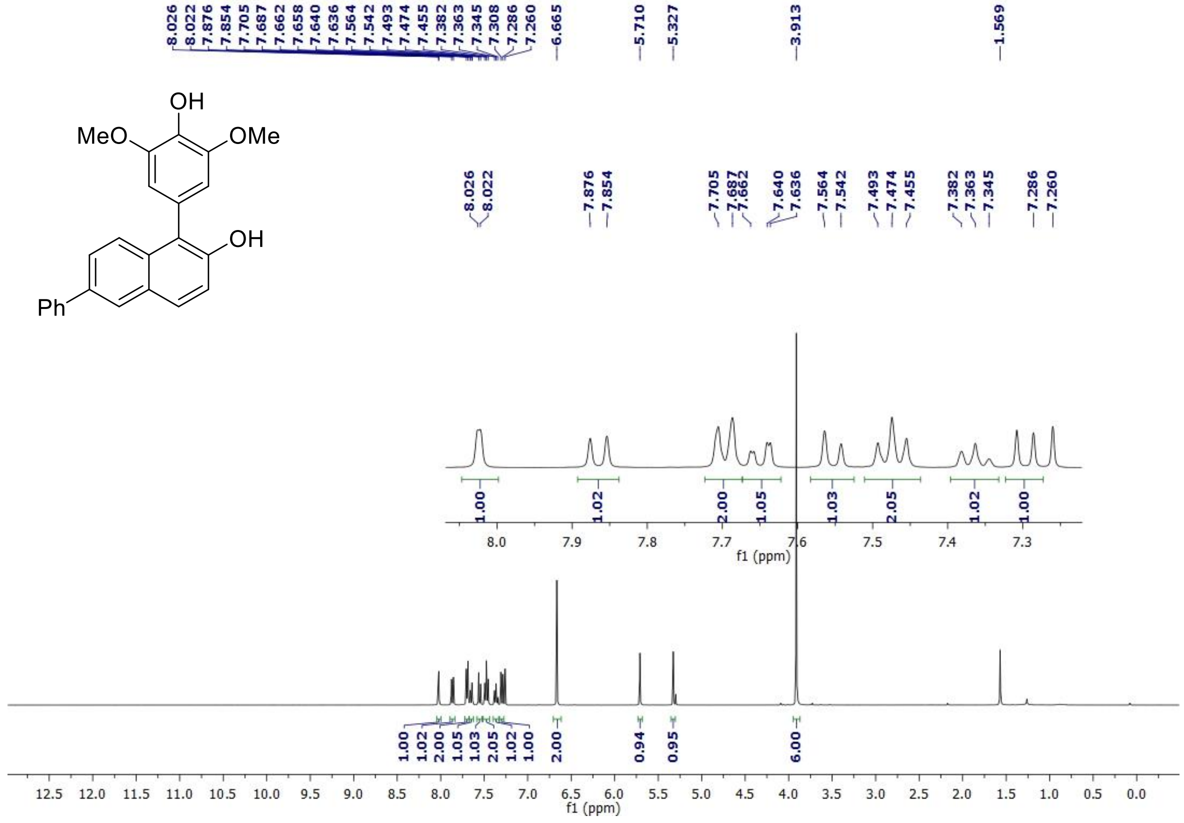

3d ^{13}C NMR (100 MHz, $CDCl_3$)

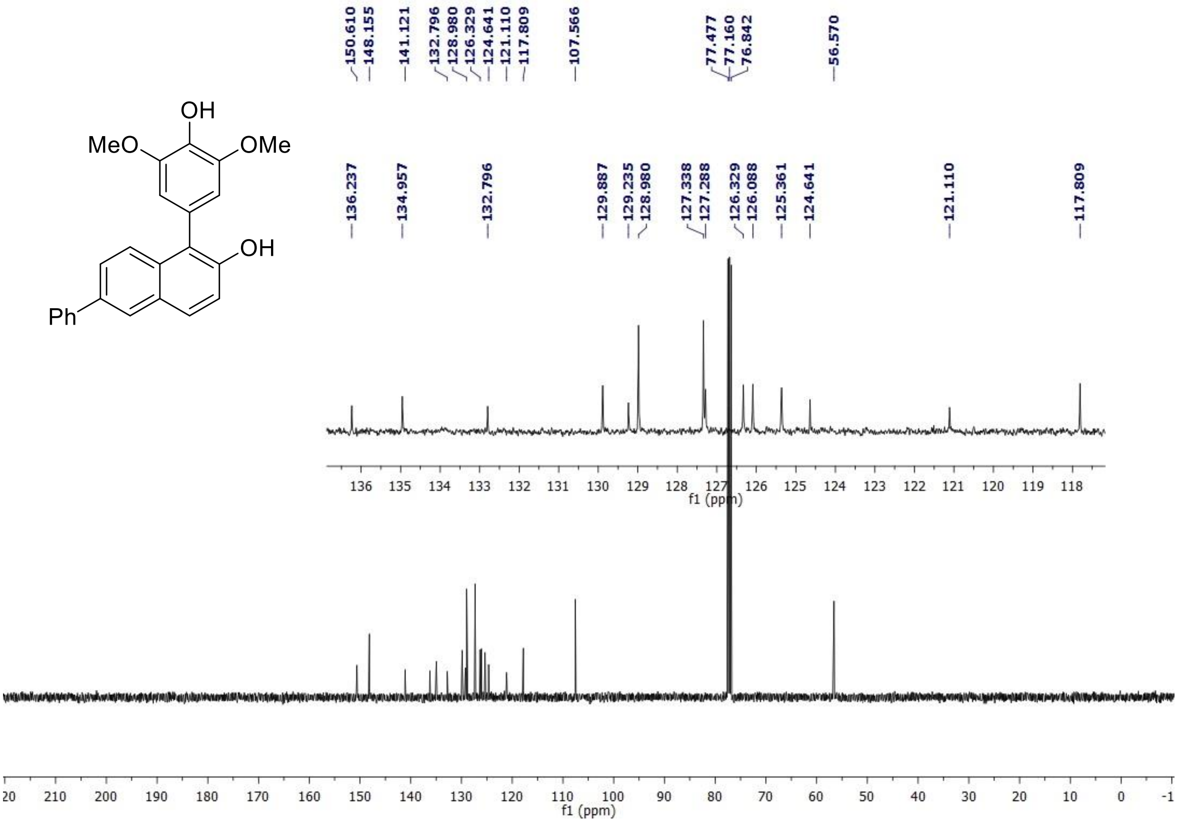

3e ^{1}H NMR (400 MHz, $CDCl_3$)

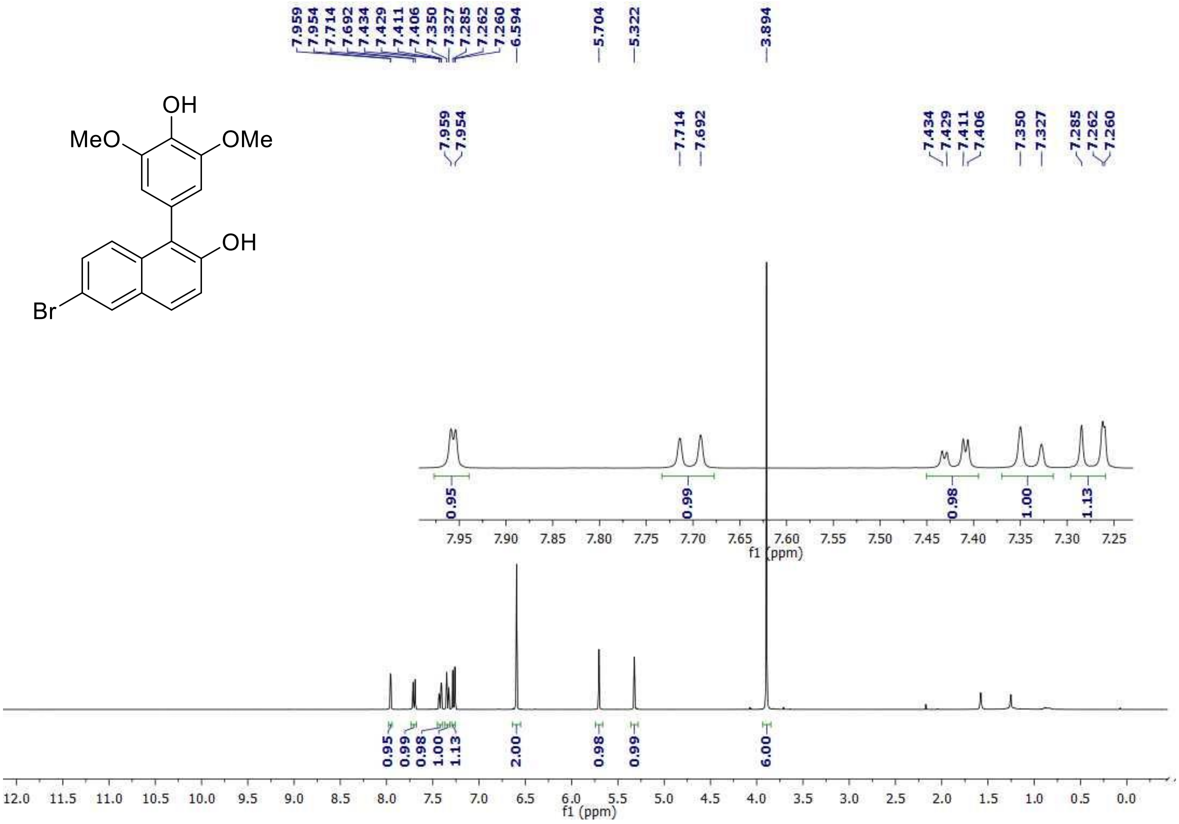

3e ^{13}C NMR (100 MHz, $CDCl_3$)

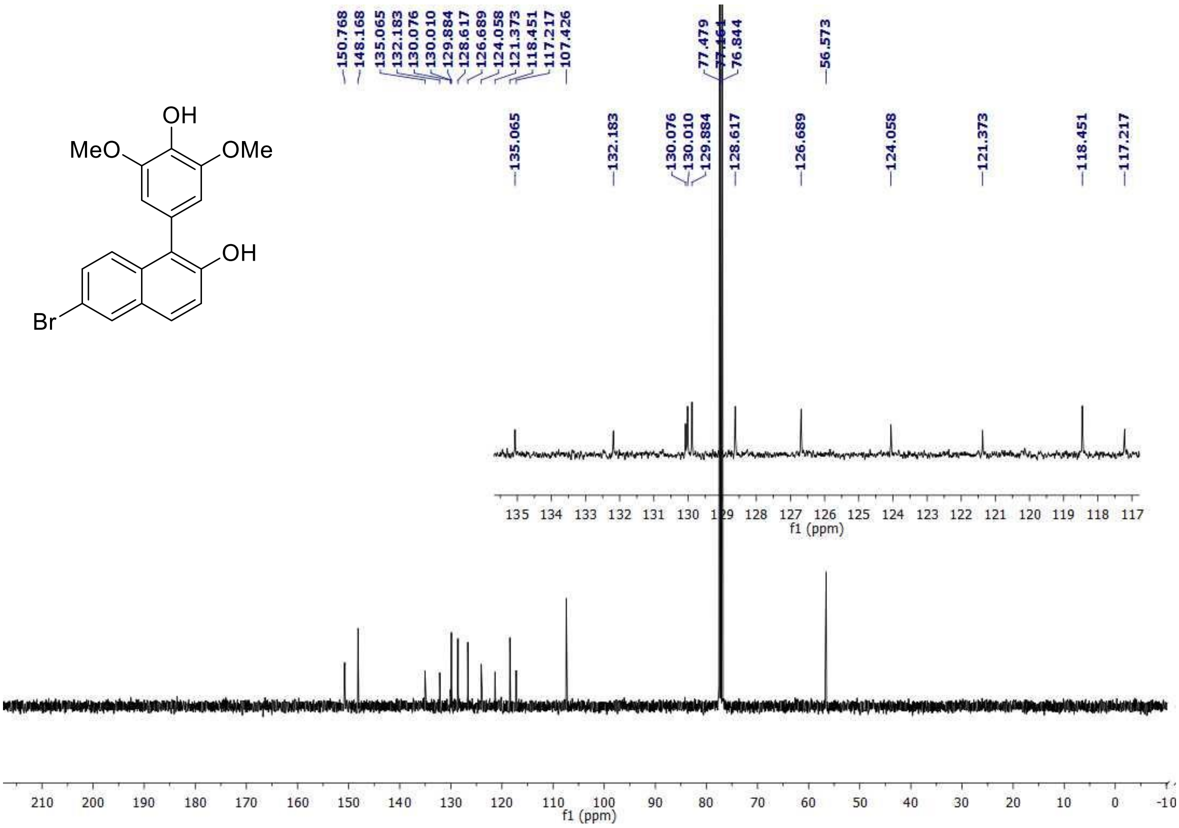

3f ^{1}H NMR (400 MHz, Acetone-d_6)

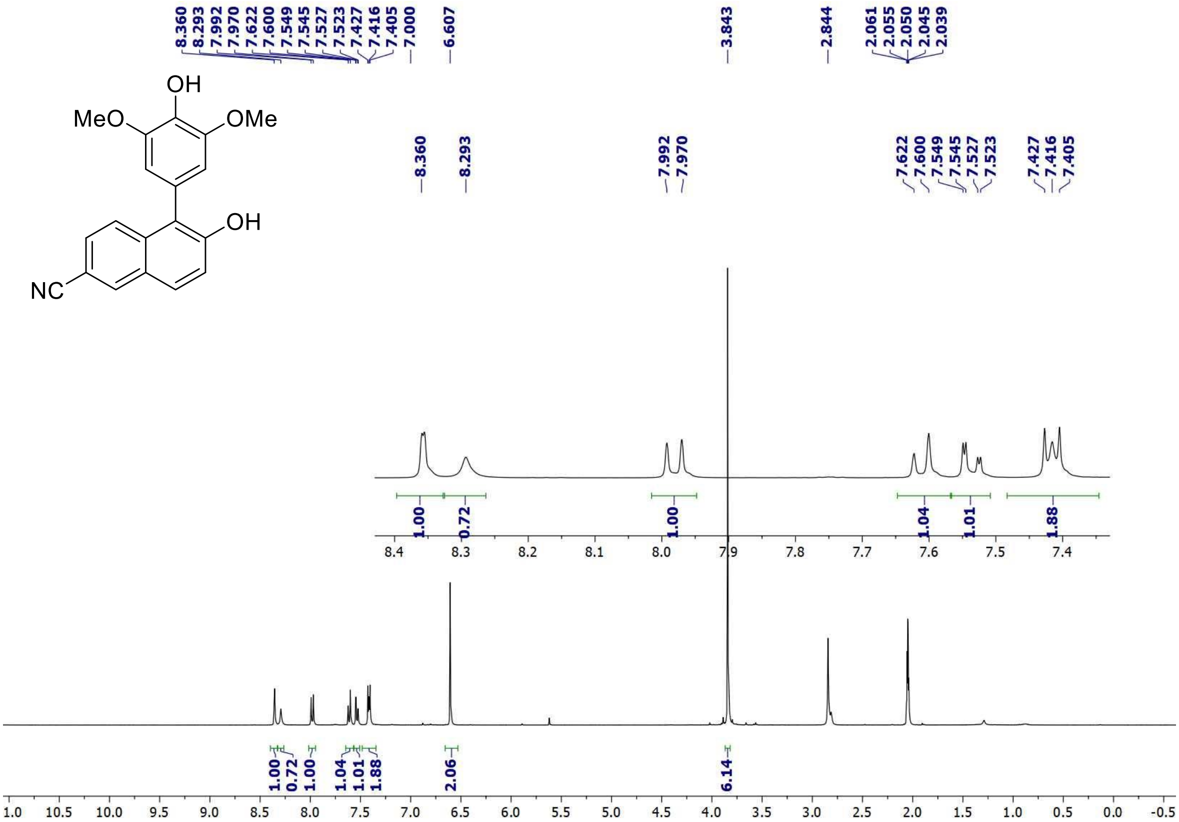

3f ^{13}C NMR (100 MHz, Acetone-d_6)

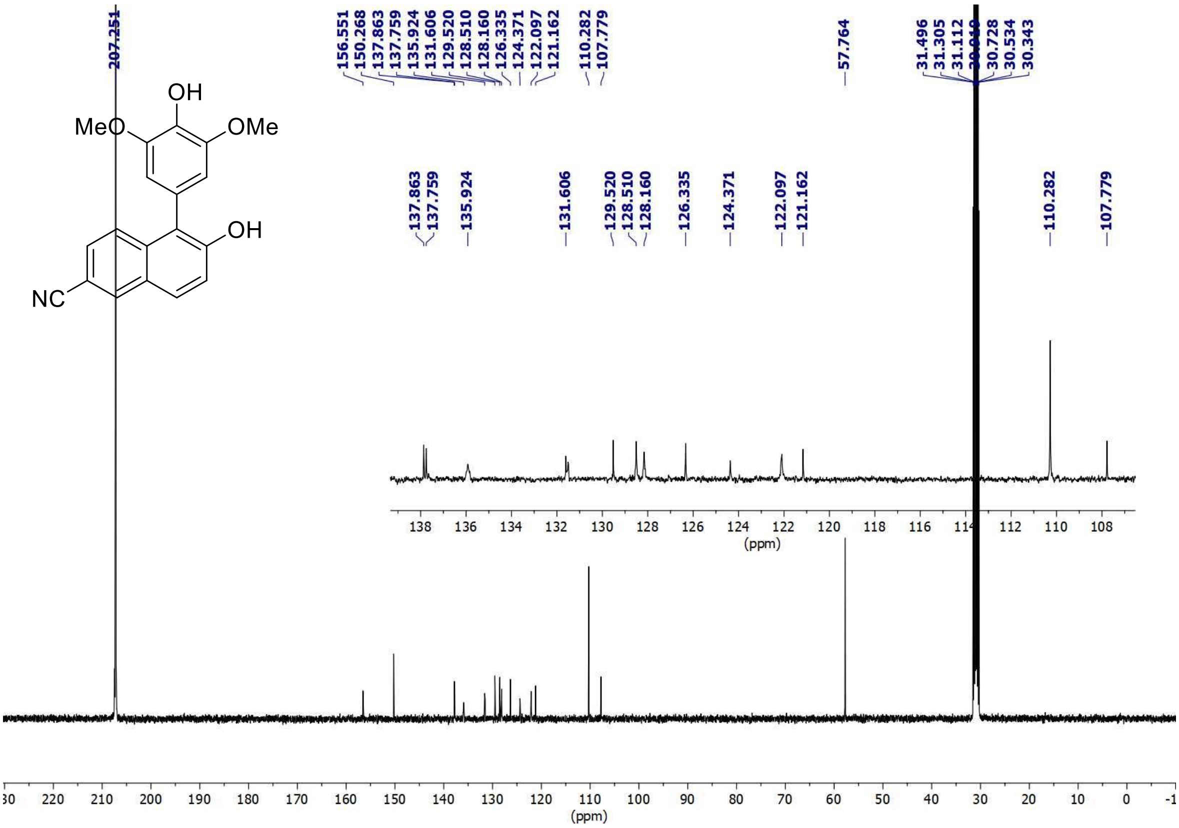

3g ^{1}H NMR (400 MHz, $CDCl_3$)

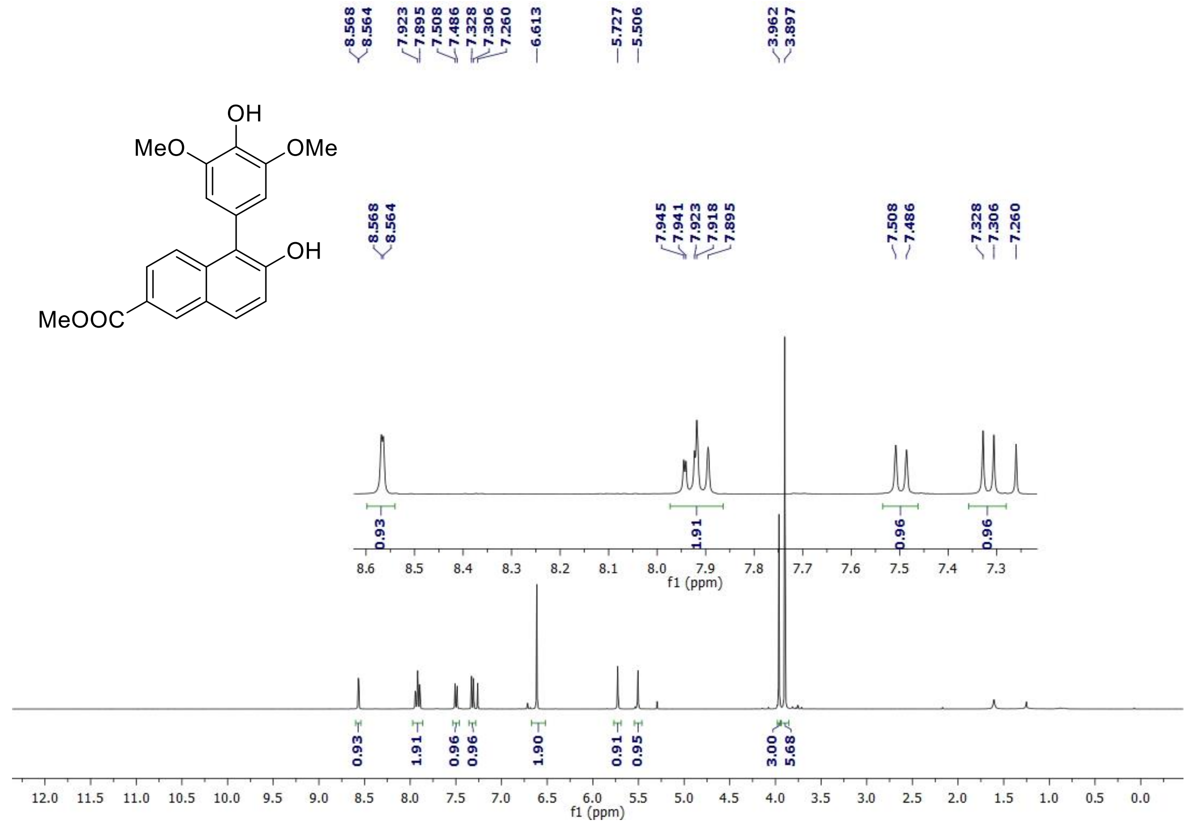

3g ^{13}C NMR (100 MHz, $CDCl_3$)

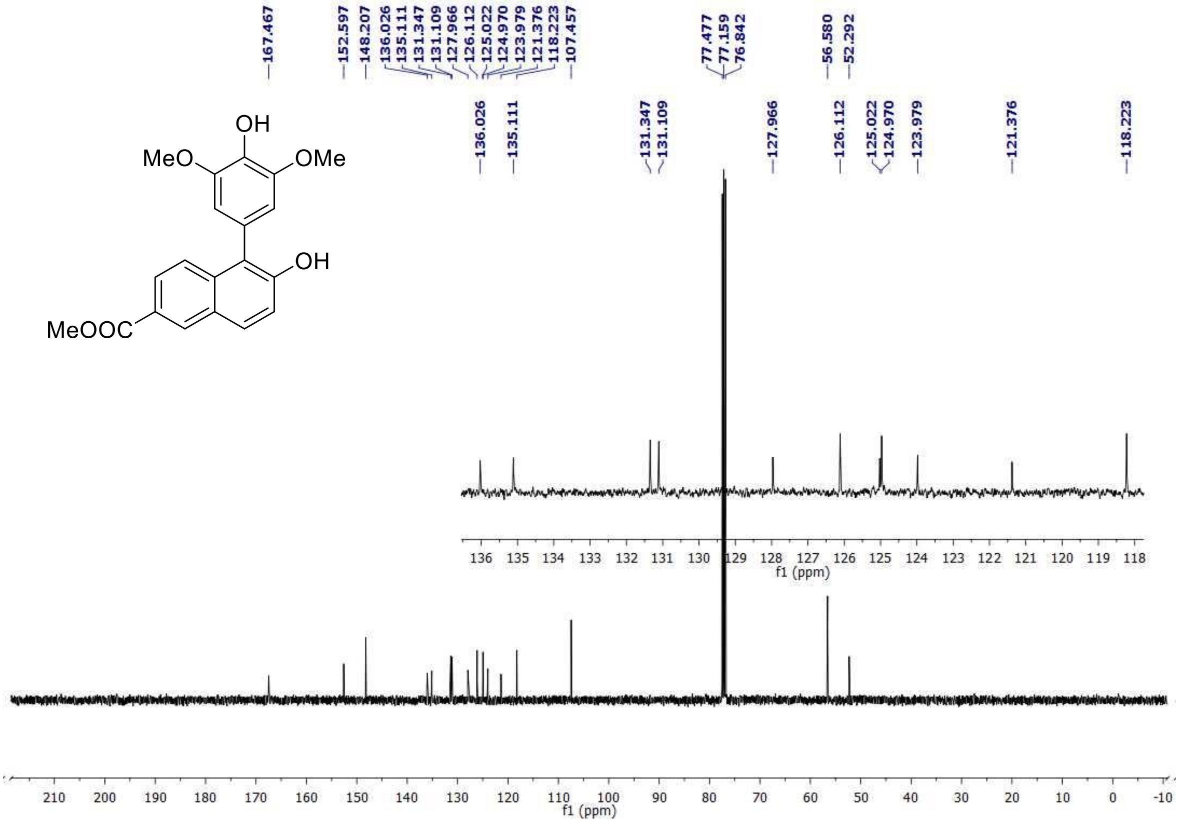

3h ^{1}H NMR (400 MHz, $CDCl_3$)

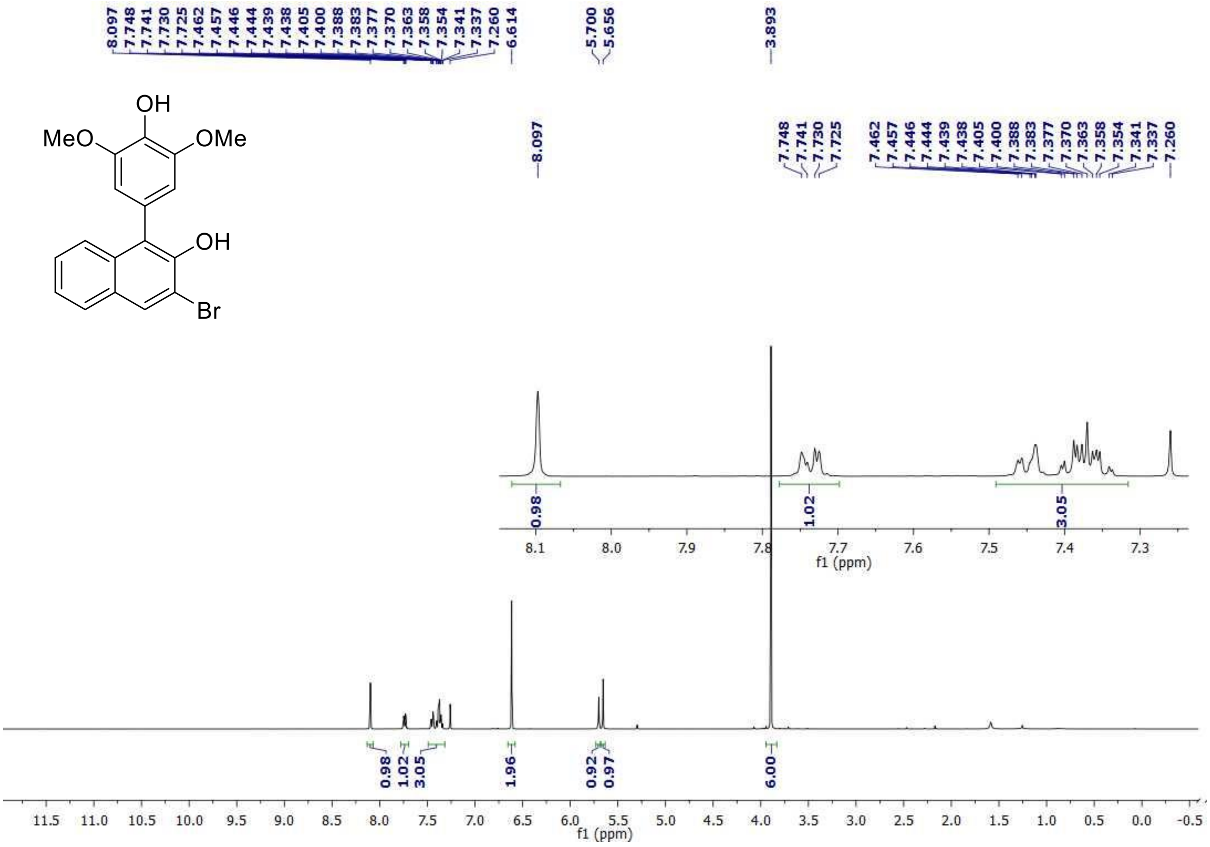

3h ^{13}C NMR (100 MHz, $CDCl_3$)

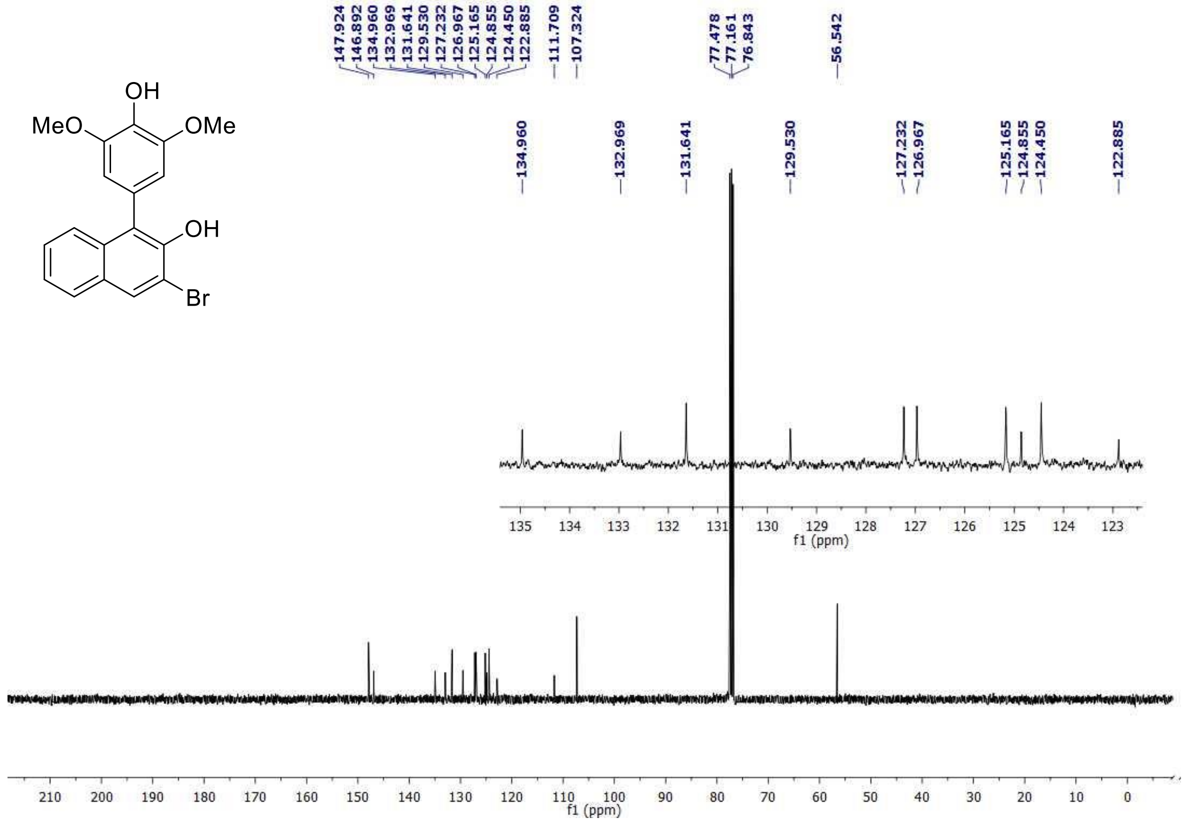

3i ^{1}H NMR (400 MHz, $CDCl_3$)

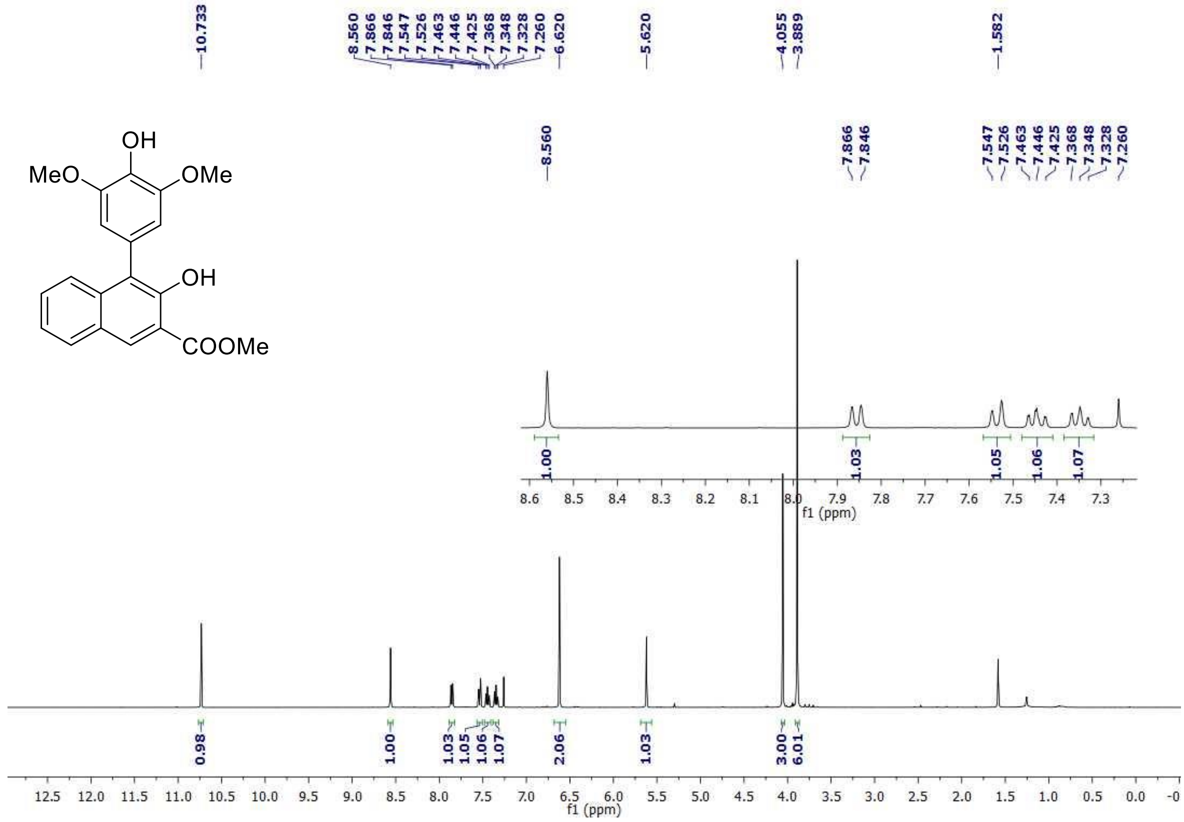

3i ^{13}C NMR (100 MHz, $CDCl_3$)

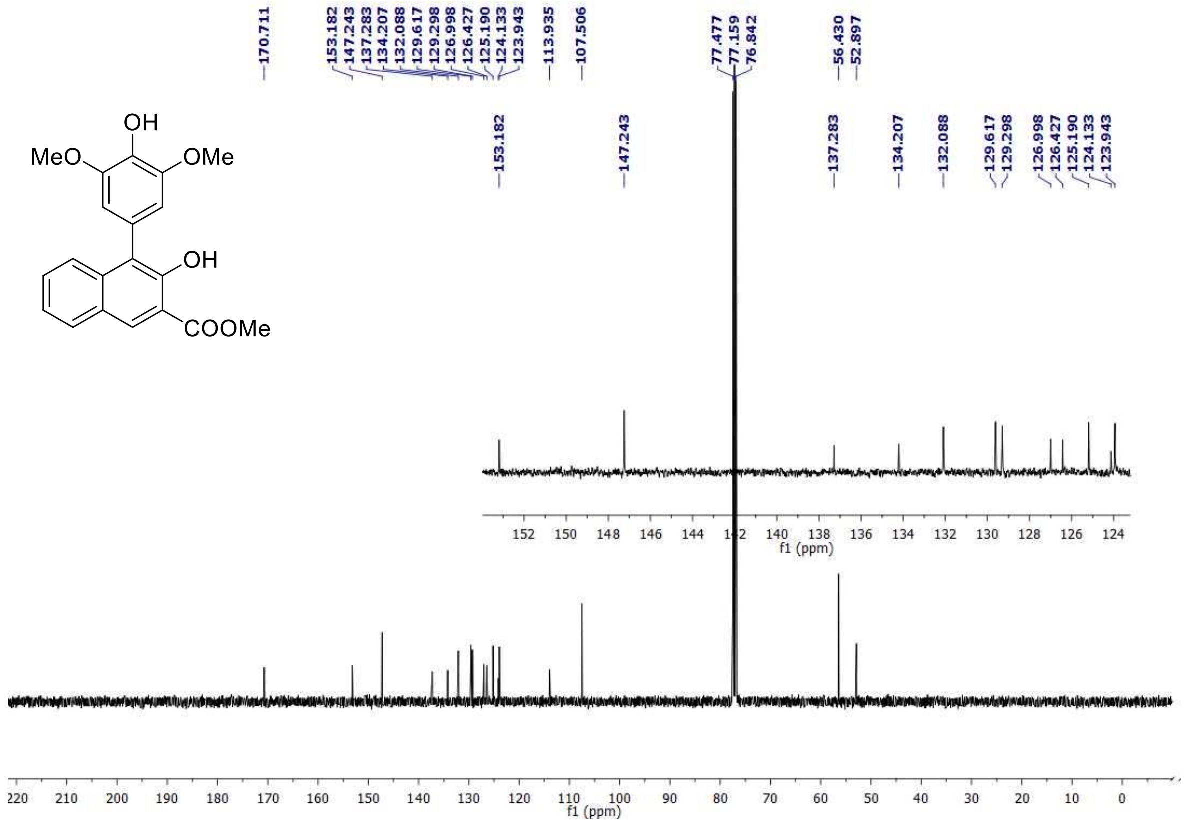

3j ^{1}H NMR (400 MHz, $CDCl_3$)

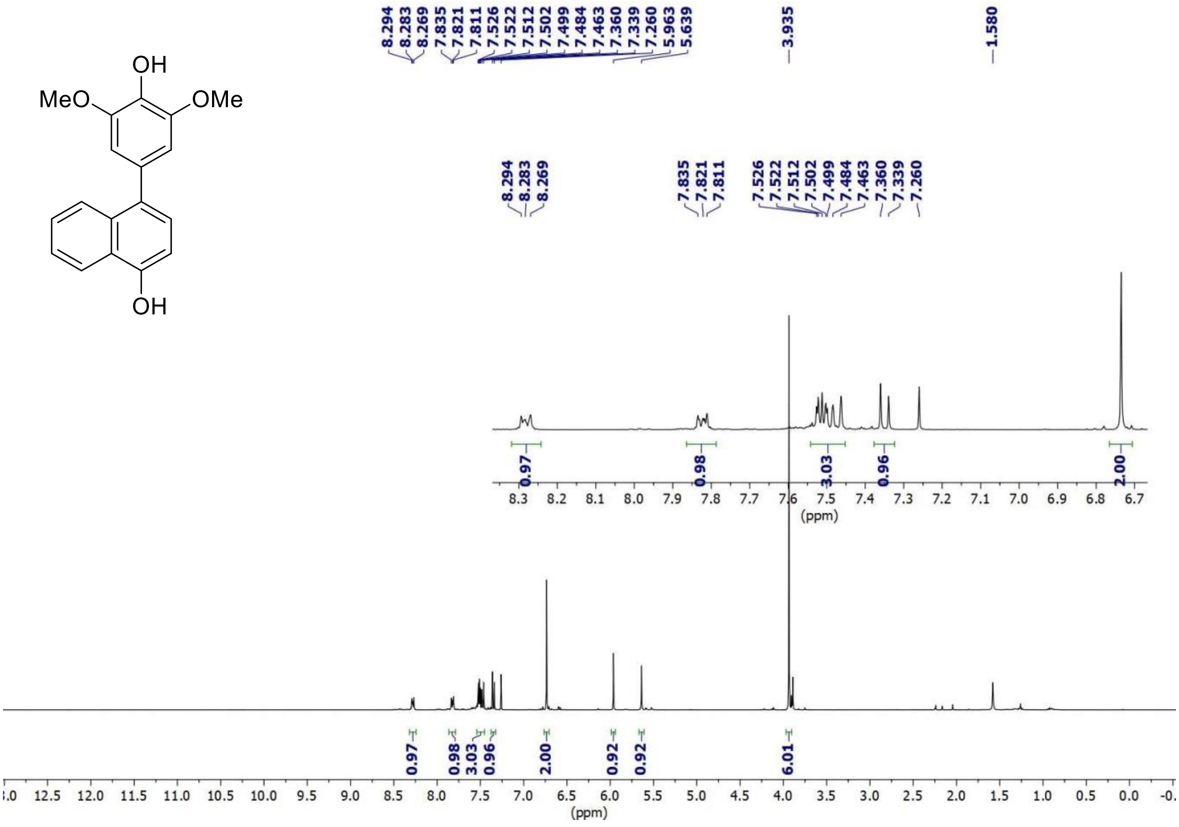

3j ^{13}C NMR (100 MHz, $CDCl_3$)

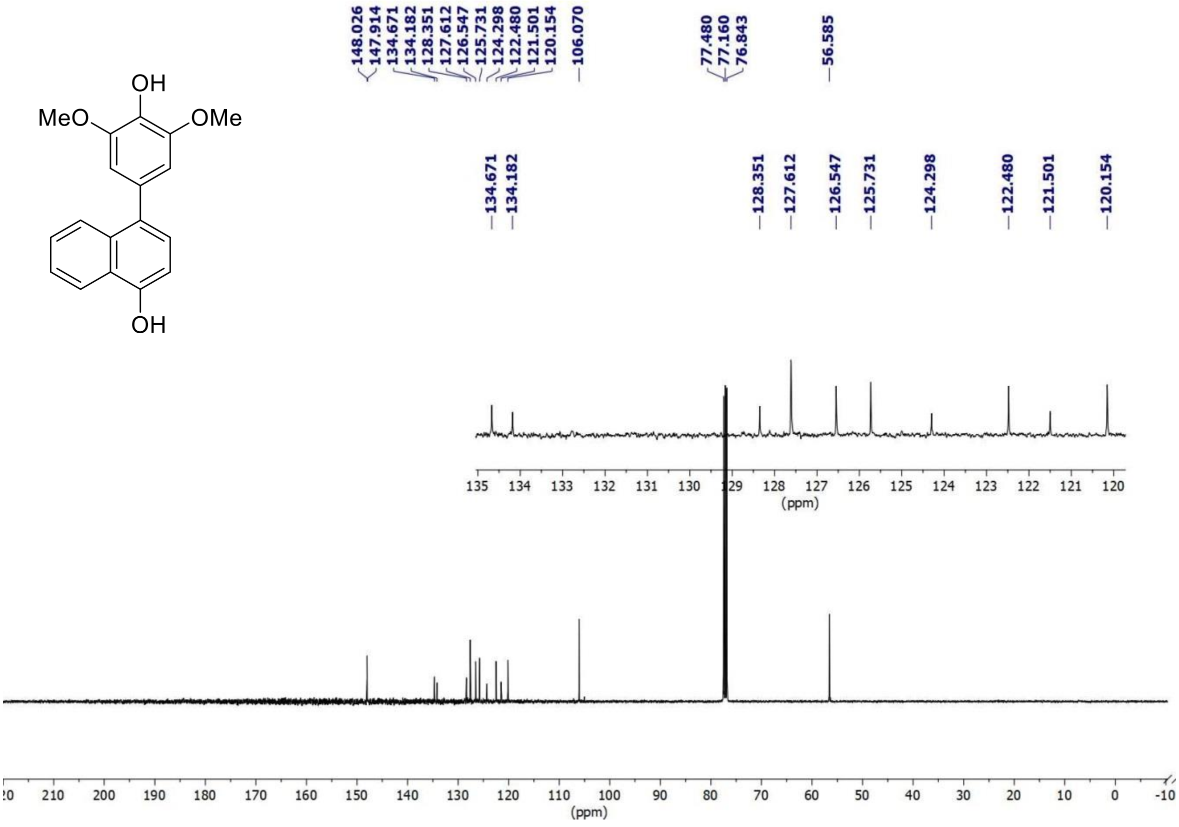

3k ^{1}H NMR (400 MHz, $CDCl_3$)

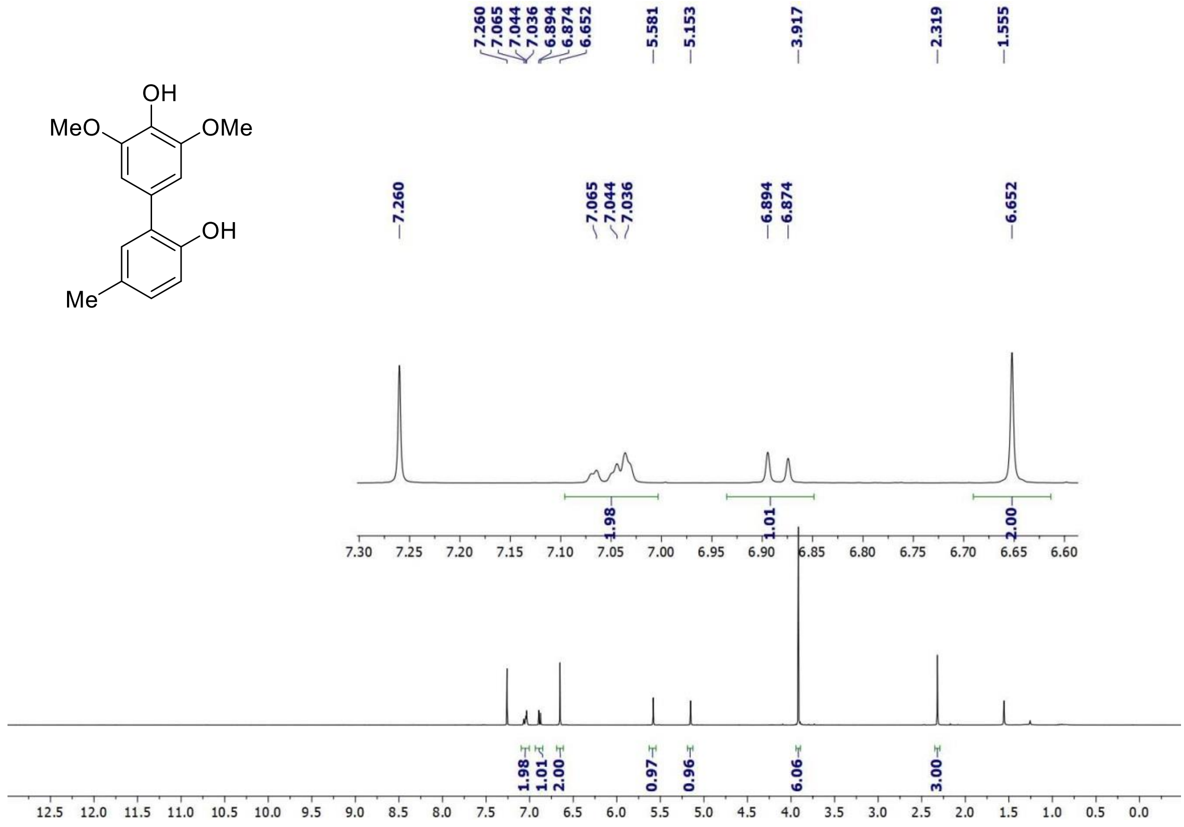

3k ^{13}C NMR (125 MHz, $CDCl_3$)

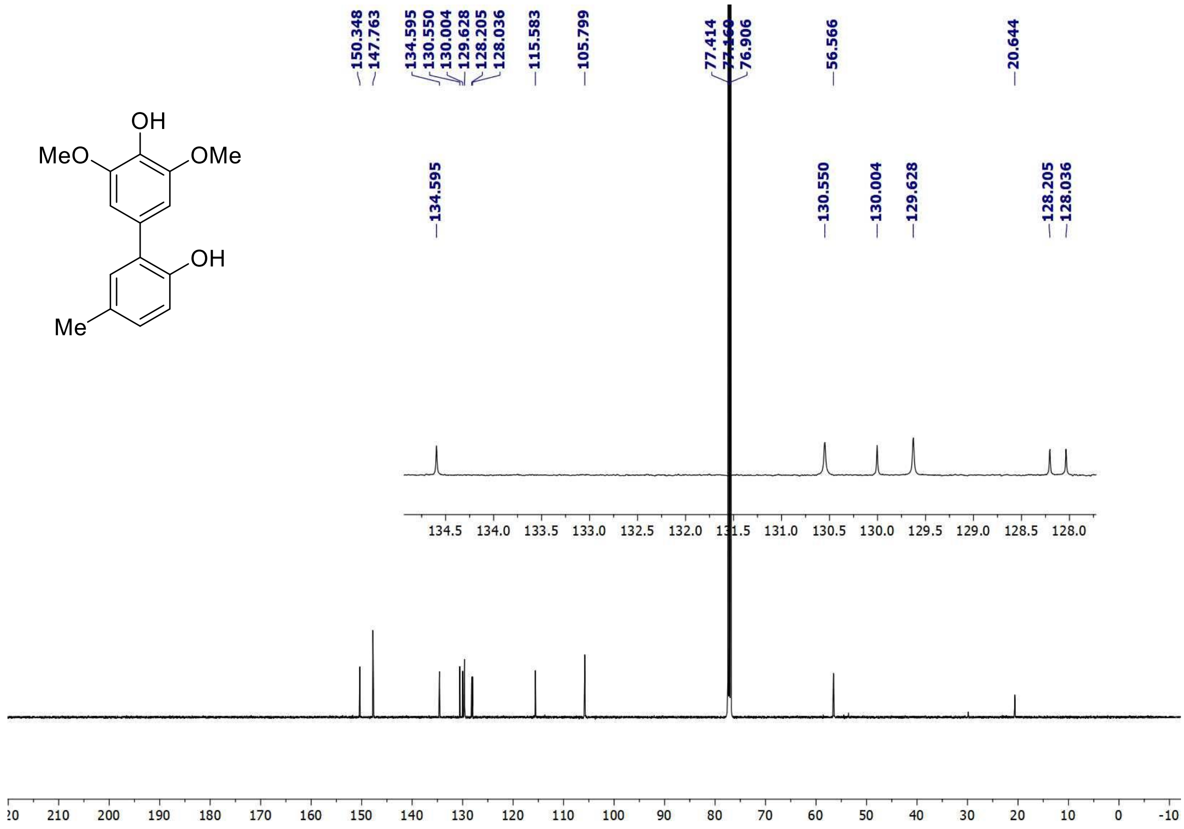

3l ^{1}H NMR (400 MHz, $CDCl_3$)

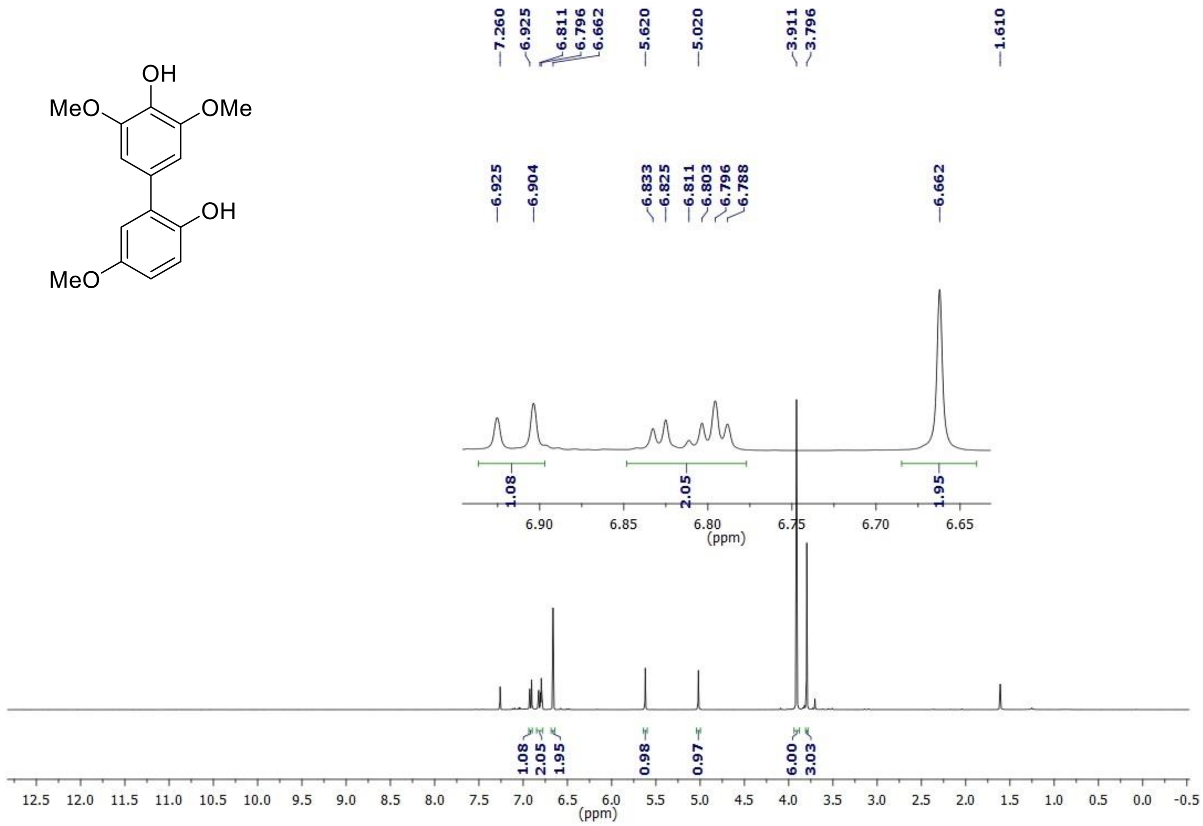

3l ^{13}C NMR (100 MHz, $CDCl_3$)

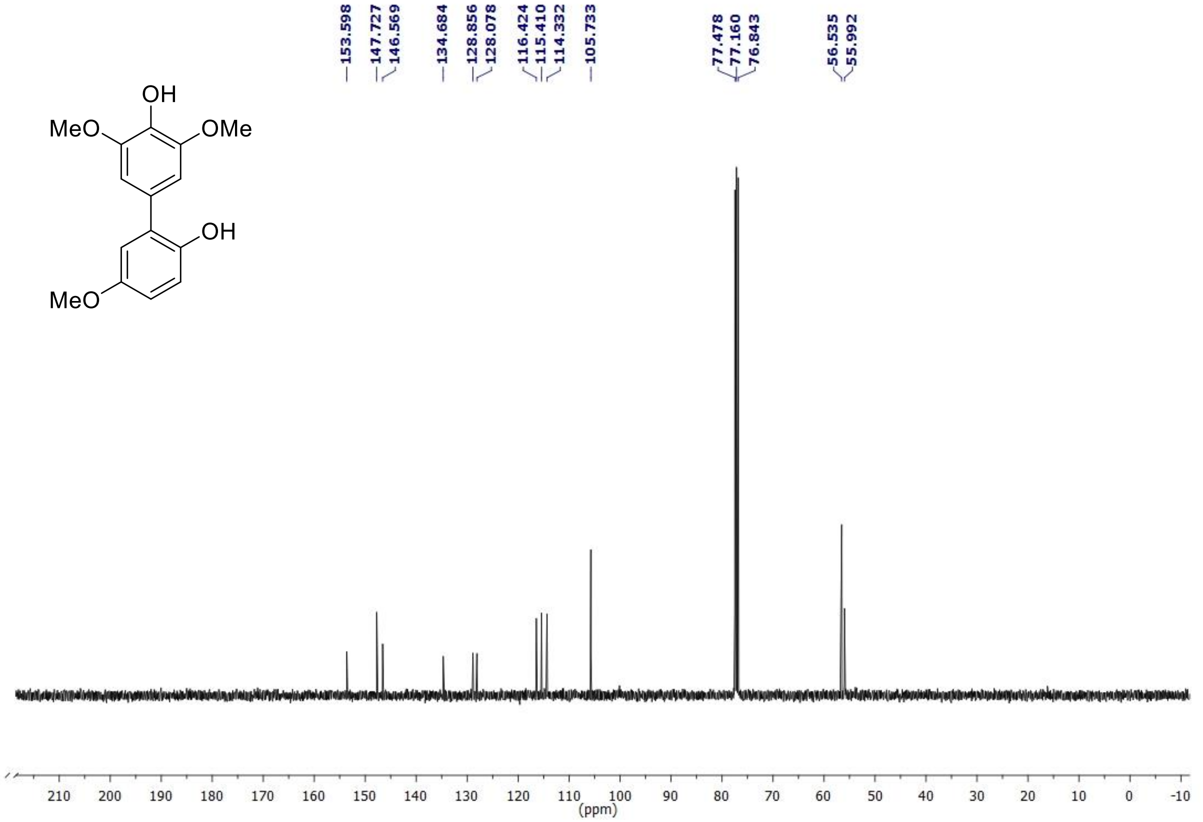

3m ^{1}H NMR (400 MHz, $CDCl_3$)

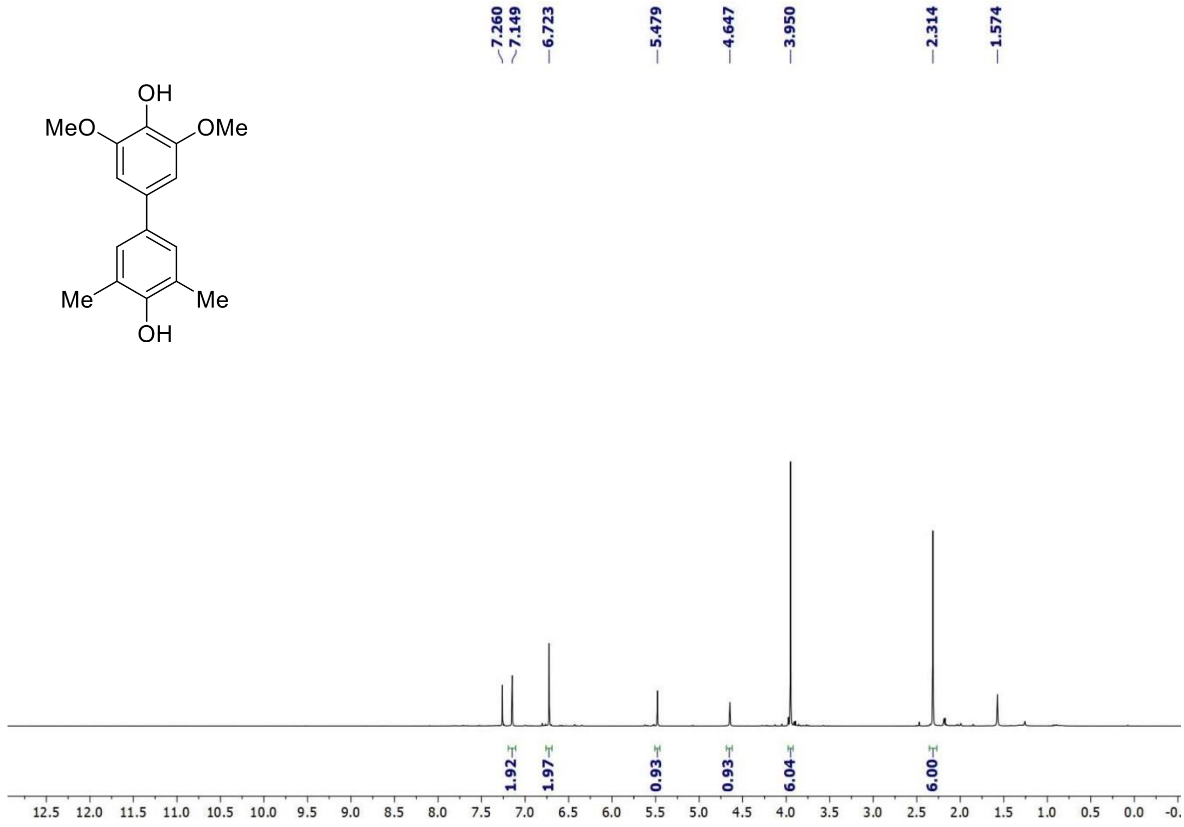

3m ^{13}C NMR (100 MHz, $CDCl_3$)

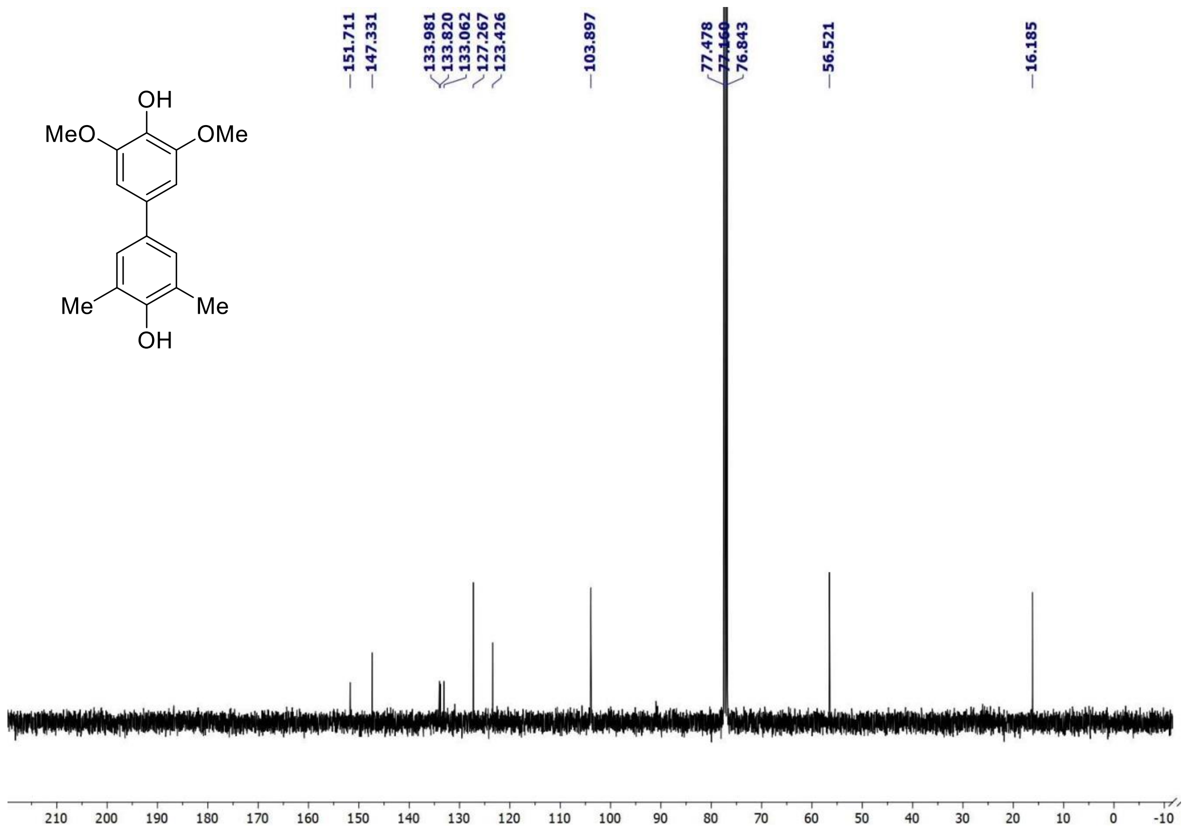

3n ^{1}H NMR (400 MHz, $CDCl_3$)

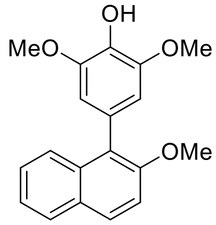

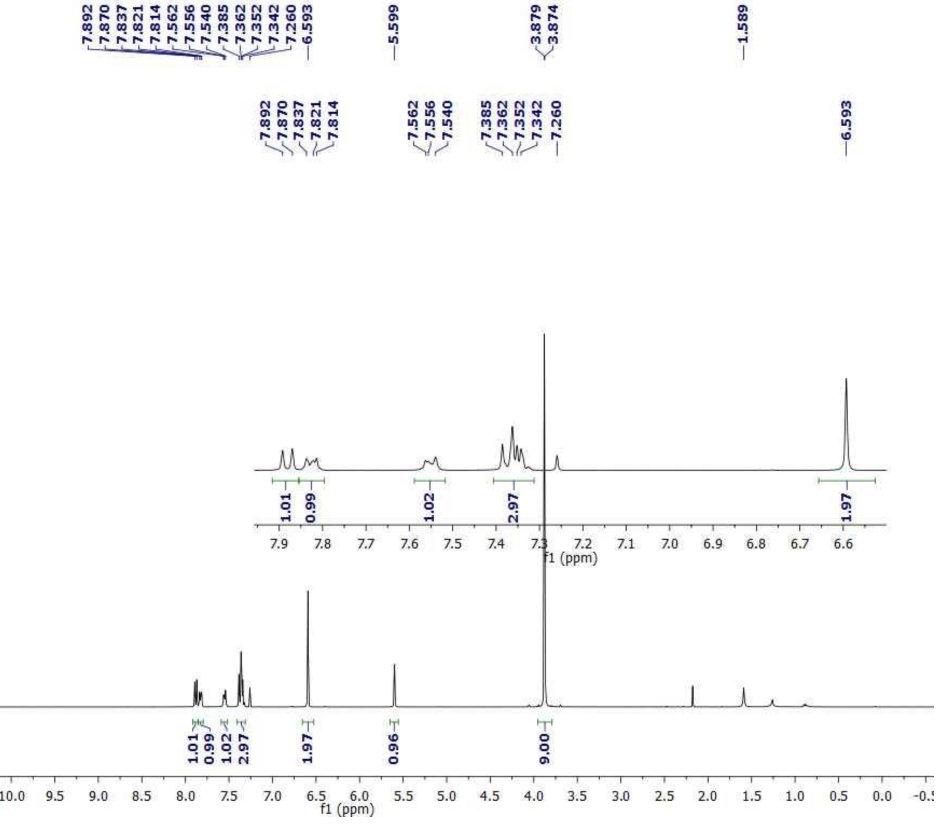

3n ^{13}C NMR (100 MHz, $CDCl_3$)

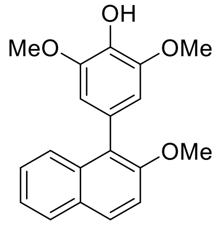

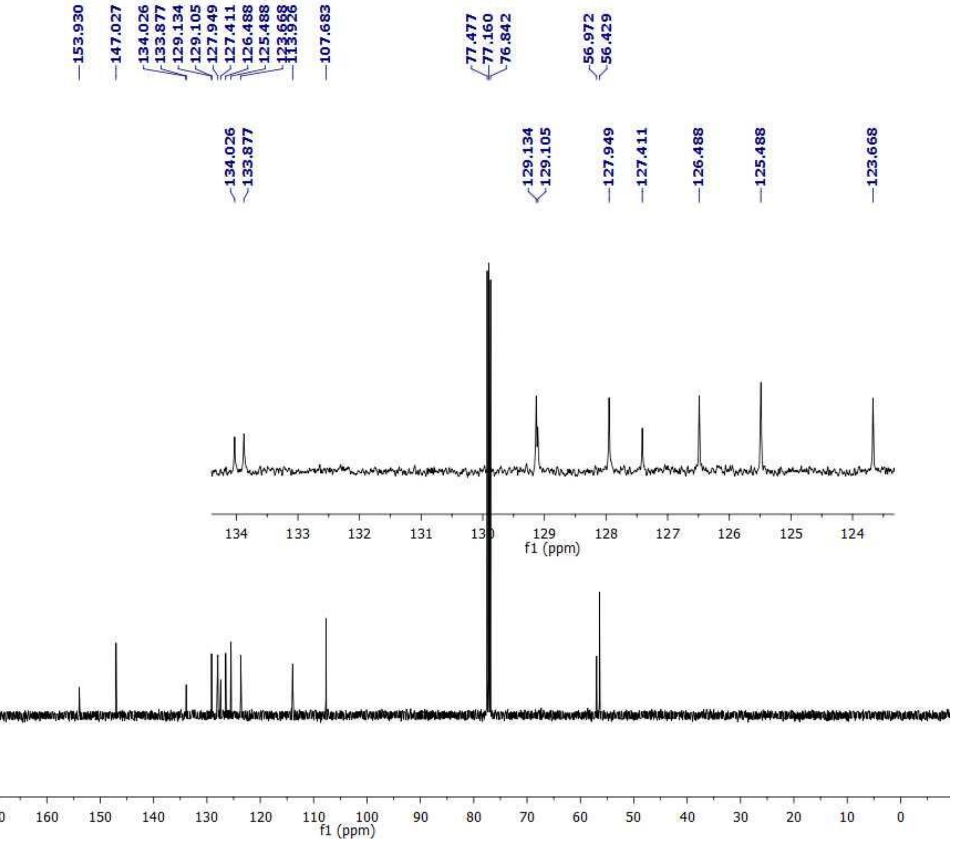

3o ^{1}H NMR (400 MHz, $CDCl_3$)

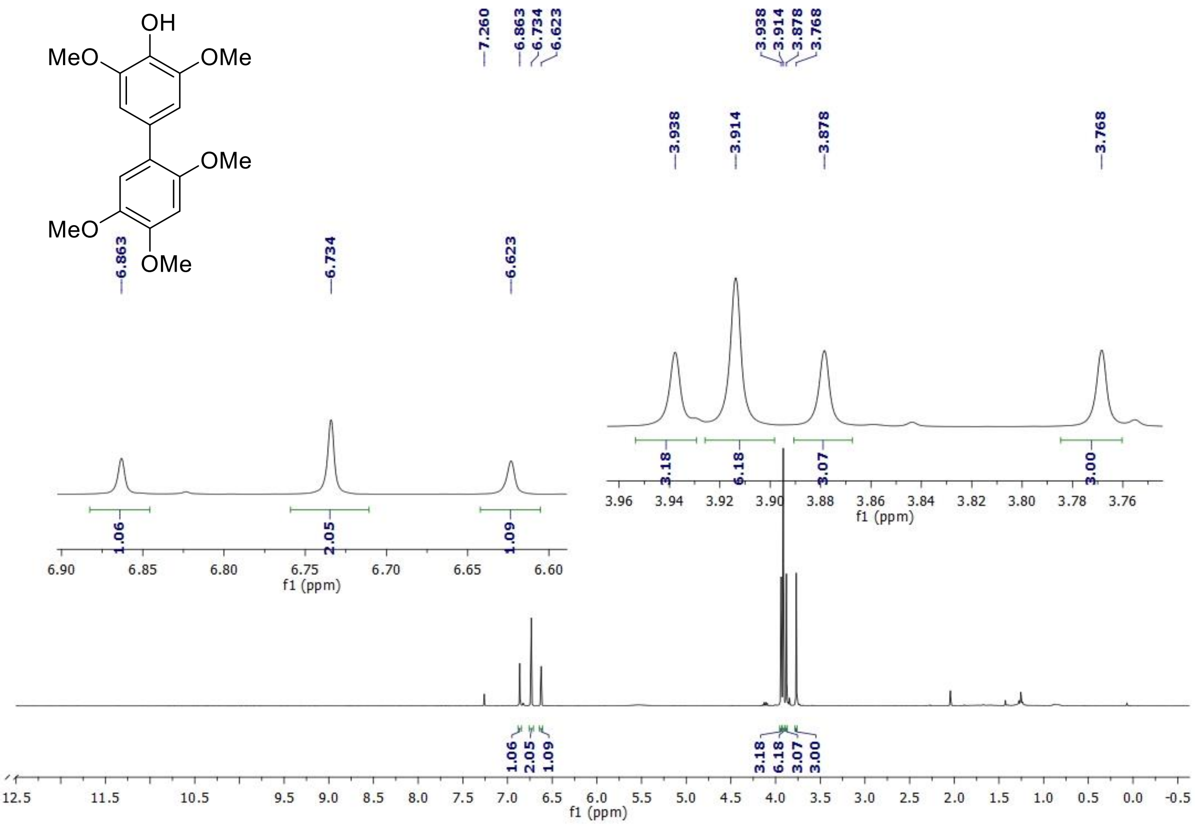

3o ^{13}C NMR (100 MHz, $CDCl_3$)

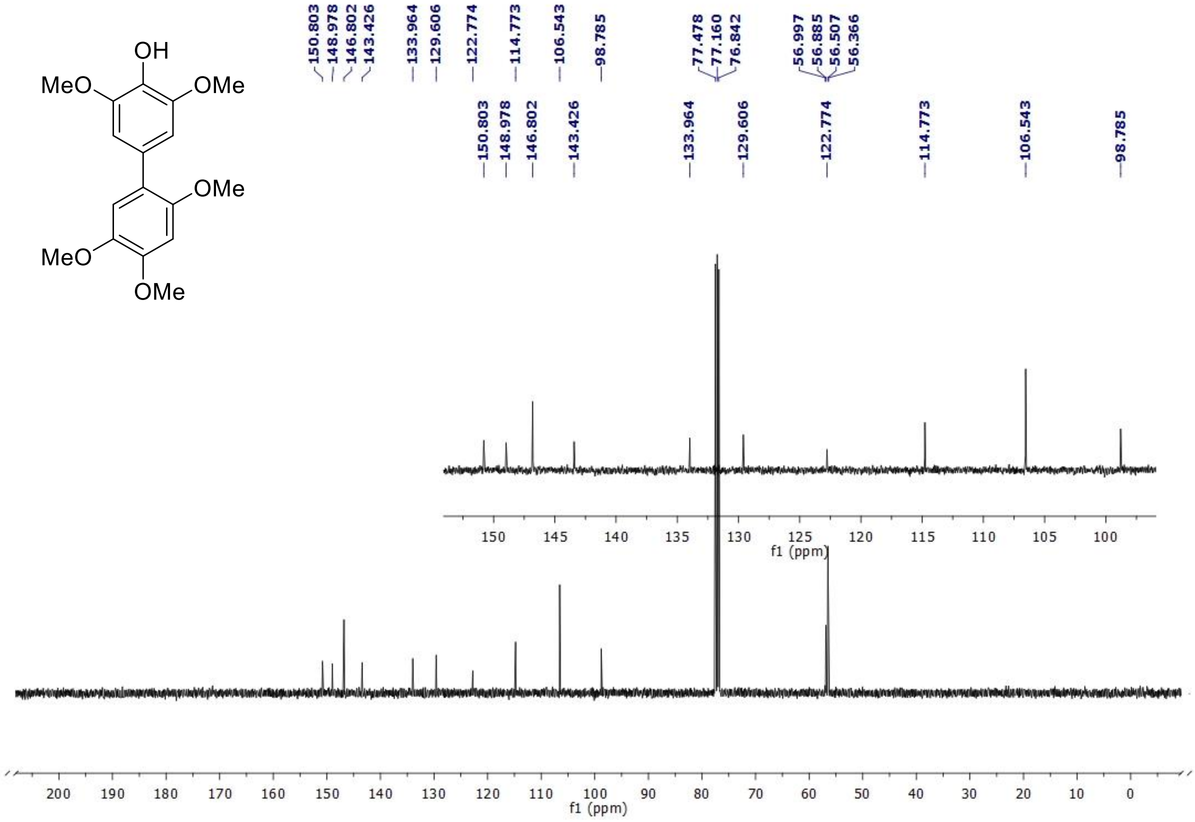

3p ^{1}H NMR (400 MHz, $CDCl_3$)

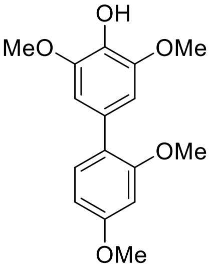

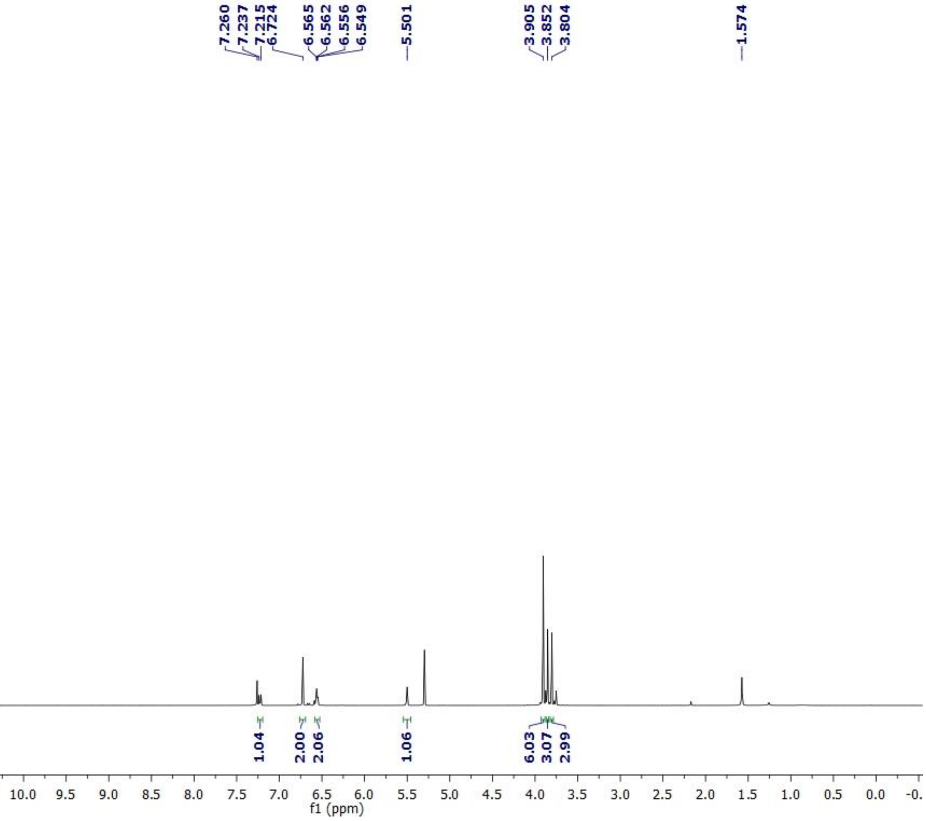

3p ^{13}C NMR (100 MHz, $CDCl_3$)

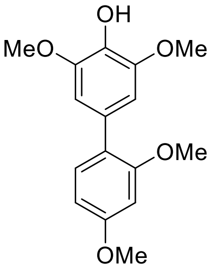

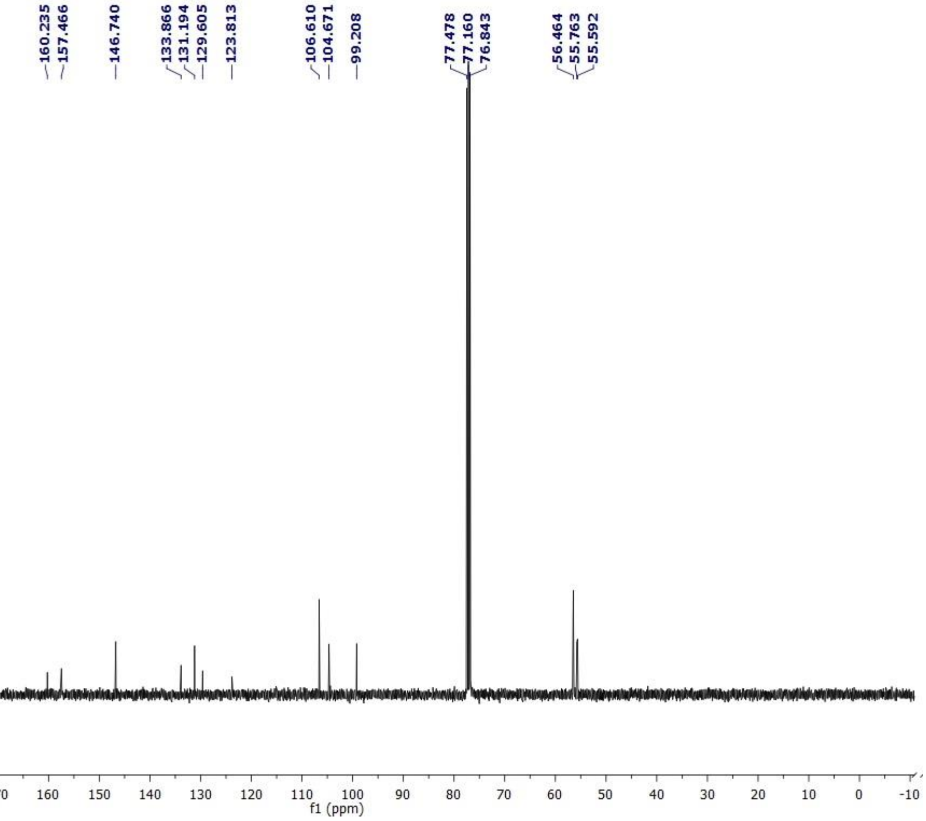

3q ^{1}H NMR (400 MHz, $CDCl_3$)

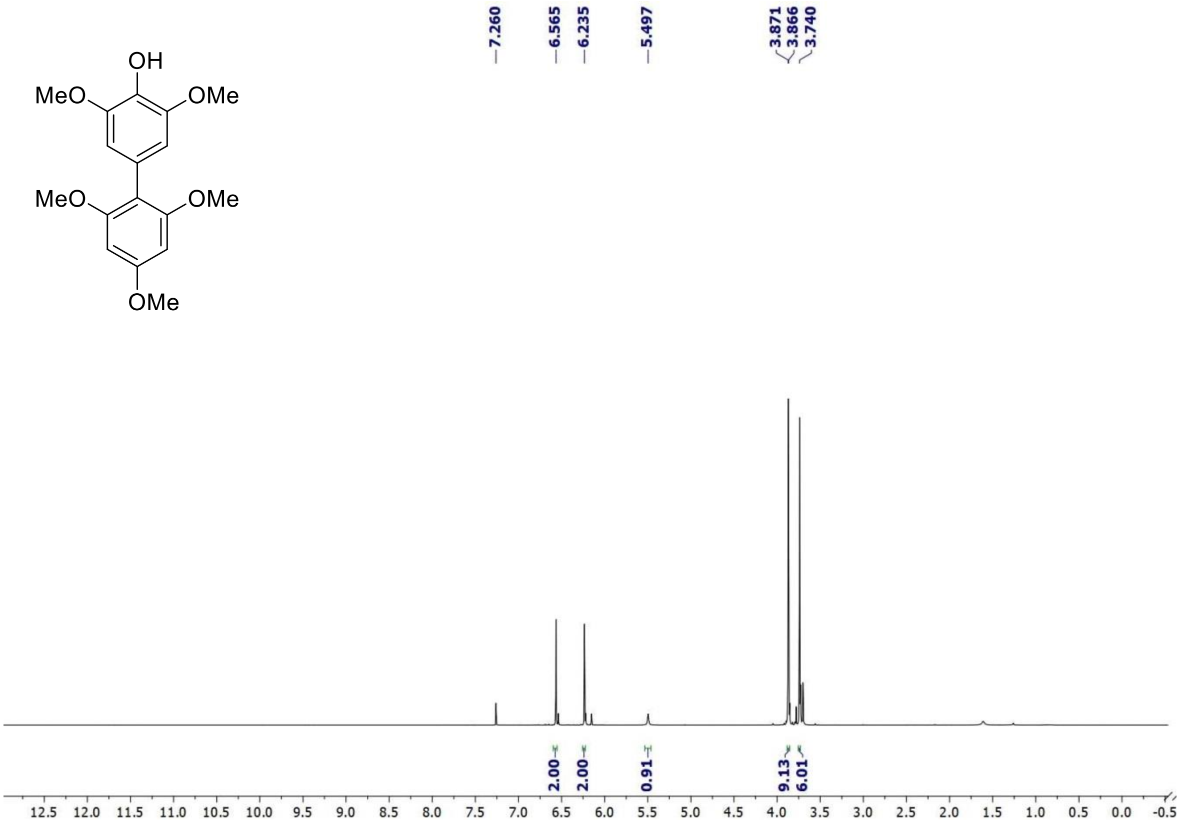

3q ^{13}C NMR (100 MHz, $CDCl_3$)

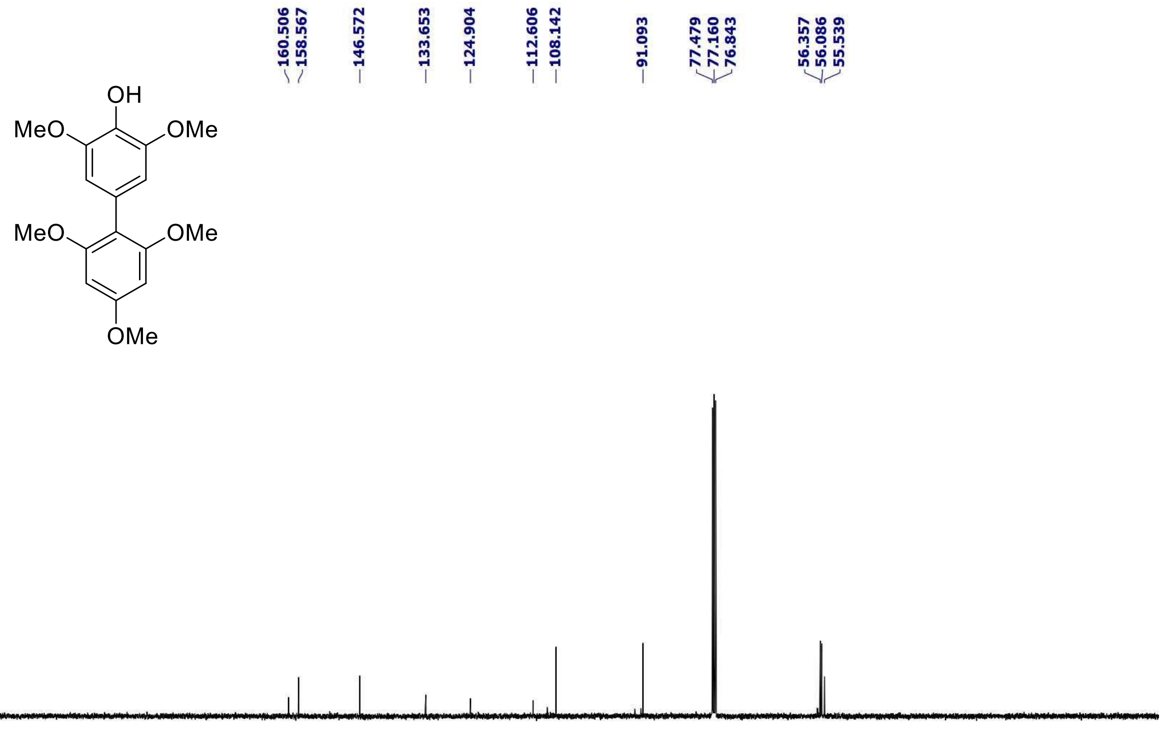

3r ^{1}H NMR (400 MHz, $CDCl_3$)

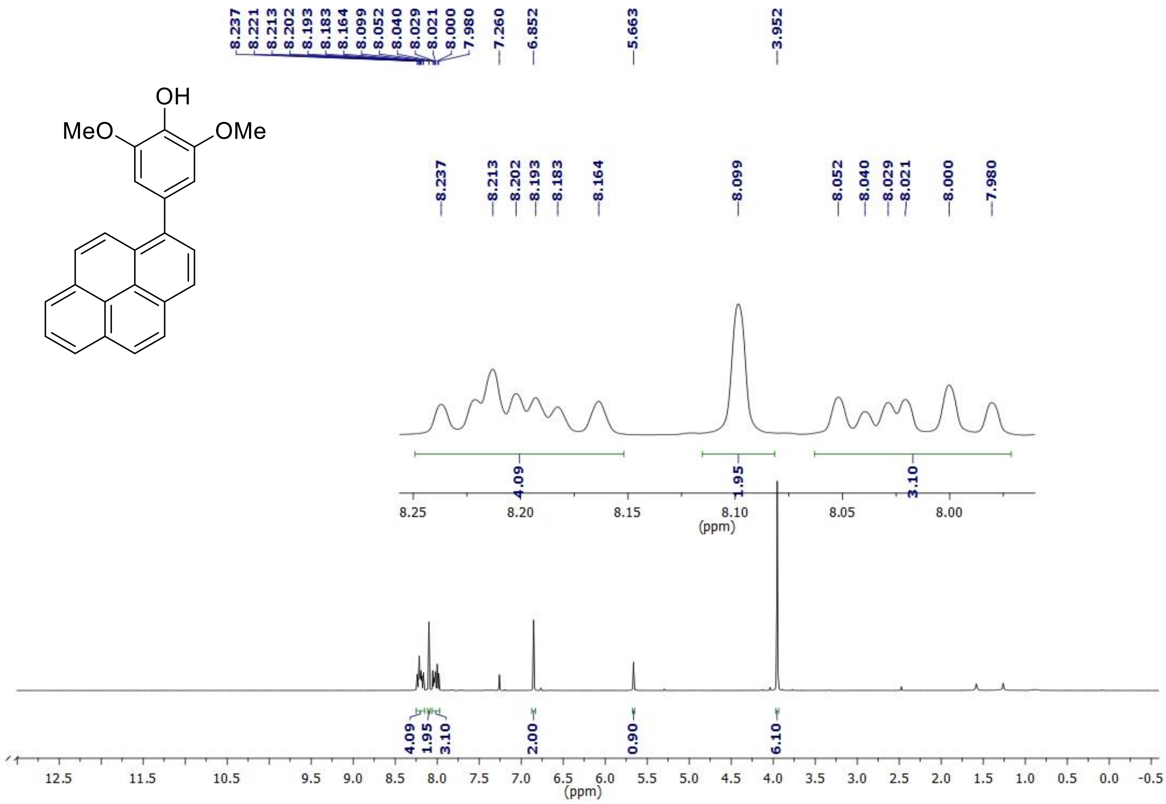

3r ^{13}C NMR (100 MHz, $CDCl_3$)

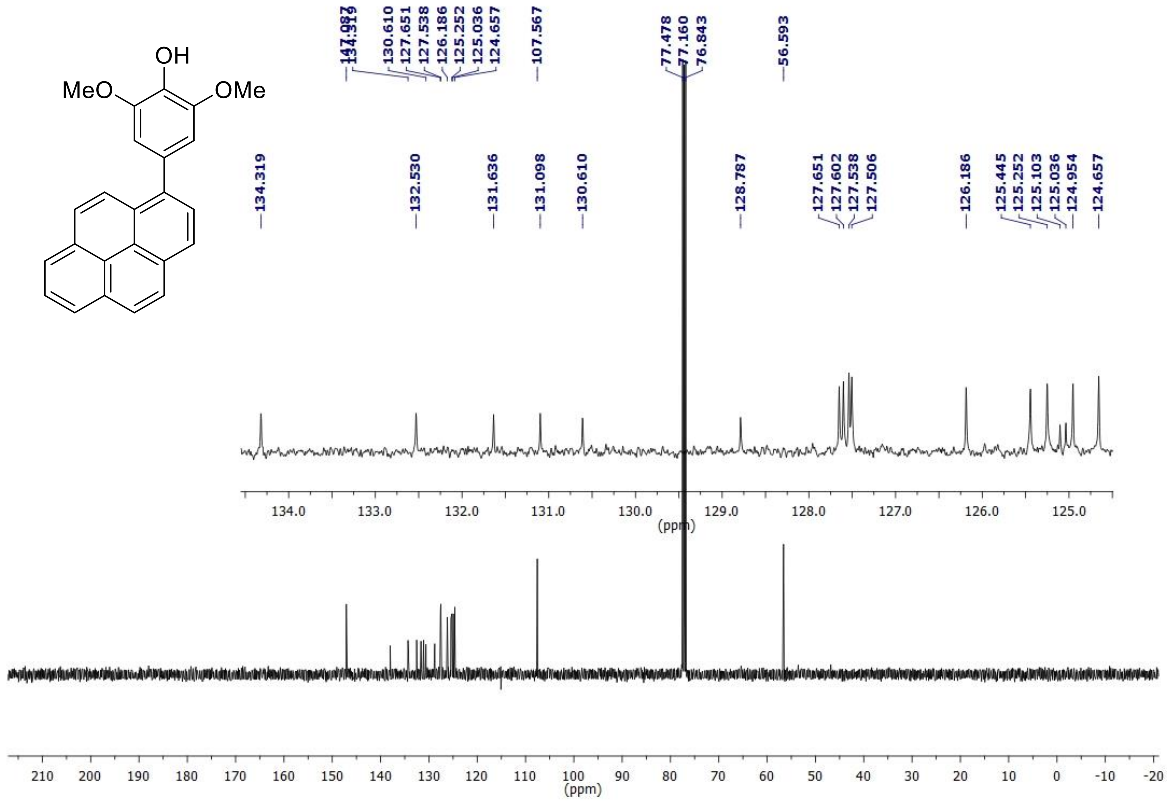

3s ^{1}H NMR (400 MHz, $CDCl_3$)

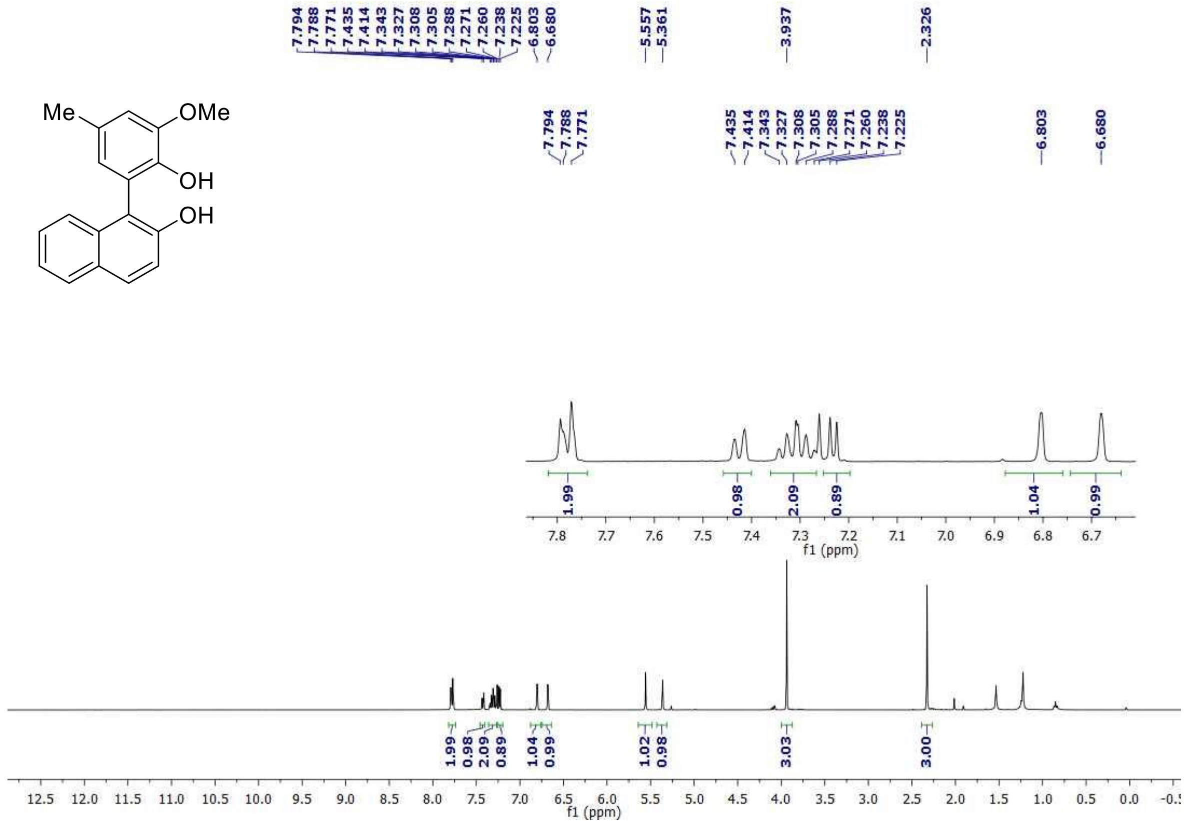

3s ^{13}C NMR (100 MHz, $CDCl_3$)

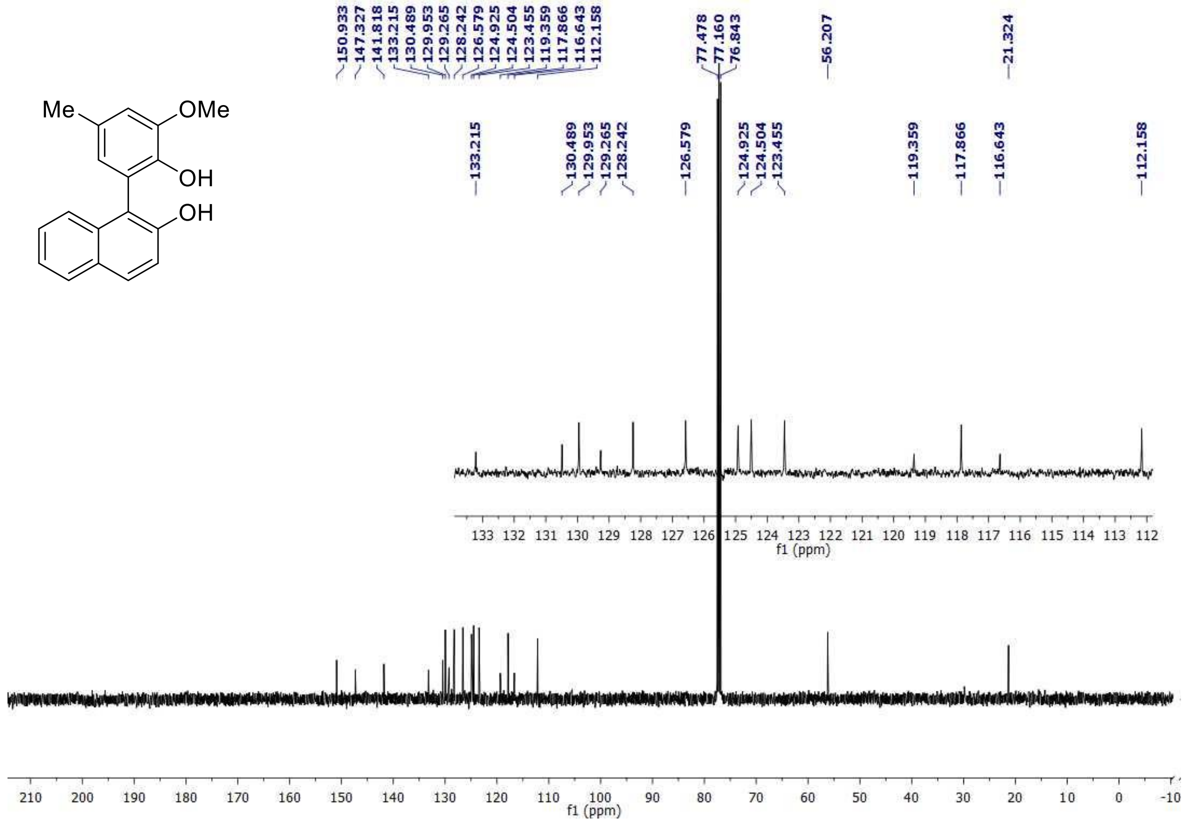

3t ^{1}H NMR (500 MHz, $CDCl_3$)

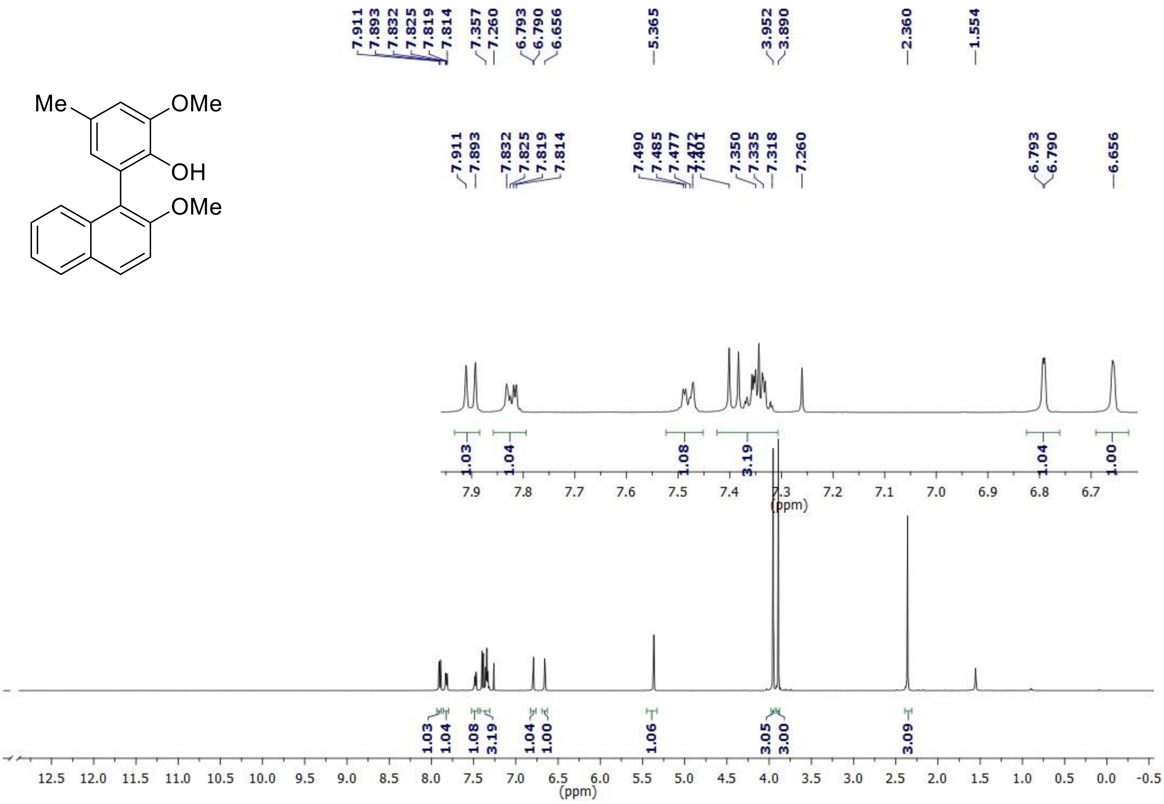

3t ^{13}C NMR (125 MHz, $CDCl_3$)

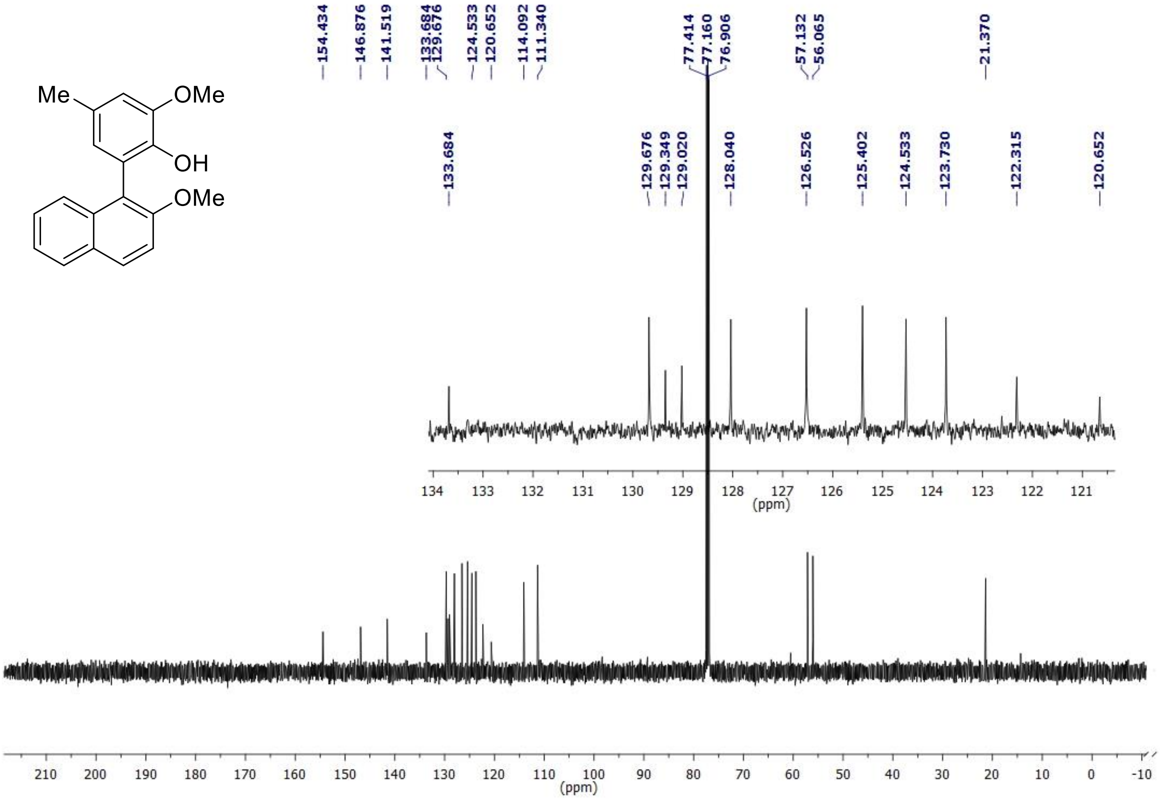

3u ^{1}H NMR (400 MHz, $CDCl_3$)

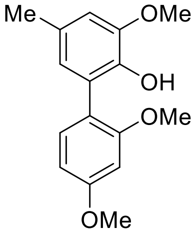

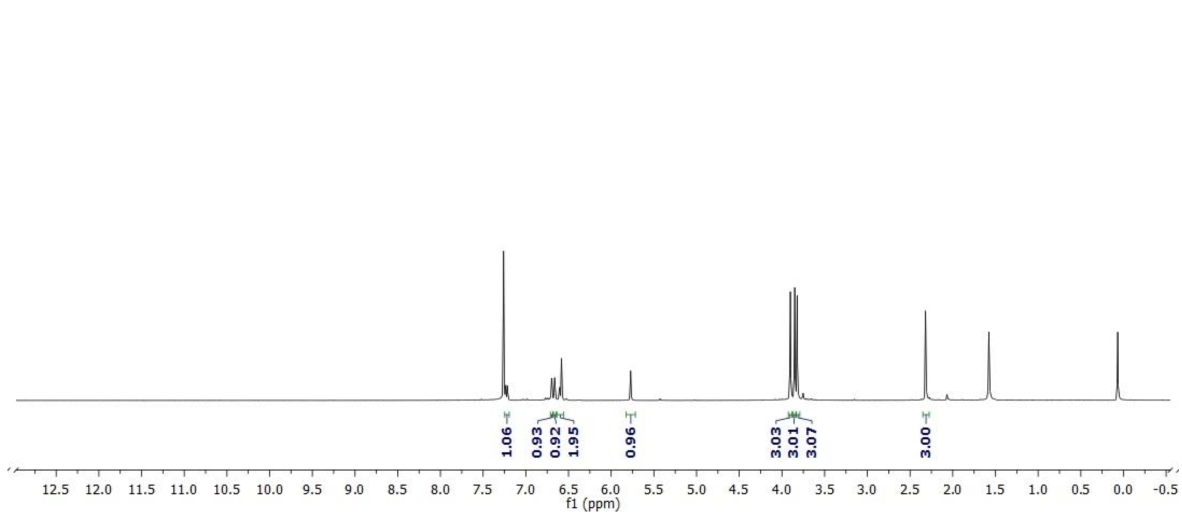

3u ^{13}C NMR (100 MHz, $CDCl_3$)

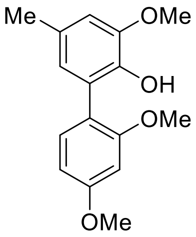

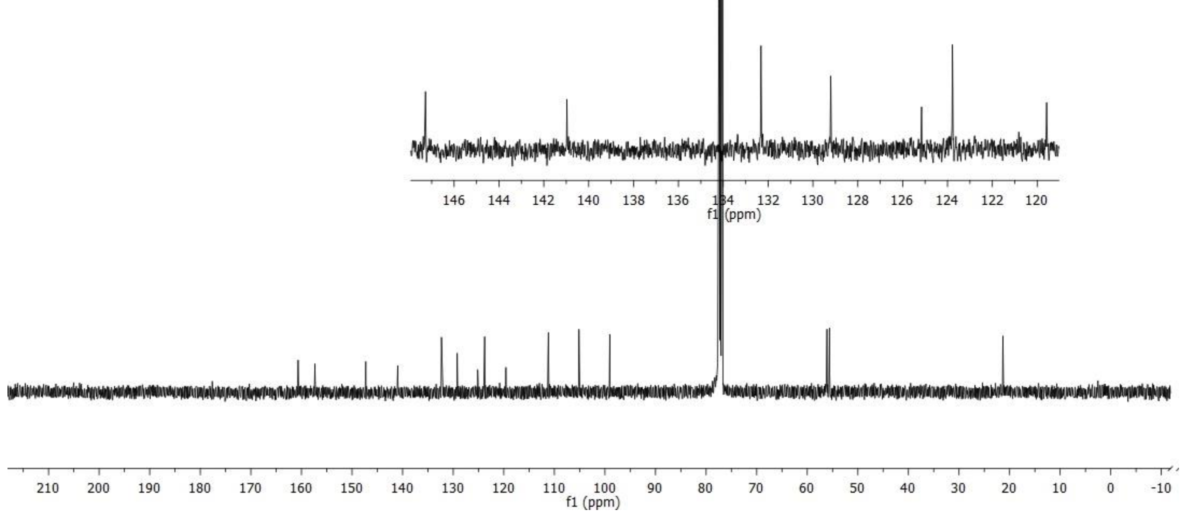

3v ^{1}H NMR (400 MHz, $CDCl_3$)

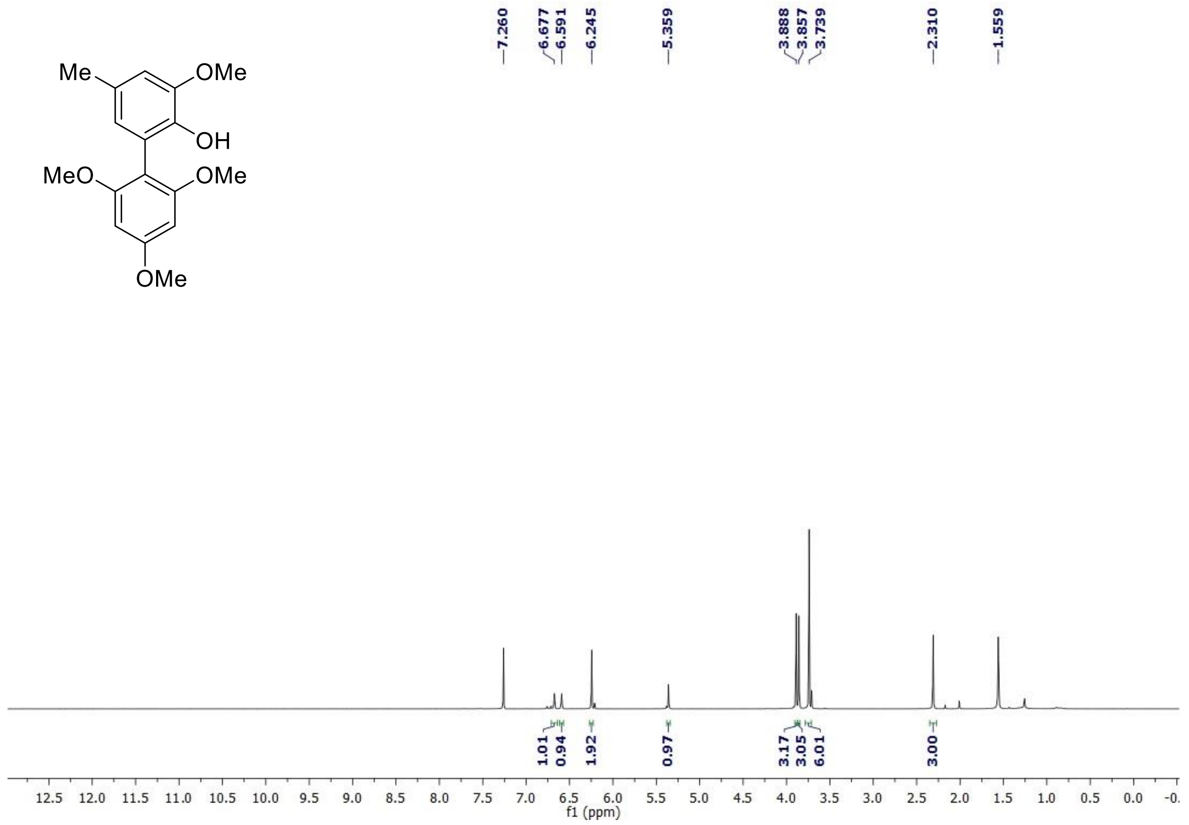

3v ^{13}C NMR (100 MHz, $CDCl_3$)

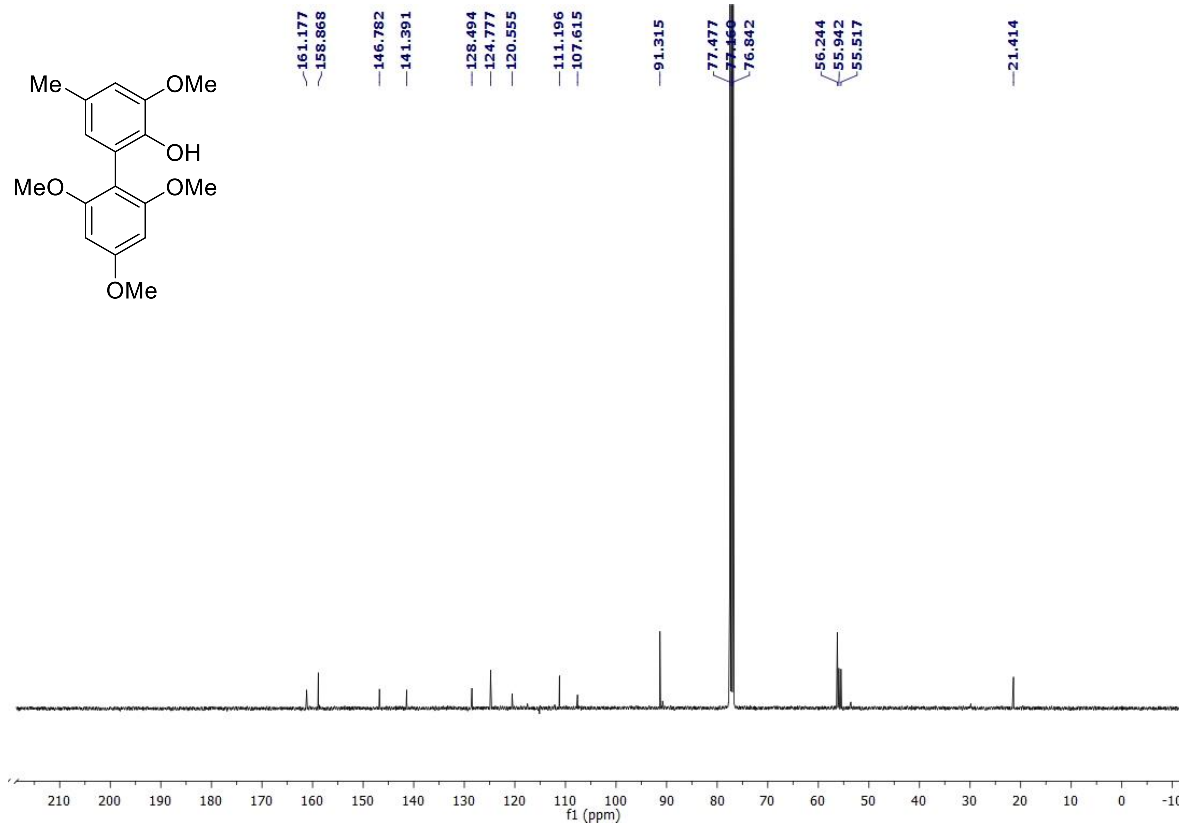

3w ^{1}H NMR (400 MHz, $CDCl_3$)

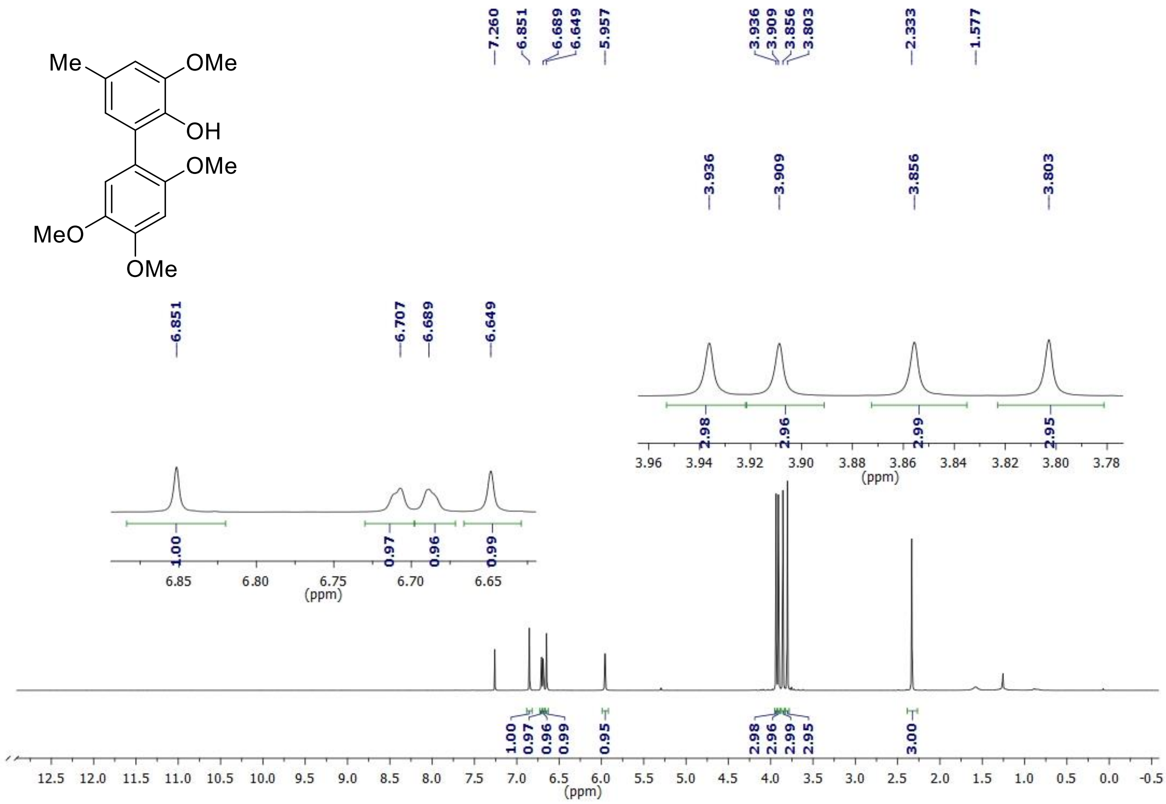

3w ^{13}C NMR (100 MHz, $CDCl_3$)

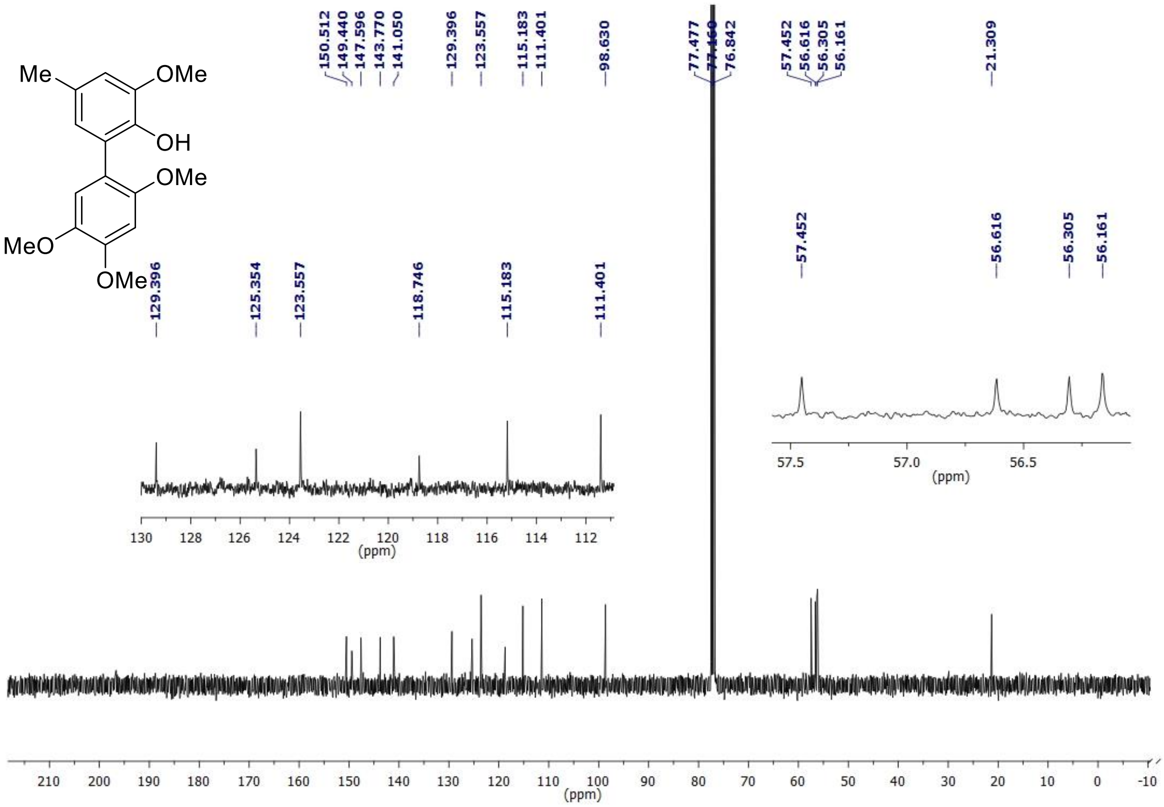

3x ^{1}H NMR (400 MHz, $CDCl_3$)

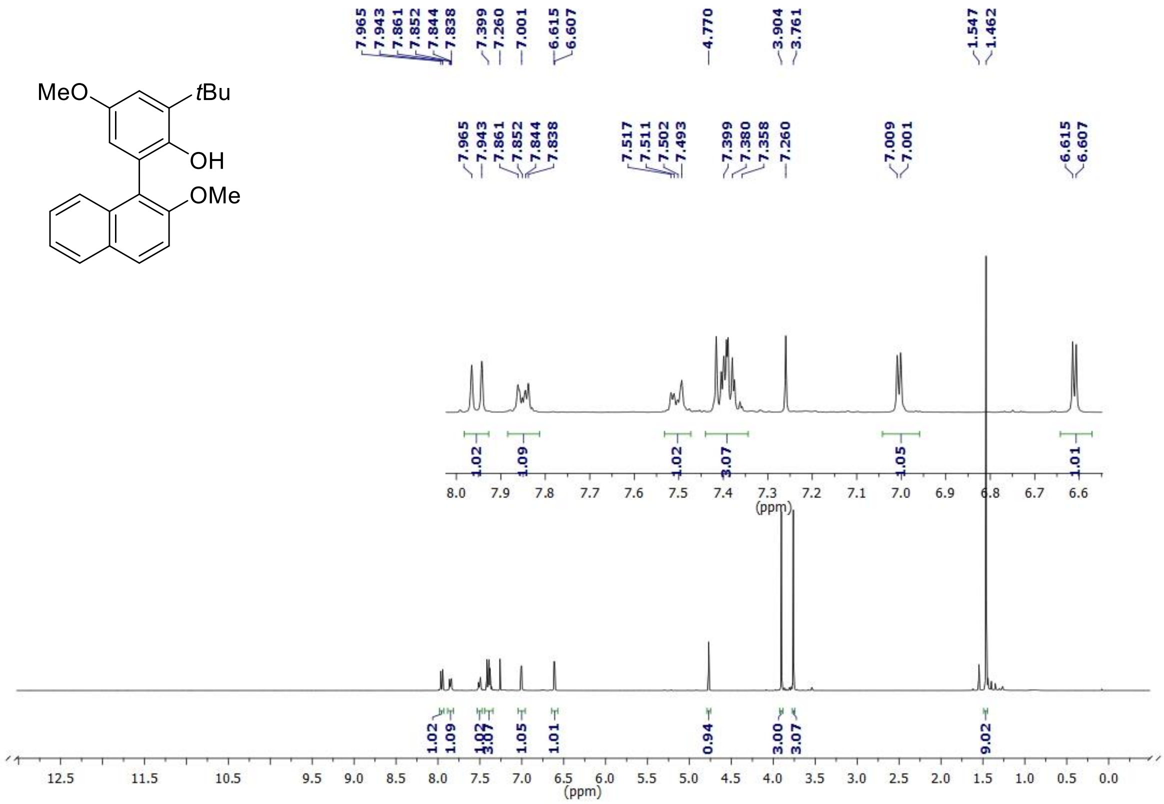

3x ^{13}C NMR (100 MHz, $CDCl_3$)

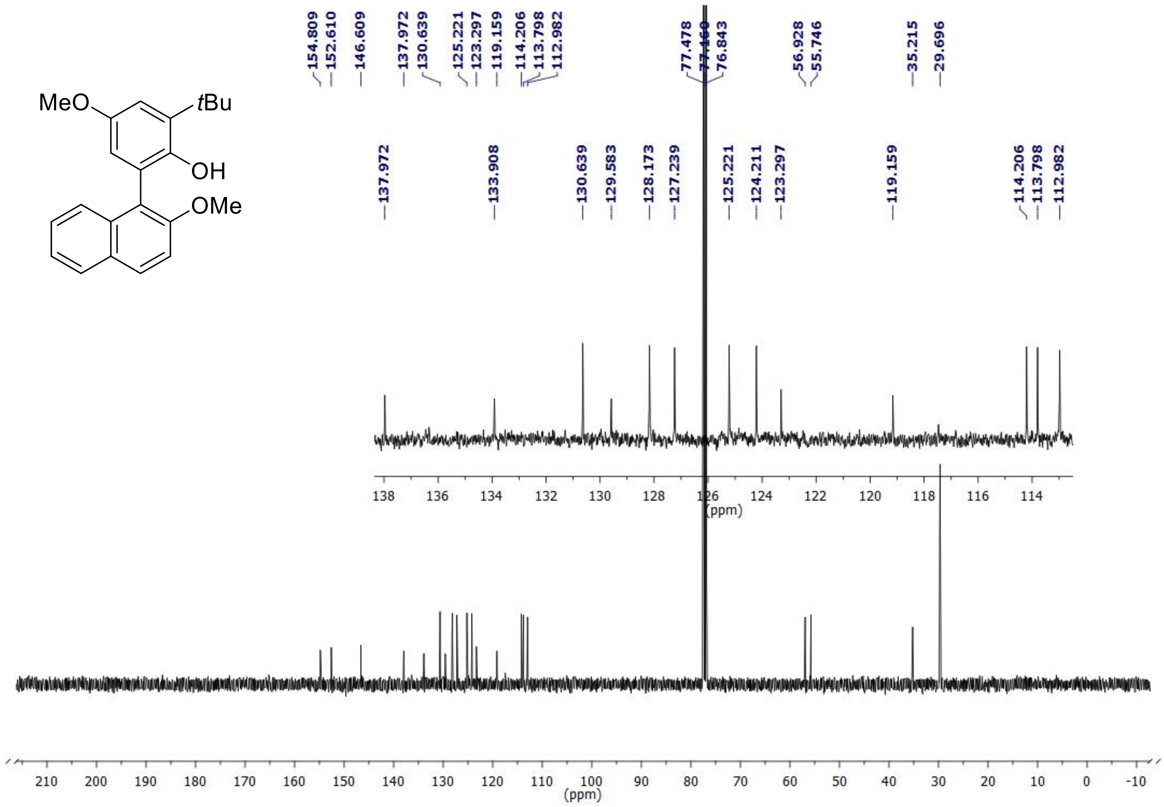

3y ^{1}H NMR (400 MHz, $CDCl_3$)

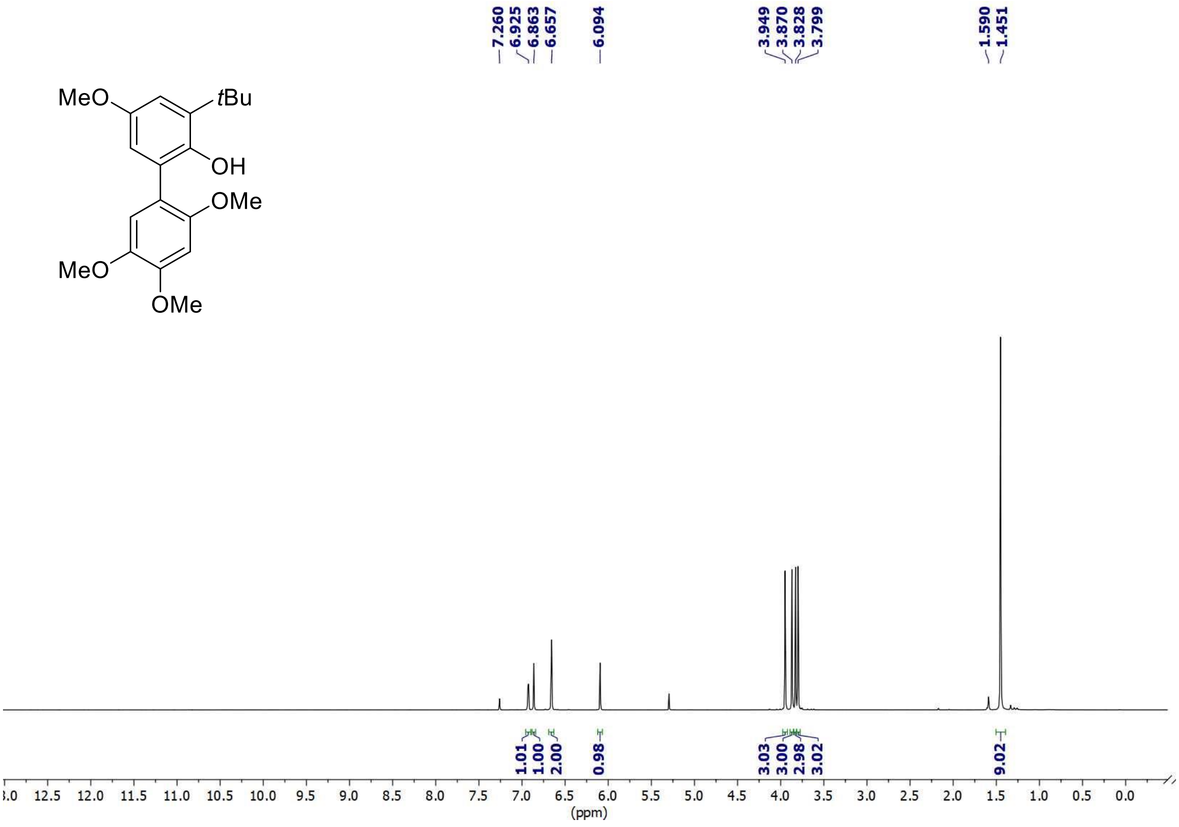

3y ^{13}C NMR (100 MHz, $CDCl_3$)

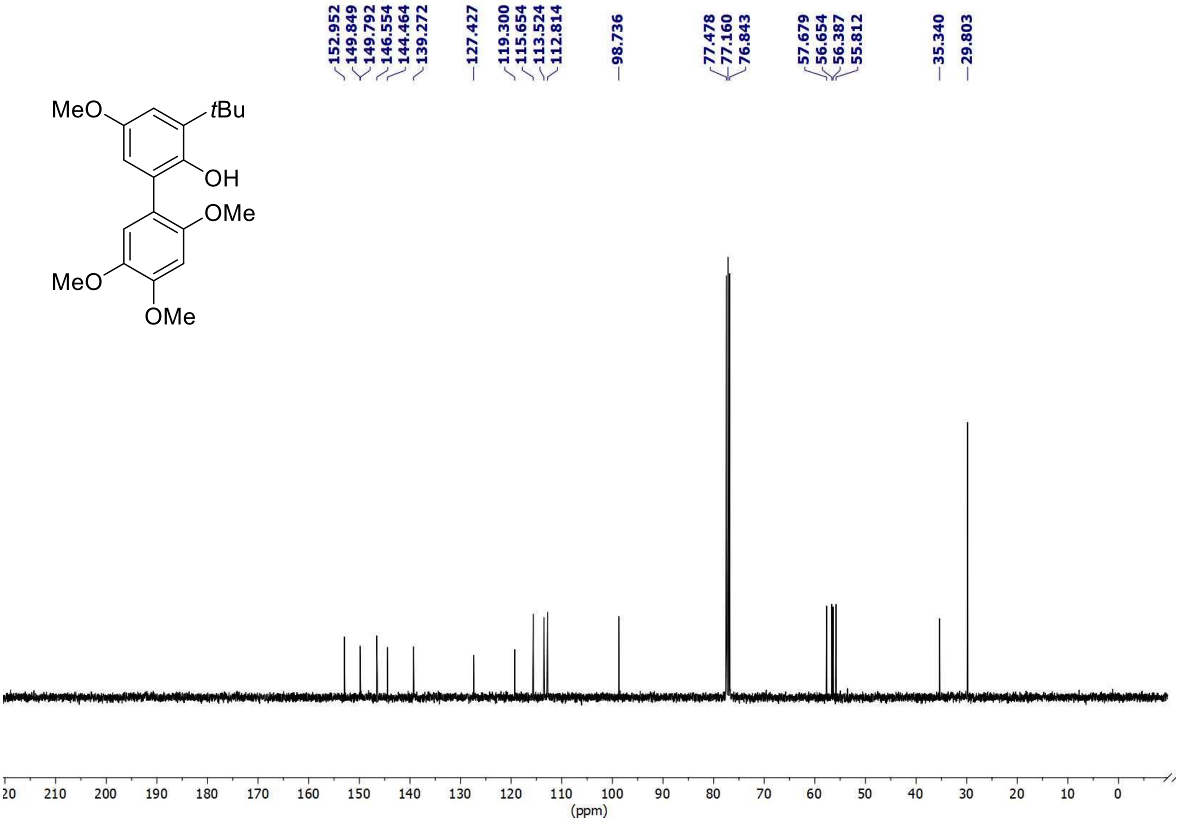

3z ^{1}H NMR (400 MHz, $CDCl_3$)

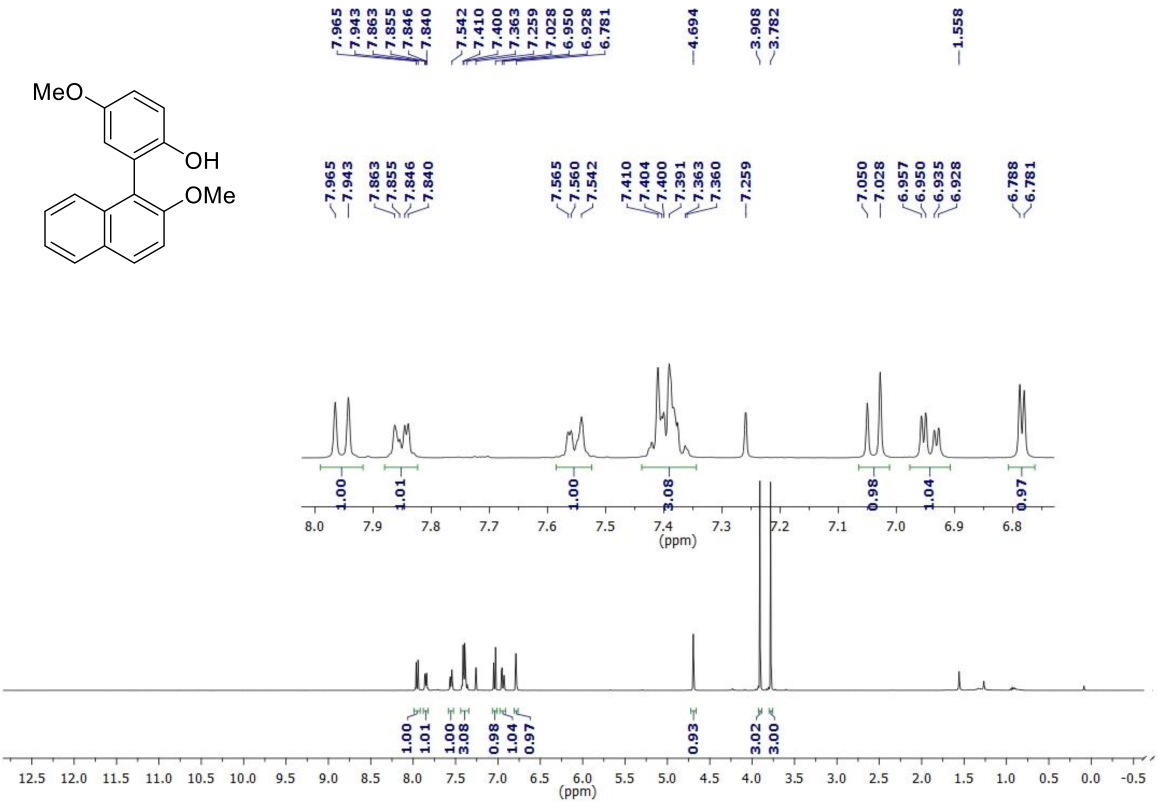

3z ^{13}C NMR (100 MHz, $CDCl_3$)

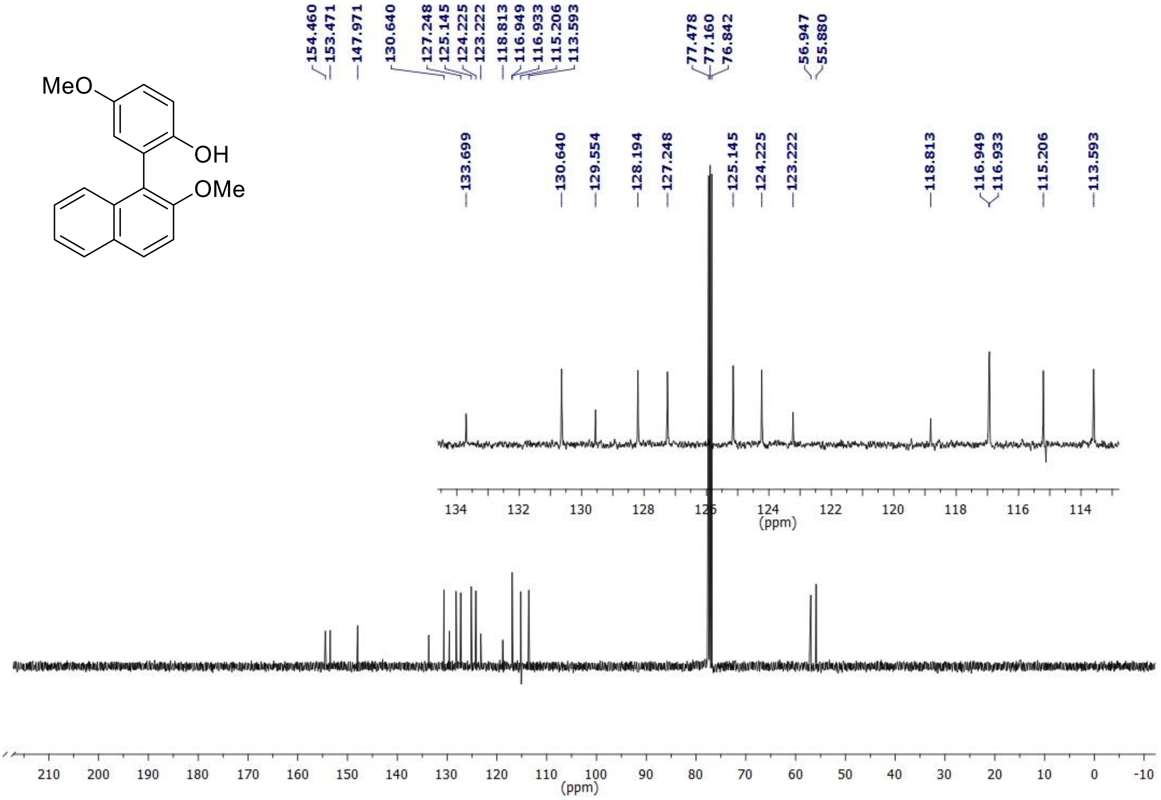

3aa ^{1}H NMR (400 MHz, $CDCl_3$)

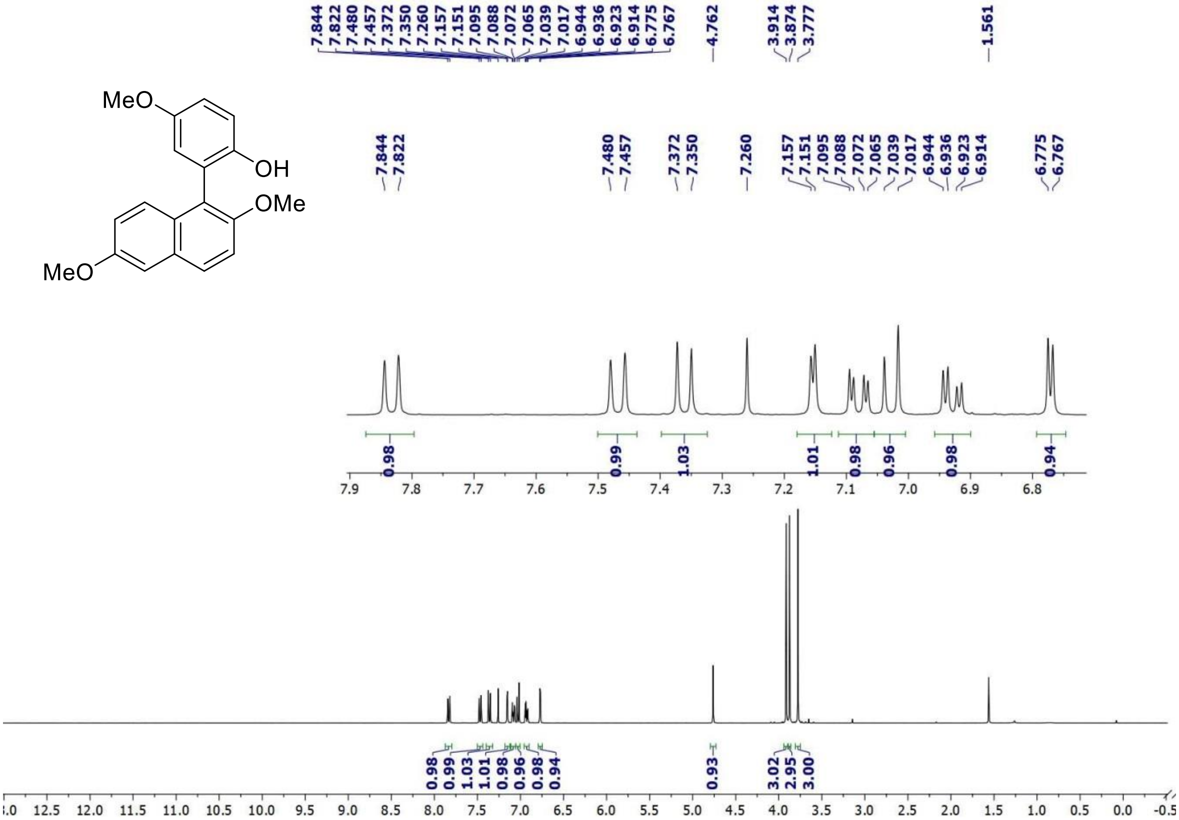

3aa ^{13}C NMR (100 MHz, $CDCl_3$)

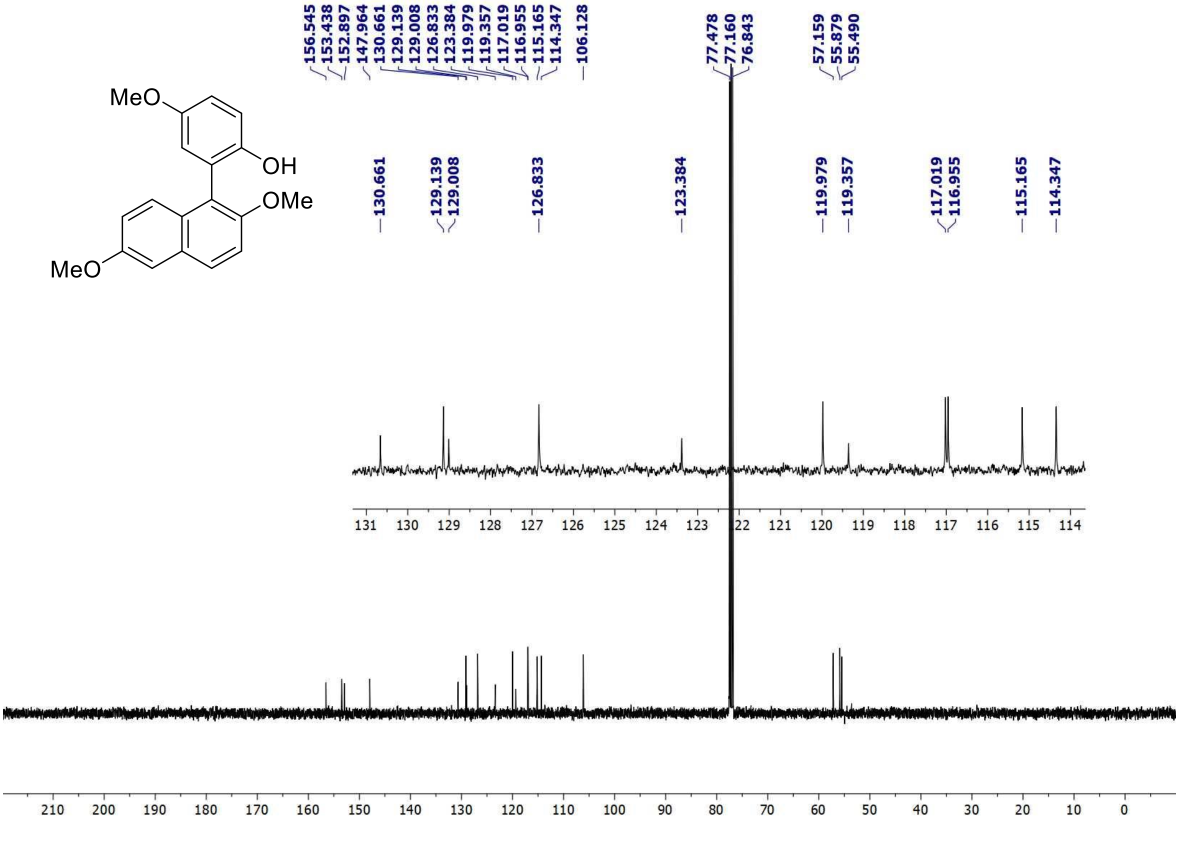

3ab ^{1}H NMR (400 MHz, $CDCl_3$)

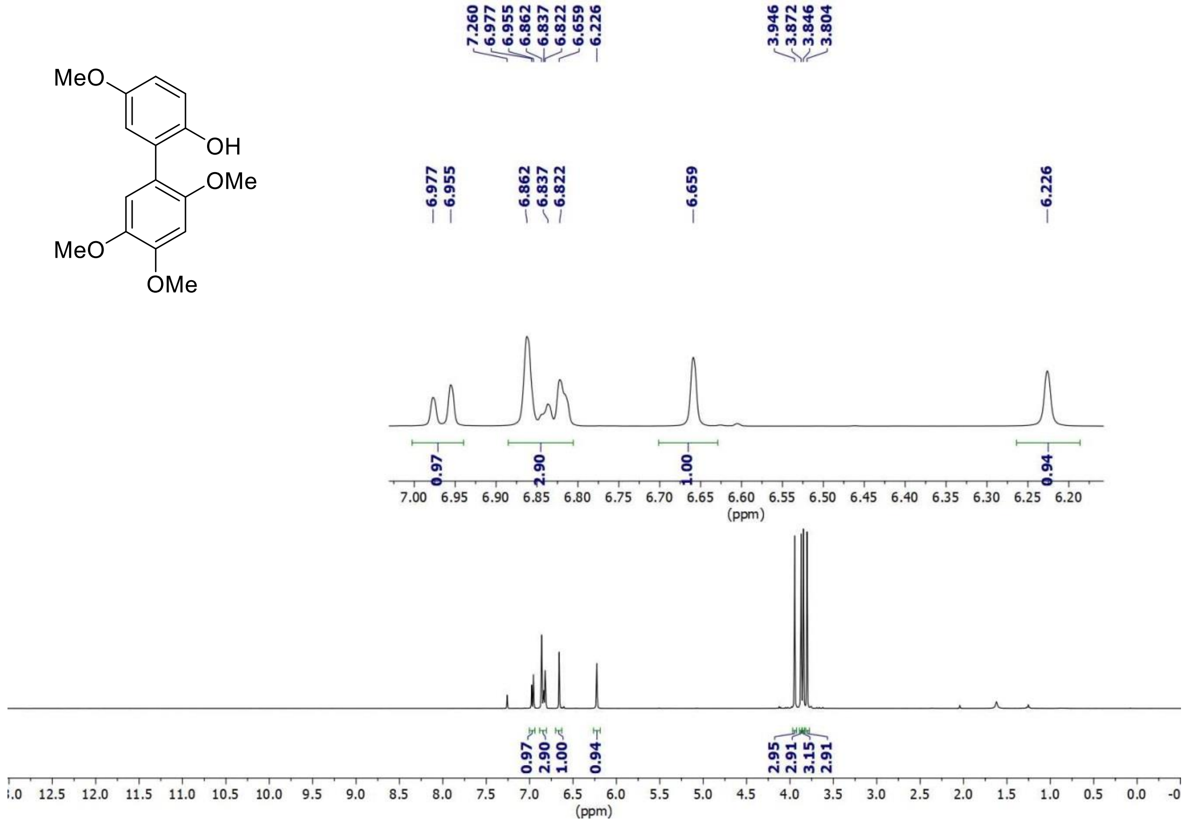

3ab ^{13}C NMR (100 MHz, $CDCl_3$)

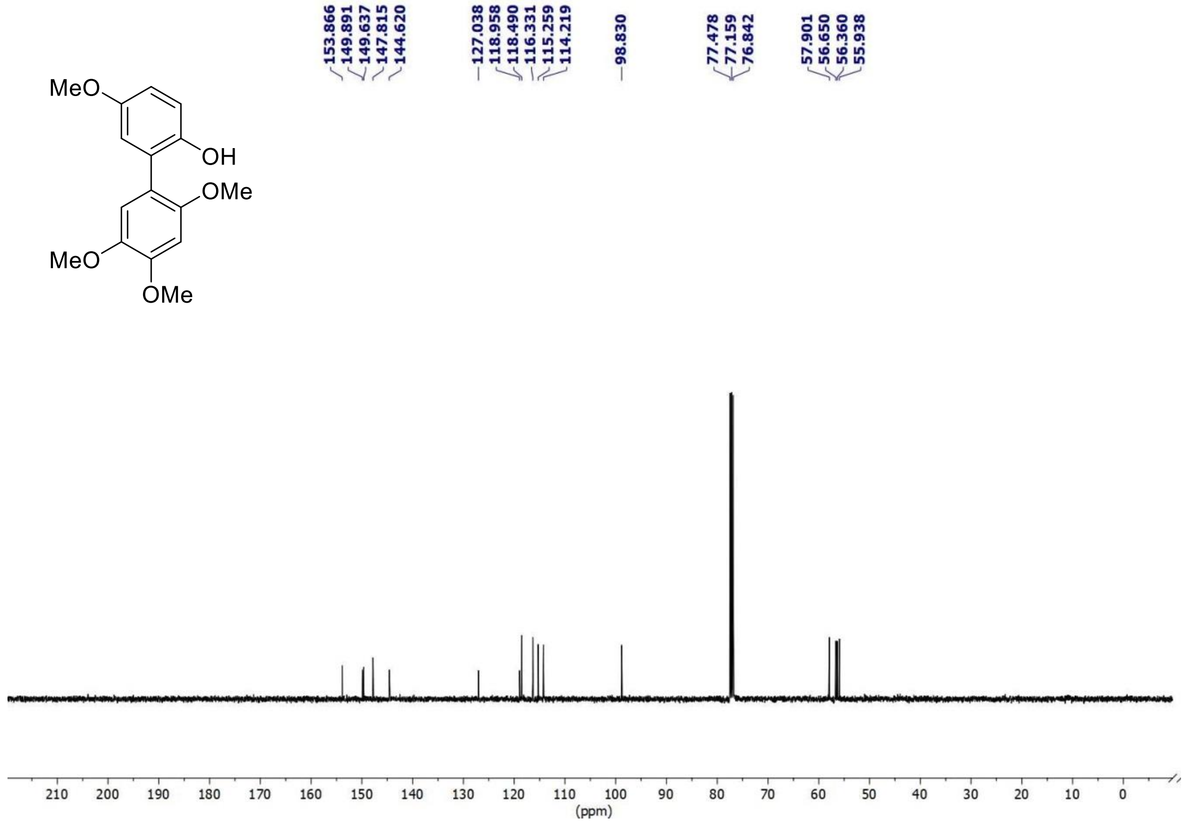

3ac ^{1}H NMR (400 MHz, $CDCl_3$)

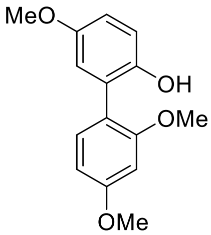

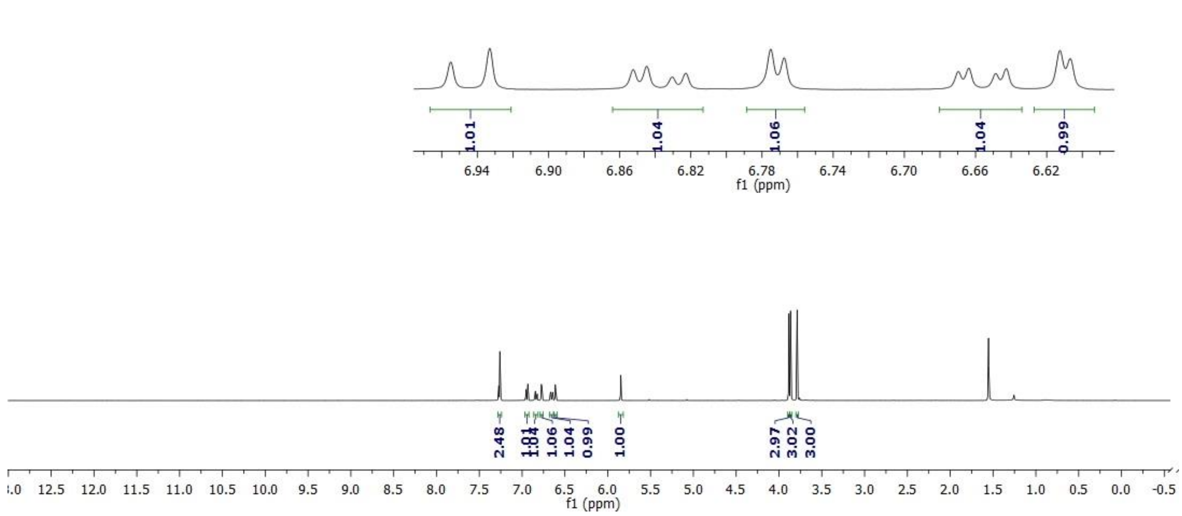

3ac ^{13}C NMR (100 MHz, $CDCl_3$)

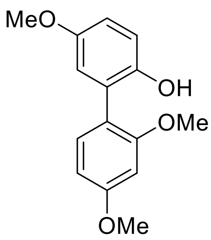

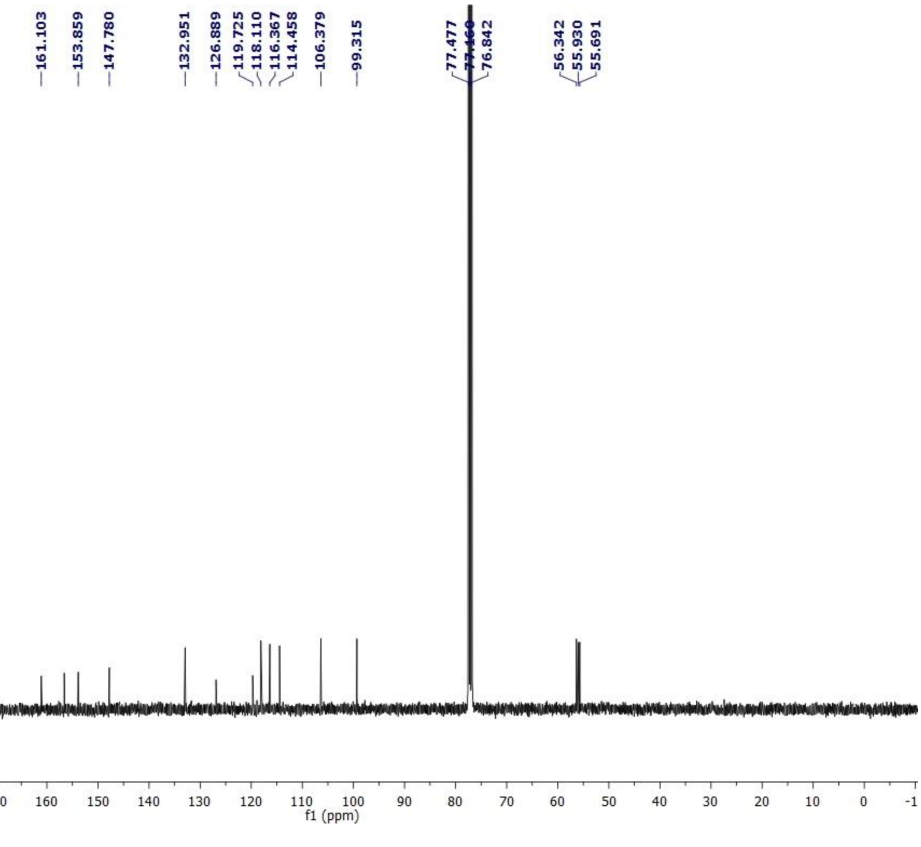

3ab' ^{1}H NMR (400 MHz, $CDCl_3$)

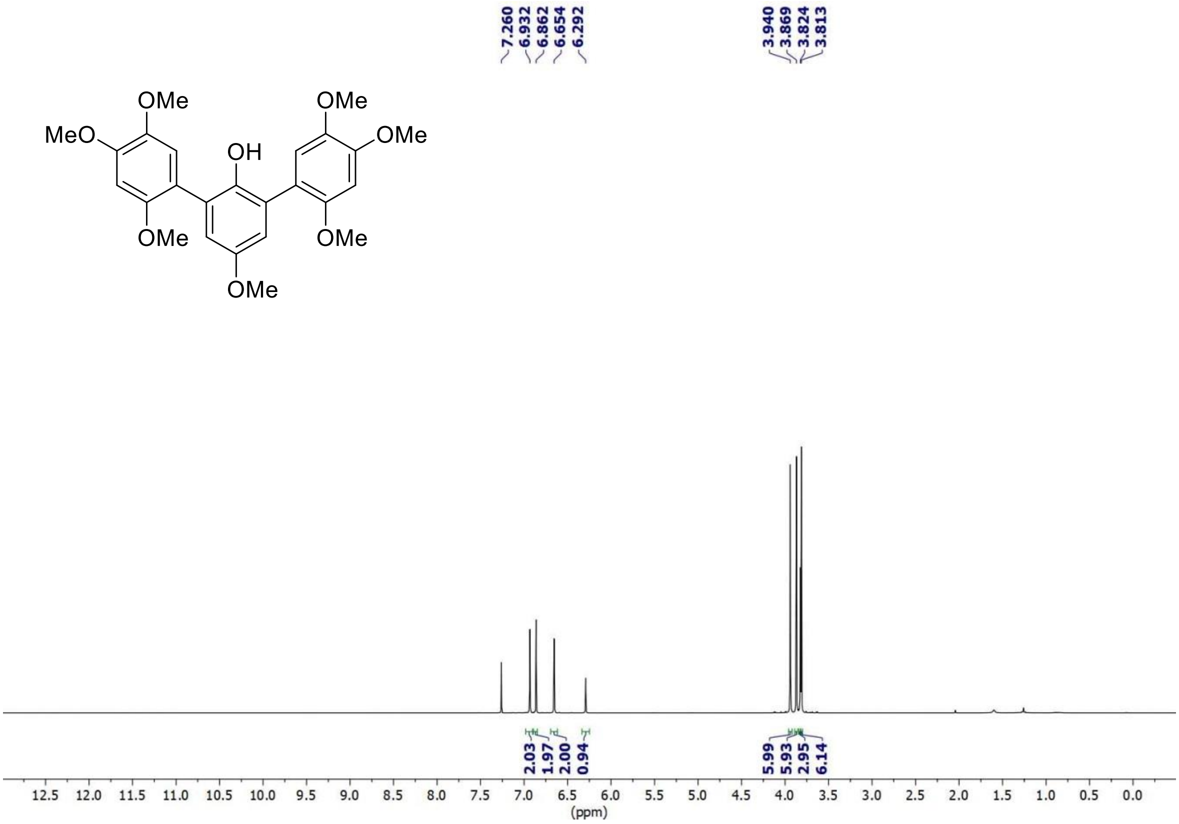

3ab' ^{13}C NMR (100 MHz, $CDCl_3$)

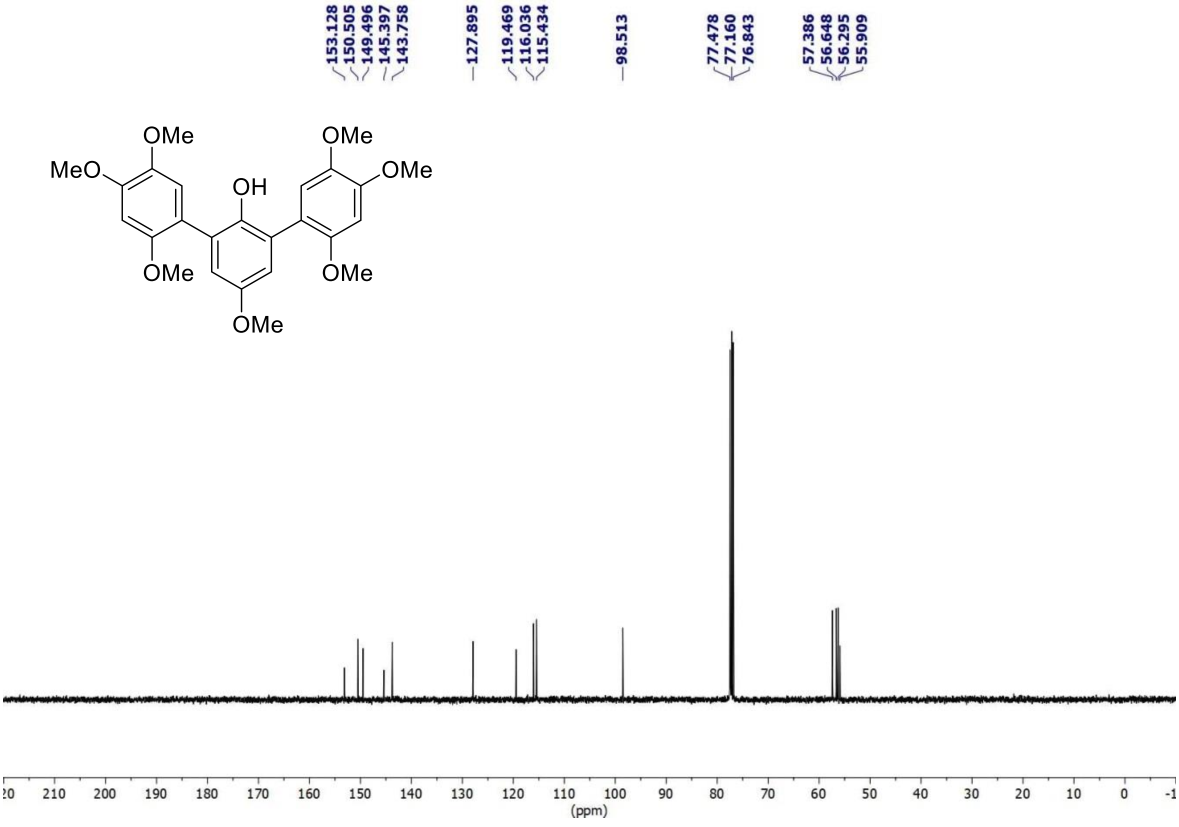

3ac' ^{1}H NMR (400 MHz, $CDCl_3$)

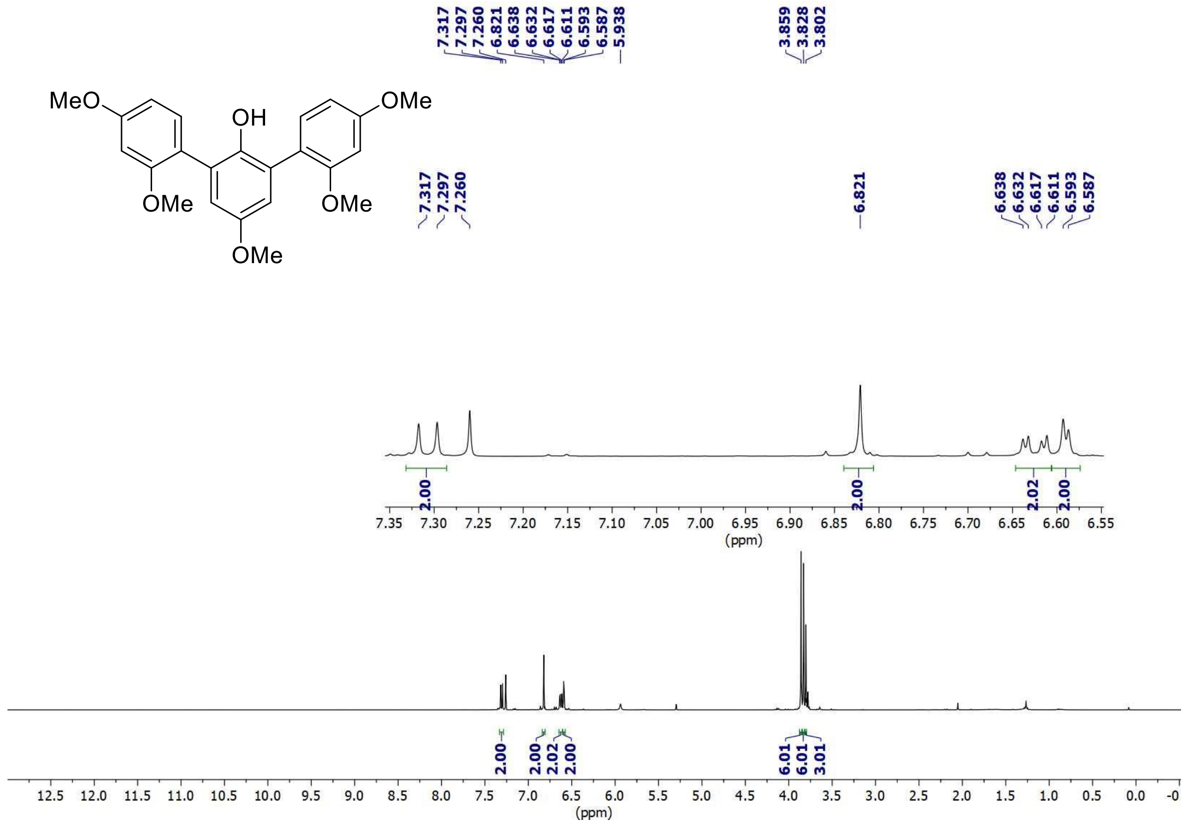

3ac' ^{13}C NMR (100 MHz, $CDCl_3$)

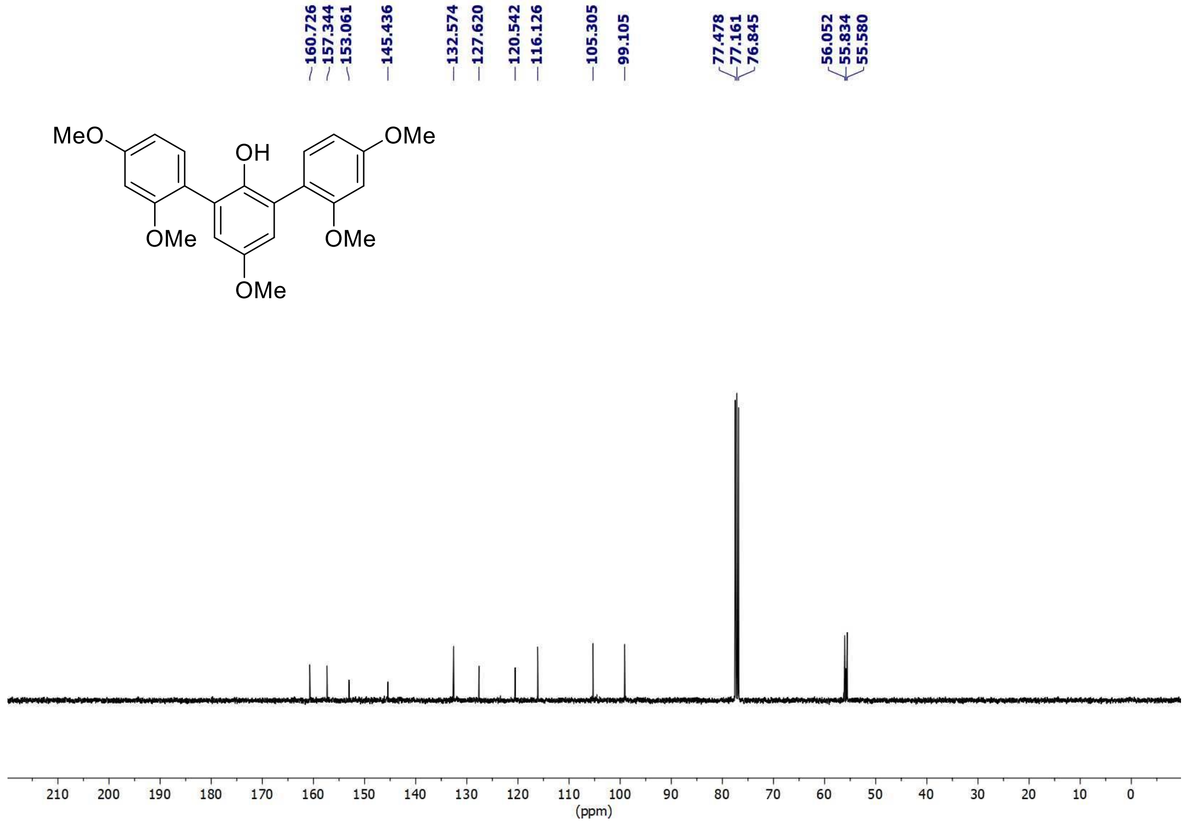

6a ^{1}H NMR (500 MHz, $CDCl_3$)

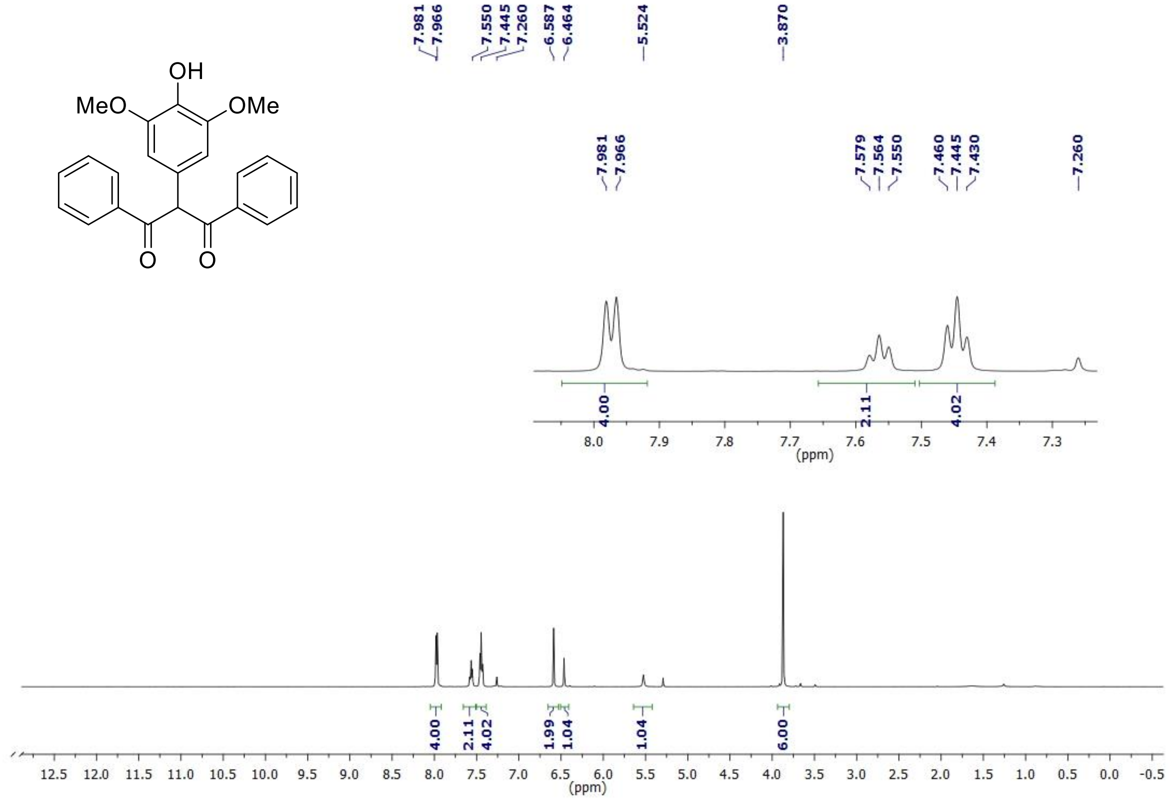

6a ^{13}C NMR (125 MHz, $CDCl_3$)

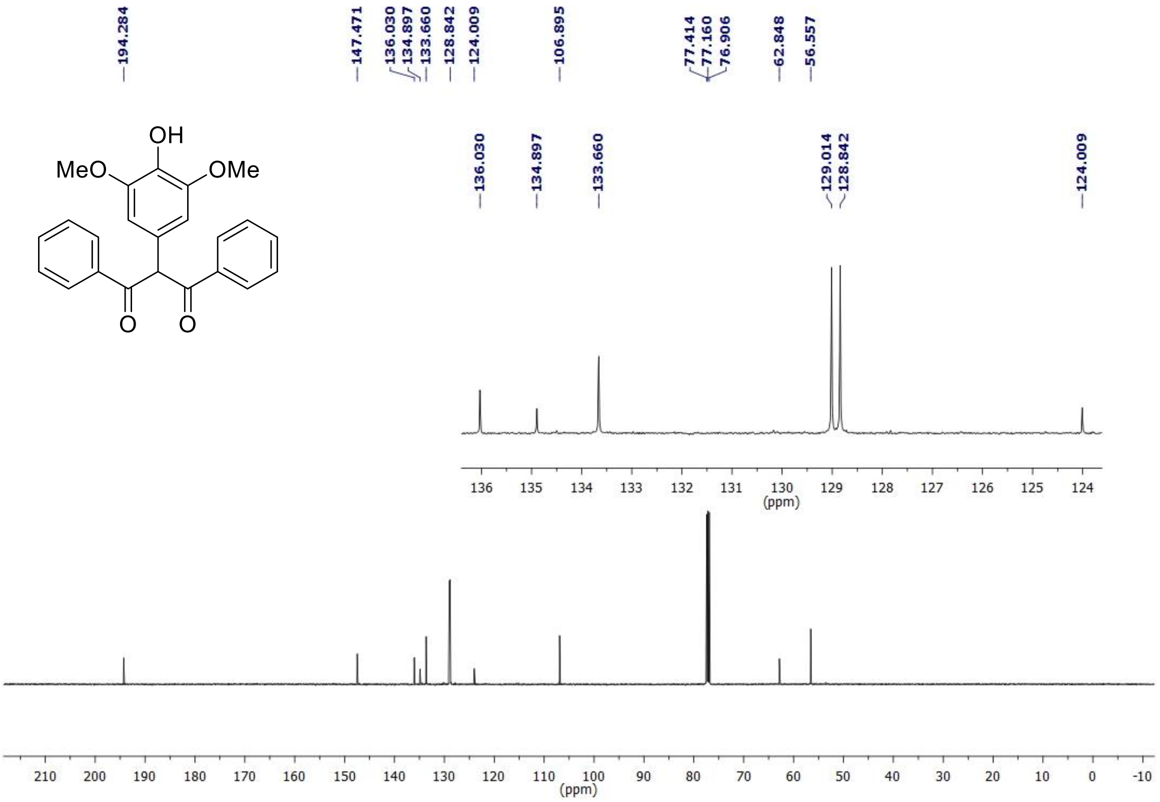

6b ^{1}H NMR (400 MHz, $CDCl_3$)

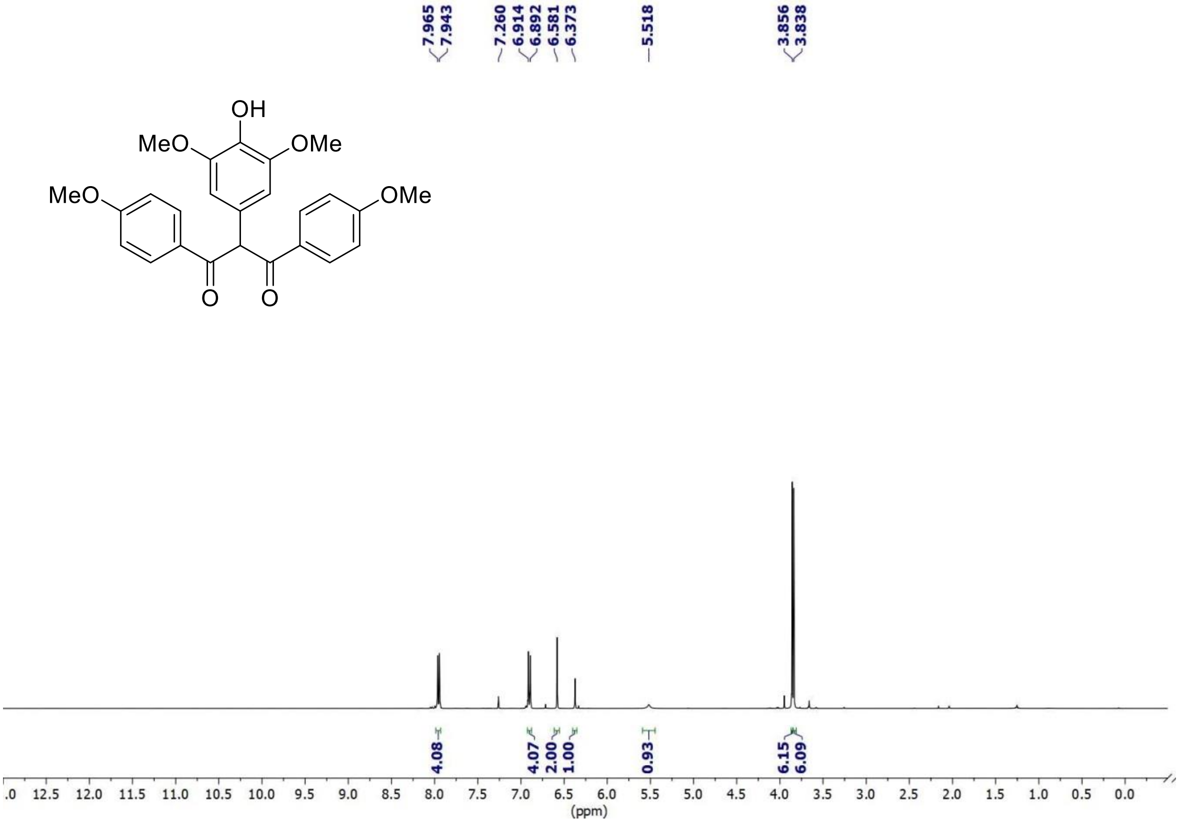

6b ^{13}C NMR (100 MHz, $CDCl_3$)

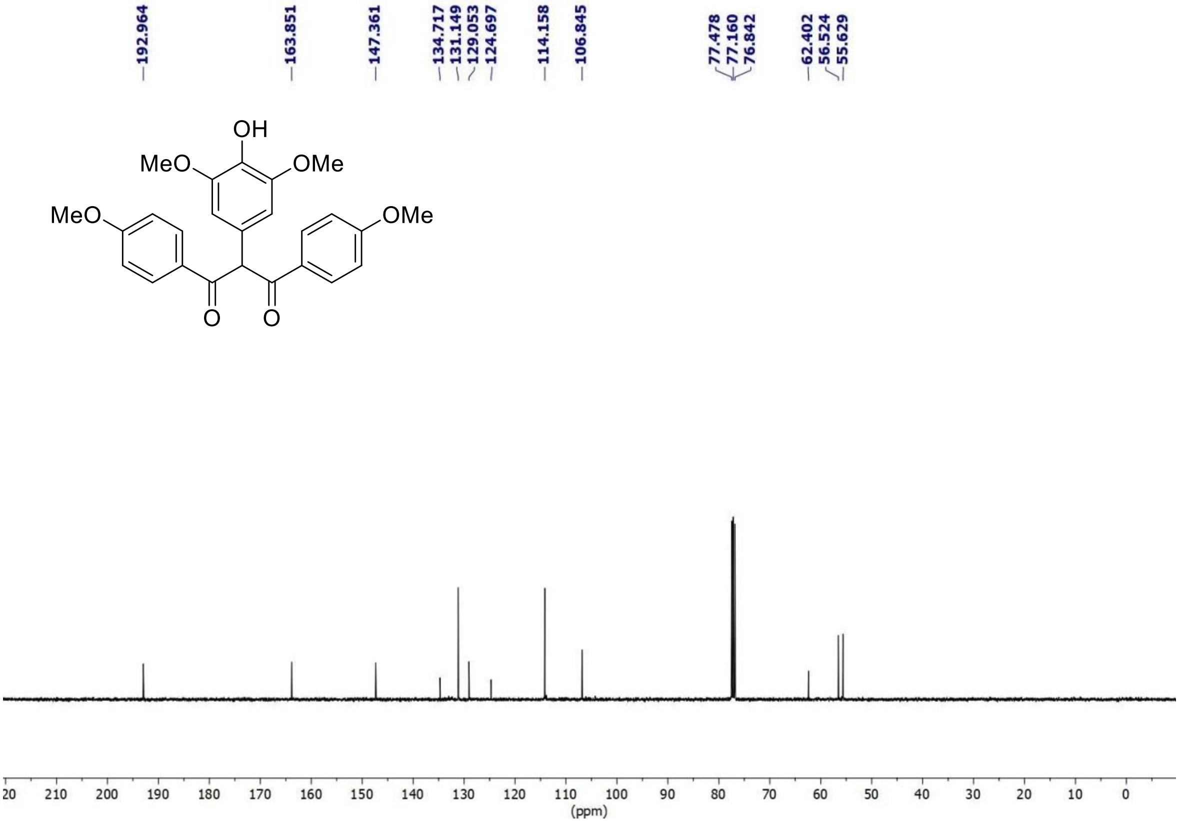

6c ^{1}H NMR (400 MHz, $CDCl_3$)

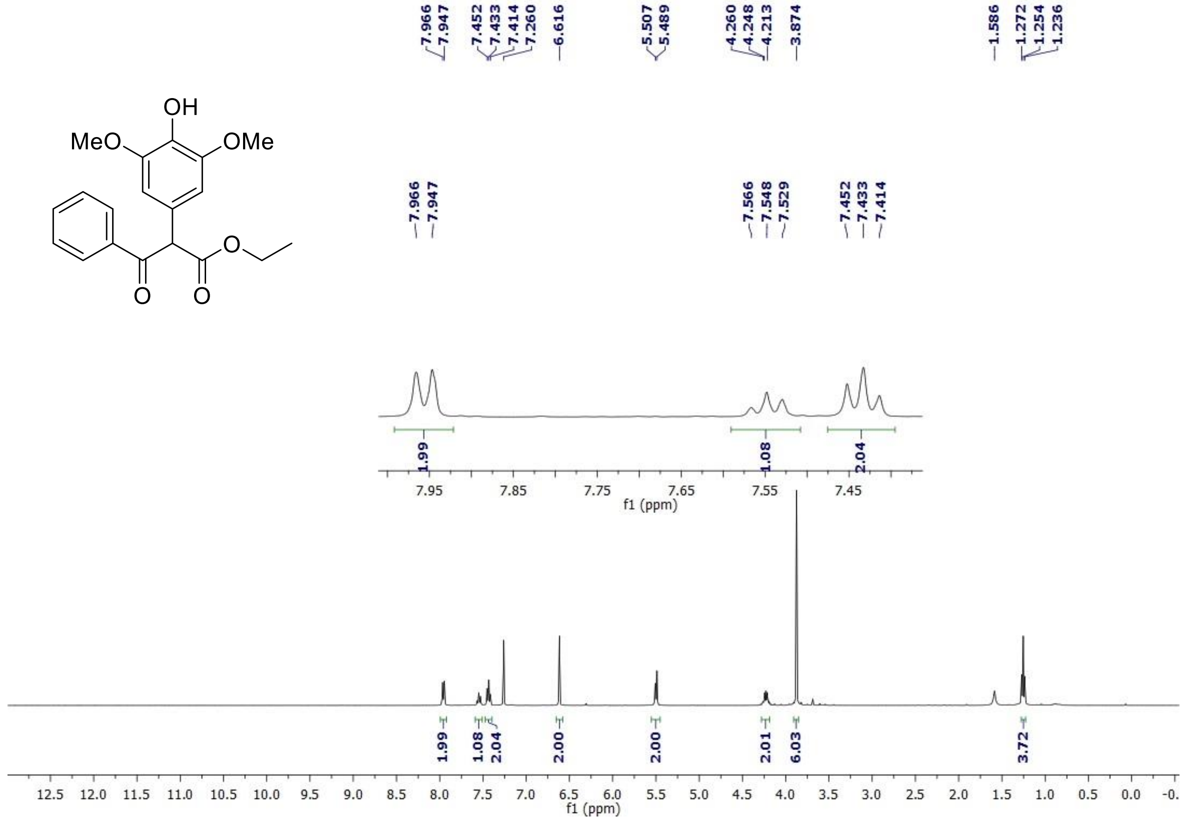

6c ^{13}C NMR (100 MHz, $CDCl_3$)

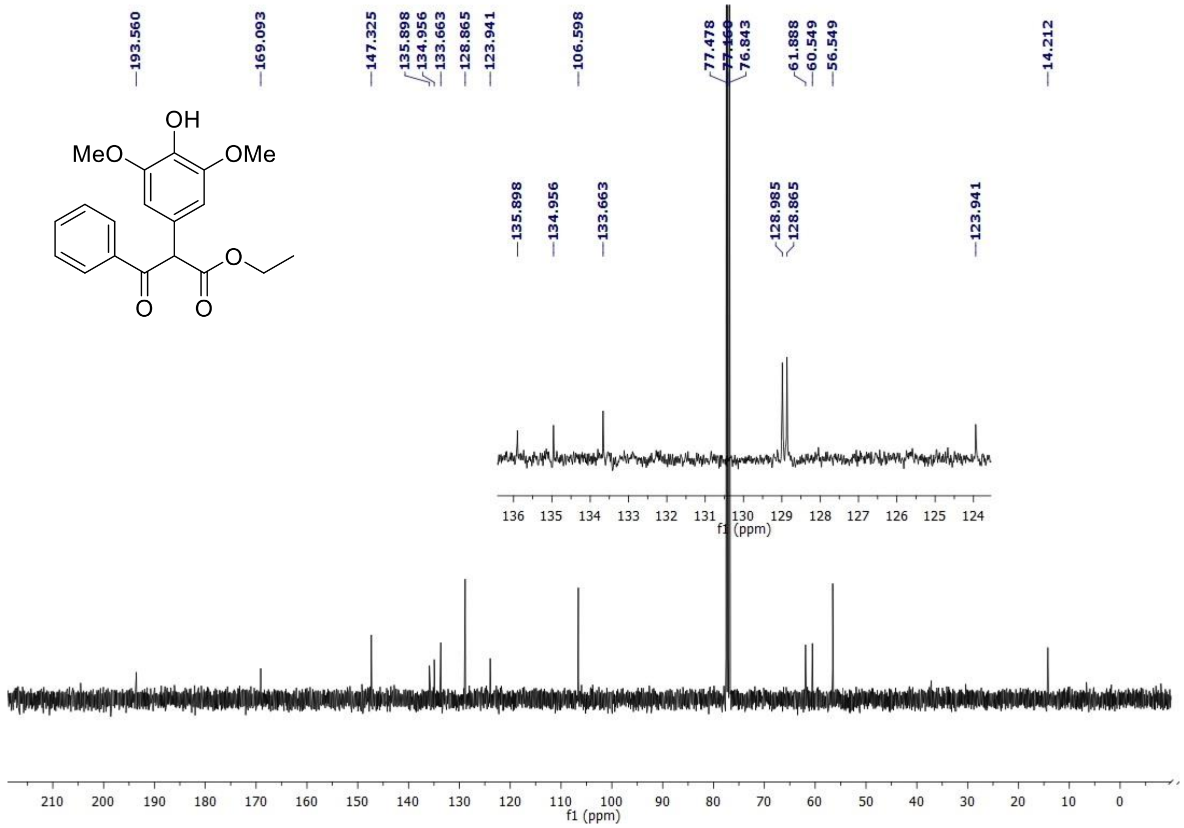

6d ^{1}H NMR (400 MHz, $CDCl_3$)

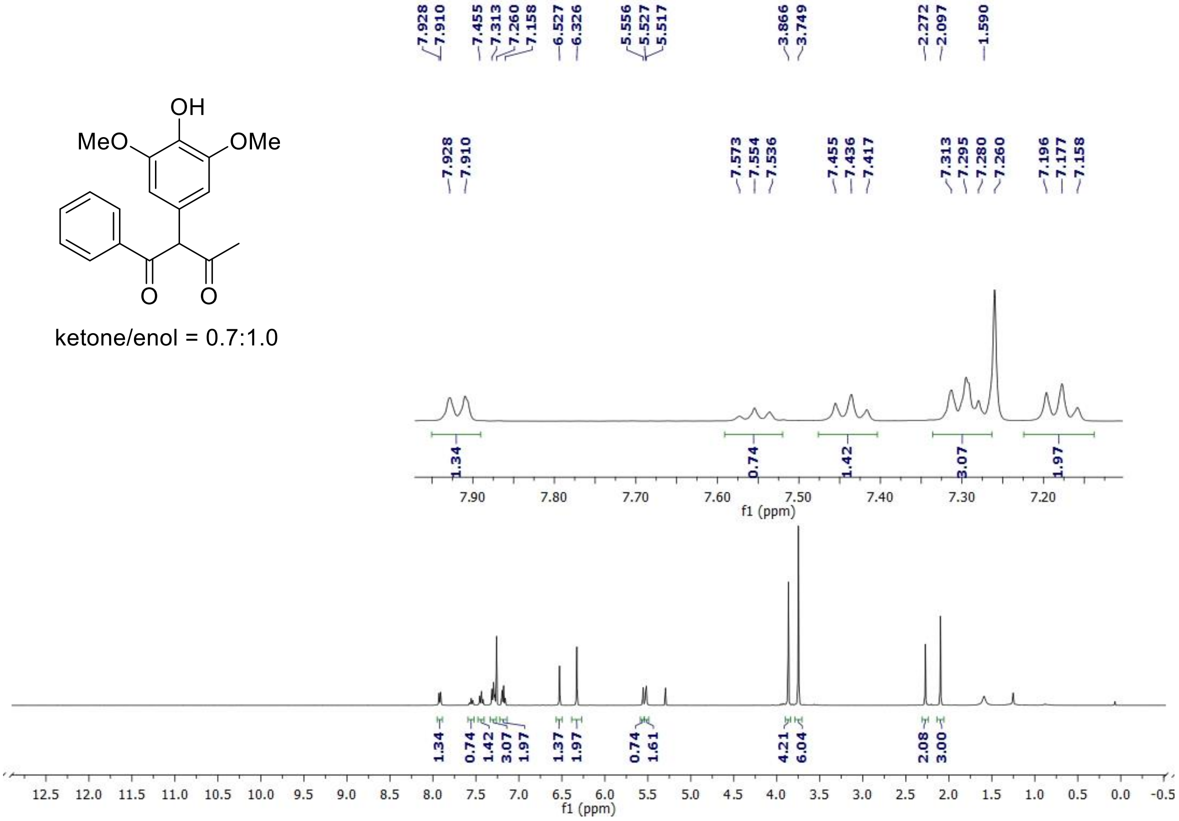

ketone/enol = 0.7:1.0

6d ^{13}C NMR (100 MHz, $CDCl_3$)

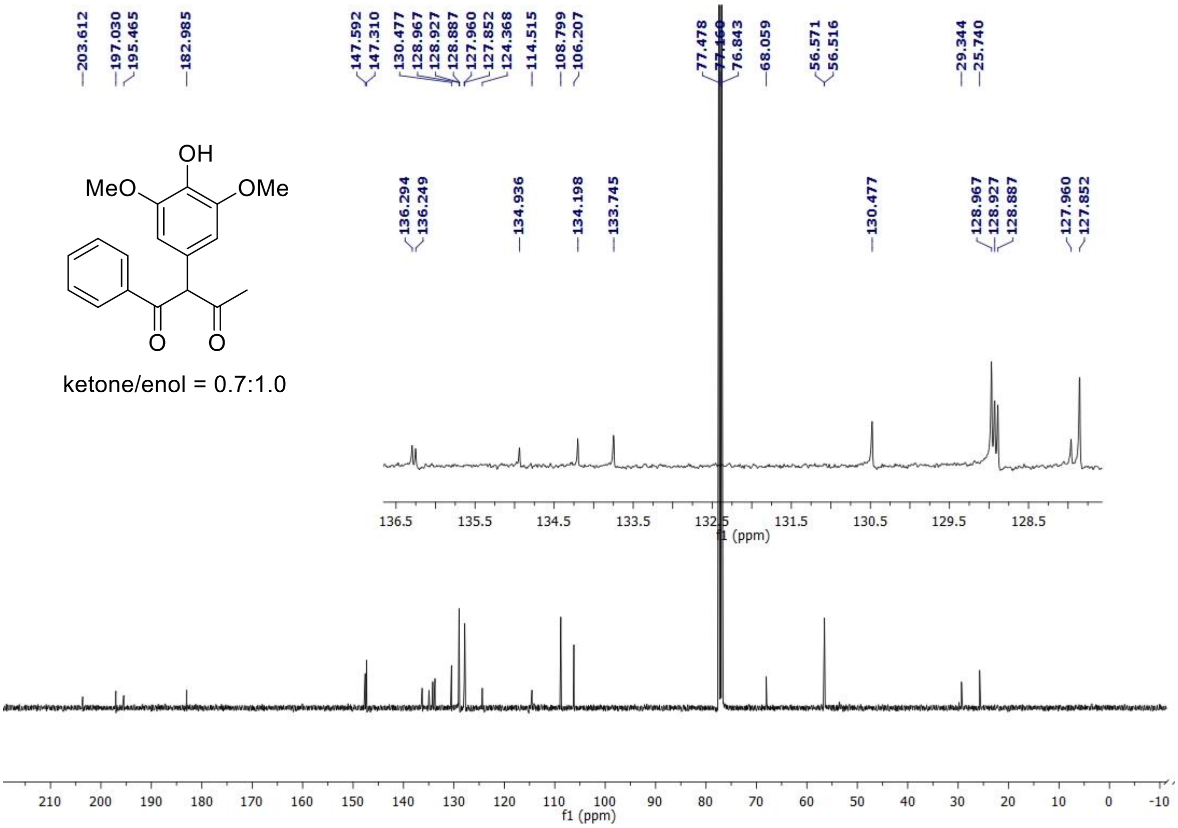

ketone/enol = 0.7:1.0

6e ^{1}H NMR (400 MHz, $CDCl_3$)

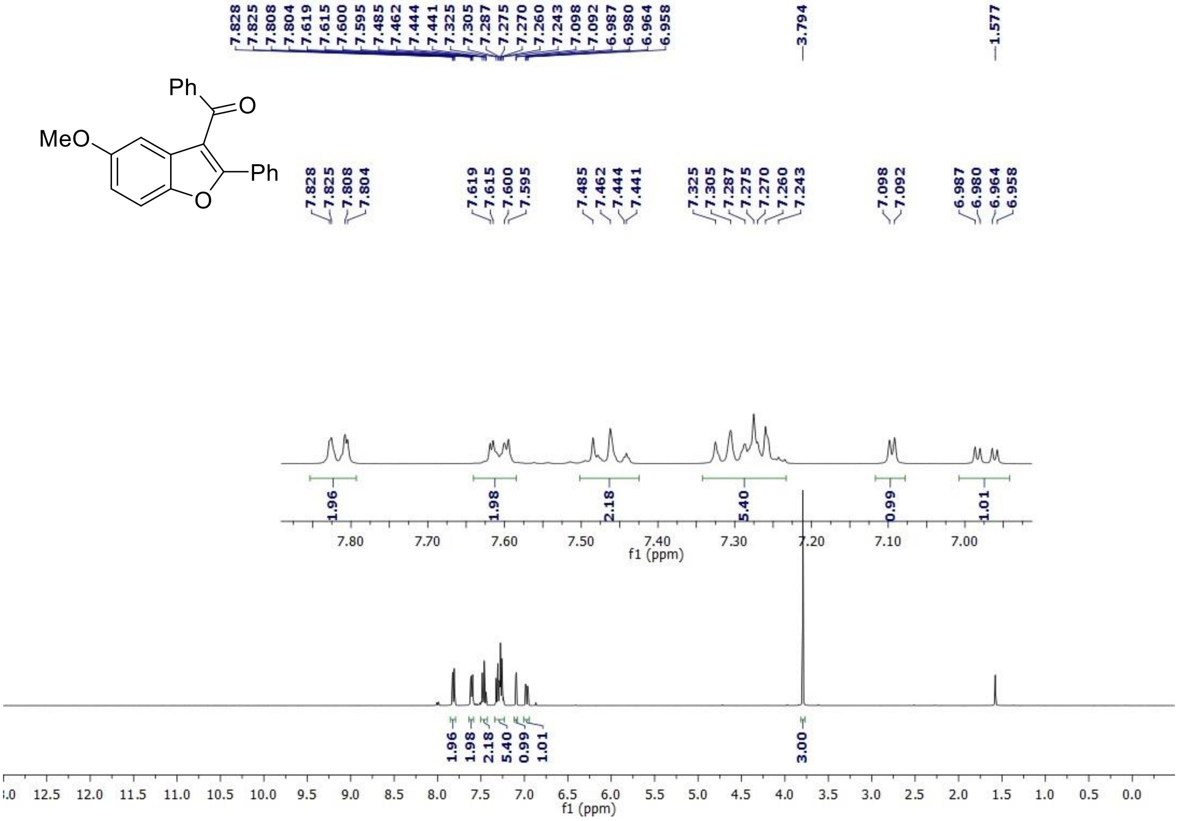

6e ^{13}C NMR (100 MHz, $CDCl_3$)

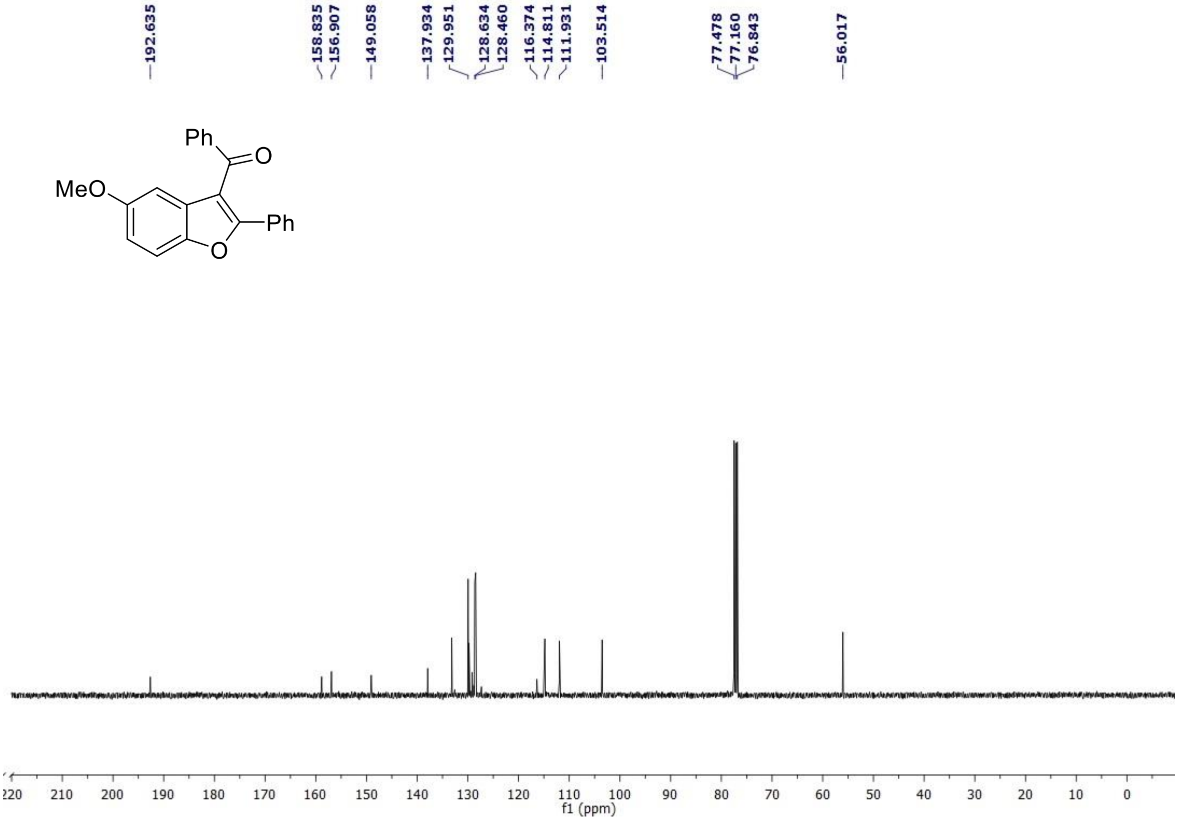

6f ^{1}H NMR (400 MHz, $CDCl_3$)

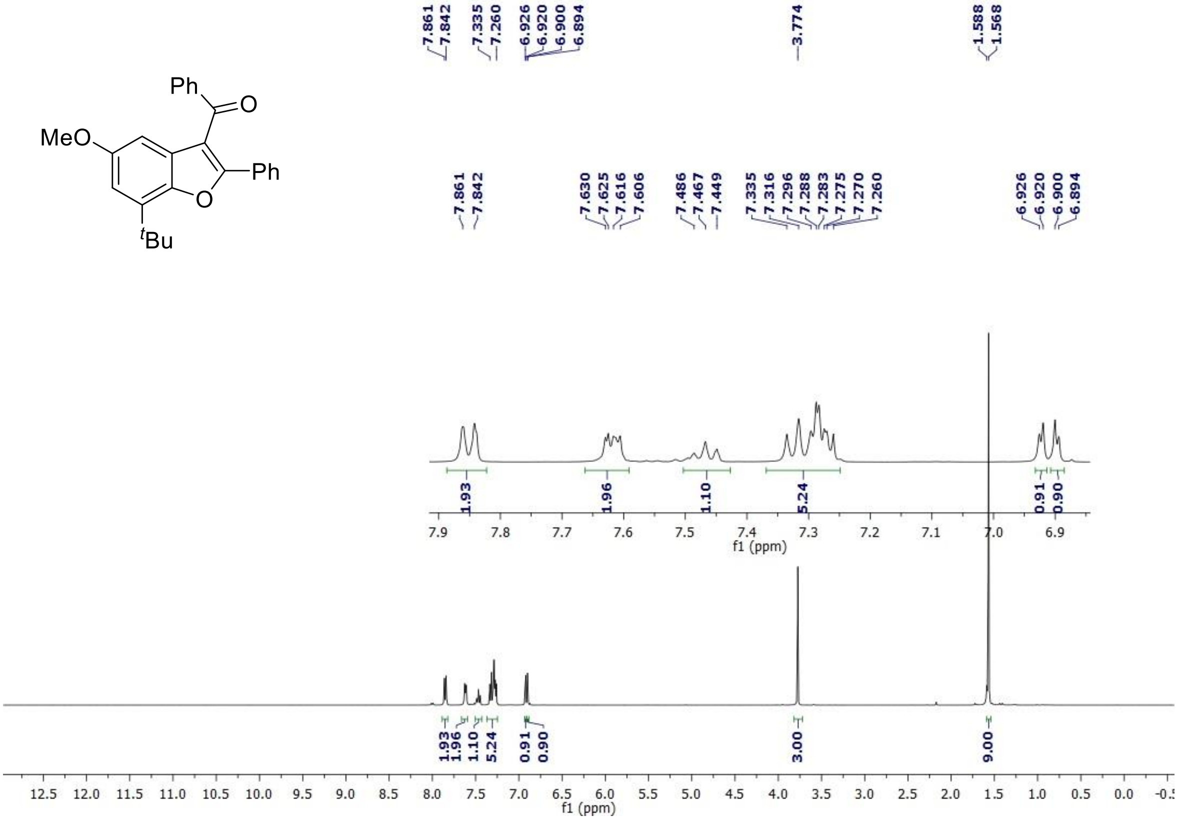

6f ^{13}C NMR (100 MHz, $CDCl_3$)

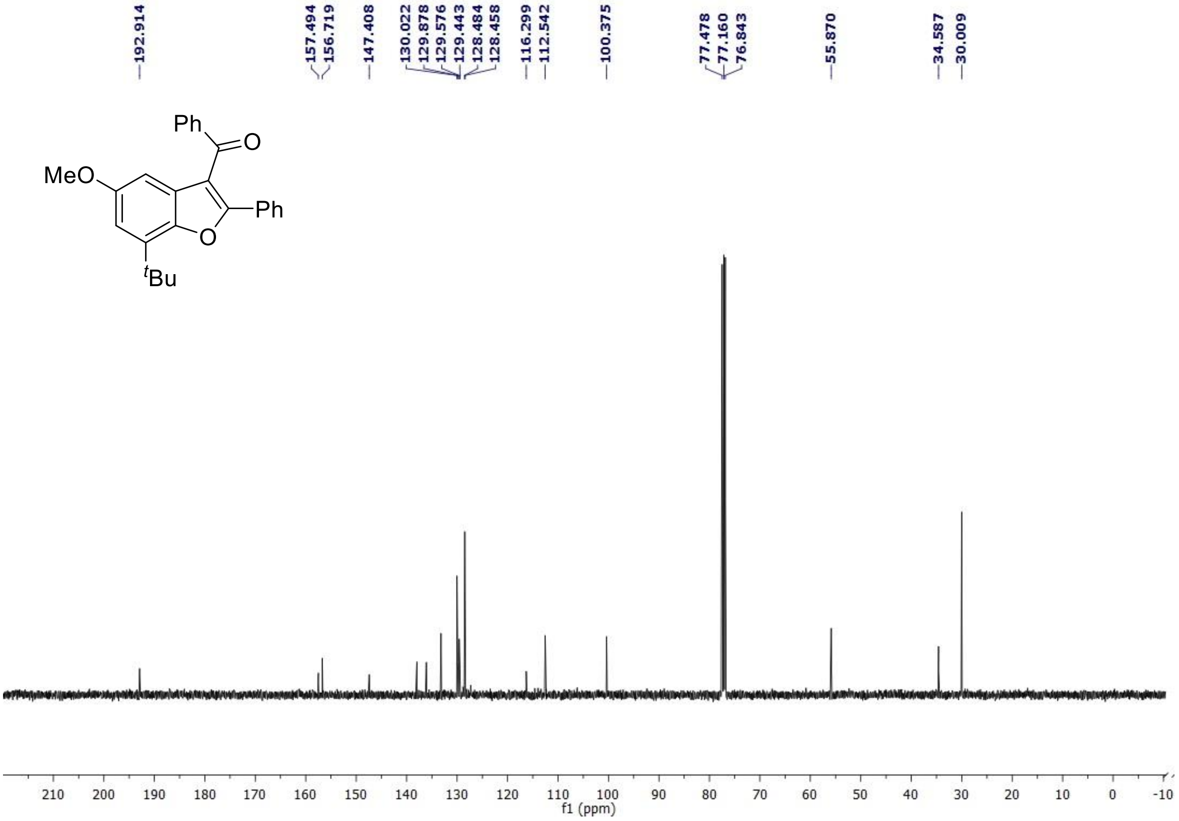

6g ^{1}H NMR (400 MHz, $CDCl_3$)

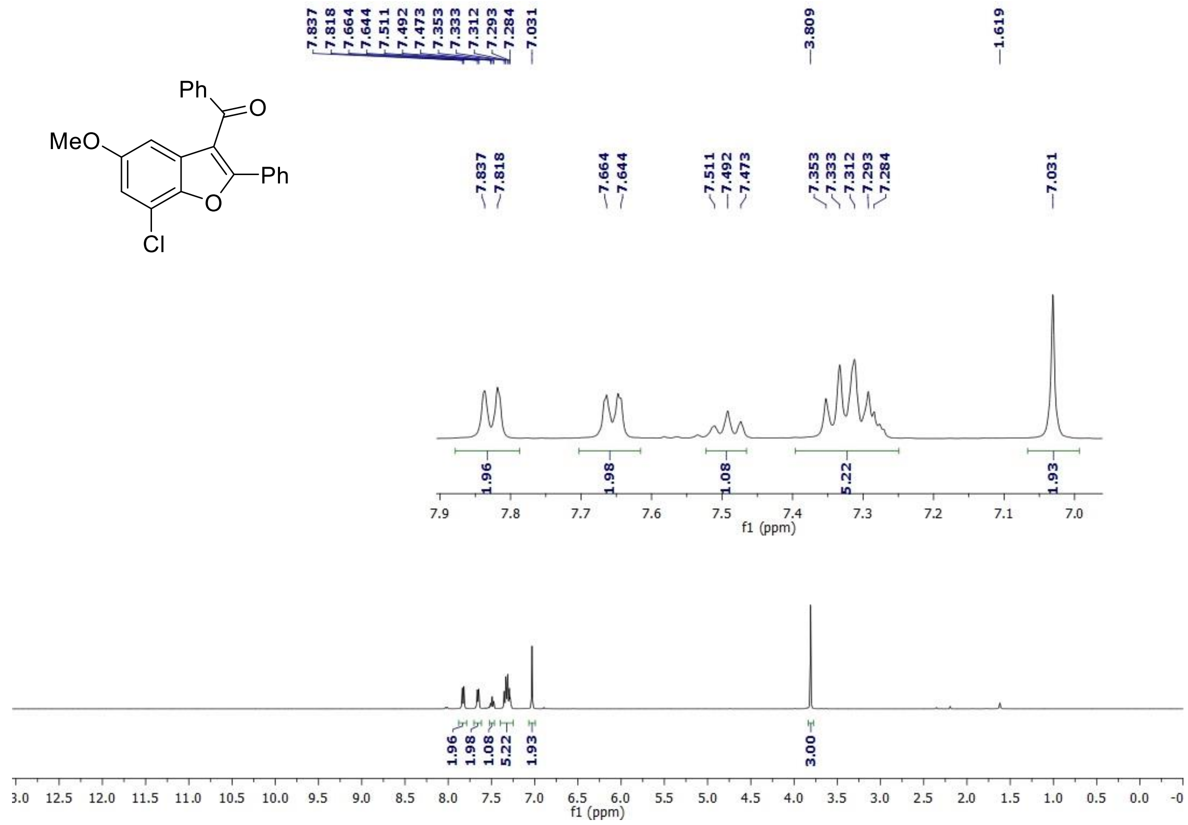

6g ^{13}C NMR (100 MHz, $CDCl_3$)

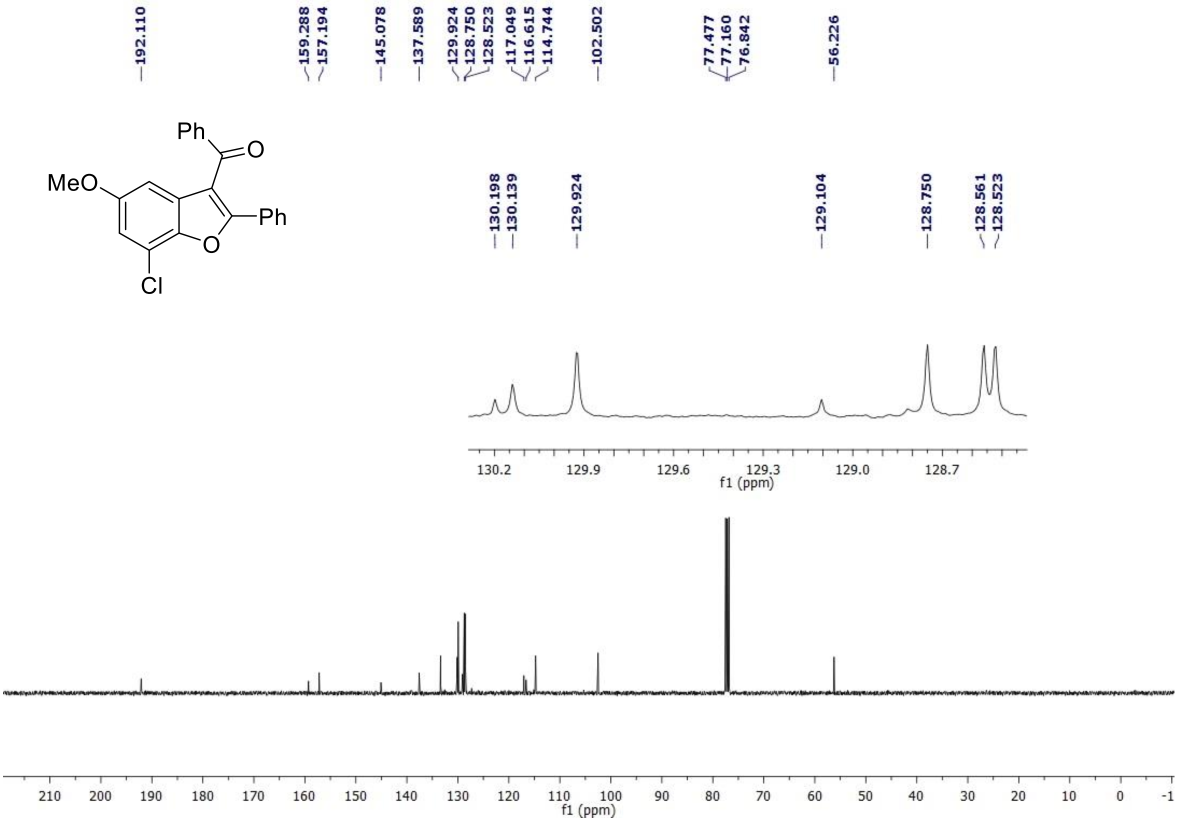

7a ^{1}H NMR (400 MHz, $CDCl_3$)

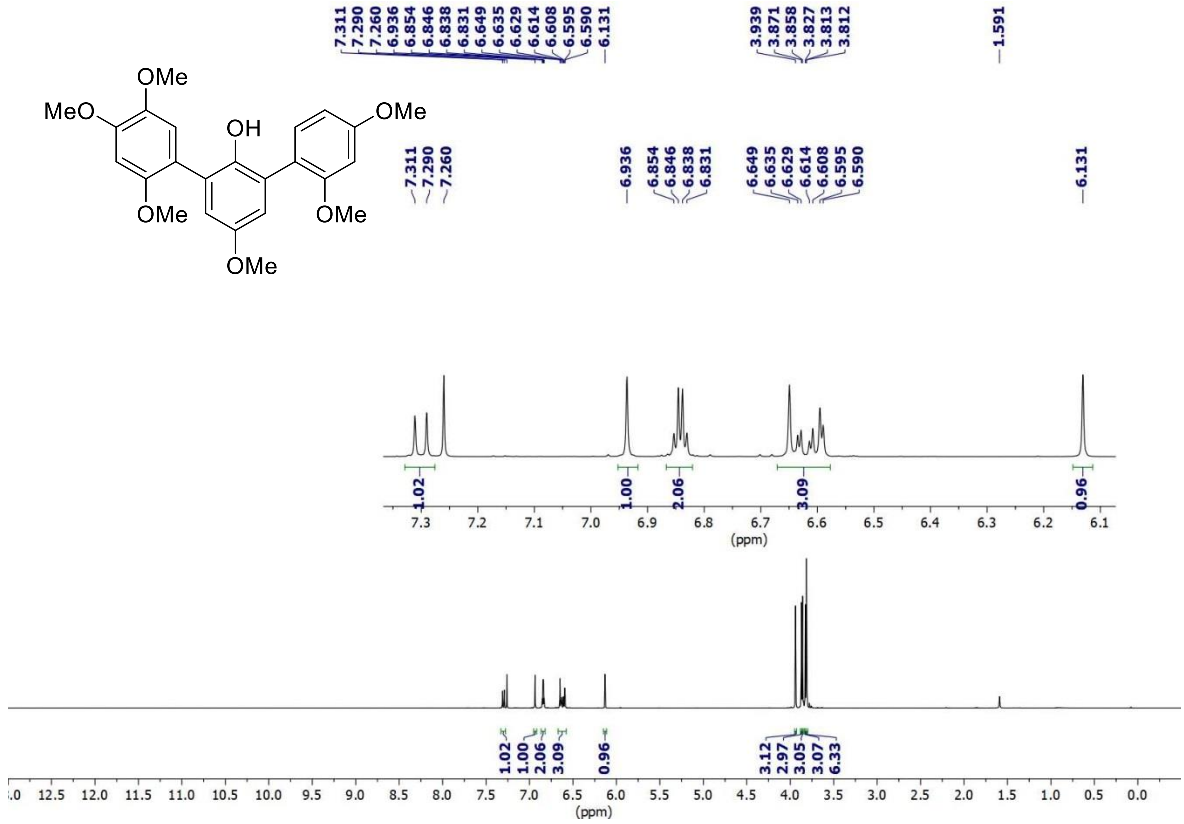

7a ^{13}C NMR (100 MHz, $CDCl_3$)

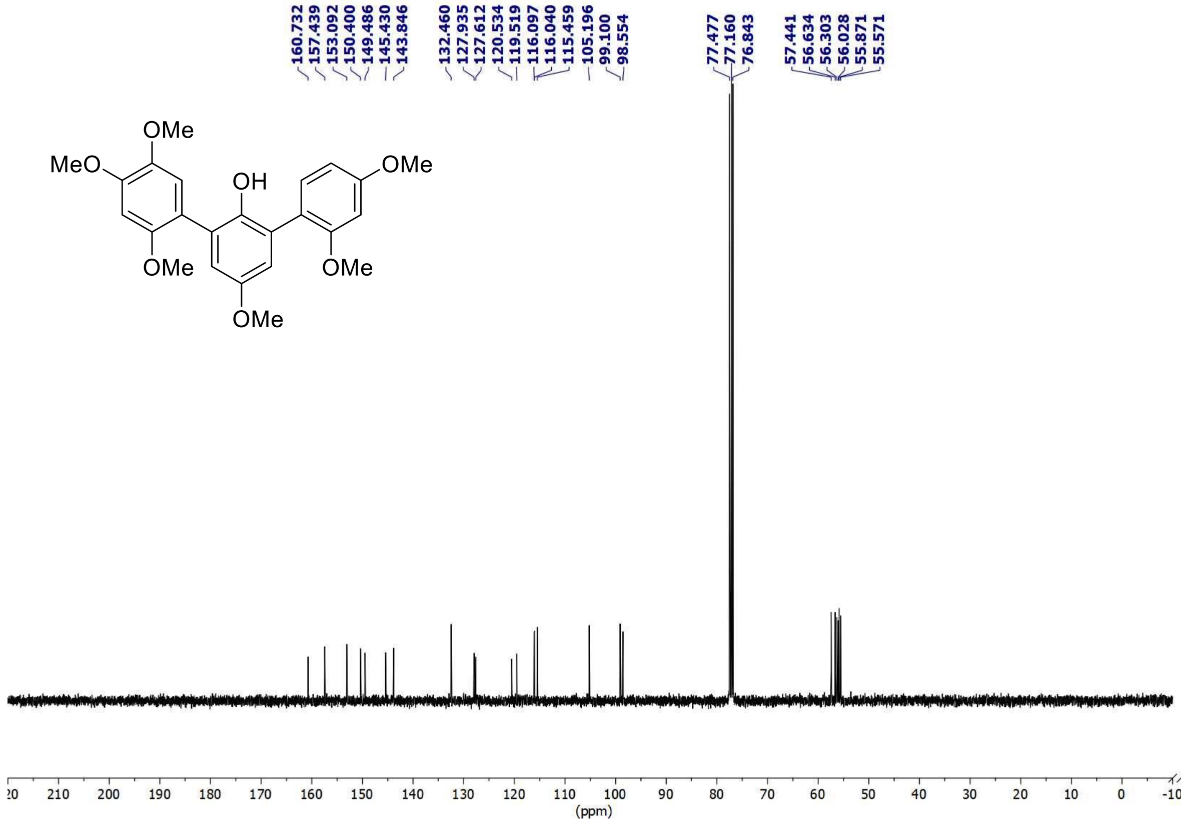

7b ^{1}H NMR (400 MHz, $CDCl_3$)

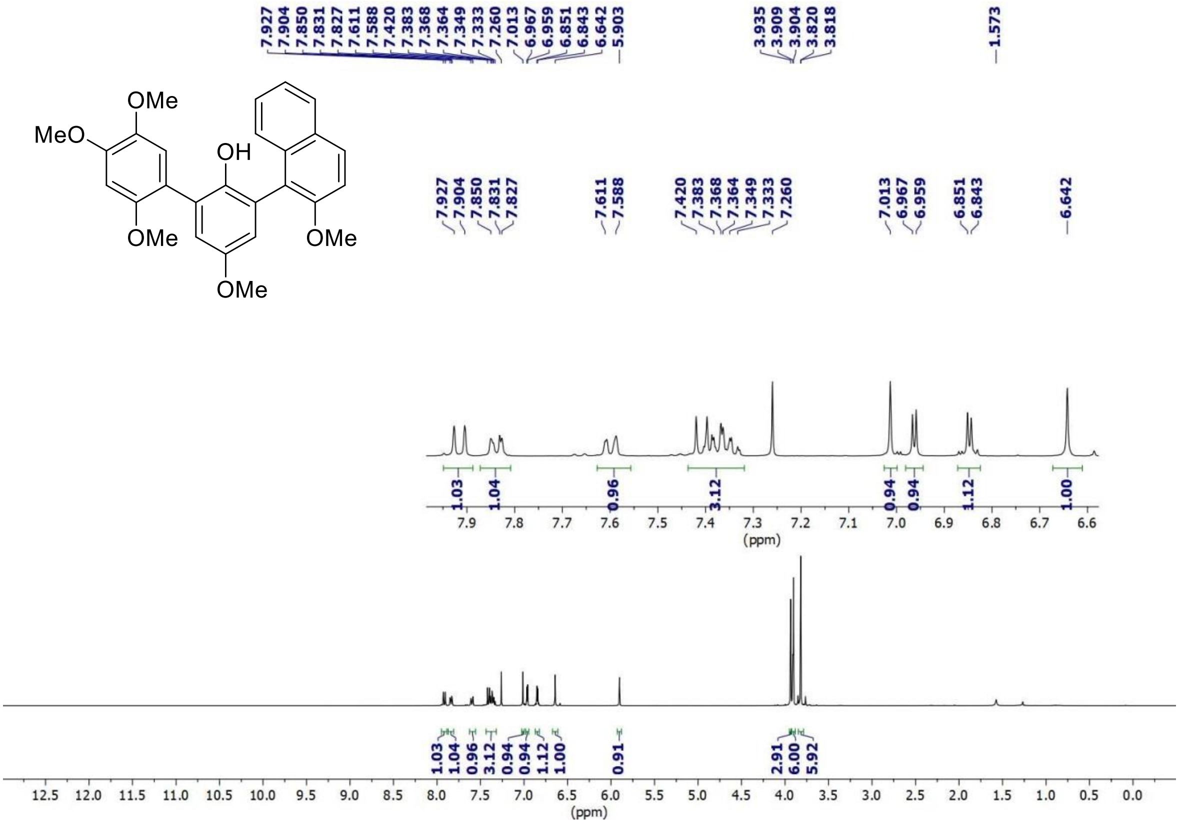

7b ^{13}C NMR (100 MHz, $CDCl_3$)

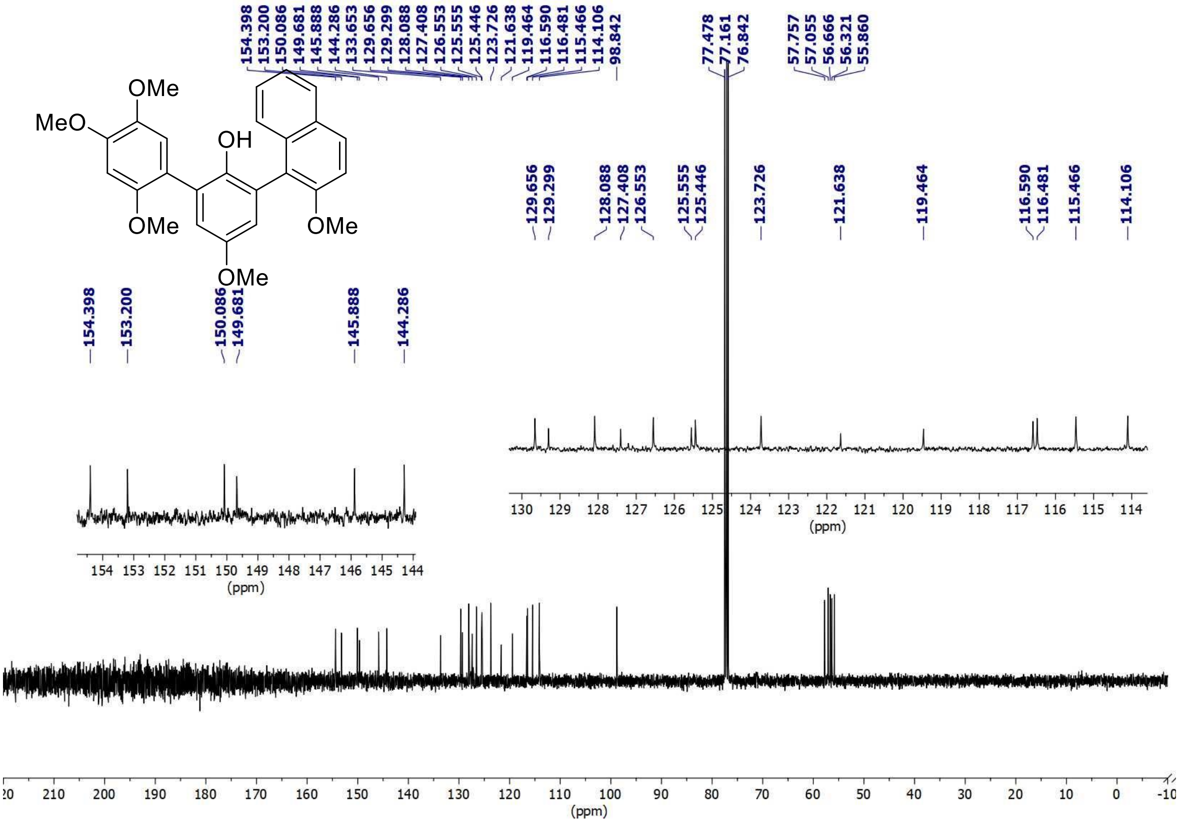

7c ^{1}H NMR (400 MHz, CDCl$_3$)

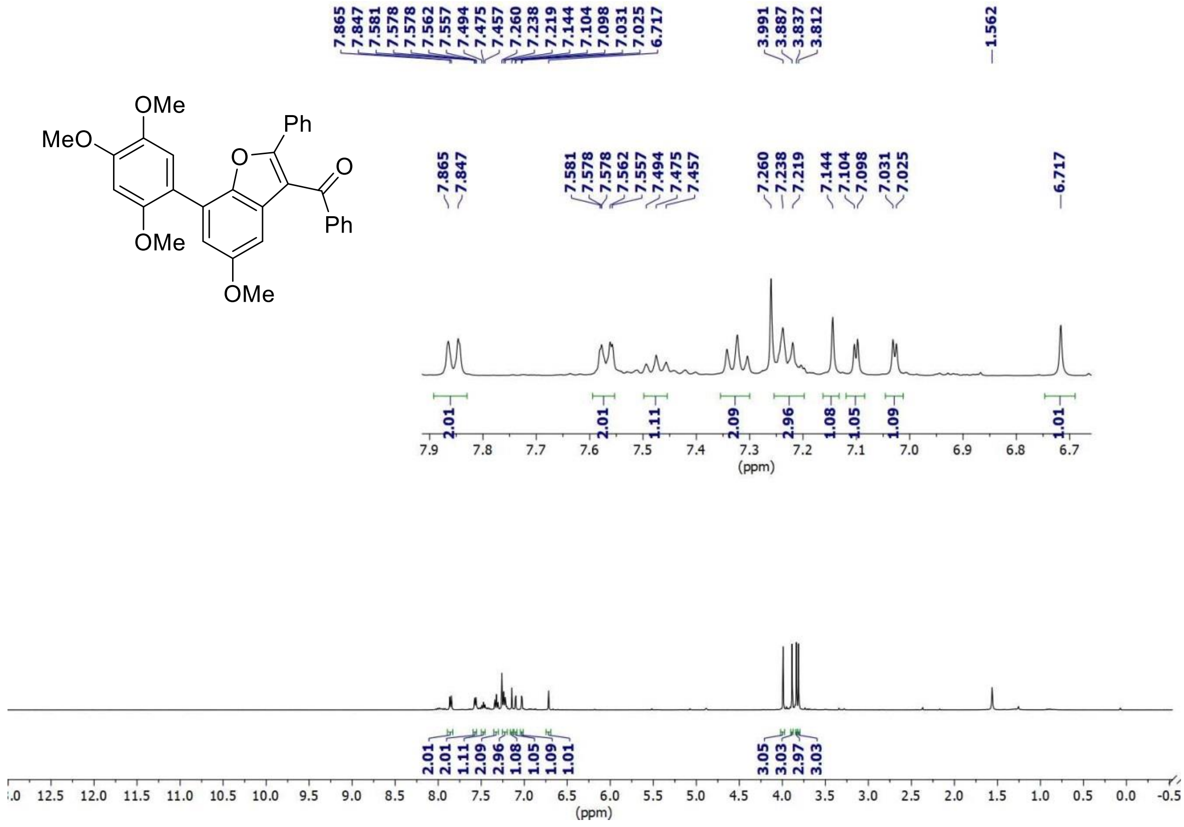

7c ^{13}C NMR (100 MHz, CDCl$_3$)

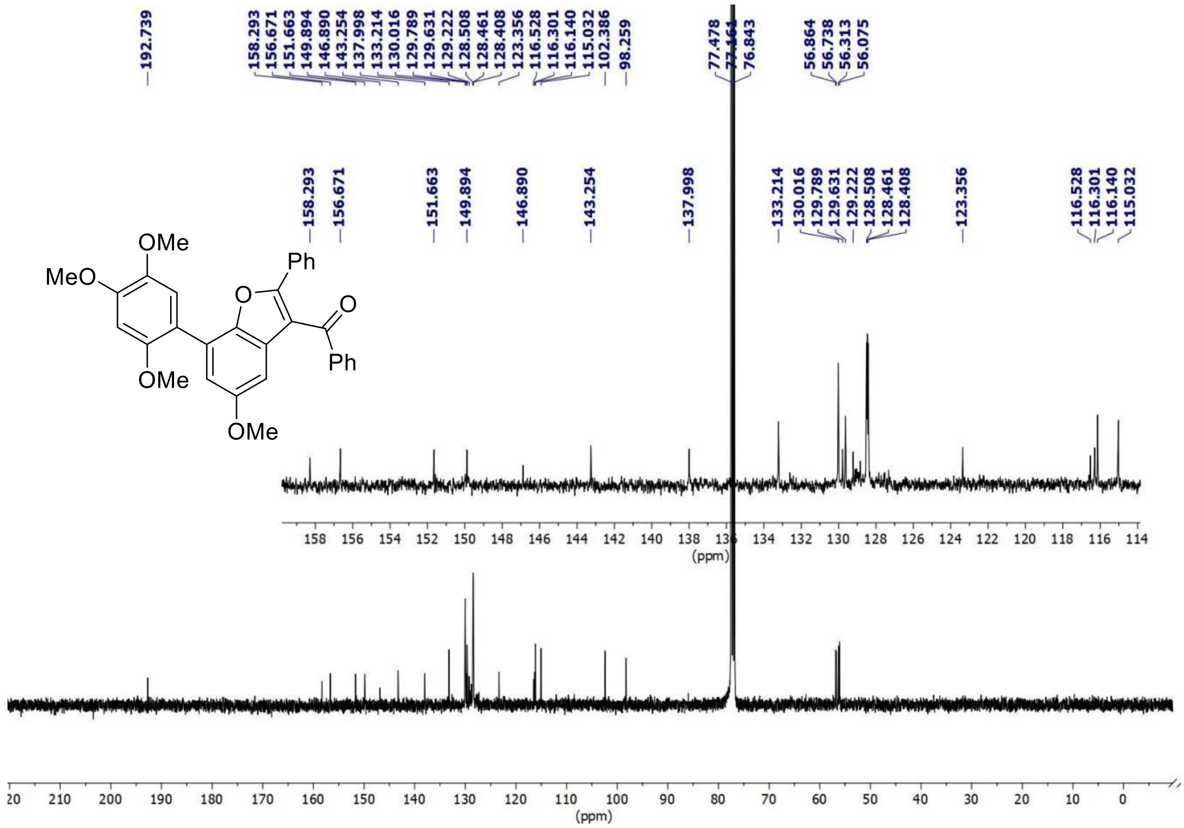

7d ^{1}H NMR (400 MHz, $CDCl_3$)

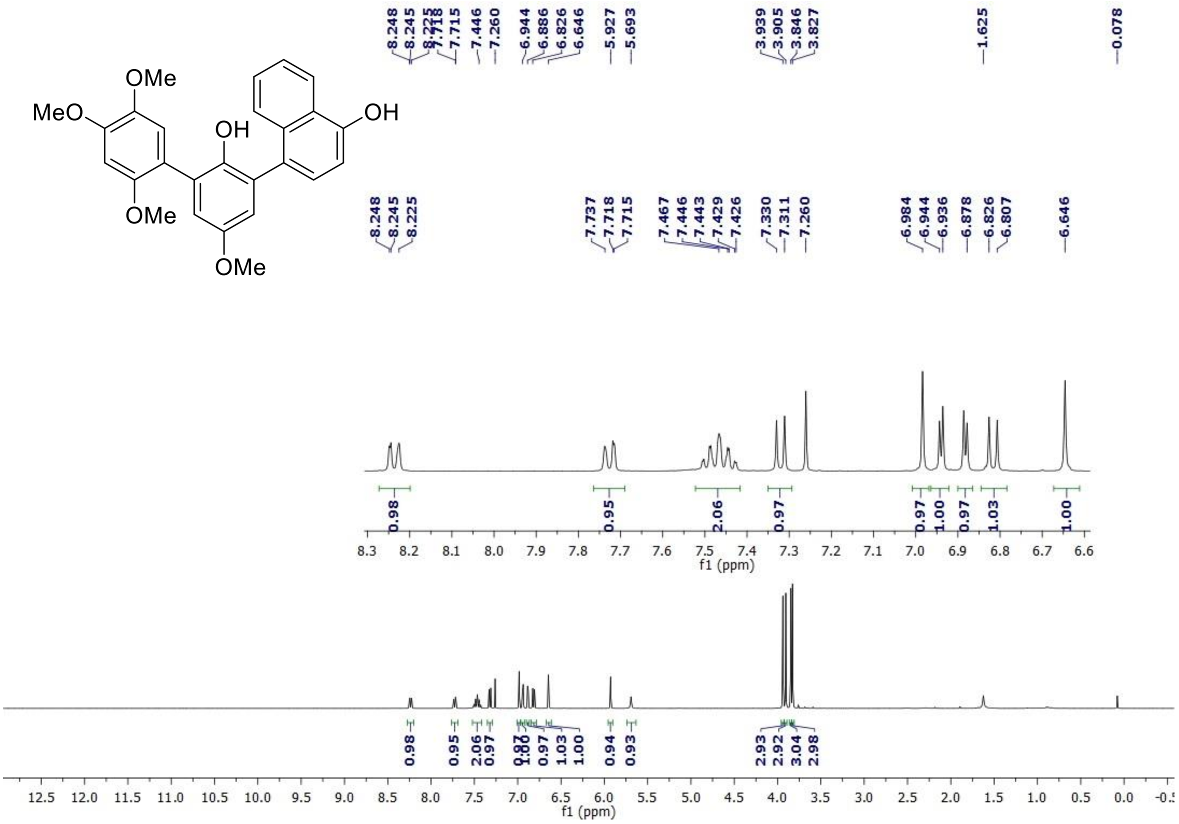

7d ^{13}C NMR (100 MHz, $CDCl_3$)

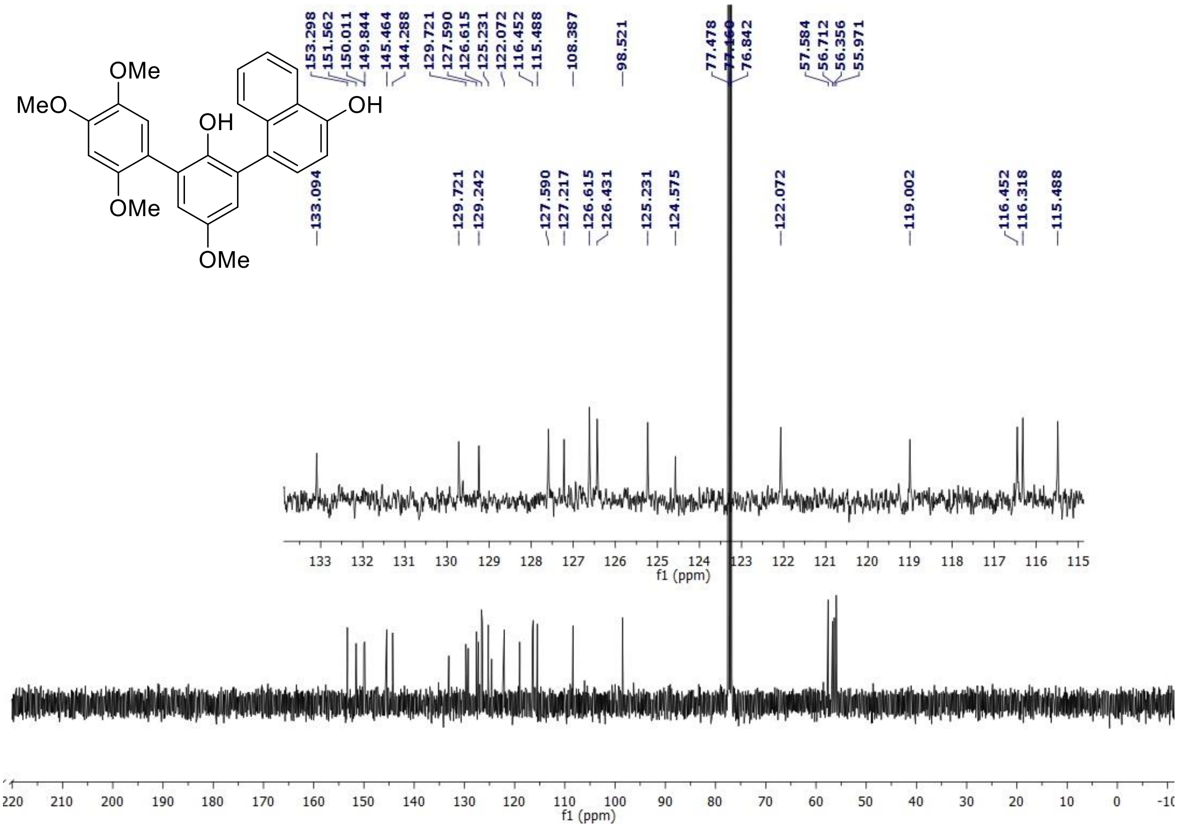

7e ^{1}H NMR (400 MHz, $CDCl_3$)

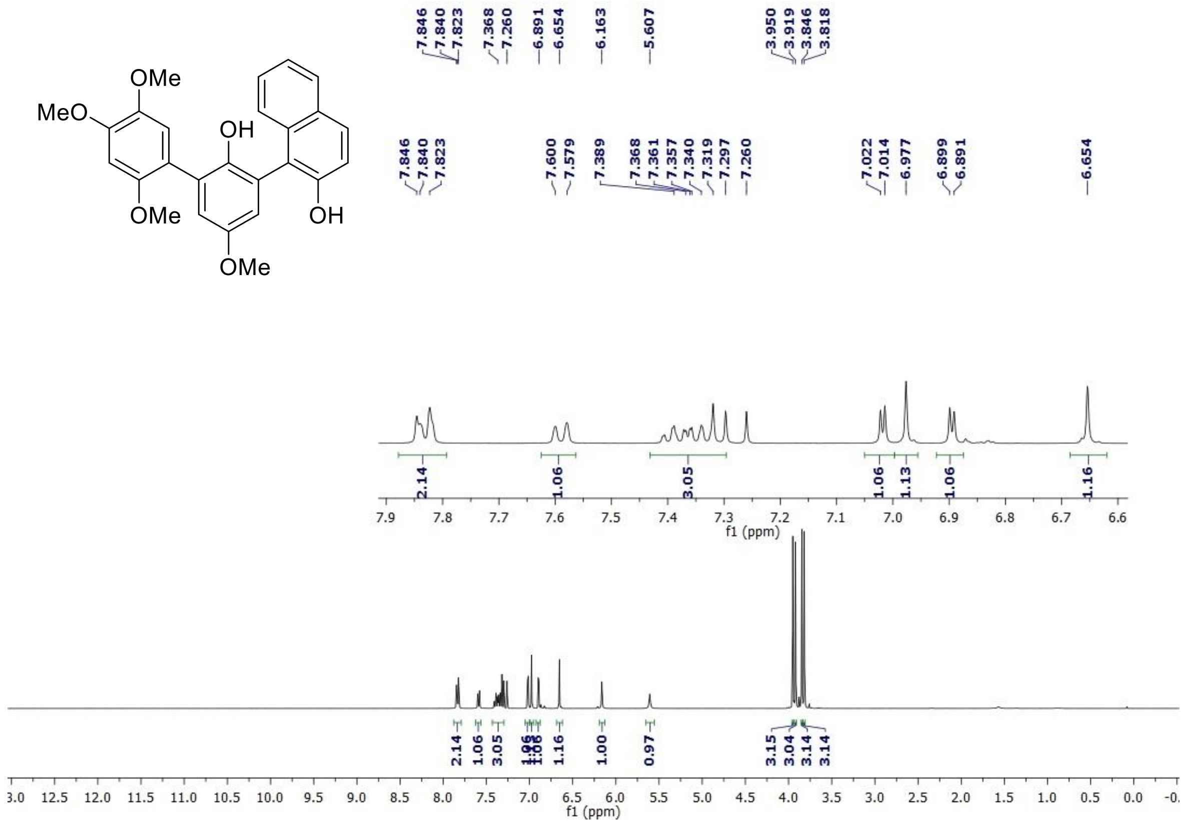

7e ^{13}C NMR (100 MHz, $CDCl_3$)

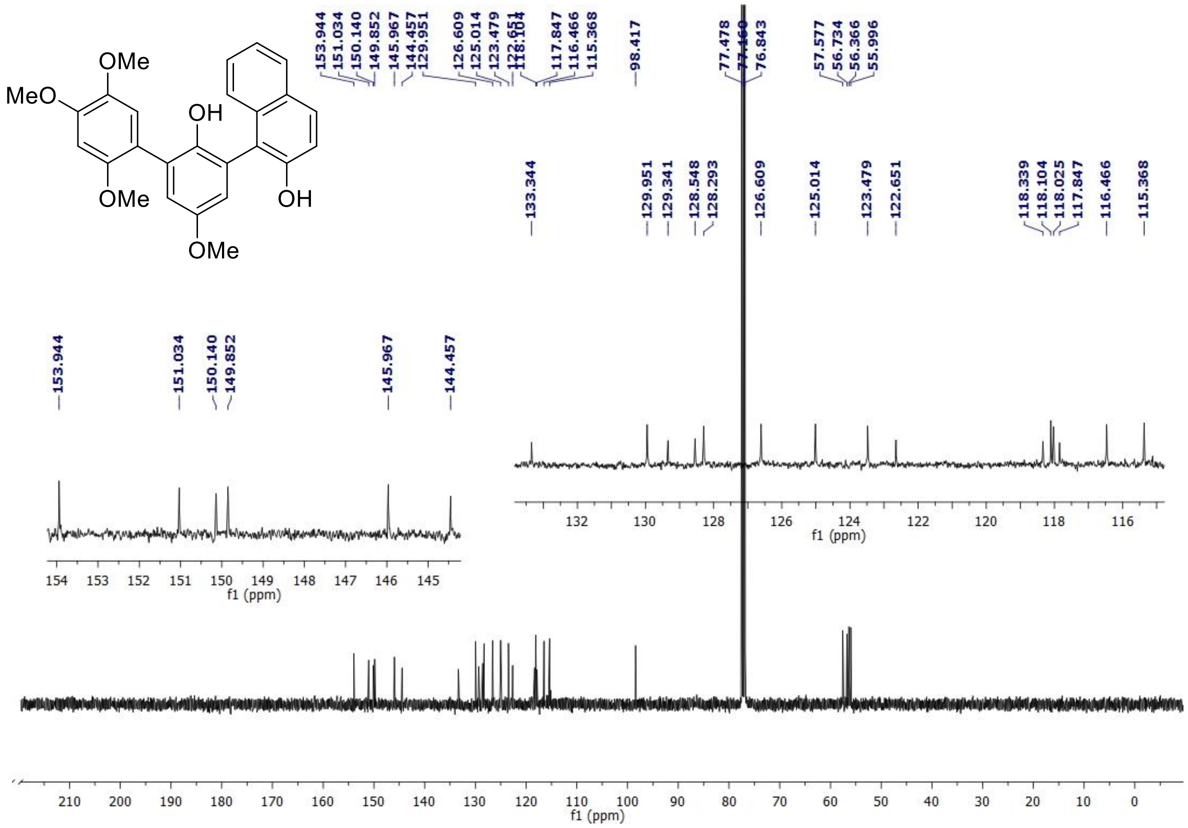

7f ^{1}H NMR (400 MHz, $CDCl_3$)

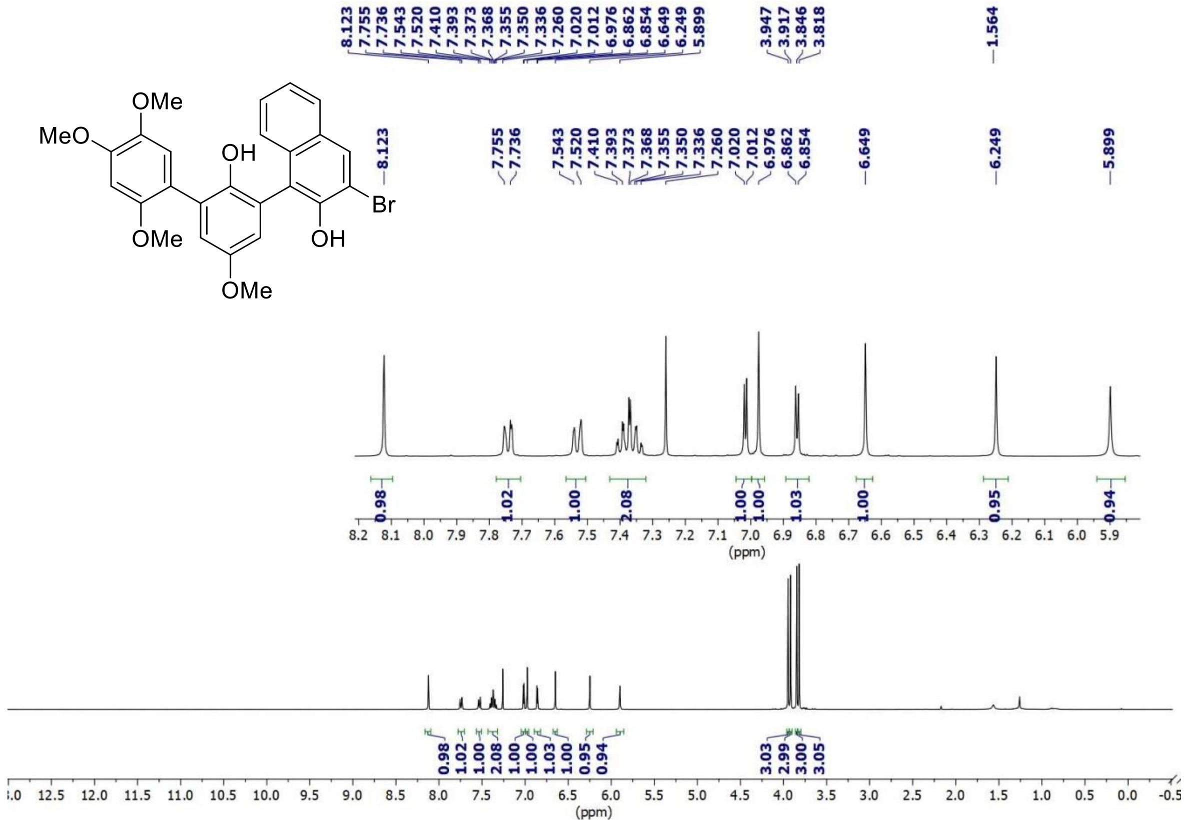

7f ^{13}C NMR (100 MHz, $CDCl_3$)

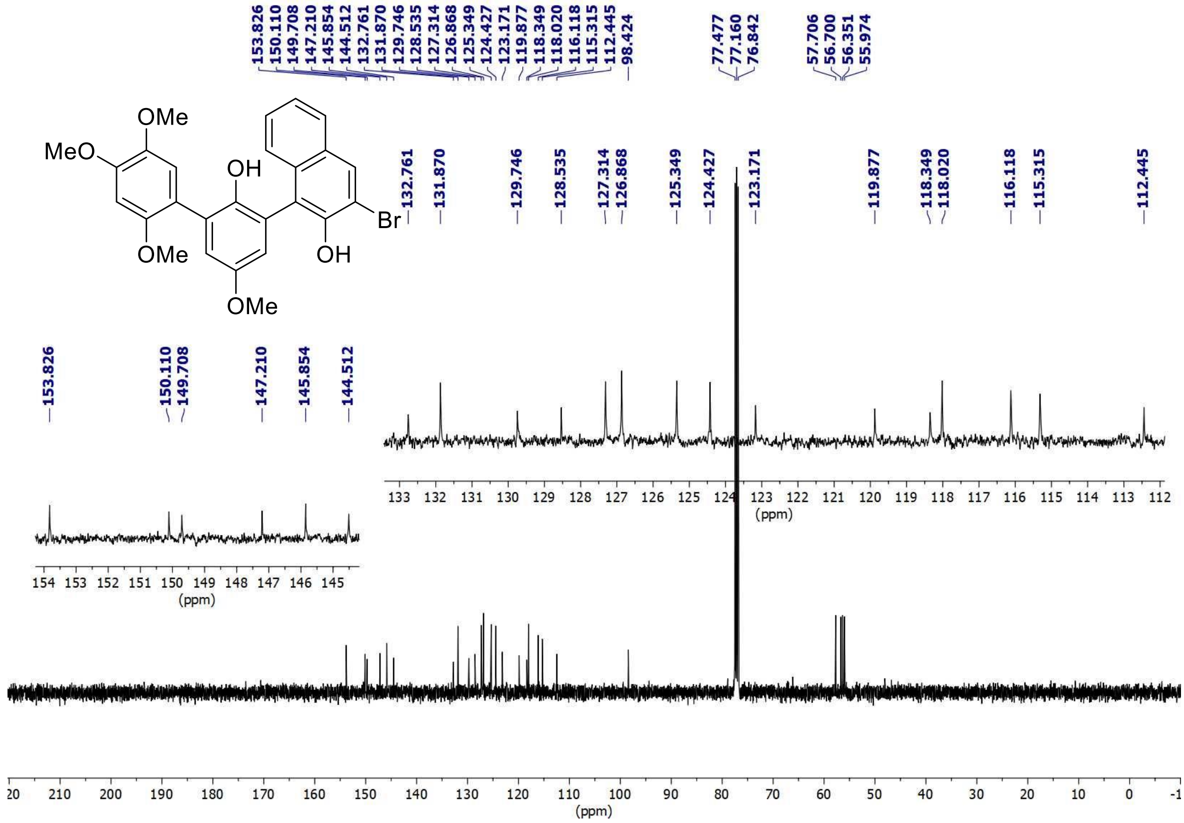

7g ^{1}H NMR (400 MHz, $CDCl_3$)

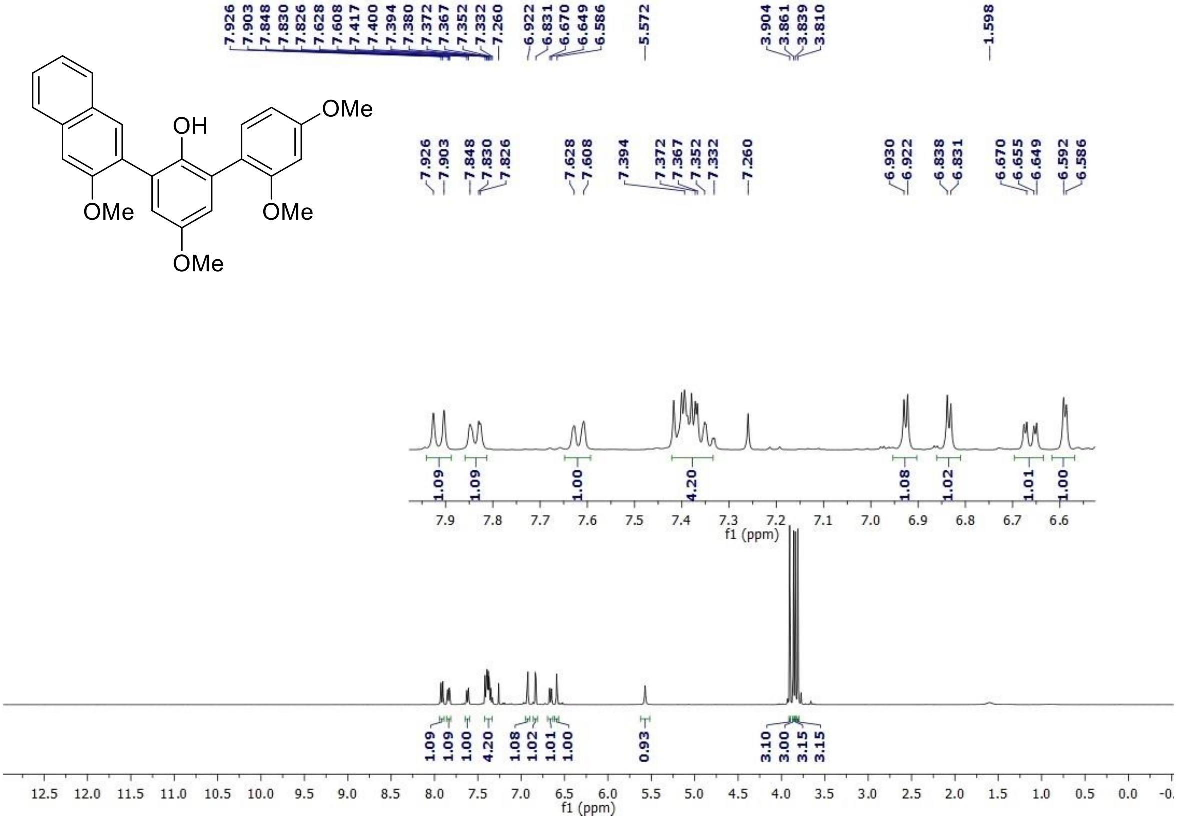

7g ^{13}C NMR (100 MHz, $CDCl_3$)

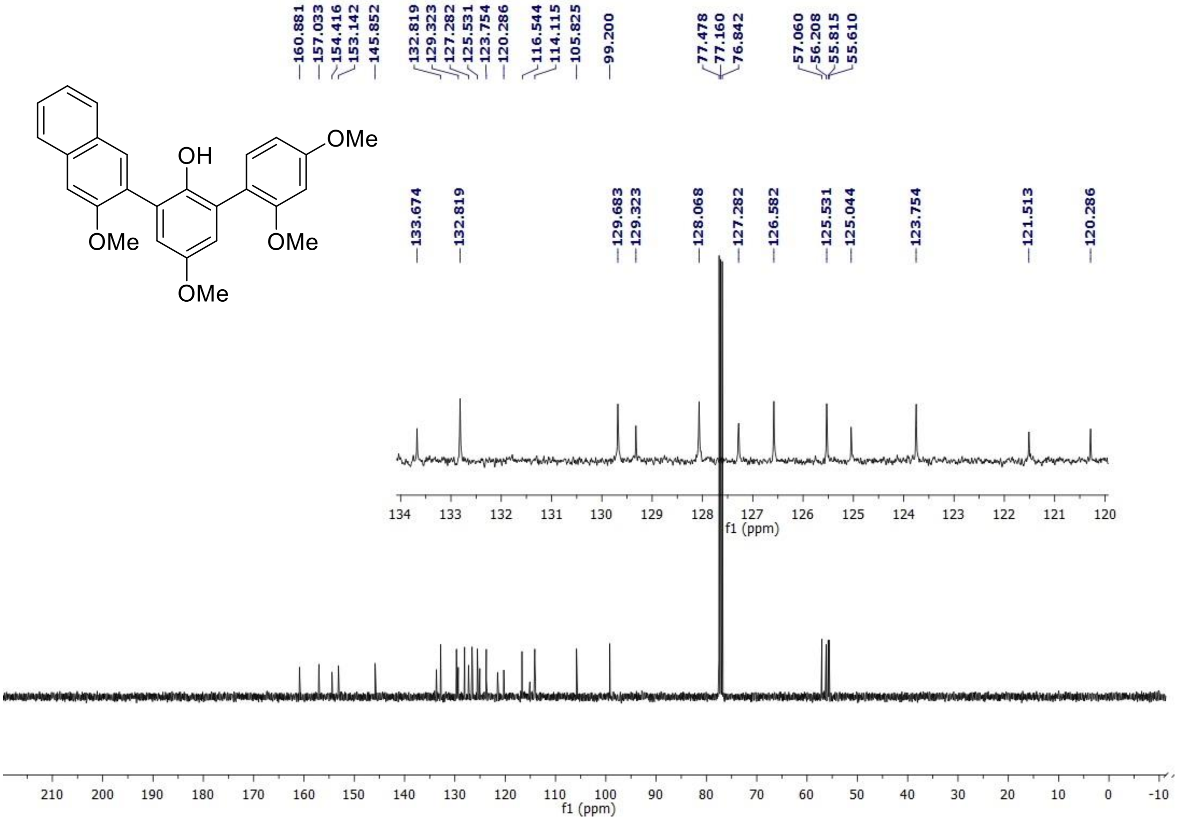

7h ^{1}H NMR (400 MHz, $CDCl_3$)

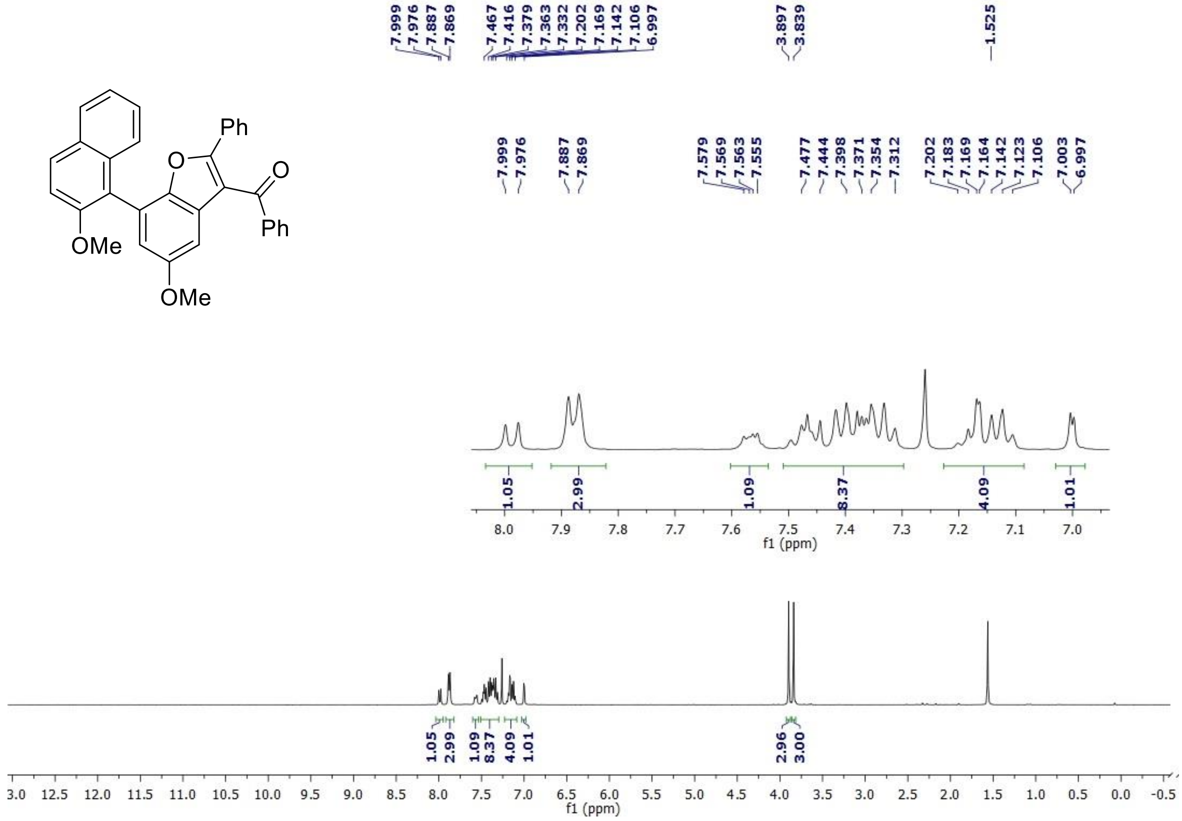

7h ^{13}C NMR (100 MHz, $CDCl_3$)

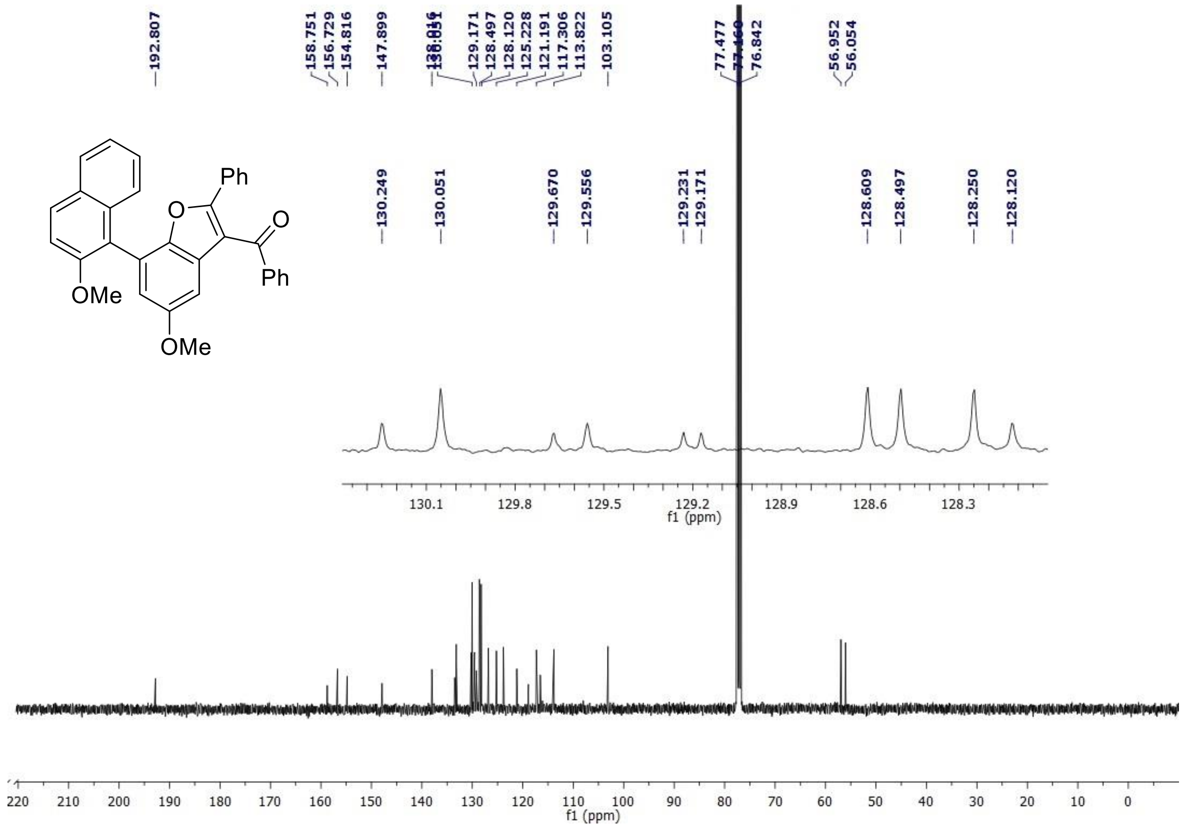

Supplement: SC-011-C9SC05668H-s001 [file SC-011-C9SC05668H-s001.pdf]
